# Ice in biomolecular cryocrystallography

**DOI:** 10.1107/S2059798321001170

**Published:** 2021-03-30

**Authors:** David W. Moreau, Hakan Atakisi, Robert E. Thorne

**Affiliations:** a Physics Department, Cornell University, Ithaca, NY 14853, USA

**Keywords:** protein crystallography, ice, stacking disorder, structure-factor error

## Abstract

Diffraction data acquired from cryocooled protein crystals often include diffraction from ice. Analysis of ice diffraction from crystals of three proteins shows that the ice formed within solvent cavities during rapid cooling is comprised of a stacking-disordered mixture of hexagonal and cubic planes, with the cubic plane fraction increasing with increasing cryoprotectant concentration and increasing cooling rate. Building on the work of Thorn and coworkers [Thorn *et al.* (2017 ▸), *Acta Cryst.* D**73**, 729–727], a revised metric is defined for detecting ice from deposited protein structure-factor data, and this metric is validated using full-frame diffraction data from the Integrated Resource for Reproducibility in Macromolecular Crystallography. Using this revised metric and improved algorithms, an analysis of structure-factor data from a random sample of 89 827 PDB entries collected at cryogenic temperatures indicates that roughly 16% show evidence of ice contamination, and that this fraction increases with increasing solvent content and maximum solvent-cavity size. By examining the ice diffraction-peak positions at which structure-factor perturbations are observed, it is found that roughly 25% of crystals exhibit ice with primarily hexagonal character, indicating that inadequate cooling rates and/or cryoprotectant concentrations were used, while the remaining 75% show ice with a stacking-disordered or cubic character.

## Abstract 2

Analysis of diffraction data from three proteins indicates that the ice formed in internal crystal solvent is stacking-disordered. Application of a revised metric and algorithm for detecting ice from protein structure-factor data indicates that roughly 3.9% of PDB entries exhibit ice that is primarily hexagonal and 11.7% exhibit stacking-disordered ice.

## 1. Introduction   

Ice diffraction frequently contaminates diffraction data collected from biomolecular crystals at cryogenic temperatures (Rupp, 2009 ▸; Pflugrath, 2015 ▸). Ice may form during crystal cooling in solvent present within solvent cavities in the crystal (Moreau *et al.*, 2019 ▸) or in residual solvent on the crystal surface (Garman & Mitchell, 1996 ▸). Ice may also appear as contaminating frost on the sample or sample-holder surface, due to exposure to moist ambient air during handling or data collection, or from accumulated frost in the liquid nitrogen used to initially cool and to store the crystals (Pflugrath, 2004 ▸). Ice that forms from solvent confined to the solvent cavities of the crystal, from bulk-like solvent containing substantial cryoprotectant or from bulk-like solvent that is rapidly cooled is typically highly polycrystalline, producing continuous and largely isotropic ice rings. Ice that forms from bulk-like solvent containing little cryoprotectant or that is cooled slowly tends to be comprised of fewer, larger crystals, producing ‘lumpy’, anisotropic, quasi-continuous diffraction rings. Frost is typically comprised of an even smaller number of larger dendritic crystals.

When only a small number of large ice crystals are present in the X-ray beam (as is often the case with frost), ice diffraction may manifest as discrete, isolated ice diffraction peaks. When such a peak overlaps with a protein diffraction peak, the measured intensity will be larger than those of symmetry-related reflections and/or will be larger than that predicted from Wilson statistics (Blessing, 1997 ▸). This allows them to be identified as outliers and rejected by standard diffraction-frame merging or scaling software (Read, 1999 ▸).

When many ice crystals are present in the X-ray beam, the resulting continuous or quasi-continuous diffraction rings will overlap with protein Bragg peaks and interfere with the background-subtraction process when the Bragg peaks are integrated. The integration procedure sums the pixel values selected as being associated with the Bragg peak, which includes X-ray counts from diffraction and scattering sources other than the long-range ordered component of the protein crystal. For each individual protein crystal Bragg peak, background-subtraction algorithms estimate the X-ray counts from these sources based on pixel values that are near to but not associated with that Bragg peak. Each integration program estimates the background counts from these pixel values in a slightly different manner. *XDS* assumes a constant background beneath each protein Bragg peak equal to the average value of the neighbouring pixels (Kabsch, 2010 ▸), *MOSFLM* fits a plane to the neighbouring pixels (Leslie, 2006 ▸) and *DIALS* provides options to model the logarithm of the background as a constant or a plane (Parkhurst *et al.*, 2016 ▸). When the ice rings are narrow in comparison with the box in which the background is measured, the background is no longer adequately modelled by a constant value or by a linear plane. In the case of a direct overlap, the ice diffraction will be weaker or absent in the neighbouring pixels used to estimate the background. The estimated background will then be considerably smaller than the true background, leading to an overestimate of the true value of the protein Bragg peak. If the ice ring is near to but does not overlap with the protein Bragg peak, it can still adversely affect the background-estimation procedure. Ice diffraction in neighbouring pixels used to estimate the background will be stronger than ice diffraction overlapping the Bragg peak. This leads to an overestimate of the background and, in turn, to an underestimate of the protein Bragg peak. Parkhurst *et al.* (2017 ▸) illustrates these two scenarios in their Fig. 2 and thoroughly explain how ice rings lead to incorrect estimates of the background.

A background-subtraction algorithm providing much improved management of ice rings, the Global Background Model (GBM), has been developed and implemented within the *DIALS* package (Parkhurst *et al.*, 2017 ▸). Instead of a constant or planar background, the GBM method performs a pixel-by-pixel average of the background across all diffraction frames in a data set (excluding those frames where the pixel is part of a protein Bragg peak, but including frames where it is part of ice diffraction), median-filters these averaged values in azimuthal rings at each resolution and then scales this average background image to match the observed background around each individual Bragg reflection during integration of that Bragg reflection. The use of a single scaling parameter (for each Bragg reflection) allows fast integration and may prevent overfitting of the background. This algorithm greatly reduces ice-related biasing of ice-ring intensities. It works particularly well when the ice diffraction has the form of homogeneous, isotropic rings and less well when the ice rings are ‘lumpy’ or in general have a structure that varies azimuthally and between frames.

Here, we examine the nature and effects of ice on diffraction data in protein cryocrystallography. We first examine ice diffraction in diffraction frames that we have collected from crystals of three proteins and in reference diffraction-frame data obtained from the Integrated Resource for Reproducibility in Macromolecular Crystallography (IRRMC; Grabowski *et al.*, 2016 ▸). Fits to ice diffraction from ice formed inside protein crystals indicate that the ice is not purely hexagonal, purely cubic or a simple mixture of the two. Instead, the predominant form of ice is a stacking-disordered mixture of cubic and hexagonal planes, with the cubic fraction increasing from ∼50% with increasing cryoprotectant concentration (Moreau *et al.*, 2019 ▸). In contrast, ice diffraction from ice formed in drops of aqueous cryoprotectant solutions at lower cryoprotectant concentrations, lower cooling rates and/or at higher temperatures has a primarily hexagonal character. As the cryoprotectant concentration increases, the cooling rate increases and/or the temperature of ice formation decreases, the diffraction develops a stacking-disordered character with an increasing cubic fraction.

We then extend the methods developed by Thorn *et al.* (2017 ▸) for detecting the presence of ice based on experimental protein structure-factor data alone (*i.e.* without access to the full diffraction frames). Our approach obtains more accurate ice detection with far fewer false positives and false negatives and is based on a *p*-value testing the null hypothesis that the structure factors are not biased by ice diffraction. Using our revised ice-detection metric and improved algorithms, we find that roughly 16% of PDB entries show structure-factor perturbations from ice contamination, consistent with the results of Thorn and coworkers. Of these, roughly 25% show evidence of ice contamination at the positions of all-hexagonal ice peaks, and for the remaining 75% the observed peaks are consistent with stacking-disordered ice with a substantial cubic fraction.

## 2. Methods   

### 2.1. Crystal growth and preparation   

Crystallization conditions for equine spleen apoferritin, thaumatin and tetragonal hen egg-white lysozyme were as described in Moreau *et al.* (2019 ▸) and are given in Supplementary Section S1. Crystals were used as-grown, or else were soaked in solutions containing 10, 20 or 40%(*v*/*v*) glycerol for at least 5 min. Crystals were then transferred to a separate drop of NVH oil (Cargille) and manipulated until all external solvent was removed from their surface, as indicated by a near-disappearance of the crystal due to the close match between its refractive index and that of the oil (Warkentin & Thorne, 2009 ▸). Crystals were mounted on microfabricated loops in a spherical blob of NVH oil to prevent dehydration during data collection and stored in MicroRT tubes (MiTeGen) containing mother liquor or cryoprotectant solution for ∼1 h (to produce crystals with consistent, handling-independent hydration) prior to data collection.

### 2.2. X-ray diffraction data collection   

X-ray data were collected from cryocooled protein crystals on station F1 at the Cornell High-Energy Synchrotron Source (CHESS) using a PILATUS 6M detector. A cold nitrogen-gas stream (Oxford Cryosystems Cryostream 700) with a flow rate of 5 l min^−1^ and programmed to the desired final sample temperature was directed at the crystal. The cold gas stream was initially blocked using an air-blade shutter, each crystal was placed in the X-ray beam at room temperature and ten frames totalling 5° in rotation were collected to assess the crystal for damage and dehydration. The gas stream was then unblocked, cooling the crystal to its final temperature in ∼1 s or less. Additional diffraction data at *T* = 100 K were collected from crystals plunge-cooled into liquid nitrogen.

X-ray diffraction data from cryocooled glycerol/water solutions were collected using the same CHESS station and experimental protocols. Samples were prepared by injecting ∼10 nl of solution into a long thin-walled polyester tube of 250 µm in diameter and ∼2 cm in length, resulting in a ∼200 µm long plug of liquid. The tube was then affixed to a goniometer base (MiTeGen GB-B1A) and centred in the X-ray beam. Supplementary Fig. S1 shows an image of the sample in the beam path.

### 2.3. Ice diffraction-peak identification   

Discrete ice diffraction peaks, associated with diffraction from a single ice crystal, were identified by searching for pixels that were located near expected ice-ring positions and were not associated with protein diffraction, having values approximately five times larger than the local background intensity. The detailed algorithm used is described in Supplementary Section S2.

### 2.4. Ice diffraction modelling   

Ice within and on the surface of protein crystals may be hexagonal (I_h_), cubic (I_c_), a mixture of these crystal forms (I_h_ + I_c_) or stacking-disordered (I_sd_), in which cubic and hexagonal planes are randomly stacked, as shown in Supplementary Fig. S2 [adapted from Fig. 7 in Moreau *et al.* (2019 ▸) and Fig. 9 in Malkin *et al.* (2015 ▸)]. Diffraction images collected at CHESS and 22 sets of raw diffraction images showing clear visual evidence of ice obtained from the IRRMC archive (Supplementary Section S3), including 13 IRRMC sets used by Parhurst *et al.* (2017 ▸), were fitted with models of stacking-disordered ice or of a mixture of cubic and hexagonal ice using the methods described in Moreau *et al.* (2019 ▸).

Model fitting used the methods described in Moreau *et al.* (2019 ▸). Diffraction images were loaded into Python using *FabIO* (Knudsen *et al.*, 2013 ▸), and the *pyFAI* integration package (Ashiotis *et al.*, 2015 ▸) was used to remove the protein Bragg peaks and azimuthally average the frames within resolution bins. Powder diffraction patterns of stacking-disordered ice were generated using *DIFFaX* (Treacy *et al.*, 1991 ▸). The model consists of (001) planes of hexagonal ice randomly stacked with (111) planes of cubic ice. The probability of a cubic plane being followed by a hexagonal plane is Φ_ch_ and that of a hexagonal plane being followed by a cubic plane is Φ_hc_ (Kuhs *et al.*, 2012 ▸; Malkin *et al.*, 2015 ▸). These stacking probabilities, the unit-cell parameters and the instrumental broadening were optimized to fit the *DIFFaX* models to the observed azimuthally averaged diffraction using the *scipy.optimize.minimize* program in the SciPy Python library (Virtanen *et al.*, 2020 ▸). The atomic *B* factors were fixed to 1.5 Å^2^ in all cases. The background was determined by evaluating a tenth-order polynomial fit to the difference *I*_bg_(*q*) = *I*_exp_(*q*) − *I*_*DIFFaX*_(*q*) between the *DIFFaX* models and our experimental diffraction patterns.

Azimuthally averaged diffraction versus resolution-bin data were also fitted assuming pure hexagonal ice, pure cubic ice and a mixture of hexagonal and cubic ice crystallites, using the same methods as implemented to fit the stacking-disordered ice diffraction. For pure hexagonal ice Φ_hh_ and Φ_cc_ were fixed to 1 and 0, respectively, and for pure cubic ice Φ_hh_ and Φ_cc_ were fixed to 0 and 1, respectively, and the unit-cell and broadening parameters were allowed to vary during optimization. For the mixture of hexagonal and cubic ice, a linear combination of pure hexagonal and cubic ice diffraction patterns generated using equivalent unit-cell and broadening parameters were optimized to fit the observed diffraction. No parameters were held fixed between cases.

### 2.5. Estimation of ice-crystallite sizes   

The size of ice crystallites formed within a protein crystal was estimated from the data of Moreau *et al.* (2019 ▸) using the observed ice diffraction-ring breadths after azimuthal averaging. Prior to freezing, all external solvent was removed from each protein crystal in this data set. Any observed ice then formed from solvent that was within the interior of the protein crystal, and not on its surface, prior to cooling.

If an ice crystal has a finite size, its diffraction peaks are radially broadened by an amount inversely proportional to the crystal size. As crystal size decreases, the breadth, β, of the diffraction peaks in 2θ increases according to Scherrer’s equation,
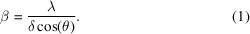
Here, the breadth of a peak is estimated as the integrated area beneath the peak divided by its maximum amplitude (Stokes & Wilson, 1942 ▸), λ is the wavelength, θ is the Bragg angle and δ is the apparent size of the crystallite. The actual crystallite size is proportionally related to the apparent crystallite size by Scherrer’s constant (Langford & Wilson, 1978 ▸), which tends to be close to one.

Strain, dislocations and planar faults can generate additional peak broadening (Baker, 2002 ▸; Thürmer & Bartelt, 2008 ▸). Limited prior knowledge of their character and distribution prevents accurate modelling of their contribution to the observed broadening (Ungár *et al.*, 1998 ▸). However, their contribution increases more rapidly with angle than that due to crystallite size. For this reason, only the lowest angle Bragg reflection not broadened by stacking disorder, the (002) reflection of hexagonal ice, was used to estimate crystallite size.

The raw measured peak breadths β_raw_ include contributions from instrumental broadening. This arises primarily from the dispersion and divergence of the X-ray source. Additional broadening of 1D ice-ring widths obtained by azimuthal averaging of 2D detector images arises from errors in defining the experimental geometry. To estimate the magnitude of the source broadening β_source_, a polycrystalline hexagonal ice sample with a large grain size was prepared, so that the powder diffraction-peak breadths were dominated by source effects (Supplementary Fig. S3). To minimize beam-geometry errors, the assumed beam centre and the angle between the incident X-ray beam direction and the detector normal were optimized to minimize the sum of the breadths of the observed ice rings. The corrected breadths of the hexagonal ice calibrant are shown in Supplementary Fig. S4 and have an average of β_source_ = 0.037°, which is on a par with expectations based on the known divergence and dispersion of the X-ray source.

For protein crystals used in this analysis, raw experimental peak breadths were determined from 1D diffraction patterns as the integrated area beneath the peak divided by its maximum amplitude. The beam centre and detector tilt were optimized to minimize these raw measured breadths. The broadening of the observed (detector-angle and beam centre position corrected) ice diffraction peaks is a convolution of the intrinsic and instrumental broadenings. The increase in breadth of a peak following a convolution depends on the shape of the functions involved. The peaks of the hexagonal ice calibrant were Gaussian and the peaks of the ice associated with protein crystals were Lorentzian. The appropriate subtraction formula to determine the intrinsic peak broadening is given by Olivero & Longbothum (1977 ▸),
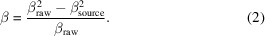
Additional details of our methods and the analysis used to estimate ice-crystallite size are given in Supplementary Section S4.

### 2.6. Analysis of deposited PDB structure factors for ice   

Following the procedure used by Thorn *et al.* (2017 ▸) in their *AUSPEX* ice-detection method, we developed a statistical algorithm to calculate a *p*-value testing the null hypothesis that there is no biasing of the integrated Bragg peaks from ice diffraction. Here, we provide an overview of our approach. Additional details are given in Supplementary Section S5. A Python script implementing our algorithm is also included in the supporting information.

Our algorithm is based on two separate metrics that compare the distribution of measured protein structure factors at the expected 2θ values/resolutions of ice diffraction with those observed near to but off the ice peaks. These metrics monitor changes in the mean structure-factor intensity and the fraction of structure factors with low intensities. A *p*-value is formulated based on these metrics. Experimenters sometimes exclude structure factors at resolutions near the ice rings, and in this case the PDB-deposited structure factors should show no ice biasing. To flag these deposited data sets, a second *p*-value is formulated to test the null hypothesis that Bragg peaks are not excluded from ice-ring regions based on a third metric that tracks the number of measured structure factors.

#### 2.6.1. Ground-truth data   

The first 1200 IRRMC depositions with resolutions better than 3.5 Å were used to create a ground-truth data set. The first 1000 entries were used as a training set to calibrate the ice-detection algorithm. Thumbnail diffraction images from the IRRMC webpage and a scatter plot of the corresponding PDB-deposited integrated intensities versus resolution were inspected. If ice rings were visible in the diffraction images or the integrated intensities appeared biased due to ice, the data set was flagged as containing ice. Diffraction frames for entries in the training set that produced false positives or negatives with the final algorithm were downloaded for closer inspection and reclassified if needed. The last 200 entries served as a test set to gauge the performance of the algorithm through estimated false positives and negatives. To ensure correct classification in this test set, diffraction images for each entry were downloaded for visual inspection as opposed to viewing thumbnails. The training and test data sets had 194 and 60 entries, respectively, that were classified as containing ice. When scored solely on observed structure-factor biasing, 34 entries in the test data set were classified as containing ice.

#### 2.6.2. Ice Finder Score   

**Figure 1:**
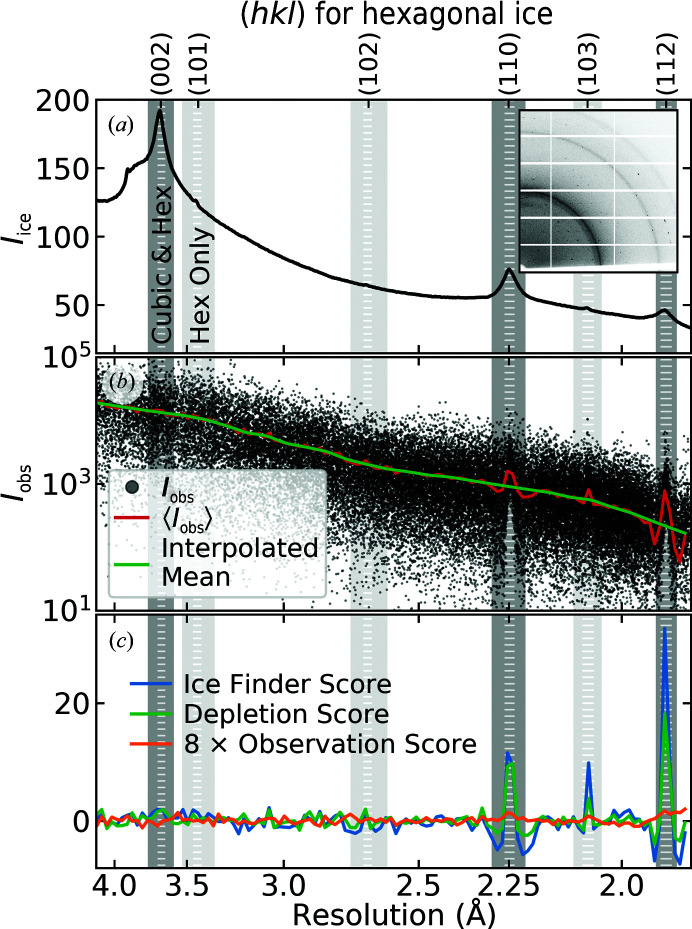
Identification of ice in the deposited structure factors of PDB entry 4h3w. (*a*) The 2D diffraction pattern and azimuthally averaged 1D background suggest that the ice is primarily stacking-disordered, I_sd_. The resolution ranges examined for structure-factor biasing using our Ice Contamination Score, corresponding to shared peak positions for hexagonal ice I_h_, cubic ice I_c_ and stacking-disordered ice I_sd_, are indicated by darker vertical shading. The resolution ranges examined to establish whether hexagonal ice is present, corresponding to I_h_ peak positions that are absent from I_c_ and that are strongly suppressed in I_sd_ for typical cubic/hexagonal stacking fractions, are indicated by lighter vertical shading. In both cases, the shaded regions represent the interpolation range; the regions marked by vertically stacked horizontal lines represent the range searched for evidence of ice contamination. (*b*) Measured diffraction-peak intensities integrated by *XDS* from PDB entry 4h3w at *T* = 100 K. The red line shows the average *I*_obs_ value in bins of width 0.0025 Å^−1^. The green line shows the mean intensities calculated from coarser bins and then interpolated between bins and through the dark vertically shaded regions. (*c*) Ice Finder, Depletion and Observation Scores calculated for this data set.

Fig. 1 ▸(*a*) (inset) shows a single diffraction frame for PDB entry 4h3w taken from the IRRMC. Fig. 1 ▸(*b*) shows a scatter plot of the *I*_obs_ values deposited in the PDB for this entry versus resolution. Thorn and coworkers used visual inspection of structure factor versus resolution patterns as in Fig. 1 ▸(*b*) to assess whether ice contamination was present in PDB-deposited structure factors for 156 IRRMC data sets. They validated these conclusions by examining the raw diffraction frames from the IRRMC as in Fig. 1 ▸(*a*) (inset). They then took structure-factor data from 200 randomly chosen PDB entries, used the same visual scoring (based on plots as in Fig. 1 ▸ *b*) as for the IRRMC data to determine whether they displayed ice contamination, and developed an algorithm for detecting ice contamination in deposited structure factors based on this data set. Thorn and coworkers then benchmarked their algorithm with a second set of 200 randomly chosen PDB entries.

Thorn and coworkers’ ice-detection algorithm, *AUSPEX*, looks for changes in the measured background-subtracted protein crystal Bragg reflection intensities within bins of fixed width in inverse resolution. The Ice Finder Score (IFS) is calculated for each inverse resolution bin as
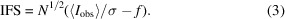
Here, *N*, 〈*I*_obs_〉 and σ are the number, mean and standard deviation of the measured Bragg intensities in a given inverse resolution bin. *f* is the expected normalized mean of the intensities (*i.e.* the mean divided by the standard deviation), estimated by the general trend of intensity values in bins away from the ice rings. To establish *f*, the mean and standard deviation of the intensities in coarse inverse-resolution bins of size 0.01 Å^−1^ were calculated. These were then linearly interpolated through regions containing ice rings and to reduce the bin size to 0.0025 Å^−1^, as shown by the green line in the example in Fig. 1 ▸(*b*). These interpolated values were used to calculate a normalized mean for each 0.0025 Å^−1^ bin, which was then smoothed with a Gaussian filter with a standard deviation of 0.01 Å^−1^ to give a final estimate of *f*. We also used a final bin size of 0.0025 Å^−1^, as this reduced fluctuations while still being smaller than the ∼0.005 Å^−1^ width of the regions biased by ice. Fig. 1 ▸(*c*) shows the IFS calculated for PDB entry 4h3w.

#### 2.6.3. Depletion Score   

We define a second metric, the Depletion Score (DS), which detects ice by looking for the depletion of low-intensity Bragg peak values at the ice-ring resolutions. It tracks the fraction of Bragg peaks in an inverse-resolution bin with intensities smaller than the expected mean intensity.

The Depletion Score was calculated using the same binning scheme as used for the IFS. The expected fraction of Bragg reflections with intensities smaller than the mean, 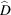, was estimated by calculating the fraction of Bragg reflections with intensities smaller than the mean in coarse inverse-resolution bins of 0.01 Å^−1^ and linearly interpolating this through regions containing ice rings and to increase the sampling to 0.0025 Å^−1^. The fraction of Bragg reflections smaller than the mean, *D*, was then recalculated in finer inverse-resolution bins of size 0.0025 Å^−1^ and subtracted from the expected fraction to estimate the Depletion Score,
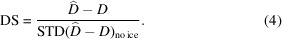
The chosen normalization is by the standard deviation of the difference calculated in regions away from the ice rings. While a fraction or multiple of the mean could be used, the algorithm is relatively insensitive to this factor. Fig. 1 ▸(*c*) shows the DS calculated for PDB entry 4h3w.

#### 2.6.4. Observation Score   

We define a third metric, the Observation Score (OS), which looks for a reduction in the number of observations within a resolution bin. This could occur if the experimenter excludes resolution regions overlapping the ice rings or if the integration and scaling algorithms throw out peaks as outliers. This metric is formed by counting the number of Bragg reflections *N* in inverse resolution bins of size 0.0025 Å^−1^ and comparing with an expected number of Bragg reflections, 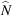,
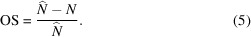
The expected number of Bragg reflections is generated from *N* by removing the regions overlapping ice rings, linearly interpolating through these regions and smoothing with a Gaussian filter with a standard deviation of 0.01 Å^−1^. Fig. 1 ▸(*c*) shows the Observation Score calculated for PDB entry 4h3w.

#### 2.6.5. A combined metric: *p*_ice_   

Our IFS and DS are continuous functions spanning the resolution range of the data set. These can be condensed into a single *p*-value for a data set. Pure hexagonal ice has 11 diffraction rings between 4 and 1.5 Å resolution (Table 1 ▸). Cubic ice has three diffraction rings, the locations of which match those of the (002), (110) and (112) hexagonal ice rings (Table 1 ▸, dark shading). Only rings at the locations of cubic ice are not broadened by stacking disorder, and these three rings typically had the largest pixel counts. We chose to focus only on these three ice-ring locations that are common to all forms of ice. Interpolations were made through larger resolution ranges about each location, while ice biasing was searched for in narrower ranges. Fig. 1 ▸ shows the interpolation regions with shading and the search regions within these using regularly spaced white horizontal lines. The resolutions of these regions are given in Supplementary Table S1. Fortes *et al.* (2004 ▸) reports a reference .cif file for hexagonal ice. As described in Supplementary Section S5.1, our focus on only three ice rings improved interpolations and had no detrimental effect on ice detection.

At each of these three ice-ring locations we calculate an Ice Contamination Score (ICS) as a weighted average of the IFS and DS,
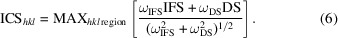
The weighting factors ω_IFS_ and ω_DS_ were chosen using Glass’s estimator of effect size (Glass, 1976 ▸). This was calculated as the difference of the means of the subsets of entries with and without ice diffraction normalized by the standard deviation,
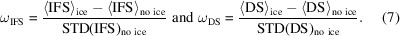
The maximum over a resolution range including the ice-ring location in (6) must be taken to account for small variations in ice-ring diffraction resolution and the resulting maximum of the structure-factor biasing. The maximum operation results in a random variable approximately following the generalized extreme value distribution (GEV; Jenkinson, 1955 ▸).

Depending on the resolution of the data set, we have can have one, two or three ICSs. These are combined to form a weighted average 〈ICS〉 with weights ω_*hkl*_. Protein crystals have much larger *B* factors than ice crystals. As resolution increases, the protein diffraction intensity decreases relative to the ice diffraction intensity, so higher resolution ice rings tend to have a more significant impact on the integrated intensities and ice biasing is easier to detect at higher resolutions. The weighting of the ICSs is based on the relative scale of ice to protein diffraction intensities to reflect the relative ice-detection capacity at each ice-ring location,
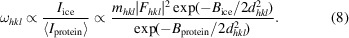
Here, *m_hkl_*, *F_hkl_* and *d_hkl_* are the multiplicity, structure factor and resolution for each of the three ice rings. *B*_ice_ and *B*_protein_ are the Wilson *B* factors of the ice and protein diffraction, respectively. *B*_ice_ is set to 1.5 Å^2^, the value used in our modelling of ice diffraction. *B*_protein_ is set to 35 Å^2^ based on the average *B* factor determined from our ground-truth data set. The weighting parameters are summarized in Supplementary Table S2.

A null distribution for 〈ICS〉 was formulated using the data sets flagged as not containing ice in the 1000-entry training set. The 〈ICS〉 was calculated for each entry and a GEV distribution was fitted to a histogram of the values, as shown in Supplementary Fig. S5. The *p*-value associated with an 〈ICS〉 is the value of the null cumulative distribution at this point: *p*_ice_ = CDF(〈ICS〉). The threshold *p*-value used to reject the null hypothesis was set to reduce the false-discovery rate (the fraction of data sets flagged for ice that were false positives) in the training set to less than 5% (Benjamini & Hochberg, 1995 ▸).

#### 2.6.6. Missing observations   

In the scenario where ice is present in the diffraction images and is accounted for by excluding integrated intensities in the region near the ice-ring resolutions, the deposited integrated intensities are no longer biased and the presence of ice diffraction is not reflected by *p*_ice_. A second *p*-value, *p*_obs_, is formulated to test the null hypothesis that observations have not been removed at the ice-ring locations. When structure factors are intentionally excluded for ice biasing, the exclusion tends to be made uniformly over a large resolution range. To reflect this, in each of the three ice-ring interpolation regions the mean of the observation score OS is taken and the mean maximum observation score is calculated as 〈OS〉. A null distribution is created by fitting a GEV distribution to these mean values taken from the entries without ice in the training data set. The *p*-value is calculated from the cumulative distribution function. The threshold *p*-value to reject the null hypothesis was set to reduce the false-discovery rate in the training set to less than 0.5%.

#### 2.6.7. Analysing PDB entries for the presence and type of ice   

This algorithm was applied to detect the presence of ice and its correlation with protein crystal properties such as solvent content and solvent-cavity size. To explore the correlation of ice with solvent-cavity size, the set of 16 953 PDB entries analysed in Moreau *et al.* (2019 ▸) was used. To explore correlations with unit-cell size, solvent content and year of data collection (quantities that are computationally much less complex to determine than solvent-cavity size), a larger set of 99 929 PDB entries was used. The lowest resolution ice ring used for detection is at 3.661 Å; ice-detection metrics become noisy and give false positives when there are too few Bragg reflections, and no ice is expected above and near the bulk freezing temperature of water. This larger set was thus pared of entries with resolution worse than 3.661 Å, fewer than 5000 Bragg reflections or unreported data-collection temperatures, leaving 94 752 data sets. This data set was further separated into two data sets: one with reported data-collection temperatures below 240 K and the other with reported data-collection temperatures above 240 K, with 89 827 and 4925 entries, respectively. Using these same exclusions, the 16 953-entry data set was reduced to 15 811 entries. The ‘year’ of each entry was taken as the reported year of data collection; if this was unavailable, the year of deposition was used instead.

To distinguish cubic/stacking-disordered ice from hexagonal ice, PDB entries that were flagged for ice were analysed a second time. Scoring was now performed at three hexagonal ice-peak locations not common to cubic ice, the (101), (102) and (103) peaks, using parameters for the weighting function ω_*hkl*_ listed in Supplementary Table S2. These locations were chosen because the ice rings there are strongly suppressed in typical stacking-disordered ice. Using three locations as in the initial analysis gave comparably reliable background interpolations. Of the five other hexagonal ice-ring locations at resolutions worse than 1.5 Å, the (100) ring is broadened by stacking disorder but is still distinguishable unless the cubic stacking fraction is very large, and so would also be suitable. Biasing effects of the (200) and (201) rings would be difficult to separate from that of the unbroadened (112) ring due to their close proximity and relatively weak intensity. The (202) and (203) hexagonal rings are at 1.72 and 1.52 Å, which are above the high-resolution cutoff for many PDB entries.

If ice was detected at any one of the (101), (102) or (103) ice-ring locations, the ice was classified as hexagonal; if no ice was detected at all of these additional locations, the ice was classified as stacking-disordered. Note that these classifications are not precise, as stacking-disordered ice with a large (>75%) hexagonal fraction will show peaks at all hexagonal locations (Malkin *et al.*, 2015 ▸).

## 3. Results   

### 3.1. Types of ice diffraction from bulk cryoprotectant solutions and from protein crystals   

**Figure 2:**
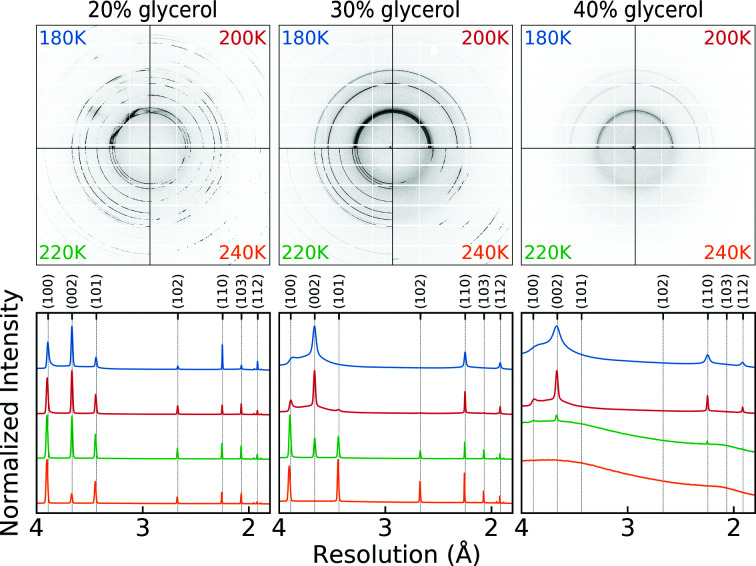
Ice diffraction from glycerol–water mixtures at different concentrations and temperatures. The top row shows 2D diffraction patterns, all plotted with the same pixel count to greyscale calibration. The bottom row shows 1D diffraction patterns obtained from the 2D patterns by azimuthal averaging. The intensity scales of the 1D patterns are individually normalized. Similar trends are observed with other common cryoprotectants, including 2-methyl-2,4-pentanediol (MPD), sucrose and polypropylene glycol.

Ice diffraction in protein crystallography can arise from ice in the internal crystal solvent, from ice formed in residual cryoprotectant-containing solvent on the crystal surface and from frost. Fig. 2 ▸ shows 2D diffraction images (top row) and 1D azimuthally averaged diffraction patterns (bottom row) of ice formed in glycerol solutions contained in 250 µm diameter, thin-wall polyester tubing and cooled in a nitrogen-gas cryostream to temperatures of between 180 and 240 K. For solutions with 20%(*v*/*v*) glycerol or lower, the diffraction obtained when cooling to all temperatures indicates that the ice is largely hexagonal and the diffraction patterns are azimuthally lumpy, indicating a relatively large grain size and a relatively small number of grains within the X-ray-illuminated volume. For 30% glycerol solutions, ice diffraction at 240 K is again azimuthally lumpy and largely hexagonal. However, the ice diffraction obtained by cooling to 180 K is isotropic, indicating a small grain size, and its resolution dependence is consistent with stacking-disordered ice with a substantial cubic fraction. For 40% glycerol solutions, no ice forms at 240 K. At 180 K, ice diffraction is isotropic and consistent with stacking-disordered ice with a largely cubic character. The integrated intensity of the ice diffraction is much smaller than for 30% and 20% glycerol solutions, indicating that a significant fraction of the illuminated sample volume has vitrified.

**Figure 3:**
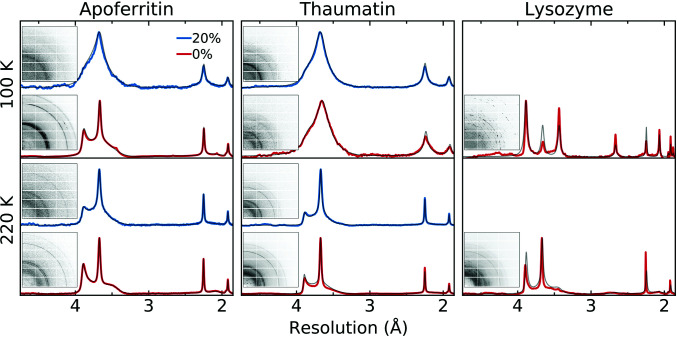
Example protein Bragg peak-subtracted, azimuthally averaged diffraction patterns from apoferritin, thaumatin and lysozyme crystals for which ice formed in internal crystal solvent. Crystals were either used as-grown (0%) or were soaked in solutions containing 20% glycerol and cooled to 220 K (top) or 100 K (bottom). The insets show the corresponding 2D diffraction patterns. Largely hexagonal ice diffraction from as-grown lysozyme crystals (which have narrow solvent cavities and little bulk-like solvent) cooled to 100 K arises from ice within solvent pockets that form inside the crystal to accommodate solvent squeezed out by the contracting protein lattice.

Fig. 3 ▸ shows examples of diffraction from internal ice in crystals of apoferritin, thaumatin and lysozyme soaked in solutions containing either 0% or 20%(*v*/*v*) glycerol, carefully blotted in a humid gas stream to remove all external solvent, transferred to NVH oil to prevent dehydration and cooled to temperatures of 220 and 100 K. As discussed in Moreau *et al.* (2019 ▸), in all cases the observed ice diffraction is neither cubic nor hexagonal nor a simple mixture of the two, but exhibits selective and anisotropic peak broadening that is characteristic of stacking-disordered ice. As the glycerol concentration increases, the cubic stacking fraction increases. The cubic fraction is largest on cooling to 100 K, which gives the largest average cooling rate between the freezing and glass-transition temperatures of the internal solvent.

**Figure 4:**
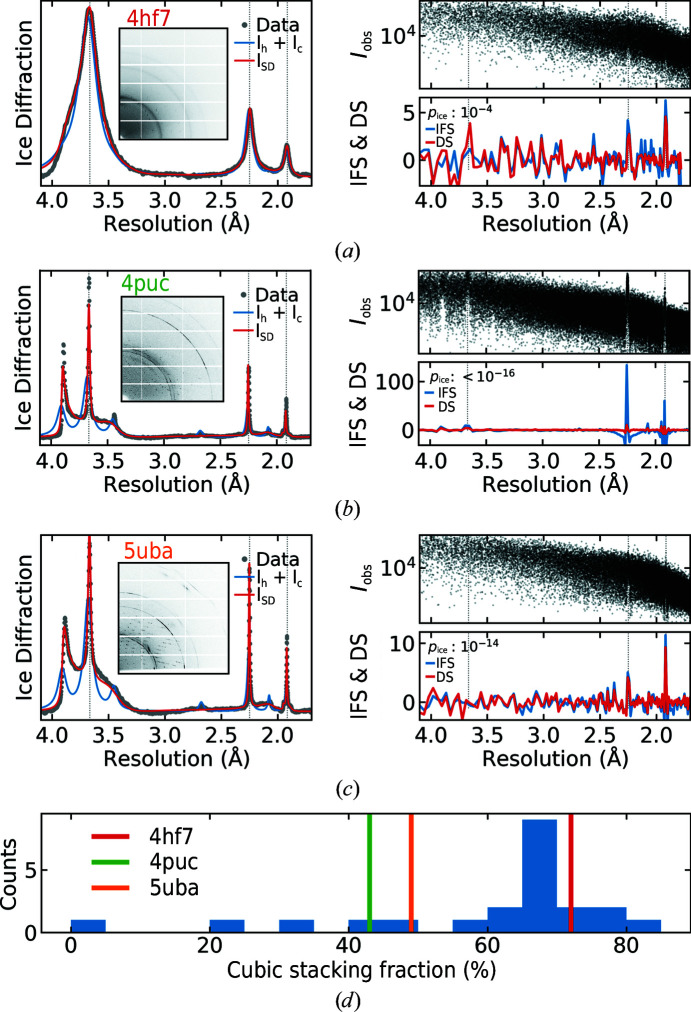
(*a*, *b*, *c*) Left column: ice diffraction from three data sets taken from the IRRMC. The top example shows ice diffraction from nearly cubic ice (PDB entry 4hf7), the middle example shows ice that is mostly stacking-disordered with some additional hexagonal component (PDB entry 4puc) and the bottom example shows stacking-disordered ice (PDB entry 5uba). Right column: structure-factor distribution and calculated Ice Formation and Depletion Scores versus resolution for each PDB entry in the left column. (*d*) Histogram of the cubic stacking fraction calculated from 22 IRRMC data sets along with the cubic stacking fraction of the three examples (also from the IRRMC).

The left column in Figs. 4 ▸(*a*)–4 ▸(*c*) shows three examples of 2D and azimuthally averaged 1D ice diffraction patterns taken from data sets in the IRRMC, along with the best fits to a mixture of hexagonal and cubic ice and to a stacking-disordered ice model. In the example shown in Fig. 4 ▸(*a*) (PDB entry 4hf7), the ice diffraction pattern nearly matches that expected for cubic ice I_c_; in the example shown in Fig. 4 ▸(*b*) (PDB entry 4puc), the ice is mostly stacking-disordered I_sd_ with an additional hexagonal component I_h_; and in the example shown in Fig. 4 ▸(*c*) (PDB entry 5uba), the ice is almost entirely stacking-disordered. For each example, the right-hand column shows a scatter plot of the *I*_obs_ values versus resolution, and the calculated Ice Finder Score (IFS) and Depletion Score (DS) as defined in Section 2.6. Fig. 4 ▸(*d*) shows a histogram of the cubic stacking fraction calculated from 22 IRRMC data sets along with the cubic stacking fraction of the three examples in Figs. 4 ▸(*a*)–4 ▸(*c*). The highly cubic diffraction from 4hf7 is more typical of ice in the deposited IRRMC data sets.

The three examples in Figs. 4 ▸(*a*)–4 ▸(*c*) were chosen because they showed large structure-factor biasing due to ice. As a result, the *p*_ice_ scores are far below the 0.05 threshold traditionally used in hypothesis testing. The trends in IFS and DS are representative of the biasing observed for each type of ice. The nearly cubic ice in the top example has the smallest peak values of IFS and DS. The broad diffraction peaks typical of cubic-like ice cause less variation in background intensity between the Bragg peaks and neighbouring regions and so have a smaller effect on the integrated intensities. Hexagonal-like ice generates ice rings that are much narrower and, for the same integrated intensity, much taller, causing large biasing of integrated intensities. The stacking-disordered ice in the bottom example gives detectable ice biasing at the higher resolution (110) and (112) ice-ring positions but not at the (002) position. Ice diffraction typically has *B* factors an order of magnitude smaller than those of protein diffraction. The diffraction strength of the protein decreases much more rapidly with increasing resolution, and so the biasing of integrated intensities at lower resolutions, where protein diffraction is relatively stronger, is less.

### 3.2. Estimates of ice-crystallite sizes   

**Figure 5:**
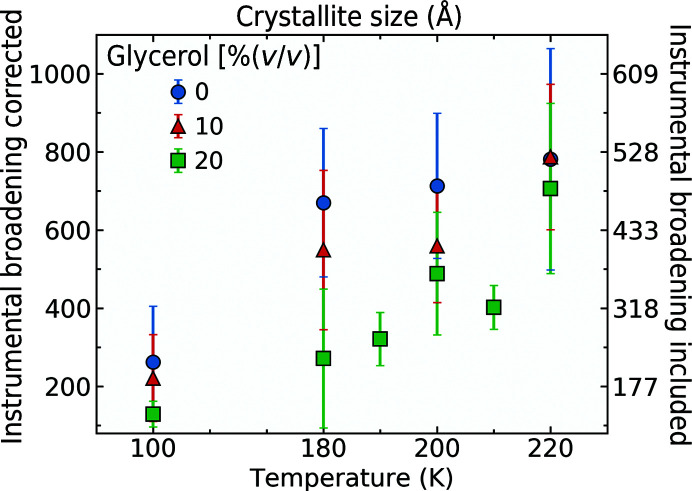
Ice-crystallite sizes for internal ice in apoferritin crystals that were soaked in different concentrations of glycerol and cooled to temperatures between 100 and 220 K. The left axis shows the crystallite size with correction for instrumental broadening. The right axis shows the crystallite sizes corresponding to the left-axis tick locations if instrumental broadening is not accounted for; these values give a lower bound on the crystallite sizes.

Fig. 5 ▸ shows estimated crystallite sizes for ice formed from solvent internal to apoferritin crystals for a range of temperatures and glycerol concentrations. The left axis displays the approximate crystallite sizes determined from ice-ring widths corrected for instrumental broadening. The tick locations on the left and right axes are the same, and the tick labels on the right axis are the crystallite sizes corresponding to the tick labels on the left axis, calculated using the full experimental peak width, uncorrected for instrumental broadening. These latter values serve as a lower bound for the crystallite size. These crystallite size estimates have considerable uncertainty because of the simplicity of the model used to determine them. However, they are ∼3–10 times larger than the solvent cavities within apoferritin (68 Å) and 3–4 orders of magnitude smaller than the protein crystals themselves (or 9–12 orders of magnitude smaller in volume). This suggests that when ice forms within the crystal, the crystal lattice must be disrupted to make space for the ice crystals as they grow during cooling. This is consistent with the large increases in mosaicity and the dramatic loss of ordered diffraction from the protein lattice when significant internal ice diffraction develops.

### 3.3. Ice diffraction-spot analysis   

**Figure 6:**
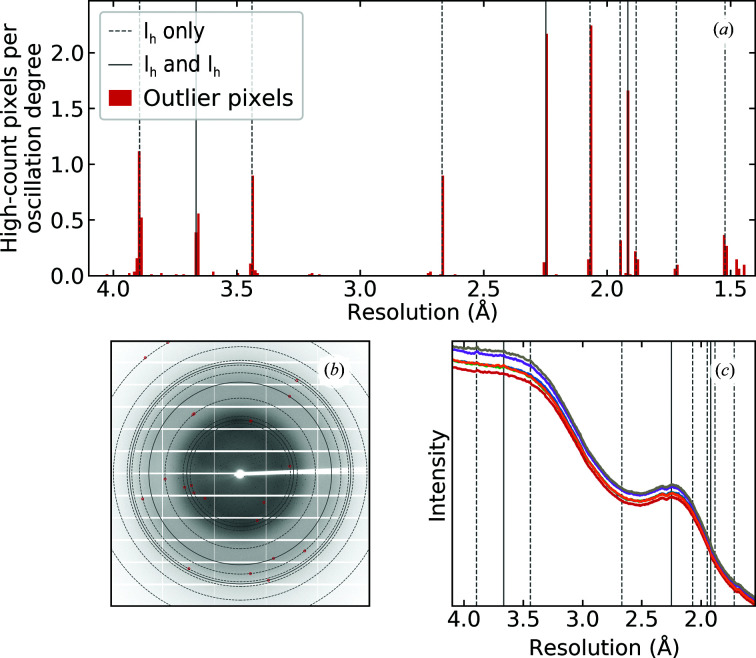
(*a*) Number of high-count pixels per oscillation degree versus resolution for PDB entry 4exr. Dashed vertical lines show resolutions where ice rings exclusive to hexagonal ice and to stacking-disordered ice with a large hexagonal fraction occur, and solid lines show resolutions common to hexagonal, cubic and stacking-disordered ice. The 2D diffraction frames taken from the IRRMC show no evidence of ice rings. (*b*) A 2D diffraction image (0.5° oscillation) for PDB entry 4exr taken from the IRRMC, with red circles drawn at the locations of outlier pixels and solid guidelines drawn at ice-ring resolutions. (*c*) 1D azimuthally averaged diffraction intensity versus resolution, calculated using every 30th 2D frame in the data set, confirming the absence of ice rings.

Histograms of outlier pixels versus resolution covering the resolution range 10–1.4 Å were generated for 60 data sets from the IRRMC archive, including 26 data sets with visible ice rings and an additional 34 data sets without visible ice rings. Details and results for these data sets are given in Supplementary Table S3. Fig. 6 ▸(*a*) shows an example histogram for PDB entry 4exr, Fig. 6 ▸(*b*) shows a single diffraction frame from this entry showing outlier pixels (zingers, ice diffraction) and Fig. 6 ▸(*c*) shows azimuthally integrated backgrounds for several frames spanning the full angular range of the data set. Even though the diffraction frames and integrated backgrounds show no evidence of ice, roughly 82% of the outlier pixels are located within 0.01 Å of one of 11 hexagonal ice-ring resolutions, which comprises ∼10% of the detector area in the 10–1.4 Å range where outlier pixels were searched for.

Of the 34 data sets from the IRRMC archive that showed no visible ice rings in the azimuthally averaged backgrounds, 22 showed significant numbers of diffraction spots at hexagonal ice-ring locations. Diffraction spots were observed at all hexagonal ice-ring resolutions, not just those that are common to hexagonal and cubic ice, indicating that the ice responsible was largely hexagonal. For these 22 data sets, the total number of diffraction spots at ice-ring locations per oscillation degree ranged between 1 and 53, with an average of 9 ± 12, and the fraction of all outlier pixels observed at hexagonal ice locations ranged from 21% to 84%. For the remaining 12 IRRMC data sets, which were both ice-ring-free and ice diffraction-spot-free, the average number of outlier pixels per oscillation degree was 3 ± 4. These could be attributed to large, but statistically plausible, background pixels and were more prevalent in data sets with weaker backgrounds.

Another 20 data sets taken from the IRRMC archive showed stacking-disordered or cubic-like ice rings. Of these, 12 showed an elevated number of ice spots at ice-ring resolutions completely suppressed by the stacking disorder and had no visible diffraction peaks at these resolutions in the 2D or 1D diffraction patterns.

Thus, for all 35 data sets showing substantial numbers of isolated ice diffraction spots, the observed spots are consistent only with hexagonal ice. Furthermore, the hexagonal ice spots are typically single pixels with very high count rates (for example much larger than the count rates observed in ice rings when they are present), indicating that they are generated by relatively large ice crystals: to produce a (112) hexagonal ice peak at its resolution of 1.916 Å that is only a single pixel wide, the ice crystal needs to be a minimum of 4000 Å in linear dimension (calculation in Supplementary Section S4). This crystal size is at least one to two orders of magnitude larger (in linear dimension, and three to six orders of magnitude larger in volume) than the size of the crystallites formed in cryoprotected solvent within protein crystals that generate the ice diffraction rings. These large hexagonal ice crystals are likely to be frost crystallized from moist ambient air that accumulated in the liquid nitrogen used to cool the crystals, that formed on the crystal during post-cooling handling or during data collection at the beamline, or that formed in the solvent surrounding the crystals when the cooling rates were small enough to allow substantial ice growth before vitrification.

Analysis of outlying pixels was also performed using the X-ray diffraction images we collected from apoferritin, thaumatin and lysozyme crystals as described in Section 2.2. These were prepared with all external solvent removed and cooled *in situ* in the nitrogen-gas cryostream. No evidence of hexagonal ice diffraction spots was observed for 221 data sets from apoferritin, lysozyme or thaumatin, including 82 data sets that showed ice rings characteristic of stacking-disordered ice formed in internal solvent.

### 3.4. Comparison of metrics for detecting ice in PDB-deposited structure factors   

Table 2 ▸ compares the ice-detection performance of our *p*_ice_, based on our Ice Contamination Score (ICS), with that of the *AUSPEX* Ice Finder Score (IFS) for the randomly selected PDB data sets used by Thorn and coworkers to benchmark their algorithm. Visual inspection of the *I*_obs_ values (Fig. 1 ▸ *b*), looking for a depletion of low-intensity Bragg peaks or a spike in high-intensity peaks at ice-ring resolutions, was used to identify data sets containing ice. Our visual classification differed from that of Thorn and coworkers for eight entries, and two entries were removed from consideration because their resolutions were less than 3.661 Å. We thus used our visual classification and 198 of the 200 data sets examined by Thorn and coworkers, as listed in Supplementary Table S4; plots as in Fig. 1 ▸(*b*) for each entry can be found in the supporting information.

For the automated classification, we used *p*-value thresholds of 0.006 and 0.00005 for *p*_ice_ and *p_n_*, respectively, to flag a data set for ice biasing. These were set to reduce the false-discovery rate in the training set to 5% and 0.5% for *p*_ice_ and *p_n_*, respectively. False positives are data sets that do not have ice by visual inspection but that are flagged as containing ice with the ice-detection algorithm; false negatives are similarly evaluated. Compared with the *AUSPEX* algorithm, our algorithm reduces the rate of false positives from 10.5% to 4.9% and the rate of false negatives from 44.4% to 16.7% for the data set used by Thorn and coworkers. For the 200-entry test data set taken from ground-truth data, which did not include any of the data sets used by Thorn and coworkers we observed false-positive and false-negative rates of 2.1% and 55%, respectively. When analyzed with *AUSPEX*, the false-positive and false-negative rates were 5.6% and 63%, respectively. Using the ground-truth data set classified solely on structure-factor biasing, the false-negative rates decreased to 18.4% and 34.2% for *p*_ice_ and *AUSPEX*, respectively. Supplementary Table S5 reports a confusion matrix for our analysis of this test set. Supplementary Section S5.3 and Supplementary Table S5 show that our *p*_ice_, which is obtained from a combination of IFS and DS, outperforms DS as well as IFS.

### 3.5. Prevalence and types of ice in the PDB   

**Figure 7:**
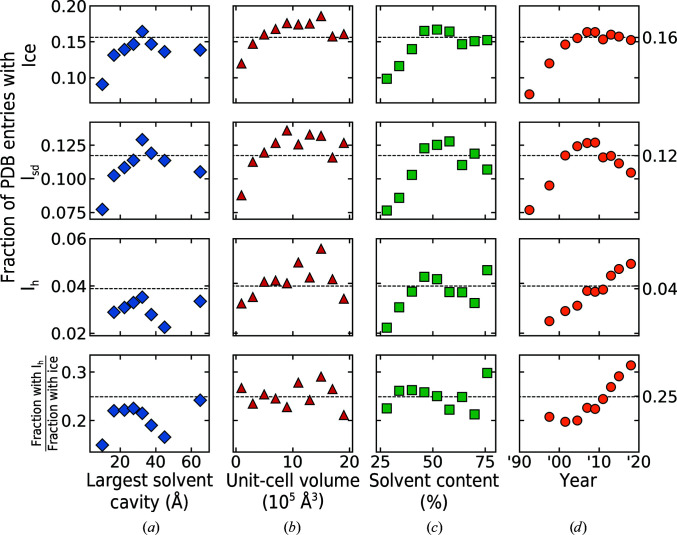
Fraction of PDB entries showing ice (first row), stacking-disordered ice I_sd_ only (second row) and hexagonal ice I_h_ (third row), and the fraction of all entries with ice contamination that show hexagonal ice (fourth row), as determined using *p*_ice_ and the methods described in Section 2.6. Column (*a*) shows the variation with largest solvent-cavity size, using the 15 811 PDB entries analysed in Moreau *et al.* (2019 ▸). Columns (*b*), (*c*) and (*d*) show the variations with unit-cell volume, solvent content and the year the data were collected for a random set of 89 827 PDB entries. Dashed horizontal lines represent averages within an entire data set.

Fig. 7 ▸ shows the distributions of ice and the relative prevalence of ‘hexagonal’ and ‘stacking-disordered’ ice (defined and determined as described in Section 2.6) versus solvent-cavity size (Fig. 7 ▸ *a*), unit-cell volume (Fig. 7 ▸ *b*), solvent content (Fig. 7 ▸ *c*) and deposition year (Fig. 7 ▸ *d*) for 15 473 (Fig. 7 ▸ *a*) and 88 558 (Figs. 7 ▸ *b*–7 ▸ *d*) randomly selected PDB entries for which data were collected at cryogenic temperature (specified as below 240 K in the PDB header, and typically ∼100 K) and extended to at least 3.661 Å resolution. The presence of ice was determined using our *p*_ice_ and the character of the ice was determined as described in Section 2.6.5. The horizontal dashed lines represent an average over the 89 827 PDB entries used in Figs. 7 ▸(*b*)–7 ▸(*d*). Roughly 16% of these entries display ice contamination, consistent with the 19% estimated by Thorn and coworkers. Of this subset, roughly 25% showed ice that we labelled as hexagonal and so were likely to have frost as a major component, but the ice could also have been ice formed in surrounding solvent if the cryoprotectant concentrations and cooling rates were modest. The remaining 75% showed ‘stacking-disordered’ ice that arose solely from residual cryoprotected solvent on the crystal surface or from internal crystal solvent. The prevalence of ice increases with increasing solvent-channel size, unit-cell volume and solvent content, suggesting that ice formed in the internal solvent is a major contributor. Since 2000, the fraction of annual deposits with ice has been relatively constant. However, the prevalence of hexagonal ice among samples that exhibit ice has steadily increased with time.

The 89 827 PDB entries with data-collection temperatures, as indicated by their PDB header files, below 240 K that were analysed in generating Fig. 7 ▸ were a subset of the larger randomly selected set of 94 752 PDB entries. A second subset of 4925 PDB entries with listed data-collection temperatures above 240 K was excluded from the analysis. However, 219 of these ‘high-temperature’ data sets were flagged to be biased by ice diffraction. Journal publications associated with a random selection of 30 of these PDB entries were examined. Of these publications, 15 indicated data collection was at cryogenic temperature, eight did not state the data-collection temperature, three stated that data were collected at room temperature and four entries did not have an associated publication. In many cases, the depositor is likely to have used the crystallization temperature instead of the data-collection temperature. Given that 4.4% of these ‘room-temperature’ entries exhibited ice, and that 16% of PDB entries with listed data-collection temperatures below 240 K show ice, roughly 28% of the 4925 PDB entries with listed experimental temperatures above 240 K were probably collected at 100 K.

## 4. Discussion and conclusions   

### 4.1. Types and origins of ice diffraction in protein cryocrystallography   

In an ideal protein cryocrystallography experiment, X-ray diffraction from all sources apart from the protein crystal is minimized. This diffraction, typically diffuse scatter from liquid water, oils or polymers associated with the crystallo­graphy mounts, varies slowly with angle and is largely isotropic, facilitating the accurate subtraction of this background from the protein lattice Bragg peaks.

In fact, intense, highly structured background diffraction generated by ice is frequently observed. This ice arises from three different sources: solvent internal to the protein crystal, solvent external to the protein crystal and accumulated frost, each of which produces characteristic diffraction patterns.

Firstly, ice may form within the solvent inside protein crystals. Ice-crystal growth causes disruption of the protein lattice, degrading the protein diffraction mosaicity and resolution. As we have -previously discussed (Moreau *et al.*, 2019 ▸), ice formation is strongly suppressed by its nanoconfinement within the protein network and is far less likely to form there than in surface solvent, even in crystals with large solvent cavities. Long-range propagation of crystalline ice order within the solvent cavities is inhibited by an intact protein lattice. As a result, ice-grain sizes tend to be small, the number of grains large and the ice-ring diffraction homogeneous and isotropic. The kinetics of ice nucleation and growth in deeply supercooled solutions (Malkin *et al.*, 2015 ▸) and within the nanoconfined environment (González Solveyra *et al.*, 2011 ▸) favours the formation of stacking-disordered ice (Lupi *et al.*, 2017 ▸). When the internal solvent has low concentrations of cryoprotectants and/or other solutes, the cubic stacking fraction is near 50%. As cryoprotectants are added and/or cooling rates are increased, the cubic stacking fraction increases (Moreau *et al.*, 2019 ▸) and the grain size decreases (Fig. 5 ▸).

Secondly, ice may form in residual solvent present on the crystal surface. This solvent typically contains substantial concentrations of cryoprotectants, including salts used for crystallization. Cryoprotectants decrease the freezing temperature and increase the glass-transition temperature. They complicate ice nucleation and growth because they must be excluded from the growing ice crystal. As ice crystals grow, cryoprotectants become concentrated in the remaining uncrystallized solvent, further decreasing its freezing temperature, increasing its glass-transition temperature and inhibiting ice-crystal growth. However, when ice forms in residual surface solvent, its growth is unhindered by the protein crystal, allowing ice to grow to a modest grain size, producing azimuthally lumpy or streaky diffraction rings. For low cryoprotectant concentrations and low cooling rates, this surface ice will have a primarily hexagonal character with a larger grain size, and the azimuthal lumpiness of the diffraction at the ice-ring positions will be pronounced. For higher cryoprotectant concentrations and/or faster cooling rates, the ice becomes stacking-disordered. The fraction of cubic planes in the stacking-disordered structure increases, the ice-grain size decreases and the ice rings become more homogeneous and isotropic with increasing cooling rate and increasing cryoprotectant concentration. These trends are evident in Fig. 2 ▸.

Thirdly, frost may be present on the crystal or on the sample-holder/loop surface. Frost may condense from moist air during post-cooling handling and during data collection in a misaligned or otherwise malfunctioning cold gas stream. Frost may accumulate in the liquid nitrogen used for cooling and storage and adhere to the crystal and loop surface. Since it forms from pure water and under modest cooling rates, frost is always pure hexagonal ice. Ice spots are by far the most common source of outlier pixels in cryogenic temperature diffraction frames, which are generated when only a small number of ice crystals are present in the X-ray beam, too few to generate continuous or quasi-continuous ice diffraction rings.

The presence of one of these forms of ice does not require the presence of another form. Frost may be present in the absence of any other form of ice because it can accumulate on the crystal while stored in liquid nitrogen at temperatures far below the glass-transition temperature of the solvent internal or external to the crystal. Because the protein crystal provides a physical obstacle to the propagation of ice from its exterior and the nanoconfinement of solvent within the crystal raises the glass-transition temperature and lowers the freezing temperature, ice can form in the external solvent without penetrating the protein crystal, particularly if the crystal lattice is not disrupted by defects.

### 4.2. Trends in ice formation versus cryoprotectant concentration and final temperature   

As the cryoprotectant concentration increases, the equilibrium freezing temperature *T*_f_ decreases [from ∼266 K at 20%(*v*/*v*) to ∼255 K at 40%(*v*/*v*)] (Lane, 1925 ▸). Assuming a roughly exponential approach to the final (cryostream) temperature *T*_final_, the average cooling rate between *T*_f_ and, for example, 1.05*T*_final_ decreases with increasing cryoprotectant concentration. This effect is most pronounced in the 240 K data, giving slower ice growth that favours hexagonal stacking. As the final temperature is lowered, the average cooling rate from *T*_f_ to, for example, 1.05*T*_final_ increases, nucleation occurs at deeper supercooling where growth rates are larger, and stacking-disordered ice is generated. The ice-growth rate decreases with increasing cryoprotectant concentration. Growing ice rejects cryoprotectant, which becomes concentrated in the remaining uncrystallized solution, raising its glass-transition temperature and decreasing the cooling rate required for it to vitrify. As a result, as the initial cryoprotectant concentration increases, a decreasing fraction of the sample will crystallize before the remaining liquid vitrifies.

The crystallite size increases as the temperature to which a crystal is cooled increases. This is consistent with the average cooling rate from the solvent freezing temperature to near the final temperature being smaller and the time available for ice to nucleate and grow being longer when the final temperature is higher. It is also consistent with smaller ice-nucleation rates and larger ice-growth rates at temperatures modestly below the freezing temperature, compared with at much lower temperatures.

As more cryoprotectant is added, the size of the crystallites decreases. Ice crystals contain no or very little cryoprotectant, and cryoprotectant molecules are excluded at the surface of the growing ice crystal. Cryoprotectant thus becomes concentrated in the remaining uncrystallized solution, lowering the freezing point of the solution, raising its glass-transition temperature and lowering the critical cooling rate required for it to vitrify. These reduce ice-growth rates, increase the fraction of solvent that vitrifies without ice formation and reduce the ice-crystallite size.

### 4.3. Detection and prevalence of ice in PDB depositions   

Both the Ice Finder Score (IFS) from the *AUSPEX* algorithm and our extension, *p*_ice_, allow the automated detection of ice biasing of experimental structure-factor amplitudes. Based on comparisons between these scores and visual ‘scoring’ of corresponding 2D diffraction frames, *p*_ice_ provides modestly lower rates of false positives and false negatives over the use of IFS alone when all quantities are calculated using our improved algorithms, largely due to the reductions in noise obtained by combining IFS and DS, and substantially lower rates of false positives and false negatives over the use of IFS as calculated using the original *AUSPEX* algorithm, due to algorithm improvements based on the analysis of large test sets that we have implemented. False negatives are typically less problematic than false positives, because structure-factor biasing in the case of false negatives was always small.

The utility of the two approaches can be scored using their *sensitivity* (the ratio of the number of true positives to the sum of the number of true positives and false negatives), which measures the ability to correctly identify entries with ice biasing, and their *specificity* (the ratio of the number of true negatives to the sum of the number of true negatives and false positives), which measures the ability to correctly identify entries that are ice-free (Altman & Bland, 1994 ▸). For a test set of 200 PDB entries, our *p*_ice_ algorithm improved the sensitivity and specificity relative to *AUSPEX* from 72% to 86% and from 92% to 95%, respectively.

The false-discovery rate of the *p*_ice_ algorithm, or the fraction of data sets flagged to contain ice that are false positives (Benjamini & Hochberg, 1995 ▸), was estimated to be 9.1% from our ground-truth data set. Extrapolating from these results, if our algorithm flags a data set as containing ice diffraction, there is a 9.1% chance that it is a false positive. While this may seem high, it is typical of *p*-values (Colquhoun, 2014 ▸). *p*-values are frequently misinterpreted and misused (Ioannidis, 2019 ▸; Nuzzo, 2014 ▸; Wasserstein & Lazar, 2016 ▸). Our *p*_ice_ metric is a quantitative measure of the compatibility between the measured 〈ICS〉 value and a distribution of 〈ICS〉 values taken from a data set of entries confirmed not to be biased by ice diffraction. A low *p*_ice_ does not definitively show that the data set has been biased by ice. *p*_ice_ is useful as a condensed and interpretable metric, but *p*-values are not designed to be used as standalone quantities (Nuzzo, 2014 ▸). A *p*-value or any other single metric flagging a set of structure factors for ice biasing should be compared with other information, such as images of diffraction frames, for confirmation.

For the broader PDB, both *AUSPEX* and our algorithm indicate similar overall levels of ice contamination, 19% and 16% of entries, respectively, a fraction that has remained roughly constant over the last 30 years. Our algorithm also gives information on the type of ice present, which is related to its origin. Of PDB entries with ice contamination, roughly 25% show hexagonal ice due to crystal/loop frosting and contamination, excess solvent surrounding the crystal and perhaps also due to crystals with mechanically damaged regions or growth defects (for example voids or inclusions) containing pools of solvent that are larger than the solvent cavities in ordered protein crystal regions. This fraction has increased by roughly 60% over the last 20 years. Diffraction from this hexagonal ice tends to be anisotropic and inhomogeneous, unlike diffraction from the stacking-disordered ice that forms in the solvent cavities of reasonably well ordered crystals. As a result, hexagonal ice diffraction tends to be much more difficult for advanced background-subtraction methods to account for and so has a larger impact on the integrated structure factors.

The increasing prevalence of hexagonal ice, even as beamline cryocooling hardware has evolved to largely eliminate frosting, suggests that the cryocooling protocols for an increasing fraction of structural targets have been inadequate. This could reflect a greater focus on challenging targets: crystals with large solvent contents, large solvent cavities, fragile lattices, inconvenient (needles, clusters) growth habits and/or for which suitable cryoprotectant conditions may be difficult to identify. Time-consuming cryoprotection protocols may have been relaxed to increase throughput, and a larger fraction of crystallographic data are now collected by those for whom crystallography is not a primary focus. The shift to remote data collection may also be a factor. While a dry shipper does an excellent job at keeping crystals cold in transit to a synchrotron facility (Owen *et al.*, 2004 ▸), it can accumulate frost if the lid is left open or if samples are frequently removed.

### 4.4. Minimizing ice in cryocrystallography   

We conclude by summarizing the factors that affect ice formation in cryocrystallography (Garman, 1999 ▸; Garman & Doublié, 2003 ▸; Pflugrath, 2015 ▸).

(i) *Cooling rates.* Cooling rates in current practice vary by at least three orders of magnitude and are most heavily dependent on the thermal mass of the sample (crystal plus surrounding liquid) and whether gas or liquid cryogens are used (Chinte *et al.*, 2005 ▸; Kriminski *et al.*, 2003 ▸; Teng & Moffat, 1998 ▸; Warkentin *et al.*, 2008 ▸; Walker *et al.*, 1998 ▸). The cooling rate also depends on the plunge speed, the choice of liquid cryogen (Teng & Moffat, 1998 ▸) and the extent of precooling by cold gas present above the liquid cryogen (Warkentin *et al.*, 2006 ▸).
(ii) *Cryoprotectant concentration.* In aqueous solutions, the minimum cooling rates required to obtain a sample with no detectable ice increase exponentially with decreasing cryoprotectant concentration (Warkentin *et al.*, 2013 ▸). Addition of cryoprotectants can damage protein crystals or interfere with ligand binding, placing an upper limit on the amount of cryoprotectant that can be added to growth solutions or soaked into a crystal. Larger cryoprotectant concentrations can be tolerated if crystals are quickly swiped through a cryoprotectant solution and cooled immediately before appreciable diffusion into the crystal occurs.
(iii) *The amount of solvent surrounding the crystal.* Ice formation in solvent internal to the crystals is strongly suppressed by nanoconfinement (Moreau *et al.*, 2019 ▸). For a given cooling rate, the external solvent requires much larger cryoprotectant concentrations to prevent ice formation. This external solvent can be wicked away (Pflugrath, 2015 ▸) or replaced with oils (Kwong & Liu, 1999 ▸; Riboldi-Tunnicliffe & Hilgenfeld, 1999 ▸; Panjikar & Tucker, 2002 ▸; Warkentin & Thorne, 2009 ▸).
(iv) *Crystal solvent content and solvent-cavity size.* Ice formation in internal solvent is strongly suppressed by nanoconfinement, and solvent within the first two hydration layers adjacent to the protein molecules generally does not crystallize. Ice is thus most likely to form within crystals with large solvent cavities and large fractions of bulk-like internal solvent (Moreau *et al.*, 2019 ▸).
(v) *Crystal perfection.* Growth defects such as dislocations, inclusions and vacancies, more general lattice-scale disorder caused by imperfect molecular packing, as well as defects/disorder created by osmotic shock during cryoprotectant soaks, inadvertent crystal dehydration and mechanical damage during handling, can all produce solvent pockets within the crystal that are much larger than the solvent cavities in ordered portions of the crystal that are identified by crystallography. Solvent in these relatively less confined regions has a higher freezing point (Findenegg *et al.*, 2008 ▸) and ice-nucleation rate (Li *et al.*, 2013 ▸). Ice may thus be orders of magnitude more likely to first nucleate in these larger solvent pockets, and the ice that forms will have a larger grain size and be more likely to generate anisotropic, lumpy diffraction than ice that forms within (initially) ordered solvent cavities.

## 5. Related literature   

The following reference is cited in the supporting information for this article: Fortes (2018 ▸).

## Supplementary Material

Supporting Information and Supplementary Figures and Tables. DOI: 10.1107/S2059798321001170/tz5104sup1.pdf

Excel workbook comparing the performance of our ice-detection methods with those of AUSPEX for ground-truth and test data sets. DOI: 10.1107/S2059798321001170/tz5104sup2.xlsx

Plots of deposited structure-factor intensities, IFS, DS and N versus resolution for the 200 PDB entries also used by Thorn and coworkers to benchmark their algorithm. DOI: 10.1107/S2059798321001170/tz5104sup3.zip

Scripts for P_Ice. The example generates some results from this paper. DOI: 10.1107/S2059798321001170/tz5104sup4.zip

## Figures and Tables

**Table 1:** Miller indices of hexagonal ice rings at resolutions numerically larger than 1.5 Å Ice rings at the resolutions indicated in the rows in bold are not broadened by stacking disorder. Cubic ice generates ice rings only at these three resolutions.

(*hkl*)	Resolution (Å)
(100)	3.895
**(002)**	**3.661**
(101)	3.438
(102)	2.667
**(110)**	**2.249**
(103)	2.068
(200)	1.947
**(112)**	**1.916**
(201)	1.882
(202)	1.719
(203)	1.522

**Table 2:** Our *p*_ice_-based ice-detection algorithm was benchmarked against the *AUSPEX* algorithm using the same 200 randomly selected PDB entries as used by Thorn *et al.* (2017 ▸) Ice biasing of the structure factors was observed by visual inspection of 37 entries. The percentage of entries visually observed to be ice-free but that were flagged as having ice is listed as the false-positive rate and the percentage of entries visually observed to have ice contamination but that were not flagged as having ice is listed as the false-negative rate.

	*p*_ice_	*AUSPEX*
False-positive rate (%)	4.9	10.4
False-negative rate (%)	16.7	40.5

## References

[bb1] Altman, D. G. & Bland, J. M. (1994). *Br. Med. J.* **308**, 1552. 10.1136/bmj.308.6943.1552 PMC2540489 8019315

[bb2] Ashiotis, G., Deschildre, A., Nawaz, Z., Wright, J. P., Karkoulis, D., Picca, F. E. & Kieffer, J. (2015). *J. Appl. Cryst.* **48**, 510–519. 10.1107/S1600576715004306 PMC4379438 25844080

[bb3] Baker, I. (2002). *Cryst. Growth Des.* **2**, 127–134.

[bb4] Benjamini, Y. & Hochberg, Y. (1995). *J. R. Stat. Soc. Ser. B*, **57**, 289–300.

[bb5] Blessing, R. H. (1997). *J. Appl. Cryst.* **30**, 421–426.

[bb6] Chinte, U., Shah, B., DeWitt, K., Kirschbaum, K., Pinkerton, A. A. & Schall, C. (2005). *J. Appl. Cryst.* **38**, 412–419.

[bb7] Colquhoun, D. (2014). *R. Soc. Open Sci.* **1**, 140216. 10.1098/rsos.140216 PMC4448847 26064558

[bb8] Findenegg, G. H., Jähnert, S., Akcakayiran, D. & Schreiber, A. (2008). *ChemPhysChem*, **9**, 2651–2659. 10.1002/cphc.200800616 19035394

[bb9] Fortes, A. D. (2018). *Acta Cryst.* B**74**, 196–216. 10.1107/S2052520618002159 29616994

[bb10] Fortes, A. D., Wood, I. G., Grigoriev, D., Alfredsson, M., Kipfstuhl, S., Knight, K. S. & Smith, R. I. (2004). *J. Chem. Phys.* **120**, 11376–11379. 10.1063/1.1765099 15268170

[bb11] Garman, E. (1999). *Acta Cryst.* D**55**, 1641–1653. 10.1107/s0907444999008653 10531512

[bb12] Garman, E. F. & Doublié, S. (2003). *Methods Enzymol.* **368**, 188–216. 10.1016/S0076-6879(03)68011-0 14674275

[bb13] Garman, E. F. & Mitchell, E. P. (1996). *J. Appl. Cryst.* **29**, 584–587.

[bb14] Glass, G. V. (1976). *Educ. Res.* **5**, 3–8.

[bb15] González Solveyra, E., de la Llave, E., Scherlis, D. A. & Molinero, V. (2011). *J. Phys. Chem. B*, **115**, 14196–14204. 10.1021/jp205008w 21863824

[bb16] Grabowski, M., Langner, K. M., Cymborowski, M., Porebski, P. J., Sroka, P., Zheng, H., Cooper, D. R., Zimmerman, M. D., Elsliger, M.-A., Burley, S. K. & Minor, W. (2016). *Acta Cryst.* D**72**, 1181–1193. 10.1107/S2059798316014716 PMC5108346 27841751

[bb17] Ioannidis, J. P. A. (2019). *Am. Stat.* **73**, 20–25.

[bb18] Jenkinson, A. F. (1955). *Q. J. R. Met. Soc.* **81**, 158–171.

[bb19] Kabsch, W. (2010). *Acta Cryst.* D**66**, 125–132. 10.1107/S0907444909047337 PMC2815665 20124692

[bb20] Knudsen, E. B., Sørensen, H. O., Wright, J. P., Goret, G. & Kieffer, J. (2013). *J. Appl. Cryst.* **46**, 537–539.

[bb21] Kriminski, S., Kazmierczak, M. & Thorne, R. E. (2003). *Acta Cryst.* D**59**, 697–708. 10.1107/s0907444903002713 12657789

[bb22] Kuhs, W. F., Sippel, C., Falenty, A. & Hansen, T. C. (2012). *Proc. Natl Acad. Sci. USA*, **109**, 21259–21264. 10.1073/pnas.1210331110 PMC3535660 23236184

[bb23] Kwong, P. D. & Liu, Y. (1999). *J. Appl. Cryst.* **32**, 102–105.

[bb24] Lane, L. B. (1925). *Ind. Eng. Chem.* **17**, 924.

[bb25] Langford, J. I. & Wilson, A. J. C. (1978). *J. Appl. Cryst.* **11**, 102–113.

[bb26] Leslie, A. G. W. (2006). *Acta Cryst.* D**62**, 48–57. 10.1107/S0907444905039107 16369093

[bb27] Li, T., Donadio, D. & Galli, G. (2013). *Nat. Commun.* **4**, 1887. 10.1038/ncomms2918 23695681

[bb28] Lupi, L., Hudait, A., Peters, B., Grünwald, M., Gotchy Mullen, R., Nguyen, A. H. & Molinero, V. (2017). *Nature*, **551**, 218–222. 10.1038/nature24279 29120424

[bb29] Malkin, T. L., Murray, B. J., Salzmann, C. G., Molinero, V., Pickering, S. J. & Whale, T. F. (2015). *Phys. Chem. Chem. Phys.* **17**, 60–76. 10.1039/c4cp02893g 25380218

[bb30] Moreau, D. W., Atakisi, H. & Thorne, R. E. (2019). *IUCrJ*, **6**, 346–356. 10.1107/S2052252519001878 PMC6503922 31098016

[bb31] Nuzzo, R. (2014). *Nature*, **506**, 150–152. 10.1038/506150a 24522584

[bb32] Olivero, J. J. & Longbothum, R. L. (1977). *J. Quant. Spectrosc. Radiat. Transfer*, **17**, 233–236.

[bb33] Owen, R. L., Pritchard, M. & Garman, E. (2004). *J. Appl. Cryst.* **37**, 1000–1003.

[bb34] Panjikar, S. & Tucker, P. A. (2002). *J. Appl. Cryst.* **35**, 117–119.

[bb35] Parkhurst, J. M., Thorn, A., Vollmar, M., Winter, G., Waterman, D. G., Fuentes-Montero, L., Gildea, R. J., Murshudov, G. N. & Evans, G. (2017). *IUCrJ*, **4**, 626–638. 10.1107/S2052252517010259 PMC5619854 28989718

[bb36] Parkhurst, J. M., Winter, G., Waterman, D. G., Fuentes-Montero, L., Gildea, R. J., Murshudov, G. N. & Evans, G. (2016). *J. Appl. Cryst.* **49**, 1912–1921. 10.1107/S1600576716013595 PMC5139990 27980508

[bb37] Pflugrath, J. W. (2004). *Methods*, **34**, 415–423. 10.1016/j.ymeth.2004.03.032 15325658

[bb38] Pflugrath, J. W. (2015). *Acta Cryst.* F**71**, 622–642. 10.1107/S2053230X15008304 PMC4461322 26057787

[bb39] Read, R. J. (1999). *Acta Cryst.* D**55**, 1759–1764. 10.1107/s0907444999008471 10531526

[bb40] Riboldi-Tunnicliffe, A. & Hilgenfeld, R. (1999). *J. Appl. Cryst.* **32**, 1003–1005.

[bb41] Rupp, B. (2009). *Biomolecular Crystallography: Principles, Practice, and Application to Structural Biology*. New York: Garland Science.

[bb42] Stokes, A. R. & Wilson, A. J. C. (1942). *Math. Proc. Camb. Philos. Soc.* **38**, 313–322.

[bb43] Teng, T.-Y. & Moffat, K. (1998). *J. Appl. Cryst.* **31**, 252–257.

[bb44] Thorn, A., Parkhurst, J., Emsley, P., Nicholls, R. A., Vollmar, M., Evans, G. & Murshudov, G. N. (2017). *Acta Cryst.* D**73**, 729–737. 10.1107/S205979831700969X PMC5586246 28876236

[bb45] Thürmer, K. & Bartelt, N. C. (2008). *Phys. Rev. B*, **77**, 195425.

[bb46] Treacy, M. M. J., Newsam, J. M. & Deem, M. W. (1991). *Proc. R. Soc. Lond. A*, **433**, 499–520.

[bb47] Ungár, T., Ott, S., Sanders, P. G., Borbély, A. & Weertman, J. R. (1998). *Acta Mater.* **46**, 3693–3699.

[bb48] Virtanen, P., Gommers, R., Oliphant, T. E., Haberland, M., Reddy, T., Cournapeau, D., Burovski, E., Peterson, P., Weckesser, W., Bright, J., van der Walt, S. J., Brett, M., Wilson, J., Millman, K. J., Mayorov, N., Nelson, A. R. J., Jones, E., Kern, R., Larson, E., Carey, C. J., Polat, Feng, Y., Moore, E. W., VanderPlas, J., Laxalde, D., Perktold, J., Cimrman, R., Henriksen, I., Quintero, E. A., Harris, C. R., Archibald, A. M., Ribeiro, A. H., Pedregosa, F. & van Mulbregt, P. (2020). *Nat. Methods*, **17**, 261–272.

[bb49] Walker, L. J., Moreno, P. O. & Hope, H. (1998). *J. Appl. Cryst.* **31**, 954–956.

[bb50] Warkentin, M., Berejnov, V., Husseini, N. S. & Thorne, R. E. (2006). *J. Appl. Cryst.* **39**, 805–811. 10.1107/S0021889806037484 PMC2866519 20461232

[bb51] Warkentin, M., Sethna, J. P. & Thorne, R. E. (2013). *Phys. Rev. Lett.* **110**, 015703. 10.1103/PhysRevLett.110.015703 23383808

[bb52] Warkentin, M., Stanislavskaia, V., Hammes, K. & Thorne, R. E. (2008). *J. Appl. Cryst.* **41**, 791–797. 10.1107/S0021889808018451 PMC2648606 19529833

[bb53] Warkentin, M. & Thorne, R. E. (2009). *J. Appl. Cryst.* **42**, 944–952. 10.1107/S0021889809023553 PMC2746722 19798409

[bb54] Wasserstein, R. L. & Lazar, N. A. (2016). *Am. Stat.* **70**, 129–133.

