# Supplementary material for: Ice in biomolecular cryocrystallography
Source: Acta Crystallogr D Struct Biol. 2021 Mar 30;77(Pt 4):540–54. doi: 10.1107/S2059798321001170 (PMC8025888; doi:10.1107/S2059798321001170)
Supplement: Supplementary file 1 [file d-77-00540-sup1.pdf]

Acta Cryst D

STRUCTURAL BIOLOGY

Volume 77 (2021)

Supporting information for article:

Ice in biomolecular cryocrystallography

David W. Moreau, Hakan Atakisi and Robert E. Thorne

S1. Protein crystallization

Crystals of equine spleen apoferritin (Sigma, catalogue no. A-3641) were grown in hanging drops consisting of 2 ml of protein at 10 mg ml^{-1} in 0.1 M sodium acetate buffer pH 6.5 and 2 ml of a well solution consisting of 2%(w/v) $CdSO_4$ and 15%(w/v) $(NH_4)_2SO_4$ in the same buffer. Cubic crystals in space group F432 grew to dimensions of 300–500 µm within one week.

Crystals of thaumatin (Sigma, catalogue no. T7638) were grown in hanging drops comprised of equal volumes of protein at 40 mg ml^{-1} in 0.1 M sodium acetate buffer pH 6.5 and a well solution consisting of 14%(w/v) potassium sodium tartrate in the same buffer. Tetragonal crystals in space group $P4_12_12$ grew to dimensions of 200–300 µm within one week.

Crystals of lysozyme (Sigma, catalogue no. L6876) were grown in hanging drops comprised of equal volumes of protein at 80 mg ml^{-1} in 0.1 M sodium acetate buffer pH 5.2 and a well solution consisting of 2.5%(w/v) NaCl in the same buffer. Tetragonal crystals in space group $P4_32_12_1$ grew to dimensions of 300–800 µm. Crystals appeared within one week and stopped growing within four weeks.

S2. Identification of ice diffraction peaks

Ice diffraction peaks associated with individual ice crystals were identified removing protein Bragg peaks from the diffraction frames and then looking for high count pixels near the expected ice ring positions. Potential protein Bragg peaks were masked within a 6-pixel radius circle based on peak locations reported in the XDS output file XDS_ASCII.HKL. Low resolution Bragg peaks and Bragg peaks near the beam stop shadow, rotational axis, and detector panel segments were occasionally excluded in the XDS output file. Regions corresponding to resolutions numerically larger than 10Å, near the beam stop shadow, along the rotational axis and within 15 pixels of the segment edges were thus masked. *pyFAI's separate* program (Ashiotis *et al.*, 2015) was used to remove Bragg peaks from each diffraction image by azimuthal median filtering and then backfilling the pixels associated with the Bragg peaks. All images in the data set were then averaged to produce a mean background image. This background was linearly scaled (multiplied by a constant) to match each frame. Pixels with intensities larger than five times the scaled mean background were identified as outliers, and a histogram of the number of these pixel observations binned against resolution was constructed. The histograms used a bin spacing of 0.01 Å, and the pixel observations were normalized by the total number of such pixels per degree of crystal rotation angle to facilitate comparison of histograms between data sets. Table 1 lists the resolutions of the 11 diffraction rings of hexagonal ice, where ice diffraction peaks should be located, based on 100 K unit cell parameters of a=4.497Å, b=7.322Å (Fortes, 2018).

S3. IRRMC data sets analyzed for ice type

The 22 IRRMC data sets analyzed in Section 2.4 for ice type by fitting using a mixture of cubic and hexagonal ice or models of stacking disordered ice were all recorded on Dectris Pilatus 6M detectors. Of these data sets, 14 were recorded using beamline BL11-1 at SSRL, 4 using beamline BL12-2 at SSRL, 2 using beamline 19-ID at APS, 1 using beamline 5.0.1 at ALS and 1 using beamline I04-1 at Diamond. These 22 data sets include the 13 data sets used by (Parkhurst *et al.*, 2017). All contained clear visual evidence of ice diffraction.

S4. Ice crystallite size determination

S4.1. Scherrer's constant

The apparent crystallite size in Scherrer's equation is related to the actual crystallite size, p, (the cube root of the crystal volume) by $p = K\delta$. The proportionality constant K is known as Scherrer's constant (Langford & Wilson, 1978), and depends on crystallite shape, crystal symmetry, Miller index and the definition of peak breadth. Scherrer's constant for a cubic crystal's (100), (110), and (111) reflections are 1.0000, 1.0607, and 1.1547 respectively (Langford & Wilson, 1978). K is assumed to be 1 for this analysis.

S4.2. Hexagonal ice calibrant

The hexagonal ice sample was generated *in situ* at the F1 CHESS station as follows. First, a 30% w/w polypropylene glycol 425 solution in a 500 μm diameter PET tube was abruptly cooled to T=200 K by unshuttering a cold gas stream. Abrupt cooling to a temperature where the ice nucleation rate was high resulted in an ideal powder pattern with a large width (indicating a small crystallite size) and having a stacking disordered intensity profile. As this sample was slowly warmed, the intensity profile evolved from stacking disordered to purely hexagonal (Kuhs *et al.*, 2012) and the peak breadths decreased (Figure S3). Above 250 K, the uniformity of the diffraction rings was lost, and each ring eventually broke up into a collection of individual Bragg peaks. The diffraction pattern recorded at 250 K (Figure S4(a)), before the loss of ring uniformity, was used to estimate the instrumental broadening.

The use of this ice sample as a calibrant does not correct broadening due to uncertainty in the experimental geometry, specifically in the assumed beam center and angle between the incident X-ray beam direction and detector normal; these modify the apparent angle of each ice peak and thus the apparent angular width. Estimates of these parameters were improved by adjusting their values to minimize the sum of the breadths of all observed ice rings. For the hexagonal calibrant, this yielded a detector tilt of 0.1° from the incident X-ray beam normal. The calibrant's corrected breadths are shown

in Figure S4 (b) and have an average of 0.037°. This breadth was then taken as an estimate of the instrumental broadening due to the incident X-ray beam divergence and energy dispersion.

The broadening due to beam divergence and energy dispersion were independently estimated for comparison. Beam divergence α adds linearly to the peak widths, $\Delta 2\theta \approx \alpha$. Broadening due to beam dispersion is estimated from Bragg's Law through uncertainty propagation,

$$\begin{aligned}\Delta 2\theta &\approx 2\frac{1}{\sqrt{1-\left(\lambda/2d\right)^2}}\frac{\lambda}{2d}\frac{\Delta\lambda}{\lambda} \\ &\approx 2\cdot\tan\left(\theta\right)\frac{\Delta\lambda}{\lambda}\end{aligned}.$$

For the F1 station, the beam divergence $\alpha = 0.028^o$, and the dispersion $\Delta\lambda/\lambda = 0.00173$ corresponds to a broadening of 0.027°. The observed hexagonal ice peaks of the calibrant sample appeared Gaussian, indicating these two factors should add in quadrature. This gives a combined broadening of 0.04°, on par with the measured peak breadth of 0.037°.

S4.3. Number of ice crystallites required for continuous diffraction rings

The minimum number of ice crystallites required to generate azimuthally continuous diffraction rings can be estimated from the illuminated sample volume and the incident beam divergence. This estimate can in turn be used to set a bound on the crystallite size. Assuming the ice has a sufficiently large grain size that its Bragg peaks have a breadth in the azimuthal direction determined solely by the beam divergence, α, the peak's arc length is

$$\Delta = F\tan\left(\alpha\right).$$

F is the sample-to-detector distance. The circumference of the diffraction ring is

$$L = 2\pi F\tan\left(2\theta\right).$$

If placed end to end, the number of crystallites needed to generate a complete diffraction ring is $N \approx L/\Delta$. This is multiplied by 10 to ensure there is significant overlap between the Bragg peaks to give

$$N \approx 10\cdot 2\pi\frac{\tan\left(2\theta\right)}{\tan\left(\alpha\right)}.$$

This corresponds to 74,000 crystallites for a diffraction peak at $2\theta = 30^o$ and a measured beam divergence of $\alpha = 0.028^o$ for the CHESS station used in our experiments on apoferritin, thaumatin and lysozyme. For a 100 μm diameter beam and a 500 μm path length, the illuminated volume is $V \approx 3.9\cdot 10^6$ μm^3. Dividing this total volume by 74,000 gives an upper bound on the crystallite volume of 53 μm^3

or, assuming a spherical crystallite, a diameter of 4.6 µm. This crystallite size gives a finite-size broadening of $\beta \approx 0.0013^{o}$, 45 times smaller than the observed hexagonal ice peak breadth, suggesting that the observed peak breadth is limited by the instrumental broadening, as assumed in this estimate.

S4.4. Source broadening subtraction

The broadening of the observed (detector angle and beam-stop position corrected) ice diffraction peaks is a convolution of the intrinsic and instrumental broadenings. The increase in breadth of a peak following a convolution depends on the shape of the functions involved. For convolutions of Gaussians, the resulting profile is Gaussian, and the breadths add in quadrature; for Lorentzians, the breadths add linearly. The convolution of a Gaussian with a breadth of β_{gauss} and a Lorentzian width a breadth of β_{lor} is a Voight profile with a breadth of (Olivero & Longbothum, 1977)

$$\beta_{voigt} \approx 0.5346\beta_{lor} + \sqrt{0.2166\beta_{lor}^2 + \beta_{gauss}^2}\,.$$

Peak profiles of the hexagonal ice calibrant were best modelled by Gaussians and are likely dominated by instrumental factors. Peak profiles for ice internal to the protein crystals were best modelled by Lorentzians. This implies that the intrinsic broadening from crystallite size and shape is not Gaussian. While we cannot be certain of its shape, we assumed it is Lorentzian because it is the dominant broadening factor. In this case, the observed breadths are β_{voigt}, the instrumental broadening estimated from the hexagonal calibrant peaks is β_{gauss}, and the desired breadth of the internal ice with instrumental effects deconvolved is β_{lor} and is given by

$$\beta_{lor} \approx \frac{\beta_{voigt}^2 - \beta_{gaus}^2}{\beta_{voigt}}\,.$$

S4.5. Size estimate of ice crystals contributing to outlier pixels

A lower bound on the size of an ice crystal that contributes to a single pixel outlier was calculated with Scherrer's equation using the angular breadth of a single pixel. For a diffraction image recorded at a sample-to-detector distance of F, wavelength of λ and pixel size μ, the angle subtended by a single pixel is

$$\beta = \tan^{-1}\left(\left(N+1\right)\mu/F\right) - \tan^{-1}\left(N\,\mu/F\right).$$

Given a diffraction angle of θ, N is the radial distance of the outlier pixel from the detector's beam center location in units of pixels

$$N = \frac{F}{\mu}\tan\left(2\theta\right).$$

The breadth can be used in Scherrer's equation to determine a lower bound on the crystallite size as

$$\delta = \frac{\lambda}{\beta \cdot \cos\left(\theta\right)}.$$

Using $F = 500$ mm, μ=0.172 mm and $\lambda = 1$ Å, the crystallite size for the (112) hexagonal ice peak at 1.916 Å resolution is ~4,000 Å.

S5. Ice Detection from structure factors

S5.1. Ice identification only at three locations

This choice to focus on the three ice ring locations common to all forms of ice, rather than all 11 locations of hexagonal ice, improved the robustness of the interpolations through the ice rings and from the 0.01 Å^{-1} to 0.0025 Å^{-1} bins. After binning the values in 0.01 Å^{-1} bins, the bins near the ice peak locations are excluded from further consideration, and the remaining bins are used to interpolate through these excluded regions. As the total number of regions where ice is being searched for increases, more regions need to be interpolated through but there are fewer neighbouring bins to guide the interpolation. Using all 11 ice ring locations often led to erroneous interpolations; interpolations were improved by focusing on the three ice ring locations common to all ice forms. In the case of cubic-like or stacking disordered ice, only ice diffraction at these three locations tends to be strong enough to bias the values, so there is no down-side to this choice. Hexagonal ice produces much narrower and taller peaks at all locations that strongly bias the values, so ice is easily detectable even when only three locations are examined.

S5.2. Comparison between Ice Finder, Depletion and Observation scores

The Ice Finder Score, Depletion Score and Observation score could be used independently to predict the presence of ice biasing in a data set. This section demonstrates that a combination of these scores performs better than a single score. The weighted average of the maximum IFS and DS were calculated for each entry in the training data set and Generalized Extreme Value Distributions were fit to these sets of weighted averages. These distributions were used to predict the presence of ice in the 200 entry test set used by Thorn *et al*. and our 200 entry ground truth test set. Table S6 shows the number of false negatives and positives for each of these analyses. A combined metric, p_{ice}, to flag a data set as biased by ice outperformed the metrics on their own.

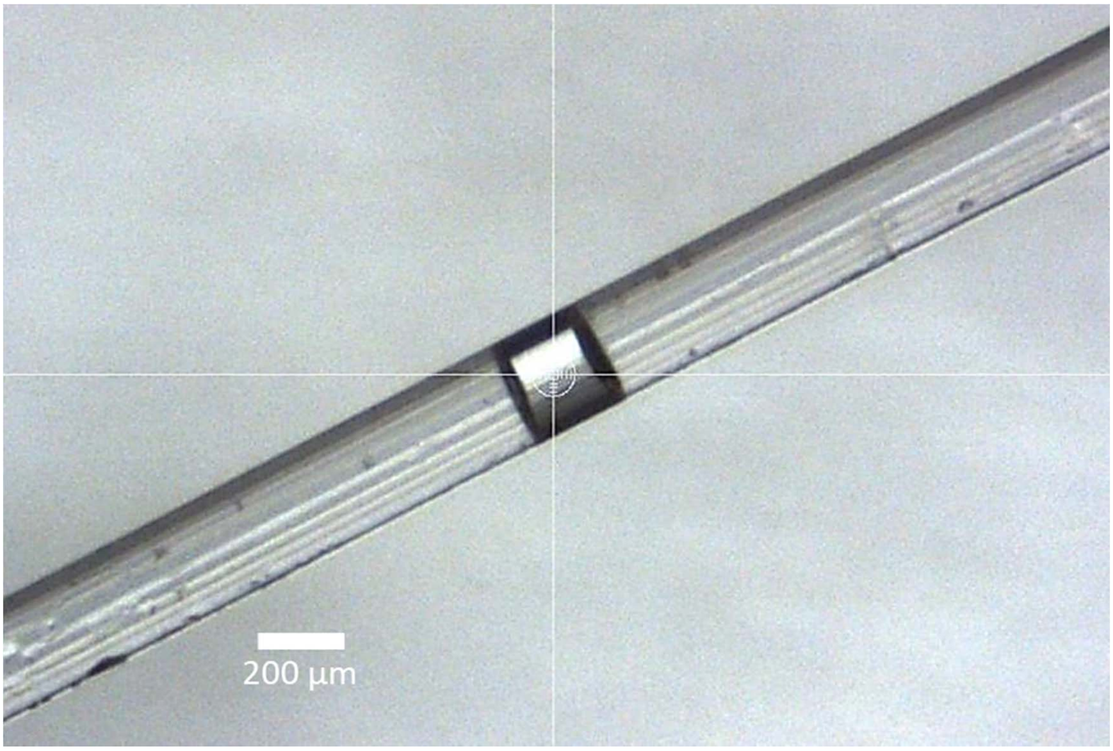

Figure S1 Collection of X-ray diffraction data from cryocooled glycerol / water solutions. Samples were prepared by injecting ~10 nl of solution into a 250 µm diameter, ~2 cm long thin-walled polyester tube. The length of tubing filled with solution was approximately 200 µm. The tube was then affixed to a goniometer base (Mitegen GB-B1A) and centered in the X-ray beam.

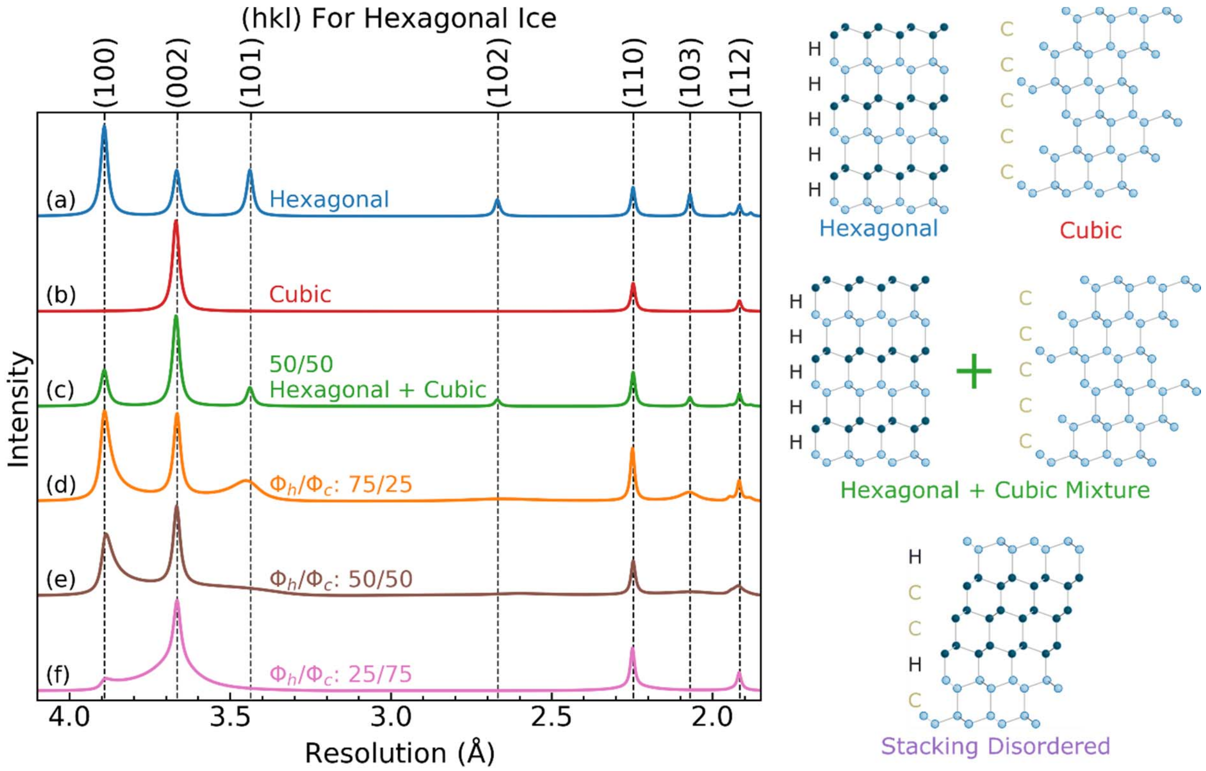

Figure S2 Left: Theoretical X-ray diffraction patterns for different types of ice generated using DIFFaX. Vertical lines show the (hkl) indices for hexagonal ice. The different types of ice are: (a) pure hexagonal, (b) pure cubic, (c) 50/50 mixture of pure hexagonal and pure cubic ice, (d) stacking disordered ice with 75% hexagonal stacking and 25% cubic stacking, (e) stacking disordered ice with 50% hexagonal and cubic stackings, (f) stacking disordered ice with 25% hexagonal stacking and 75% cubic stacking. Right: 2D visualizations of the different types of ice and how they stack. Hexagonal ice, cubic ice, and stacking disordered ice are made of two different types of atomic planes and only differ in how the planes stack. Hexagonal ice is an alternate stacking of the two atomic planes. Cubic ice can be made from either plane by shifting successive planes in the stack by 1/3 of the unit cell. In stacking disordered ice, the two types of planes stack randomly.

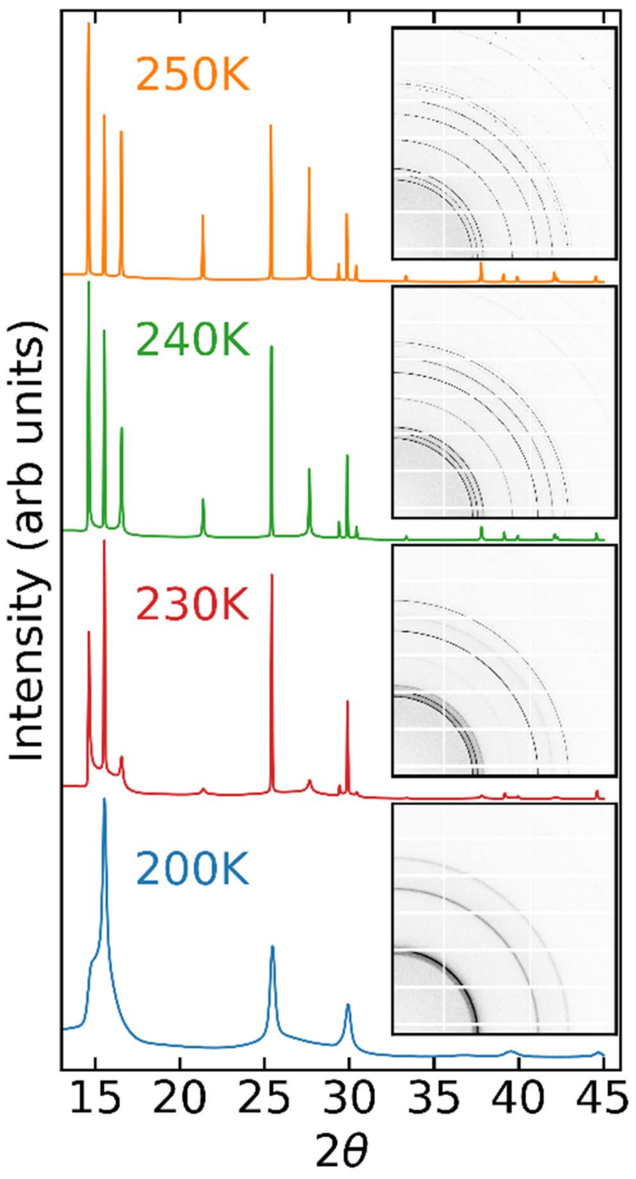

Figure S3 Generating a sample that yields pure hexagonal ice diffraction. First, a 30% w/w polypropylene glycol 425 solution in a 500 µm diameter PET tube was abruptly cooled to T=200 K by unshuttering a cold gas stream. Abrupt cooling to a temperature where the ice nucleation rate was high resulted in an ideal powder pattern with a large width (indicating a small crystallite size) and having a stacking disordered intensity profile. As this sample was slowly warmed, the intensity profile evolved from stacking disordered to purely hexagonal (Kuhs et al., 2012) and the peak breadths decreased, indicating a growth in ice grain size. Even though the solution contains propylene glycol, this component is rejected during ice growth and the ice crystals are comprised of pure water.

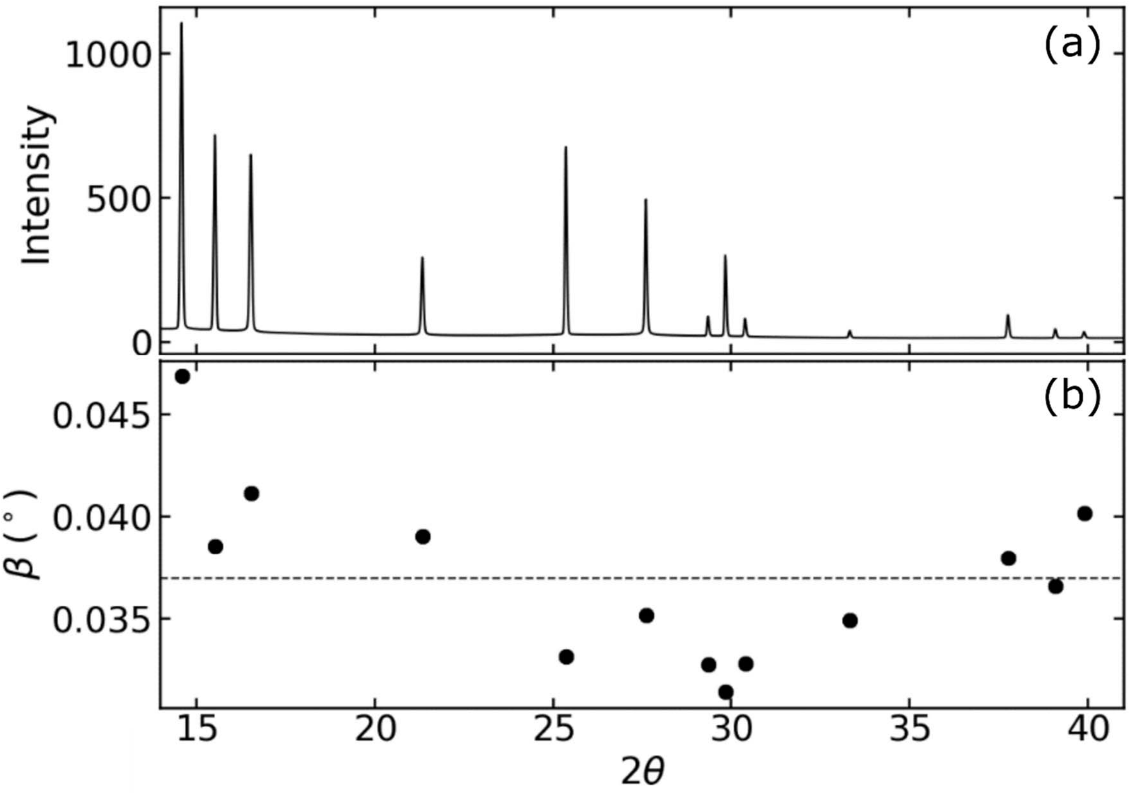

Figure S4 (a) The diffraction pattern recorded from the hexagonal ice calibration sample at 250 K, before the loss of ring uniformity. (b) Peak widths in (a), used to estimate the instrumental broadening.

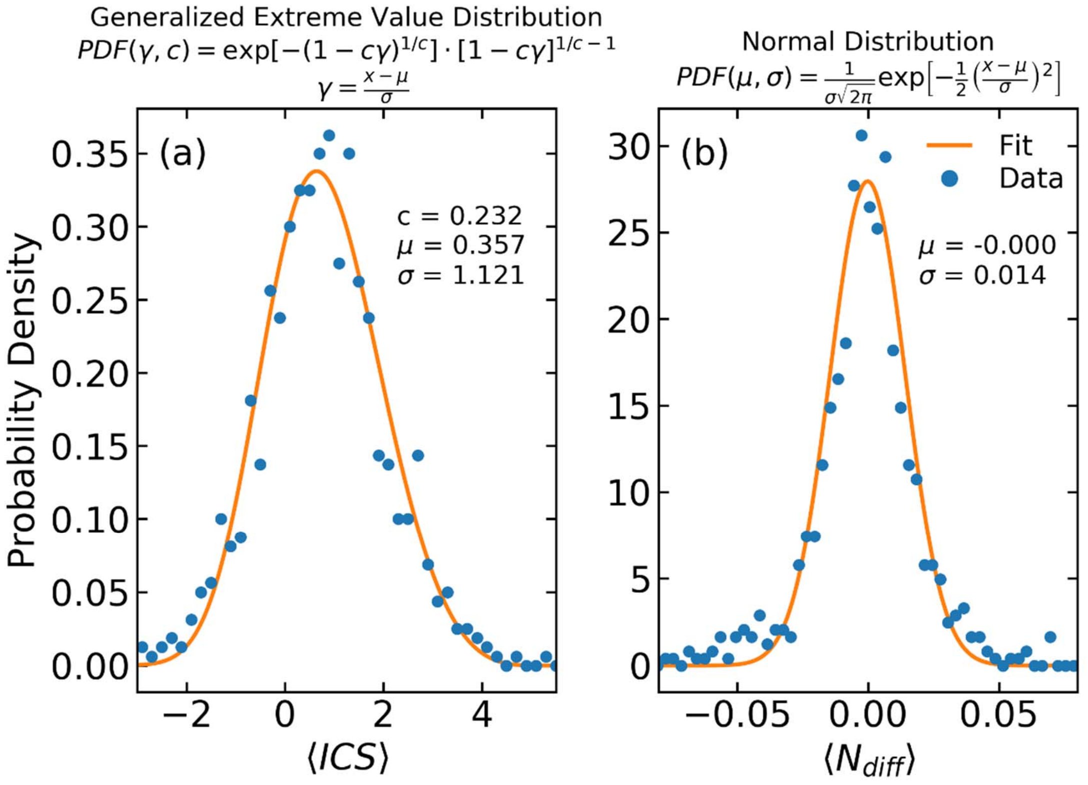

Figure S5 Histogram and fits to the Generalized Extreme Value (GEV) Distribution for (a) $\langle ICS \rangle$ and the Normal Distribution for (b) $\langle DS \rangle$. The fit equation is given above the histograms, and the fit parameters are given in the legend of each histogram.

Table S1 HKL values of ice peaks common to all forms of ice and corresponding resolution ranges that were examined for evidence of ice.

(hkl)	Interpolation Resolution Range (Å)	Search Resolution Range (Å)
(002)	3.753 – 3.581	3.686 – 3.636
(110)	2.294 – 2.209	2.261 – 2.236
(112)	1.935 – 1.897	1.926 – 1.906

Table S2 Parameters used in calculating the weightings ω_{hkl} of *ICS* values at the resolution of each ice diffraction ring into an average $\langle ICS \rangle$, given by Eq. (8).

(hkl)	Resolution (Å)	m_{hkl}	F_{hkl}	B_{ice} (Å^2)	B_p (Å^2)	ω_{hkl} Absolute	ω_{hkl} Relative
Initial Ice Detection							
(002)	3.661	2	21.84			3323	1
(110)	2.249	6	21.32	1.5	35	74804	22.5
(112)	1.916	12	14.07			227684	68.5
Hexagonal Ice Detection							
(101)	3.438	12	11.74			6879	1
(102)	2.668	12	4.330	1.5	35	2366	0.34
(103)	2.068	12	14.21			121715	17.7

Table S3 Outlier pixels identified in diffraction images of 60 IRRMC archive data sets. 26 data sets had ice rings visible in diffraction images, and 34 did not have visible ice rings. "Cubic" ice rings are at the positions of the three ice rings expected for pure cubic ice; hexagonal ice and stacking disordered ice also have rings at these positions. "Hexagonal-only ice rings" refers to the ice ring positions not common to hexagonal and cubic ice, but that may be present in stacking disordered ice having a large hexagonal plane fraction.

	Number of outlier pixels at			Fraction of outlier pixels at		
PDB ID	Any resolution	"Cubic" ice rings	Hexagonal-only ice rings	All ice rings	Hexagonal-only ice rings	Ice Spots per degree
			Hexagonal Ice			
4ezg	22255	2909	3180	0.27	0.13	22
4opm	14907	5176	4780	0.67	0.35	42
4puc	7723	2353	1328	0.48	0.30	15
5uba	13405	1986	6247	0.61	0.15	22
4q1z	7277	1552	4974	0.90	0.21	41
6ck7	29774	2172	2014	0.14	0.07	11
			Stacking disordered / cubic ice			
4dn6	1590	470	348	0.51	0.30	4
4e6e	1919	828	666	0.78	0.43	5
4ef1	11117	1089	918	0.18	0.10	5
4epz	1723	491	409	0.52	0.28	4
4fmr	46374	6581	2561	0.20	0.14	7
4hf7	1524	391	428	0.54	0.26	5
4iej	42371	498	0	0.01	0.01	0
4kw2	4633	1107	1395	0.54	0.24	8
4mjg	645	158	76	0.36	0.24	1
4ps6	7075	2868	1813	0.66	0.41	11
4h3w	44400	24675	15525	0.91	0.56	44
4poi	7817	232	104	0.04	0.03	1
5kh9	72775	2963	1296	0.06	0.04	11
4j8q	2991	1417	1080	0.83	0.47	10
4n0p	7312	373	190	0.08	0.05	1
5mq6	11718	775	1087	0.16	0.07	6
4qu7	655	183	128	0.47	0.28	2
4h4j	67091	2925	1150	0.06	0.04	6
4ps6	7075	2868	1813	0.66	0.41	11
4kh8	5557	1258	1032	0.41	0.23	9
			No Ice			
4hxc	4273	806	764	0.37	0.19	18
4ei0	16034	965	710	0.10	0.06	6
4kwy	3008	1025	1000	0.67	0.34	8
6cw0	11330	699	345	0.09	0.06	2
4z2x	1538	383	361	0.48	0.25	4

5m4l	54246	2185	756	0.05	0.04	8
6f3q	97265	2441	1179	0.04	0.03	12
4fss	4165	1173	714	0.45	0.28	6
4e2e	971	160	159	0.33	0.16	1
4qu6	4060	1395	1470	0.71	0.34	9
5vbd	3778	472	516	0.26	0.12	2
5vbt	8130	562	409	0.12	0.07	1
4j5o	21624	920	615	0.07	0.04	4
4m0h	168	0	12	0.07	0.00	0
4obi	2954	952	1000	0.66	0.32	10
4oo3	2090	867	765	0.78	0.41	9
4ygu	11473	1045	906	0.17	0.09	4
4yod	6834	1480	1067	0.37	0.22	6
4mru	26462	3354	760	0.16	0.13	3
4f53	2591	1024	598	0.63	0.40	6
4fdy	5814	1060	687	0.30	0.18	4
4kh9	7262	1820	2179	0.55	0.25	6
4g2a	7315	2478	1559	0.55	0.34	18
4lr4	15429	713	722	0.09	0.05	4
5bxg	9359	4896	2968	0.84	0.52	53
4exr	1239	617	400	0.82	0.50	5
4ecf	28943	1646	487	0.07	0.06	3
4nw4	1735	654	452	0.64	0.38	4
4pwu	623	15	39	0.09	0.02	0
4lg3	6452	795	941	0.27	0.12	7
4q0y	4686	523	480	0.21	0.11	4
4lqz	1835	422	318	0.40	0.23	3
4jm1	1985	96	93	0.10	0.05	1
4q34	3641	100	59	0.04	0.03	1

Table S4 Comparison of ice classification results obtained using our p_{ice} and AUSPEX with our visual assessment of I_{obs} values as in Fig. 1(b), for 198 of the 200 PDB entries used by Thorn et al.

			Classifications			P_{ice}		AUSPEX	
PDB ID	p_{ice}	p_n	P_{ice}	AUSPEX	Structure Factor Plots	False N	False P	False N	False P
1rdr	0.389	0.927	FALSE			FALSE	FALSE	FALSE	FALSE
1c6h	0.538	0.092	FALSE			FALSE	FALSE	FALSE	FALSE
1dcr	0.582	0.000	TRUE	TRUE		FALSE	TRUE	FALSE	TRUE
1eqm	0.719	0.332	FALSE			FALSE	FALSE	FALSE	FALSE
1r0q	0.448	0.133	FALSE			FALSE	FALSE	FALSE	FALSE
1o1p	0.665	0.701	FALSE			FALSE	FALSE	FALSE	FALSE
1jg1	0.756	0.721	FALSE			FALSE	FALSE	FALSE	FALSE
1sjb	0.987	0.556	FALSE	TRUE		FALSE	FALSE	FALSE	TRUE
1vat	0.138	0.774	FALSE			FALSE	FALSE	FALSE	FALSE
1k34	0.733	1.000	FALSE			FALSE	FALSE	FALSE	FALSE
2xyb	0.276	0.039	FALSE			FALSE	FALSE	FALSE	FALSE
2gn9	0.017	0.649	FALSE			FALSE	FALSE	FALSE	FALSE
1n6l	0.580	0.715	FALSE			FALSE	FALSE	FALSE	FALSE
1ym0	0.380	0.547	FALSE		FALSE[1]	FALSE	FALSE	FALSE	FALSE
2avq	0.453	0.533	FALSE			FALSE	FALSE	FALSE	FALSE
2bsm	0.215	0.011	FALSE	TRUE		FALSE	FALSE	FALSE	TRUE
2h9y	0.457	0.493	FALSE			FALSE	FALSE	FALSE	FALSE
1zzo	0.586	0.964	FALSE			FALSE	FALSE	FALSE	FALSE
2fbd	0.971	0.918	FALSE			FALSE	FALSE	FALSE	FALSE
3n2u	0.023	0.438	FALSE		TRUE	TRUE	FALSE	TRUE	FALSE
1yi1	0.523	0.635	FALSE			FALSE	FALSE	FALSE	FALSE
2fg4	0.244	0.574	FALSE			FALSE	FALSE	FALSE	FALSE
1yum	0.002	0.140	TRUE	TRUE	TRUE	FALSE	FALSE	FALSE	FALSE
1yg9	0.514	0.964	FALSE			FALSE	FALSE	FALSE	FALSE
1ziq	0.084	0.065	FALSE	TRUE	TRUE	TRUE	FALSE	FALSE	FALSE
1w96	0.198	0.715	FALSE			FALSE	FALSE	FALSE	FALSE
3fk3	0.996	0.105	FALSE			FALSE	FALSE	FALSE	FALSE
1vq8	0.000	0.501	TRUE	TRUE	TRUE	FALSE	FALSE	FALSE	FALSE
2b15	0.501	0.126	FALSE			FALSE	FALSE	FALSE	FALSE
2i2z	0.583	0.001	FALSE			FALSE	FALSE	FALSE	FALSE
1xea	0.006	0.788	FALSE		TRUE	TRUE	FALSE	TRUE	FALSE
2wkn	0.918	0.658	FALSE			FALSE	FALSE	FALSE	FALSE
1z6l	0.000	0.975	TRUE		TRUE	FALSE	FALSE	TRUE	FALSE
2w5p	0.312	0.003	FALSE			FALSE	FALSE	FALSE	FALSE
2fi4	0.498	0.730	FALSE			FALSE	FALSE	FALSE	FALSE
2zu2	0.118	0.396	FALSE			FALSE	FALSE	FALSE	FALSE
2o6m	0.990	0.792	FALSE			FALSE	FALSE	FALSE	FALSE
3bt0	0.452	0.252	FALSE			FALSE	FALSE	FALSE	FALSE
2qex	0.887	0.341	FALSE			FALSE	FALSE	FALSE	FALSE
2hd6	0.102	0.994	FALSE			FALSE	FALSE	FALSE	FALSE
2npz	0.999	0.682	FALSE	TRUE		FALSE	FALSE	FALSE	TRUE
2d5n	0.947	0.812	FALSE			FALSE	FALSE	FALSE	FALSE
3p88	0.736	0.948	FALSE			FALSE	FALSE	FALSE	FALSE
2ew0	0.167	0.930	FALSE			FALSE	FALSE	FALSE	FALSE
2gp9	0.553	0.337	FALSE			FALSE	FALSE	FALSE	FALSE
2gb9	0.316	0.000	TRUE			FALSE	TRUE	FALSE	FALSE

2rcu	1.000	0.000	TRUE		TRUE[1]	FALSE	FALSE	FALSE	FALSE
3vhr	0.851	0.843	FALSE			FALSE	FALSE	FALSE	FALSE
2pi5	0.980	0.677	FALSE			FALSE	FALSE	FALSE	FALSE
2z6a	0.936	0.832	FALSE			FALSE	FALSE	FALSE	FALSE
3i9u	0.002	0.780	TRUE			FALSE	TRUE	FALSE	FALSE
2g6q	0.647	0.189	FALSE			FALSE	FALSE	FALSE	FALSE
3bbm	0.451	0.343	FALSE			FALSE	FALSE	FALSE	FALSE
3vhc	0.257	0.207	FALSE			FALSE	FALSE	FALSE	FALSE
4krd	0.690	0.748	FALSE			FALSE	FALSE	FALSE	FALSE
3ehb	0.000	0.225	TRUE	TRUE	TRUE	FALSE	FALSE	FALSE	FALSE
4pmn	0.353	0.483	FALSE			FALSE	FALSE	FALSE	FALSE
2pnx	0.442	0.024	FALSE			FALSE	FALSE	FALSE	FALSE
2vw9	0.000	0.000	TRUE		TRUE	FALSE	FALSE	TRUE	FALSE
5fdq	0.854	0.297	FALSE			FALSE	FALSE	FALSE	FALSE
3dre	0.436	0.456	FALSE			FALSE	FALSE	FALSE	FALSE
2zks	0.774	0.987	FALSE		FALSE[1]	FALSE	FALSE	FALSE	FALSE
3ifu	0.262	0.000	TRUE			FALSE	TRUE	FALSE	FALSE
3a65	0.780	0.718	FALSE			FALSE	FALSE	FALSE	FALSE
3det	0.987	0.551	FALSE			FALSE	FALSE	FALSE	FALSE
3tog	0.775	0.304	FALSE	TRUE		FALSE	FALSE	FALSE	TRUE
3gl9	0.393	0.618	FALSE			FALSE	FALSE	FALSE	FALSE
2xte[2]	N/A	N/A							
4ex6	0.644	0.184	FALSE			FALSE	FALSE	FALSE	FALSE
3m9s[2]	N/A	N/A							
3n04	0.091	0.618	FALSE			FALSE	FALSE	FALSE	FALSE
4dw4	0.000	0.042	TRUE	TRUE	TRUE[1]	FALSE	FALSE	FALSE	FALSE
5d1p	0.989	0.074	FALSE			FALSE	FALSE	FALSE	FALSE
3rl5	0.529	0.102	FALSE			FALSE	FALSE	FALSE	FALSE
3hgy	0.078	0.780	FALSE			FALSE	FALSE	FALSE	FALSE
2x13	0.324	0.227	FALSE			FALSE	FALSE	FALSE	FALSE
5cdt	0.000	0.165	TRUE			FALSE	TRUE	FALSE	FALSE
3hnm	0.223	0.770	FALSE		FALSE[1]	FALSE	FALSE	FALSE	FALSE
4nt2	0.995	0.544	FALSE			FALSE	FALSE	FALSE	FALSE
3jqy	0.000	0.329	TRUE	TRUE	TRUE	FALSE	FALSE	FALSE	FALSE
3rmo	0.858	0.781	FALSE	TRUE		FALSE	FALSE	FALSE	TRUE
5fn8	0.910	0.997	FALSE			FALSE	FALSE	FALSE	FALSE
3t8v	0.320	0.422	FALSE			FALSE	FALSE	FALSE	FALSE
3ncg	0.000	0.000	TRUE	TRUE	TRUE	FALSE	FALSE	FALSE	FALSE
3nhw	0.426	0.915	FALSE			FALSE	FALSE	FALSE	FALSE
3mnl	0.417	0.369	FALSE			FALSE	FALSE	FALSE	FALSE
4f27	0.000	0.563	TRUE	TRUE	TRUE	FALSE	FALSE	FALSE	FALSE
4l17	0.096	0.807	FALSE			FALSE	FALSE	FALSE	FALSE
4ea7	0.471	0.821	FALSE	TRUE		FALSE	FALSE	FALSE	TRUE
3b1j	0.348	0.761	FALSE			FALSE	FALSE	FALSE	FALSE
3lz0	0.427	0.151	FALSE	TRUE	TRUE	TRUE	FALSE	FALSE	FALSE
4ac2	0.003	0.118	TRUE		TRUE	FALSE	FALSE	TRUE	FALSE
3x42	0.807	0.978	FALSE			FALSE	FALSE	FALSE	FALSE
4nzg	0.000	0.035	TRUE	TRUE	TRUE	FALSE	FALSE	FALSE	FALSE
3myt	0.000	0.000	TRUE	TRUE	TRUE	FALSE	FALSE	FALSE	FALSE
3osz	0.746	0.685	FALSE			FALSE	FALSE	FALSE	FALSE
3mt0	0.255	0.902	FALSE			FALSE	FALSE	FALSE	FALSE
2xwa	0.519	0.996	FALSE			FALSE	FALSE	FALSE	FALSE

4ha4	0.388	0.295	FALSE			FALSE	FALSE	FALSE	FALSE
3q3o	0.926	0.410	FALSE			FALSE	FALSE	FALSE	FALSE
3nvs	0.953	0.375	FALSE	TRUE	FALSE[1]	FALSE	FALSE	FALSE	FALSE
4h5b	0.024	0.856	FALSE			FALSE	FALSE	FALSE	FALSE
3o2n	0.204	0.808	FALSE			FALSE	FALSE	FALSE	FALSE
4a14	0.250	0.706	FALSE			FALSE	FALSE	FALSE	FALSE
5kc5	0.096	0.110	FALSE			FALSE	FALSE	FALSE	FALSE
4f5j	0.375	0.904	FALSE			FALSE	FALSE	FALSE	FALSE
4gfa	0.707	0.475	FALSE			FALSE	FALSE	FALSE	FALSE
3po9	0.945	0.995	FALSE			FALSE	FALSE	FALSE	FALSE
4elp	0.471	0.239	FALSE			FALSE	FALSE	FALSE	FALSE
3tal	0.030	0.297	FALSE			FALSE	FALSE	FALSE	FALSE
3zym	0.011	0.461	FALSE		TRUE	TRUE	FALSE	TRUE	FALSE
3us1	0.866	1.000	FALSE			FALSE	FALSE	FALSE	FALSE
4nxn	1.000	0.790	FALSE	TRUE		FALSE	FALSE	FALSE	TRUE
3sbi	0.572	0.998	FALSE			FALSE	FALSE	FALSE	FALSE
3sya	0.049	0.065	FALSE			FALSE	FALSE	FALSE	FALSE
4gba	0.004	0.465	TRUE	TRUE	TRUE	FALSE	FALSE	FALSE	FALSE
3r3m	0.157	0.242	FALSE			FALSE	FALSE	FALSE	FALSE
4e2w	0.417	0.052	FALSE	TRUE		FALSE	FALSE	FALSE	TRUE
3rib	0.945	0.086	FALSE			FALSE	FALSE	FALSE	FALSE
4bi2	0.512	0.054	FALSE			FALSE	FALSE	FALSE	FALSE
3sv9	0.703	0.246	FALSE			FALSE	FALSE	FALSE	FALSE
3suf	0.784	0.962	FALSE			FALSE	FALSE	FALSE	FALSE
4mcf	0.723	0.598	FALSE			FALSE	FALSE	FALSE	FALSE
4mw7	0.176	0.566	FALSE			FALSE	FALSE	FALSE	FALSE
4jc7	0.625	0.012	FALSE			FALSE	FALSE	FALSE	FALSE
4miy	0.527	0.489	FALSE			FALSE	FALSE	FALSE	FALSE
4x7k	0.771	0.503	FALSE			FALSE	FALSE	FALSE	FALSE
4es4	0.623	0.178	FALSE			FALSE	FALSE	FALSE	FALSE
4k37	0.003	0.310	TRUE		TRUE	FALSE	FALSE	TRUE	FALSE
3u12	0.003	0.068	TRUE			FALSE	TRUE	FALSE	FALSE
4afd	0.959	0.969	FALSE			FALSE	FALSE	FALSE	FALSE
4m1o	0.113	0.241	FALSE			FALSE	FALSE	FALSE	FALSE
4tvq	0.000	0.355	TRUE		TRUE	FALSE	FALSE	TRUE	FALSE
4mb7	0.270	0.660	FALSE			FALSE	FALSE	FALSE	FALSE
5c1x	0.180	0.872	FALSE			FALSE	FALSE	FALSE	FALSE
4lo3	0.967	0.013	FALSE			FALSE	FALSE	FALSE	FALSE
4hkx	0.000	0.897	TRUE	TRUE		FALSE	TRUE	FALSE	TRUE
3we4	0.277	0.446	FALSE			FALSE	FALSE	FALSE	FALSE
4wie	0.000	0.459	TRUE		TRUE	FALSE	FALSE	TRUE	FALSE
4iaz	0.041	0.397	FALSE			FALSE	FALSE	FALSE	FALSE
3wf7	0.242	0.844	FALSE			FALSE	FALSE	FALSE	FALSE
4kgr	0.453	1.000	FALSE	TRUE		FALSE	FALSE	FALSE	TRUE
4isz	0.303	0.601	FALSE			FALSE	FALSE	FALSE	FALSE
4ipg	0.400	0.999	FALSE			FALSE	FALSE	FALSE	FALSE
4la6	0.789	0.592	FALSE			FALSE	FALSE	FALSE	FALSE
4ly8	0.890	0.318	FALSE	TRUE	FALSE[1]	FALSE	FALSE	FALSE	FALSE
4j7y	0.000	0.996	TRUE	TRUE	TRUE	FALSE	FALSE	FALSE	FALSE
4ikm	0.876	0.977	FALSE			FALSE	FALSE	FALSE	FALSE
4hjx	0.252	0.908	FALSE			FALSE	FALSE	FALSE	FALSE
3w7v	0.712	0.211	FALSE	TRUE		FALSE	FALSE	FALSE	TRUE

4q75	0.002	0.757	TRUE		TRUE	FALSE	FALSE	TRUE	FALSE	
4mvs	1.000	0.662	FALSE			FALSE	FALSE	FALSE	FALSE	
4j29	0.308	0.374	FALSE			FALSE	FALSE	FALSE	FALSE	
4x6b	0.280	0.929	FALSE	TRUE	FALSE[1]	FALSE	FALSE	FALSE	FALSE	
4ou1	0.000	0.185	TRUE	TRUE	TRUE	FALSE	FALSE	FALSE	FALSE	
4l55	0.338	0.809	FALSE			FALSE	FALSE	FALSE	FALSE	
4nz0	0.037	0.731	FALSE			FALSE	FALSE	FALSE	FALSE	
4ccm	0.000	0.595	TRUE		TRUE	FALSE	FALSE	TRUE	FALSE	
4oq4	0.977	0.206	FALSE			FALSE	FALSE	FALSE	FALSE	
4ycu	0.000	0.706	TRUE		TRUE	FALSE	FALSE	TRUE	FALSE	
4m00	0.000	0.156	TRUE	TRUE	TRUE	FALSE	FALSE	FALSE	FALSE	
4q3o	0.000	0.574	TRUE	TRUE	TRUE	FALSE	FALSE	FALSE	FALSE	
4xet	0.065	0.002	FALSE			FALSE	FALSE	FALSE	FALSE	
4p66	0.879	0.525	FALSE			FALSE	FALSE	FALSE	FALSE	
4x8w	0.177	0.781	FALSE			FALSE	FALSE	FALSE	FALSE	
4nsv	0.167	0.436	FALSE			FALSE	FALSE	FALSE	FALSE	
4mh4	0.899	0.151	FALSE			FALSE	FALSE	FALSE	FALSE	
4yya	0.204	0.055	FALSE			FALSE	FALSE	FALSE	FALSE	
4wv8	0.132	0.387	FALSE			FALSE	FALSE	FALSE	FALSE	
4yw6	0.000	0.000	TRUE	TRUE	TRUE	FALSE	FALSE	FALSE	FALSE	
4o75	0.895	0.982	FALSE			FALSE	FALSE	FALSE	FALSE	
4qym	0.612	0.370	FALSE			FALSE	FALSE	FALSE	FALSE	
4crh	0.835	0.946	FALSE			FALSE	FALSE	FALSE	FALSE	
4xf9	0.023	0.049	FALSE			FALSE	FALSE	FALSE	FALSE	
5a7s	0.383	0.307	FALSE	TRUE		FALSE	FALSE	FALSE	TRUE	
5fjs	0.960	0.651	FALSE			FALSE	FALSE	FALSE	FALSE	
5ces	0.000	0.352	TRUE			FALSE	TRUE	FALSE	FALSE	
5hf6	0.215	0.929	FALSE			FALSE	FALSE	FALSE	FALSE	
4ryl	0.000	0.010	TRUE	TRUE	TRUE	FALSE	FALSE	FALSE	FALSE	
5d0c	0.192	0.000	TRUE		TRUE	FALSE	FALSE	TRUE	FALSE	
4zvn	0.255	0.409	FALSE			FALSE	FALSE	FALSE	FALSE	
5elf	0.832	0.807	FALSE			FALSE	FALSE	FALSE	FALSE	
4z3m	0.821	0.391	FALSE			FALSE	FALSE	FALSE	FALSE	
5c8x	0.530	0.565	FALSE			FALSE	FALSE	FALSE	FALSE	
5e5t	0.787	0.158	FALSE			FALSE	FALSE	FALSE	FALSE	
5faq	0.000	0.136	TRUE	TRUE	TRUE	FALSE	FALSE	FALSE	FALSE	
1i0v	0.820	0.022	FALSE			FALSE	FALSE	FALSE	FALSE	
2xds	0.376	0.997	FALSE			FALSE	FALSE	FALSE	FALSE	
3ap3	0.939	1.000	FALSE			FALSE	FALSE	FALSE	FALSE	
3fd5	0.889	0.915	FALSE			FALSE	FALSE	FALSE	FALSE	
3ikt	0.000	0.933	TRUE	TRUE	TRUE	FALSE	FALSE	FALSE	FALSE	
3zxh	0.000	0.786	TRUE	TRUE	TRUE	FALSE	FALSE	FALSE	FALSE	
3zzs	0.075	0.383	FALSE	TRUE		FALSE	FALSE	FALSE	TRUE	
4aw9	1.000	0.000	TRUE		TRUE	FALSE	FALSE	TRUE	FALSE	
4awa	0.138	0.432	FALSE			FALSE	FALSE	FALSE	FALSE	
4g1n	0.017	0.176	FALSE			FALSE	FALSE	FALSE	FALSE	
4kwc	0.125	1.000	FALSE			FALSE	FALSE	FALSE	FALSE	
4n9r	0.010	0.893	FALSE		TRUE	TRUE	FALSE	TRUE	FALSE	
4oq5	0.586	0.863	FALSE			FALSE	FALSE	FALSE	FALSE	
4ut0	0.619	0.675	FALSE			FALSE	FALSE	FALSE	FALSE	
		Discoveries	39	38	37	6.00	8.00	15.00	14.00	T
						16.7	4.9	41.7	8.6	F

1: Denotes entries where visual classification differed

2: Denotes entries with resolution less than 3.661 Å making ice undetectable.

Table S5 Confusion matrix for our analysis of the 198 PDB entries in Table S4.

		Visual Classification	
		No Ice	Ice
Predicted Classification	No Ice	153	6
	Ice	8	31

Table S6

	AUSPEX Test Set		Ground Truth Test Set	
	False Negatives	False Positives	False Negatives	False Positives
Combined metric: p_{ice}	6	8	7	3
IFS	9	4	9	4
DS	10	5	10	5
Observations: p_n	30	3	25	1
