# Supplementary figures and images for: Ice in biomolecular cryocrystallography (part 2 of 2)
Source: Acta Crystallogr D Struct Biol. 2021 Mar 30;77(Pt 4):540–54. doi: 10.1107/S2059798321001170 (PMC8025888; doi:10.1107/S2059798321001170)

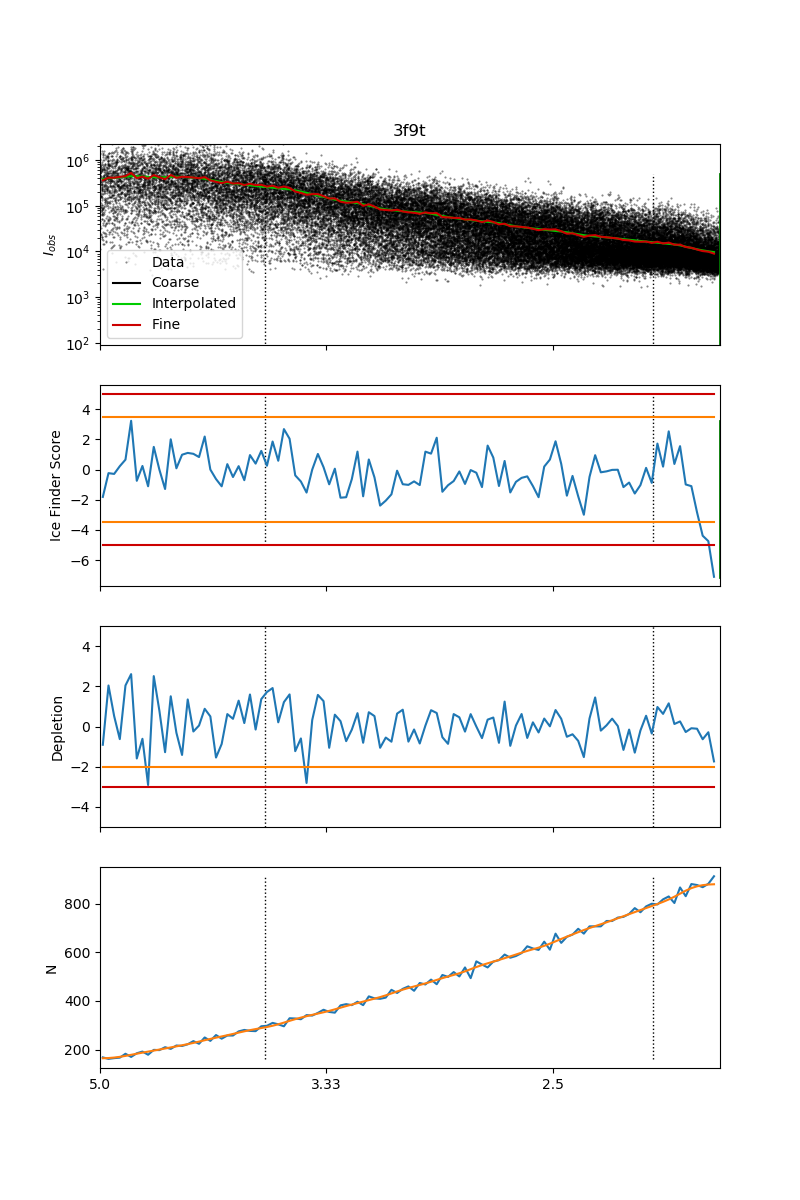

Supplement: Supplementary file 3 [file d-77-00540-sup3.zip › IceBiasingImages/3f9t.png]

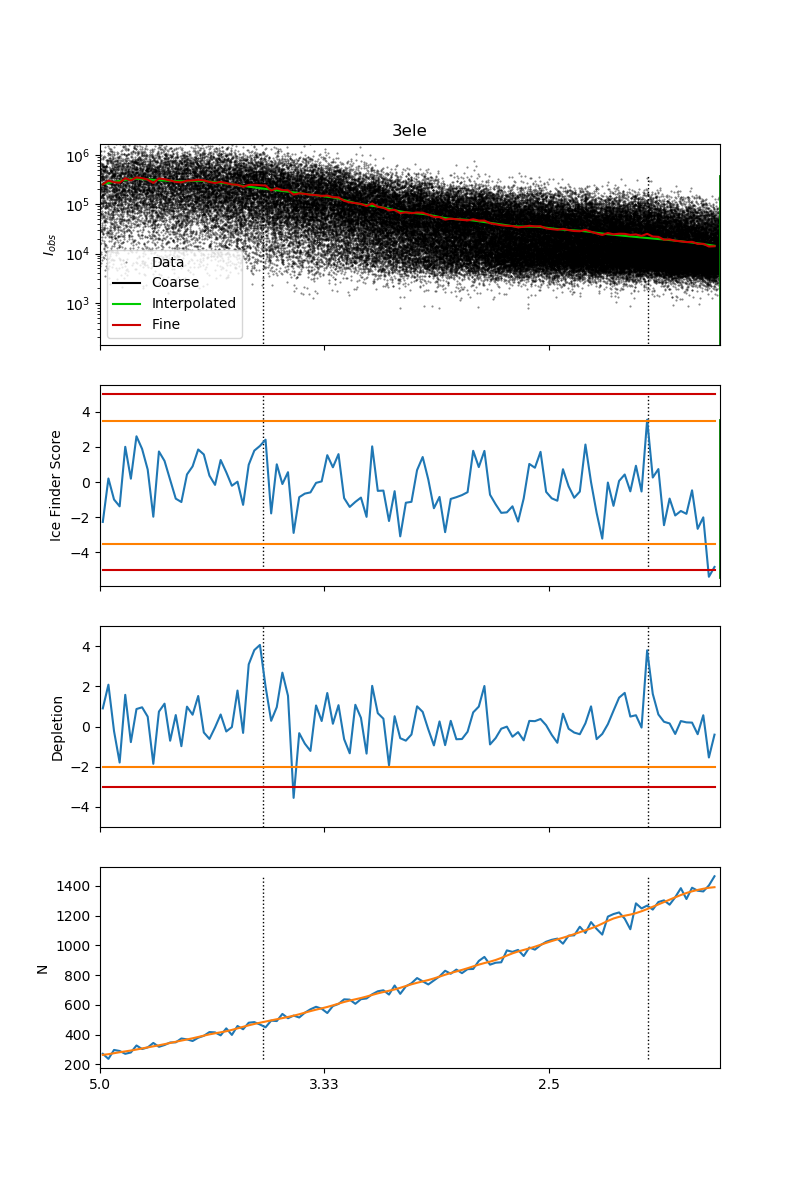

Supplement: Supplementary file 3 [file d-77-00540-sup3.zip › IceBiasingImages/3ele.png]

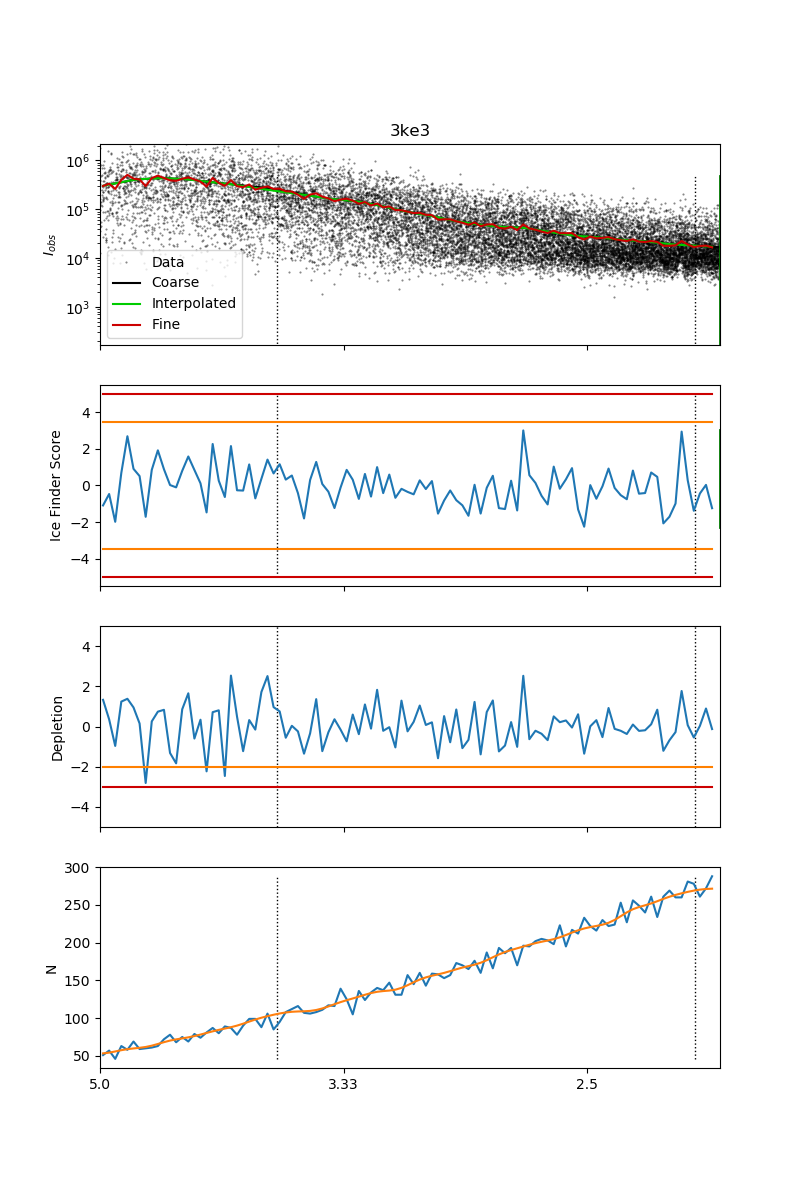

Supplement: Supplementary file 3 [file d-77-00540-sup3.zip › IceBiasingImages/3ke3.png]

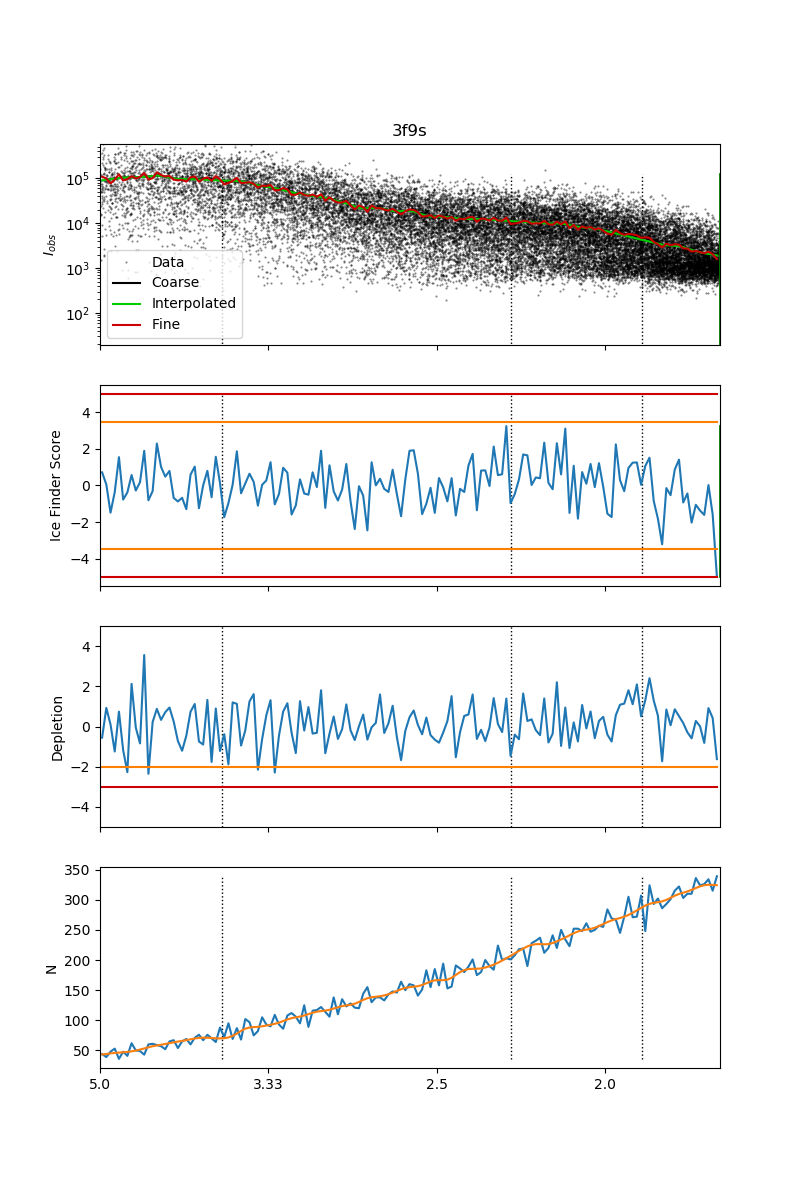

Supplement: Supplementary file 3 [file d-77-00540-sup3.zip › IceBiasingImages/3f9s.png]

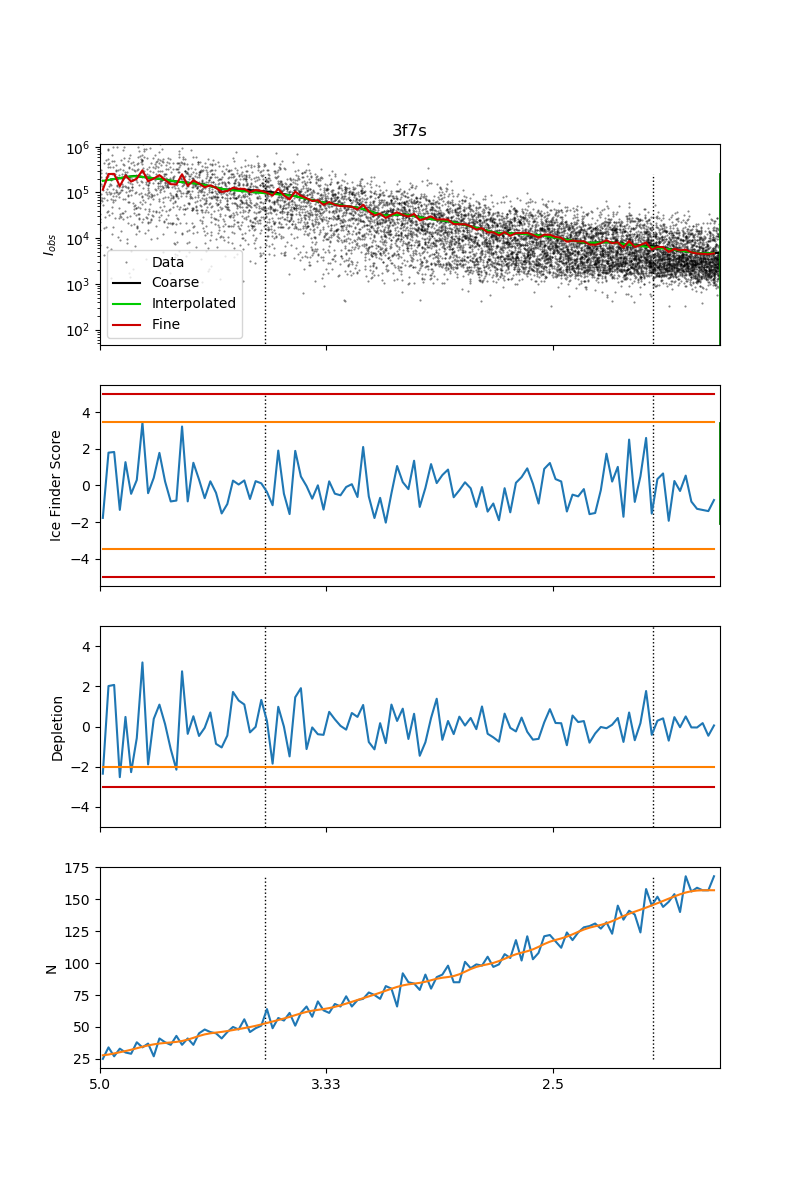

Supplement: Supplementary file 3 [file d-77-00540-sup3.zip › IceBiasingImages/3f7s.png]

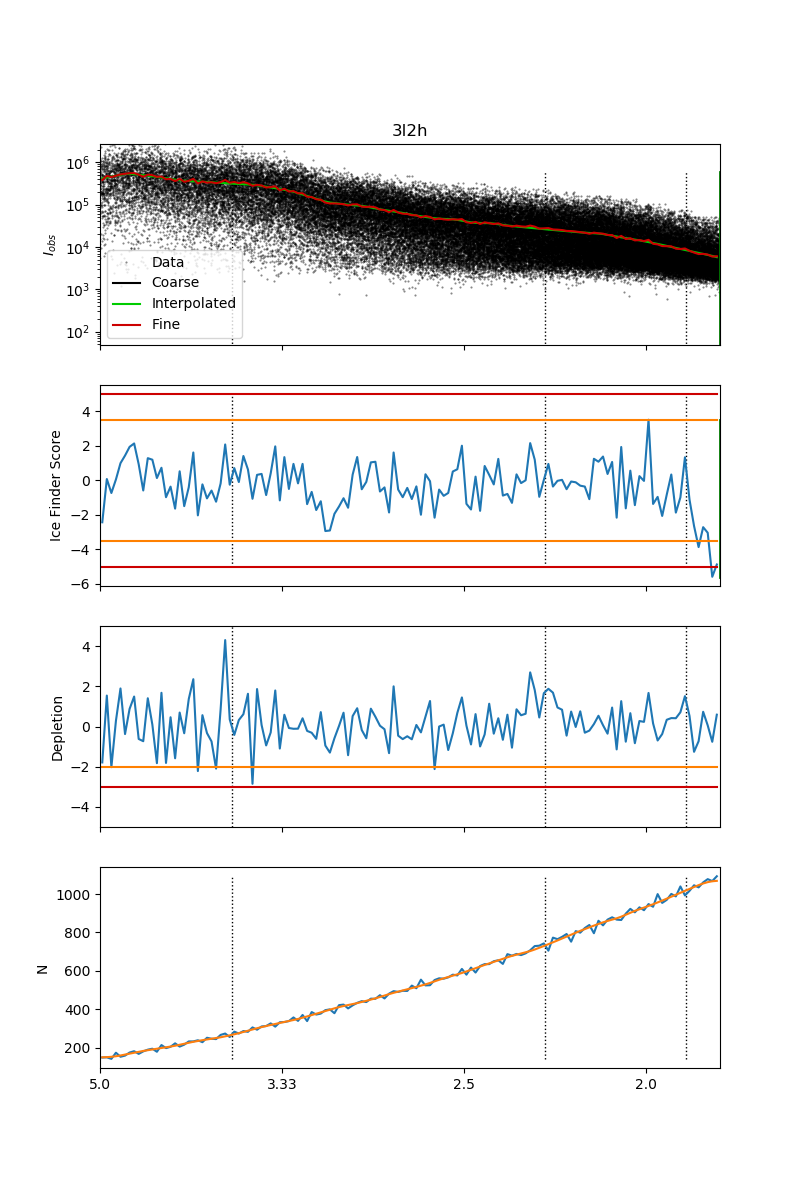

Supplement: Supplementary file 3 [file d-77-00540-sup3.zip › IceBiasingImages/3l2h.png]

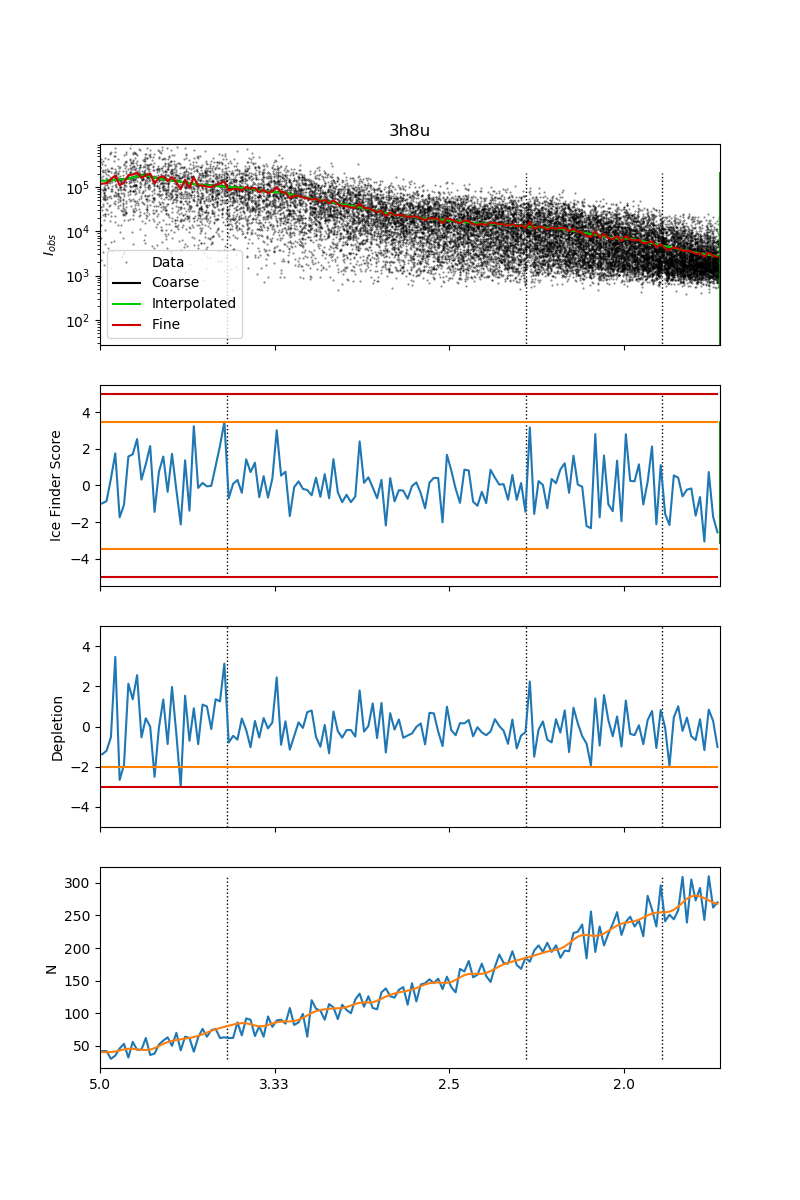

Supplement: Supplementary file 3 [file d-77-00540-sup3.zip › IceBiasingImages/3h8u.png]

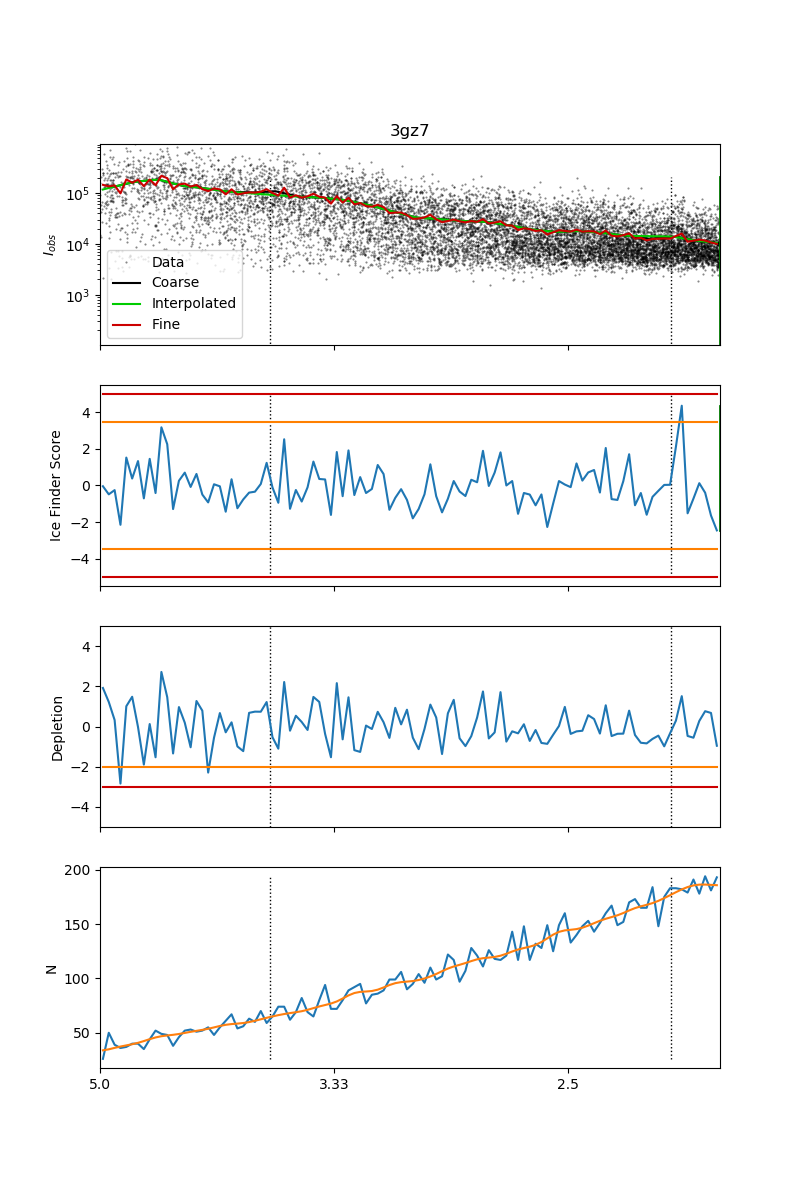

Supplement: Supplementary file 3 [file d-77-00540-sup3.zip › IceBiasingImages/3gz7.png]

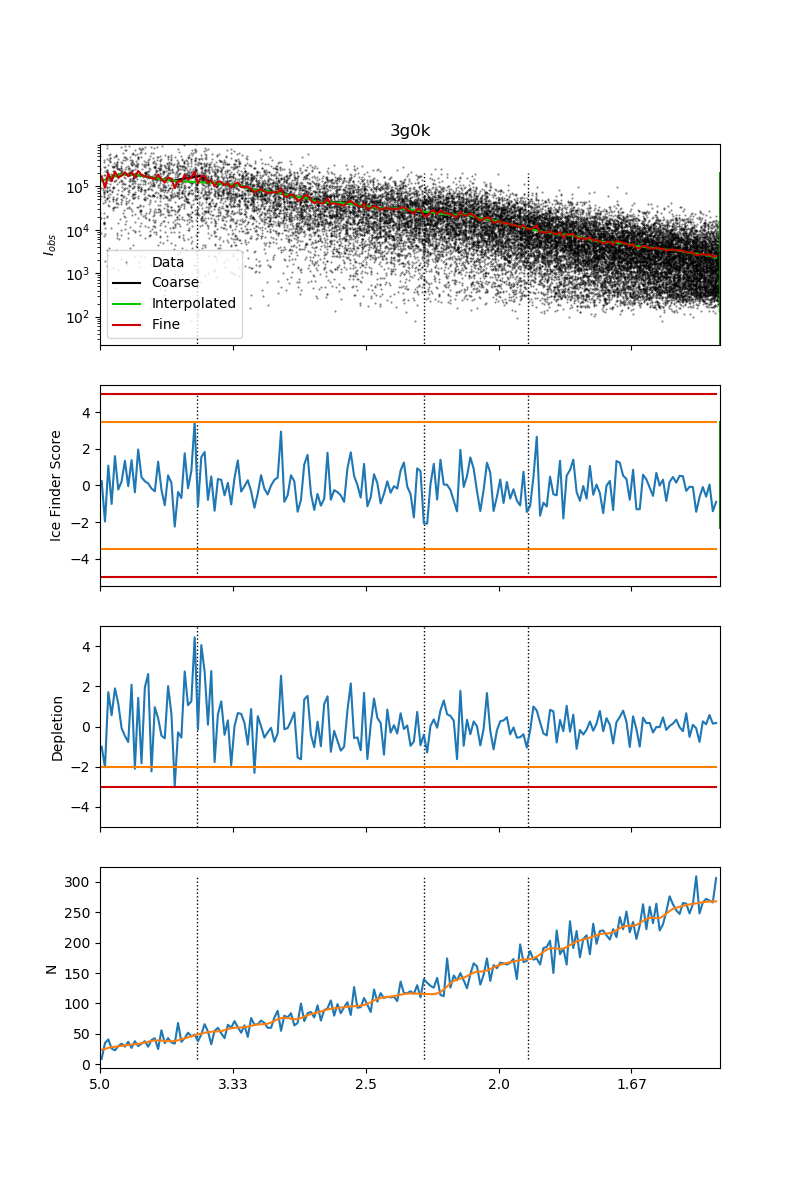

Supplement: Supplementary file 3 [file d-77-00540-sup3.zip › IceBiasingImages/3g0k.png]

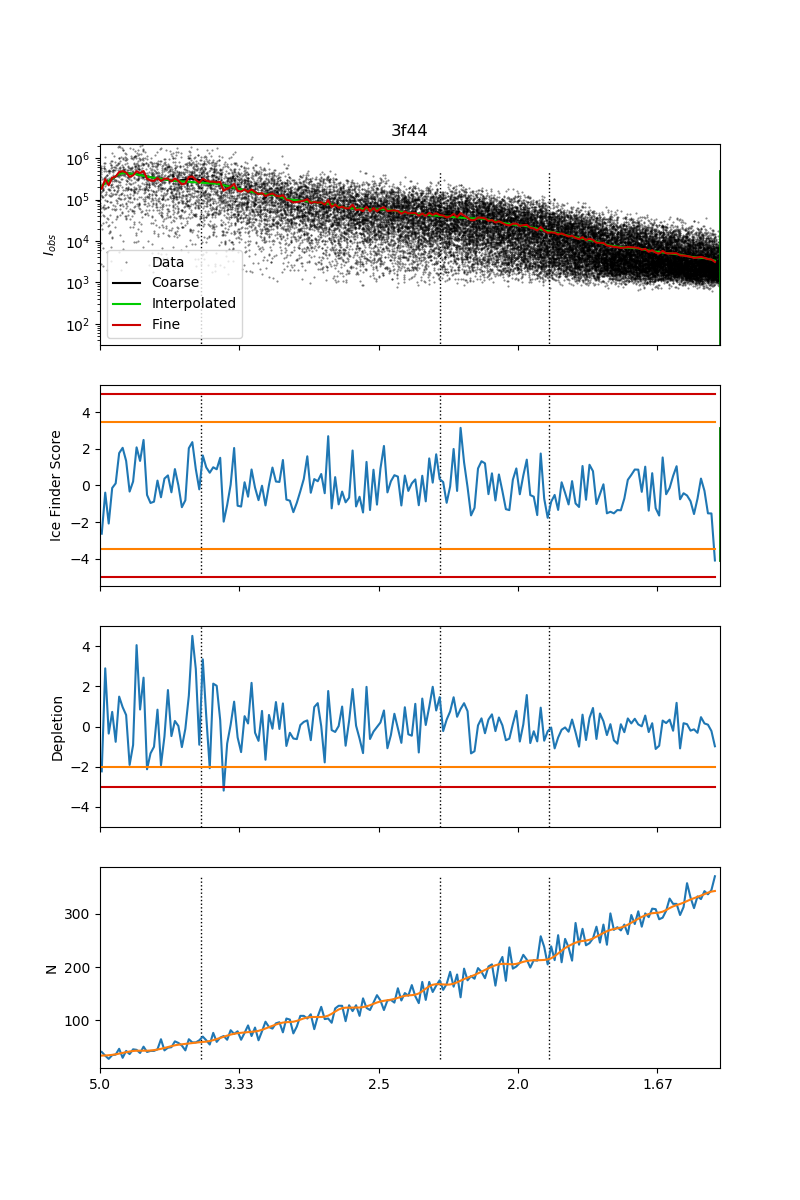

Supplement: Supplementary file 3 [file d-77-00540-sup3.zip › IceBiasingImages/3f44.png]

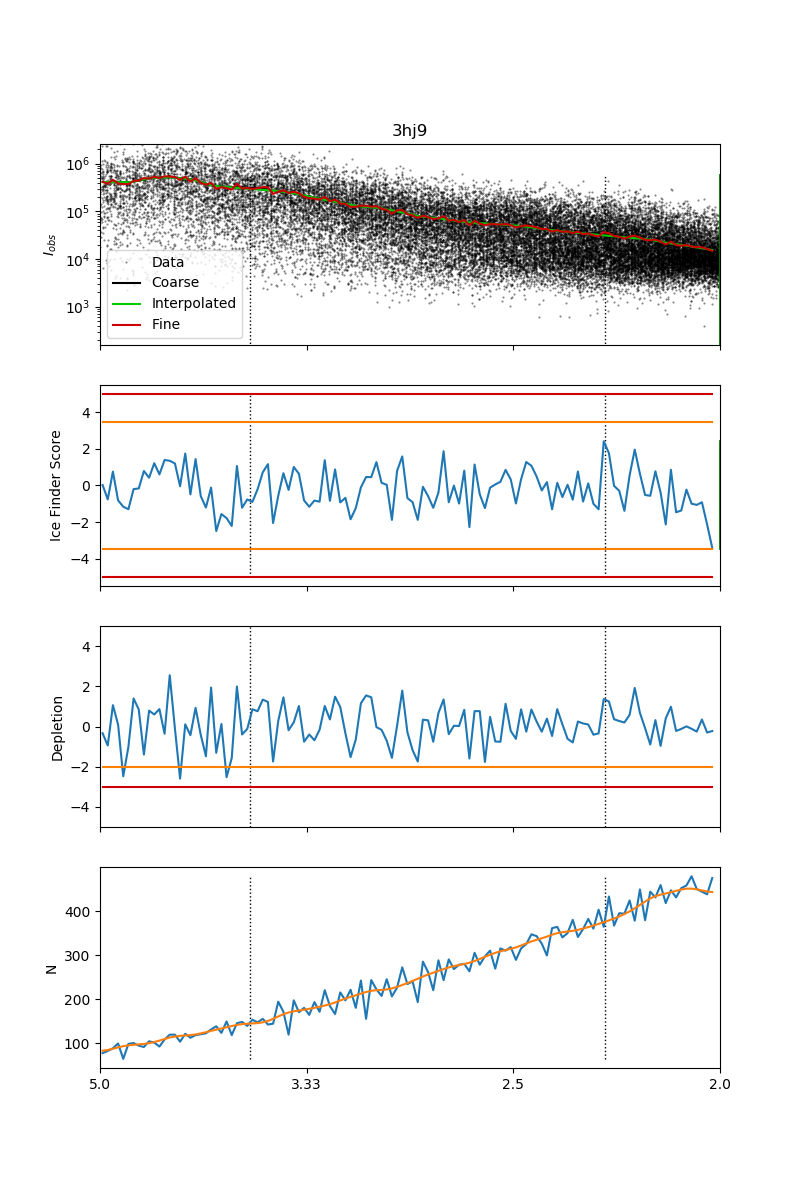

Supplement: Supplementary file 3 [file d-77-00540-sup3.zip › IceBiasingImages/3hj9.png]

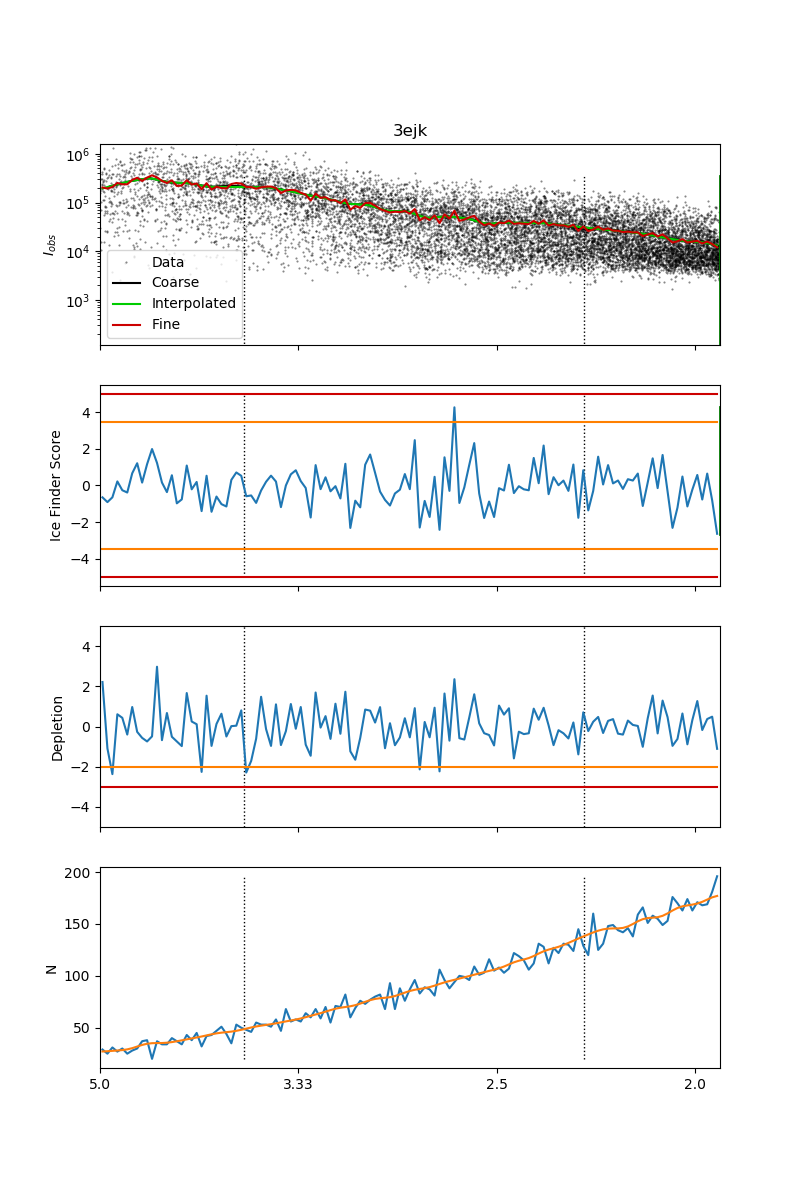

Supplement: Supplementary file 3 [file d-77-00540-sup3.zip › IceBiasingImages/3ejk.png]

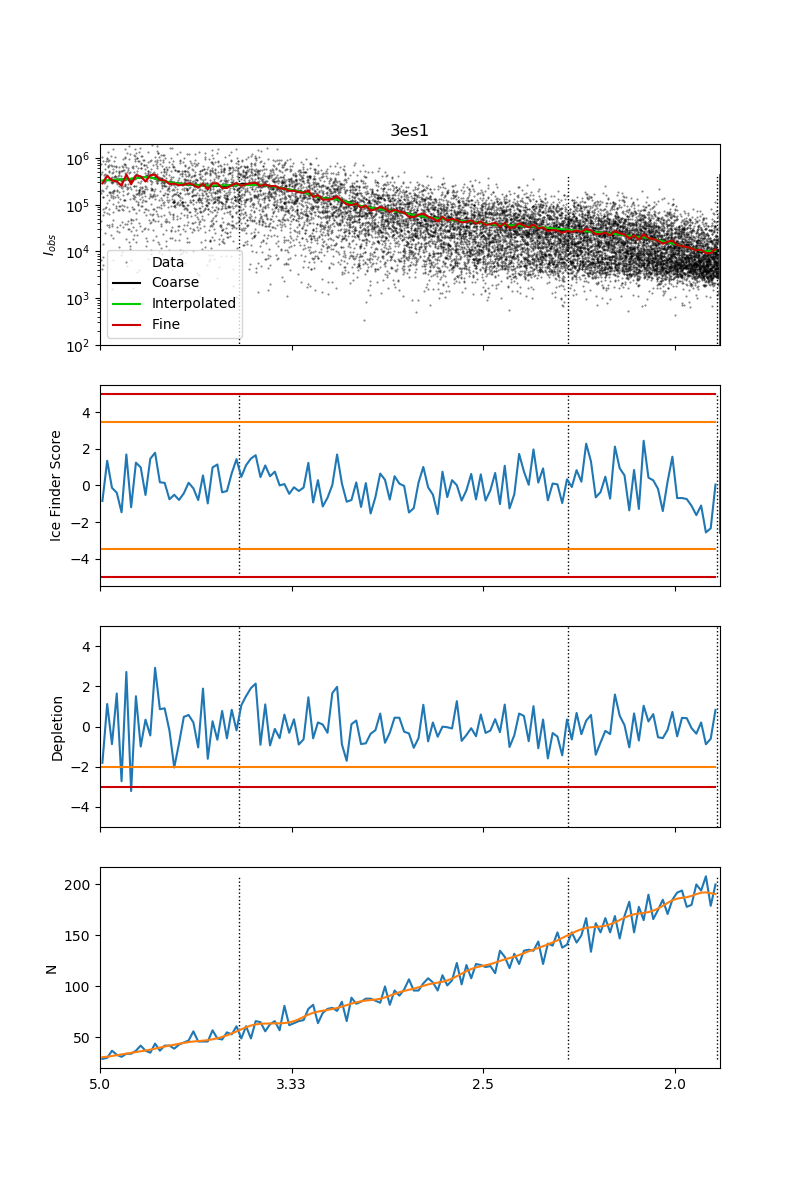

Supplement: Supplementary file 3 [file d-77-00540-sup3.zip › IceBiasingImages/3es1.png]

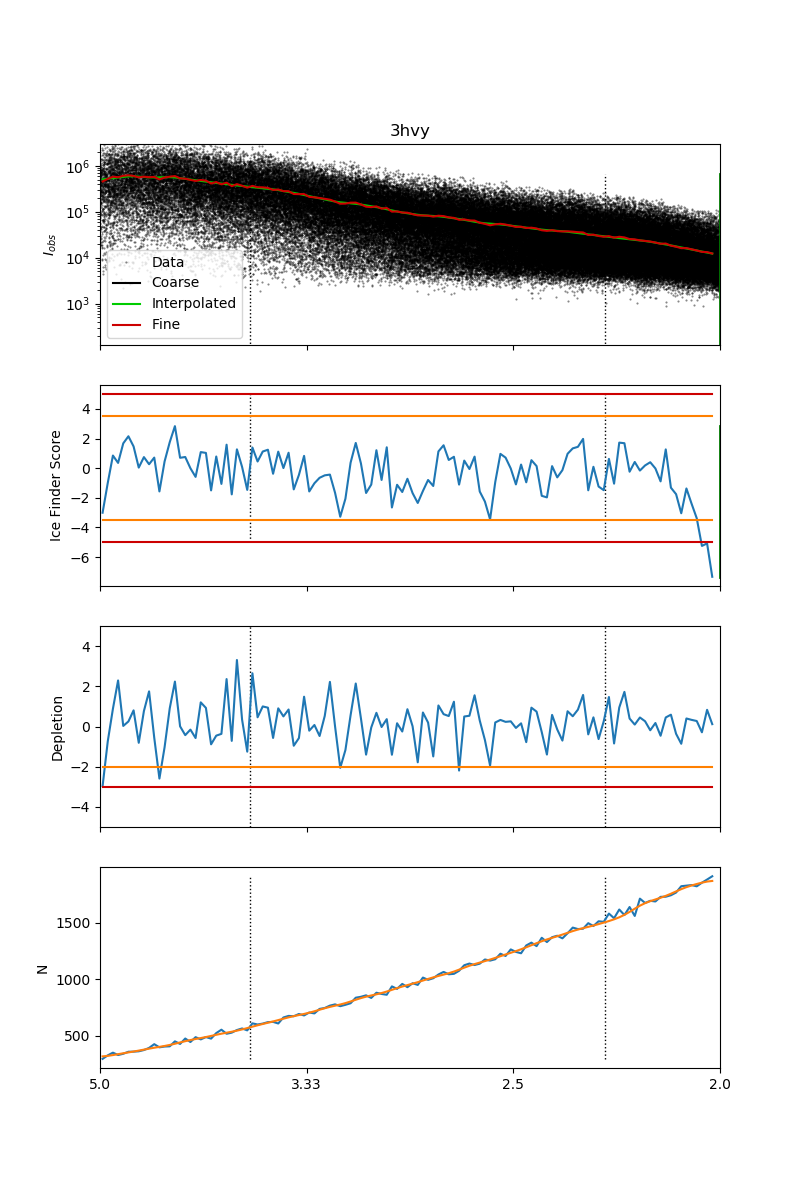

Supplement: Supplementary file 3 [file d-77-00540-sup3.zip › IceBiasingImages/3hvy.png]

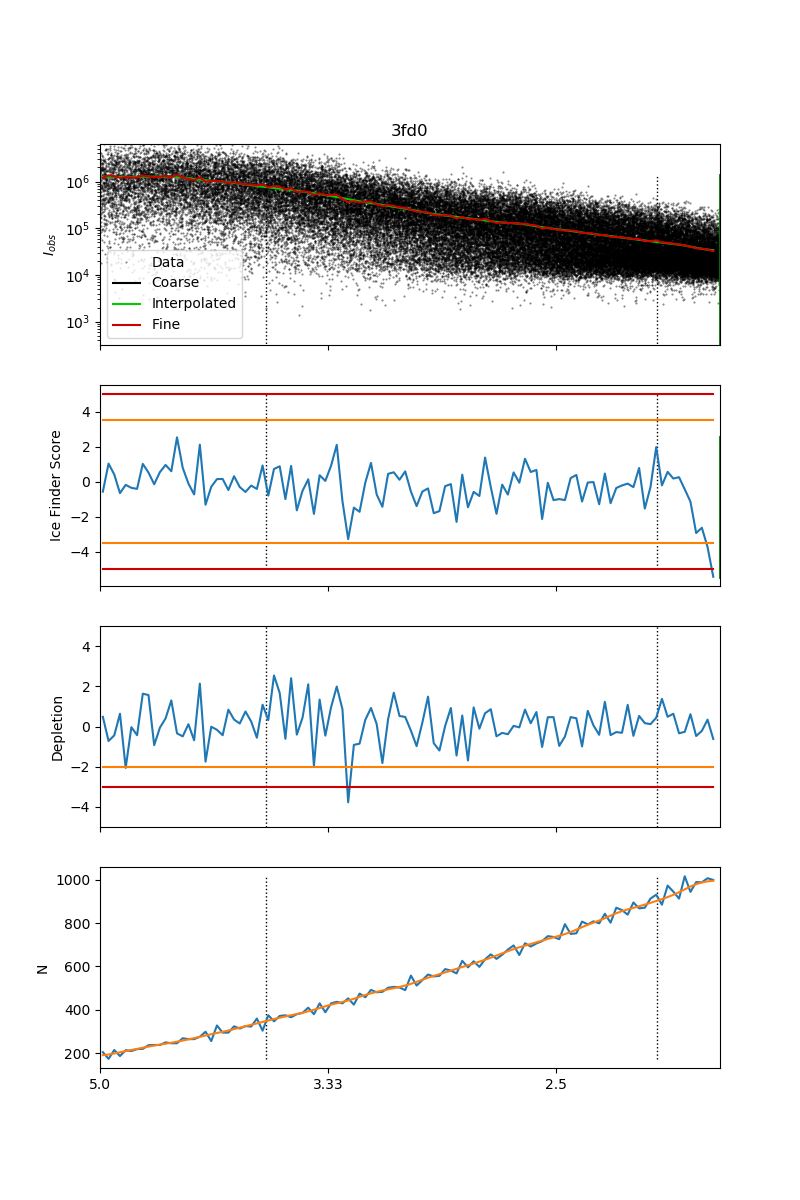

Supplement: Supplementary file 3 [file d-77-00540-sup3.zip › IceBiasingImages/3fd0.png]

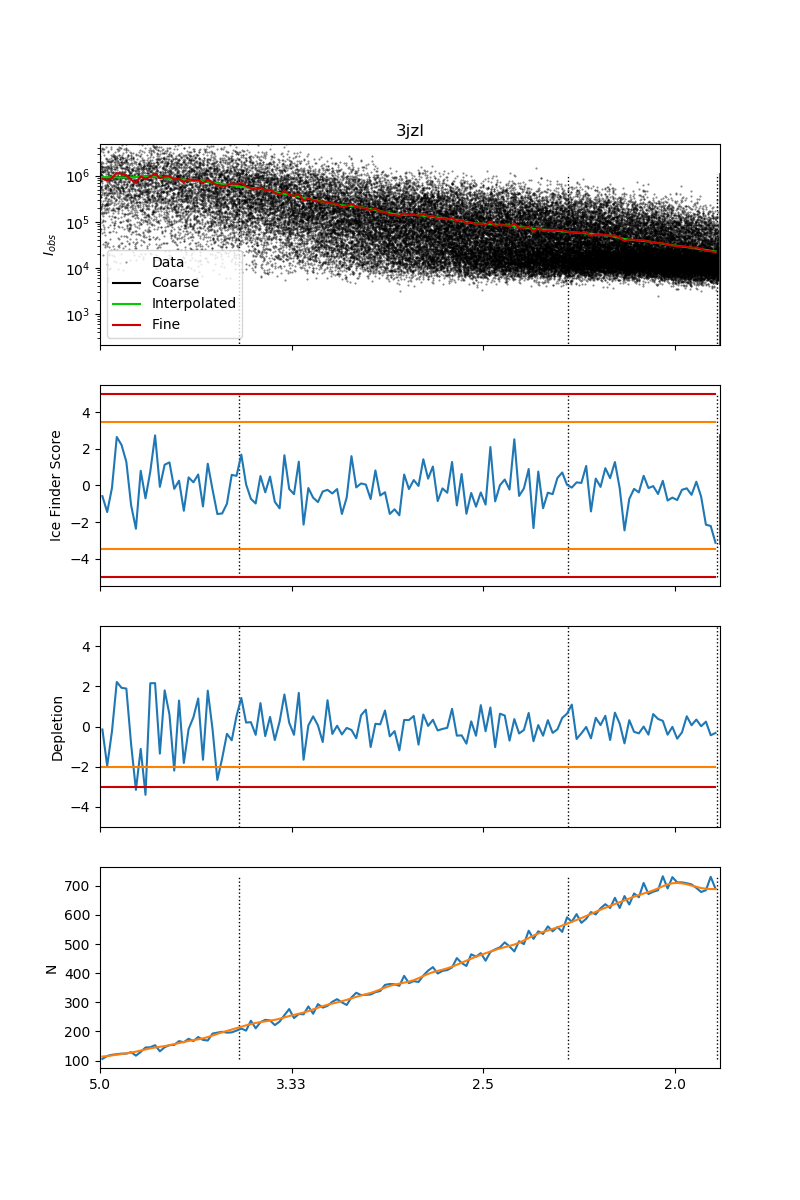

Supplement: Supplementary file 3 [file d-77-00540-sup3.zip › IceBiasingImages/3jzl.png]

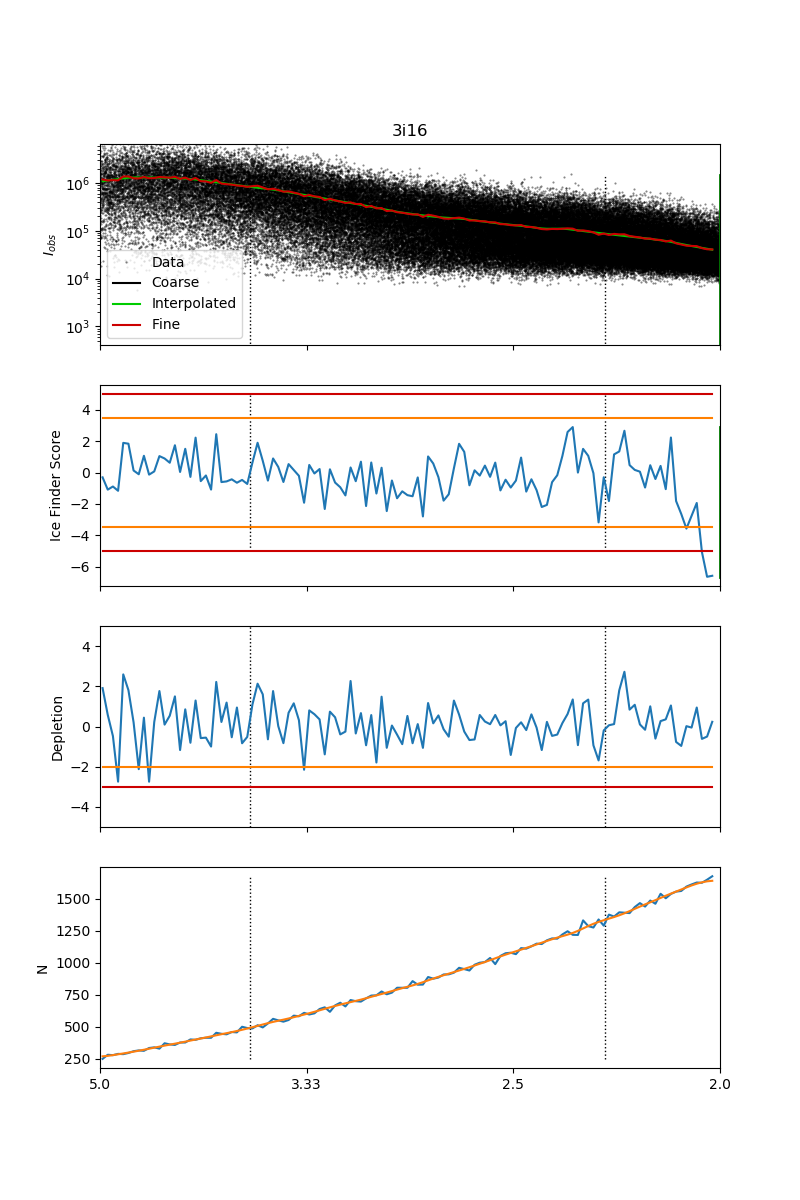

Supplement: Supplementary file 3 [file d-77-00540-sup3.zip › IceBiasingImages/3i16.png]

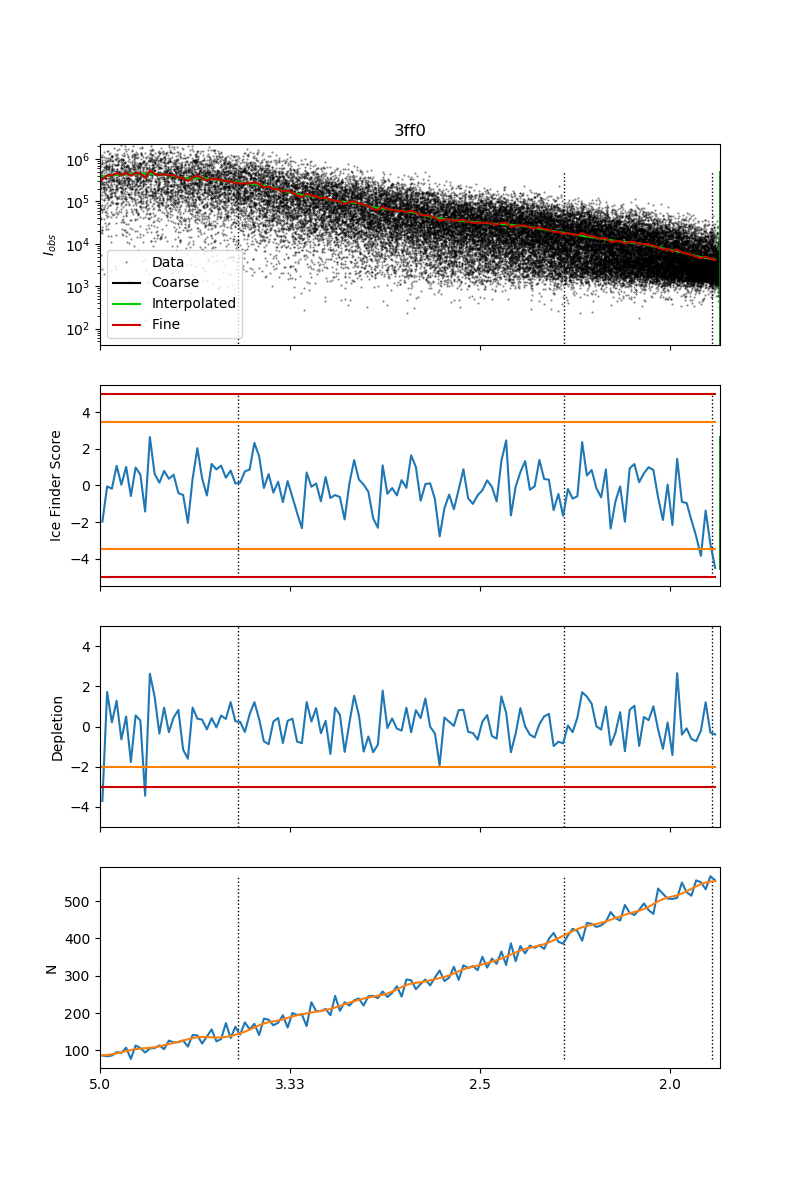

Supplement: Supplementary file 3 [file d-77-00540-sup3.zip › IceBiasingImages/3ff0.png]

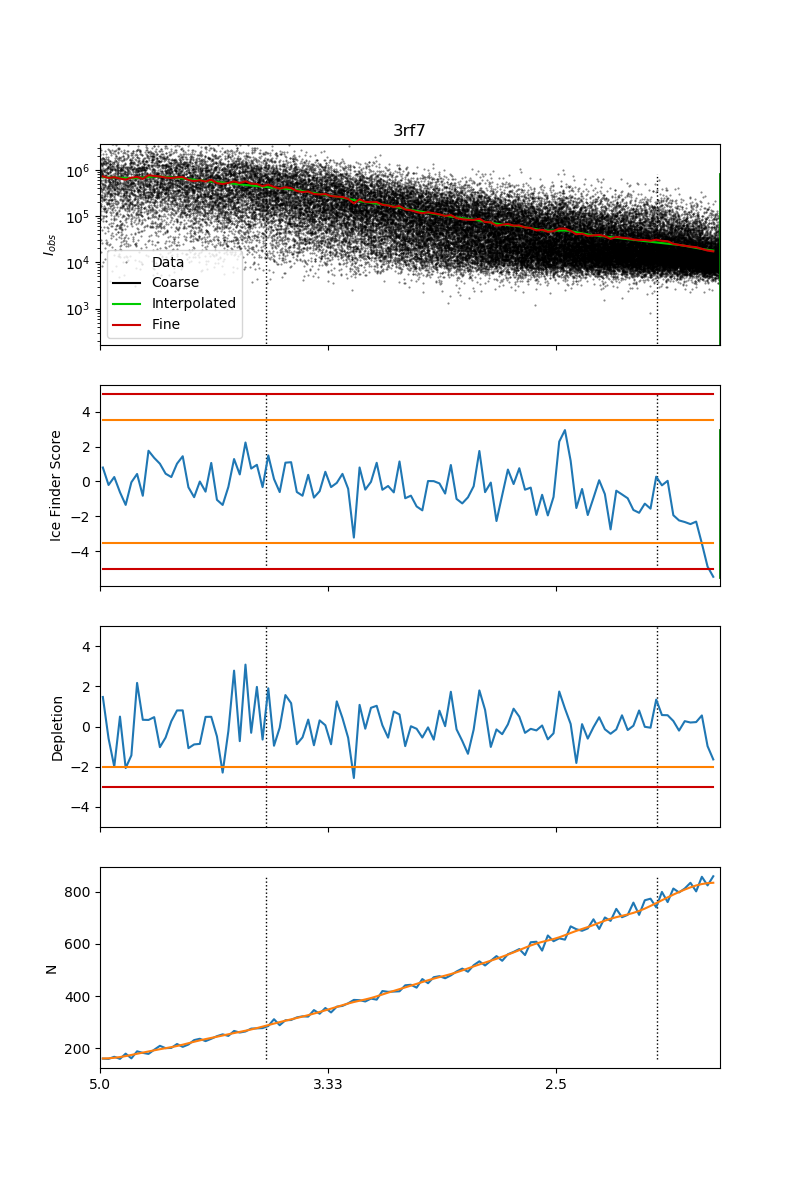

Supplement: Supplementary file 3 [file d-77-00540-sup3.zip › IceBiasingImages/3rf7.png]

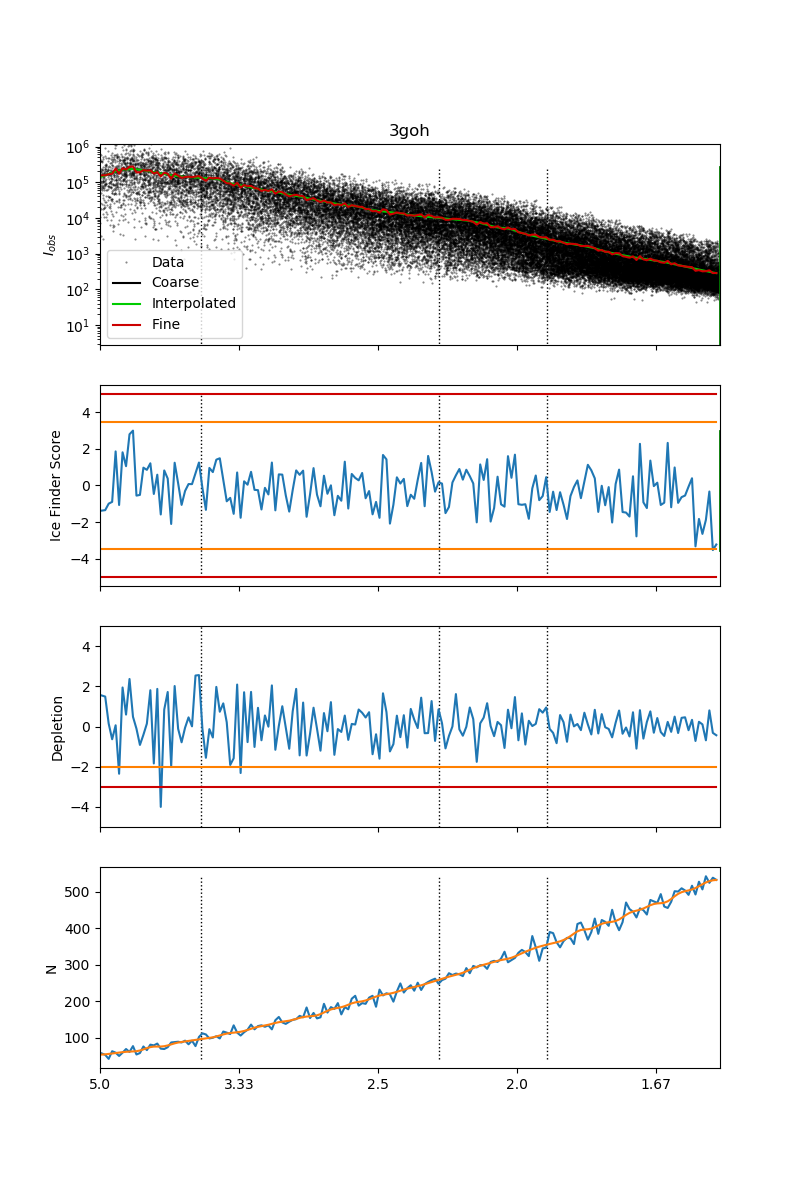

Supplement: Supplementary file 3 [file d-77-00540-sup3.zip › IceBiasingImages/3goh.png]

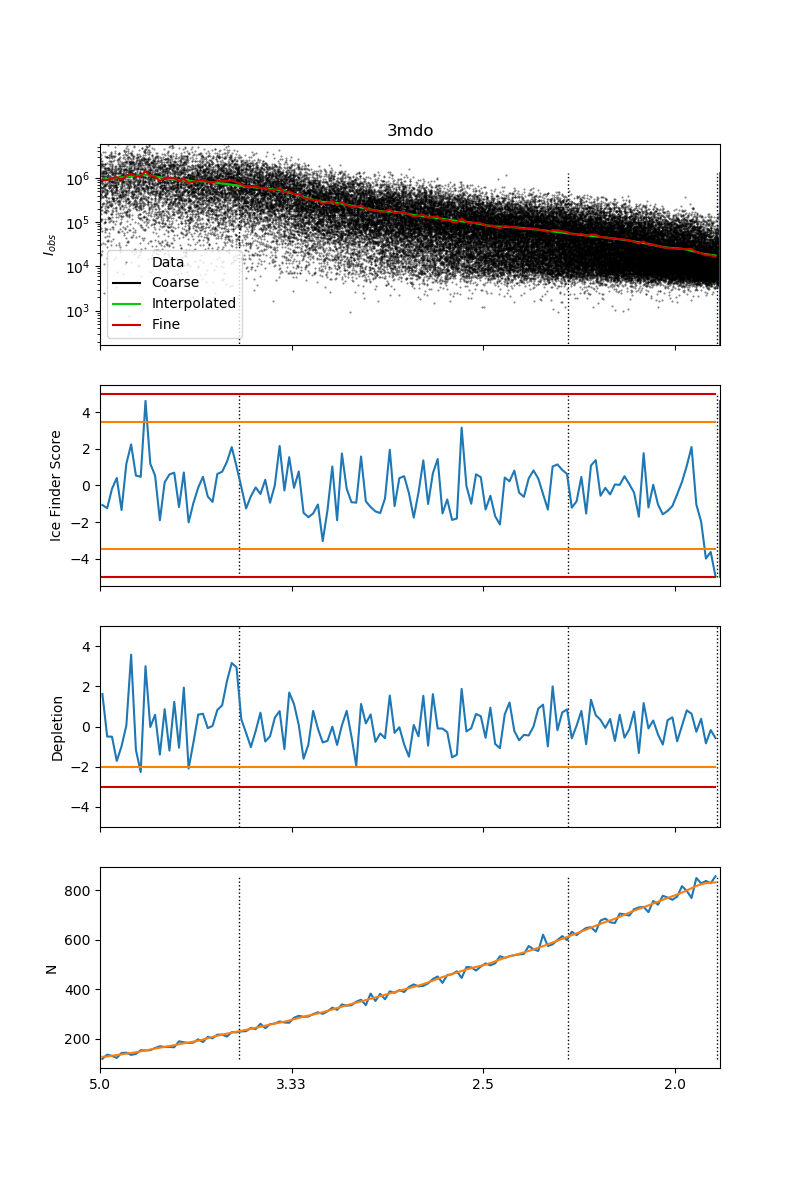

Supplement: Supplementary file 3 [file d-77-00540-sup3.zip › IceBiasingImages/3mdo.png]

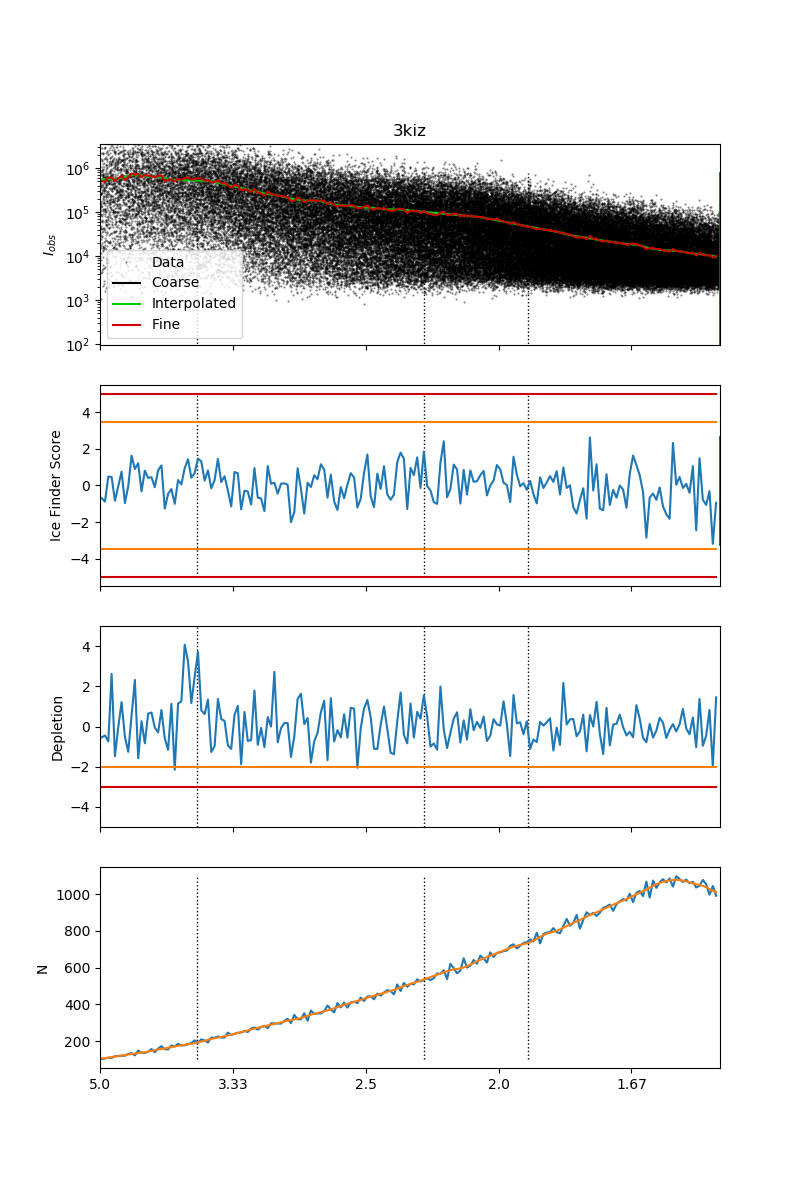

Supplement: Supplementary file 3 [file d-77-00540-sup3.zip › IceBiasingImages/3kiz.png]

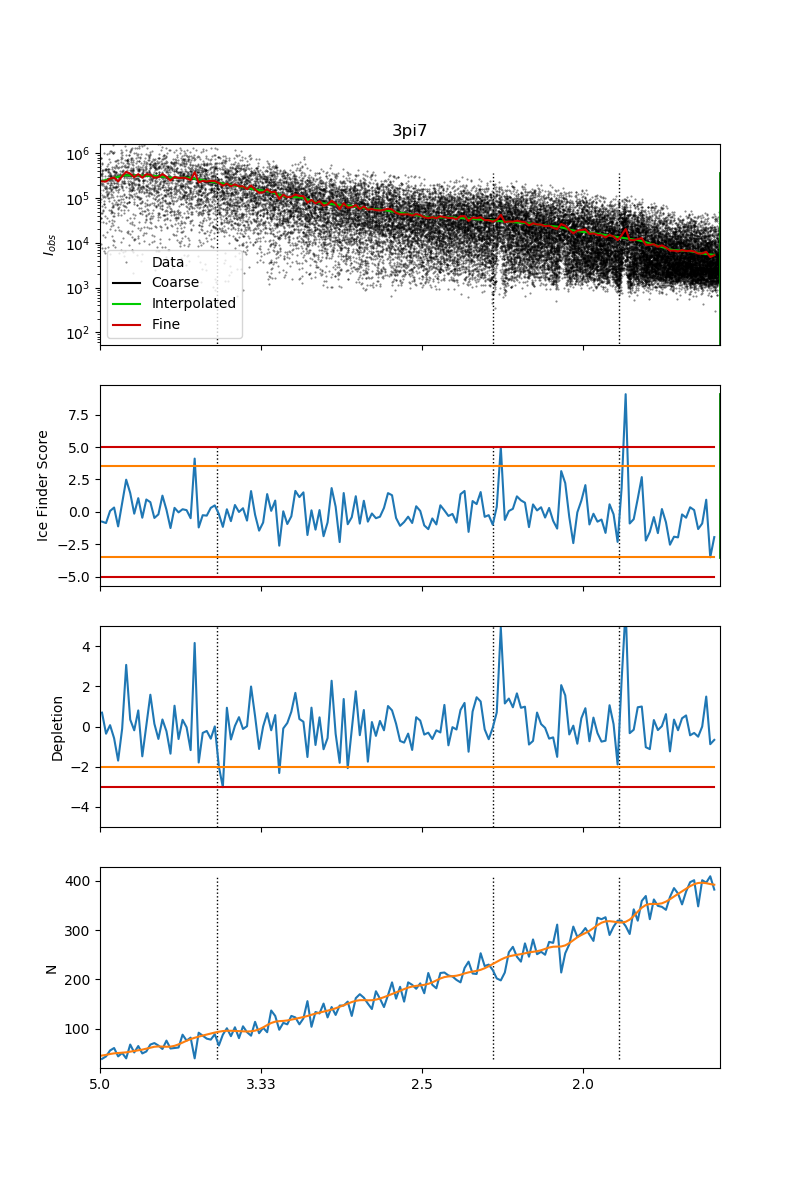

Supplement: Supplementary file 3 [file d-77-00540-sup3.zip › IceBiasingImages/3pi7.png]

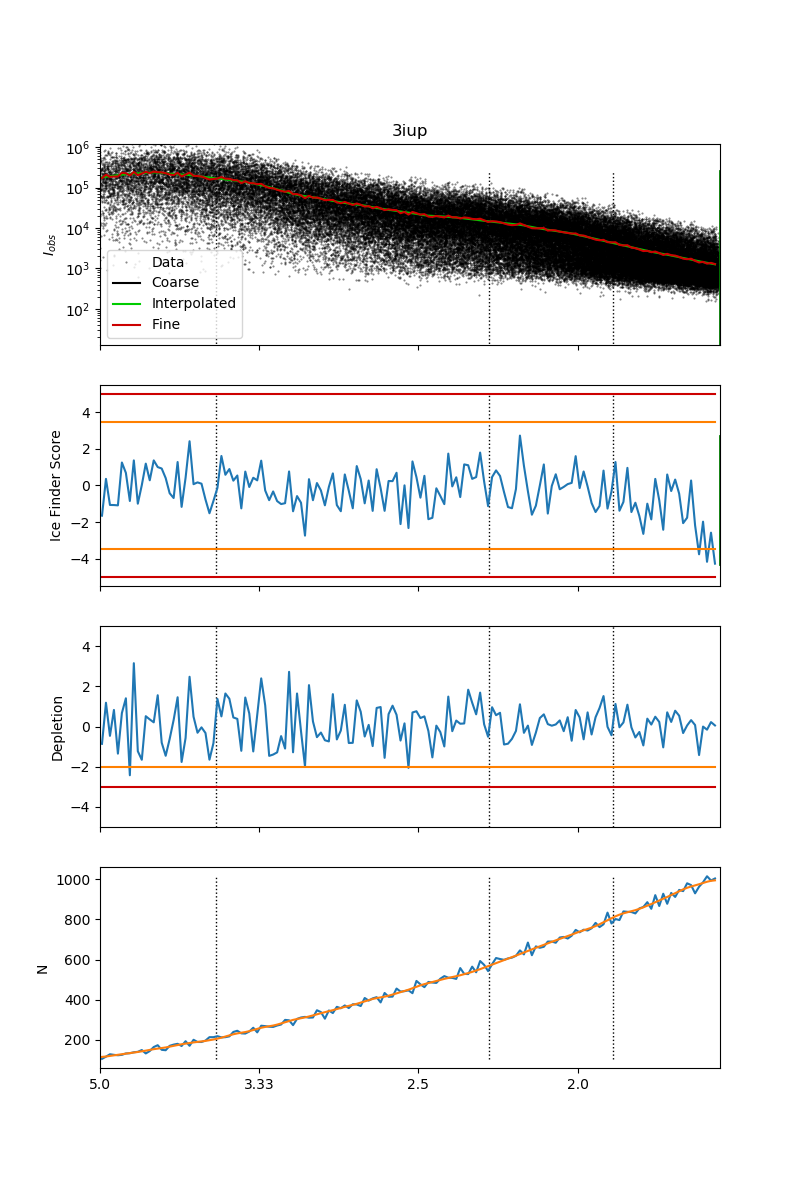

Supplement: Supplementary file 3 [file d-77-00540-sup3.zip › IceBiasingImages/3iup.png]

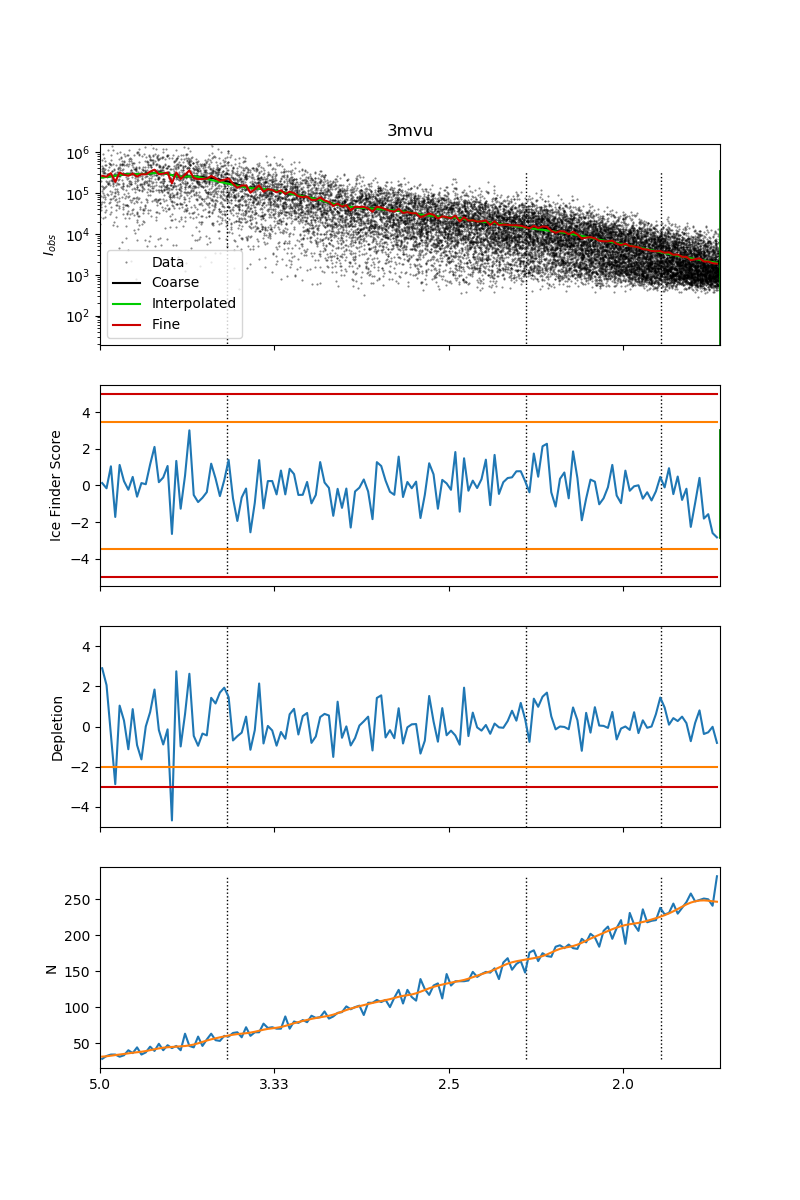

Supplement: Supplementary file 3 [file d-77-00540-sup3.zip › IceBiasingImages/3mvu.png]

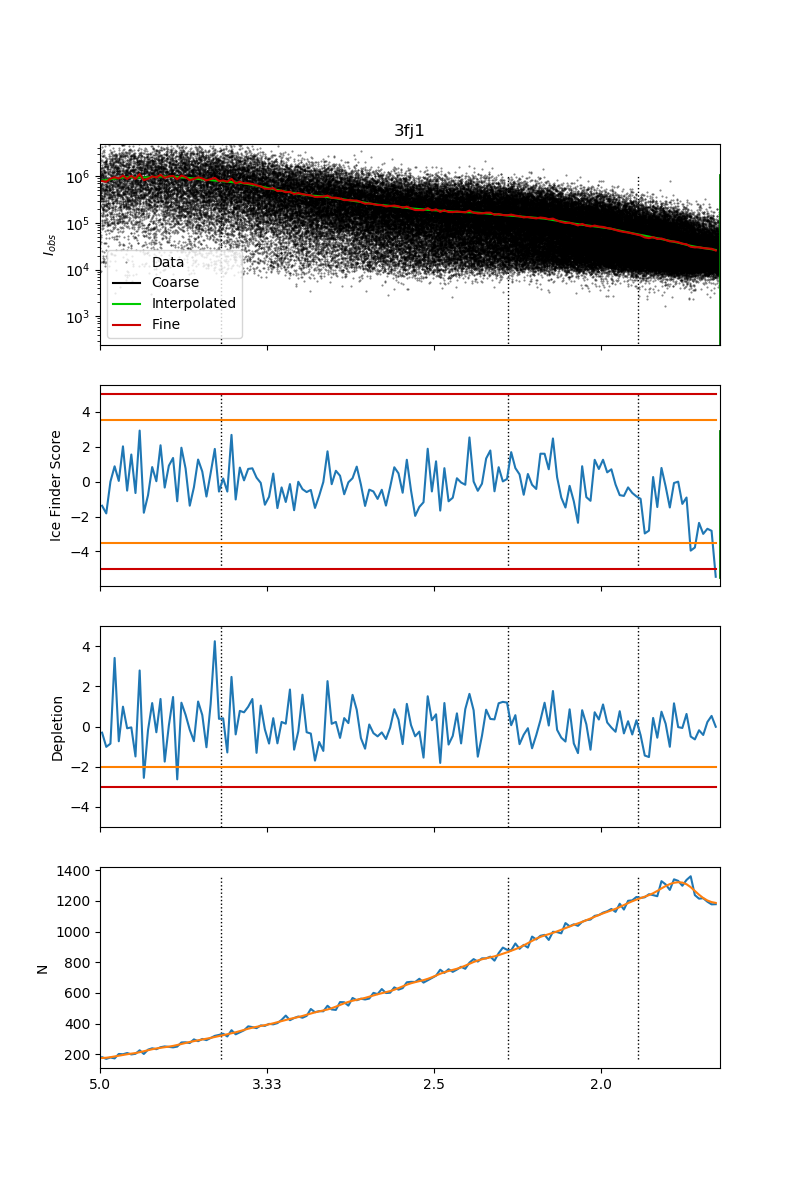

Supplement: Supplementary file 3 [file d-77-00540-sup3.zip › IceBiasingImages/3fj1.png]

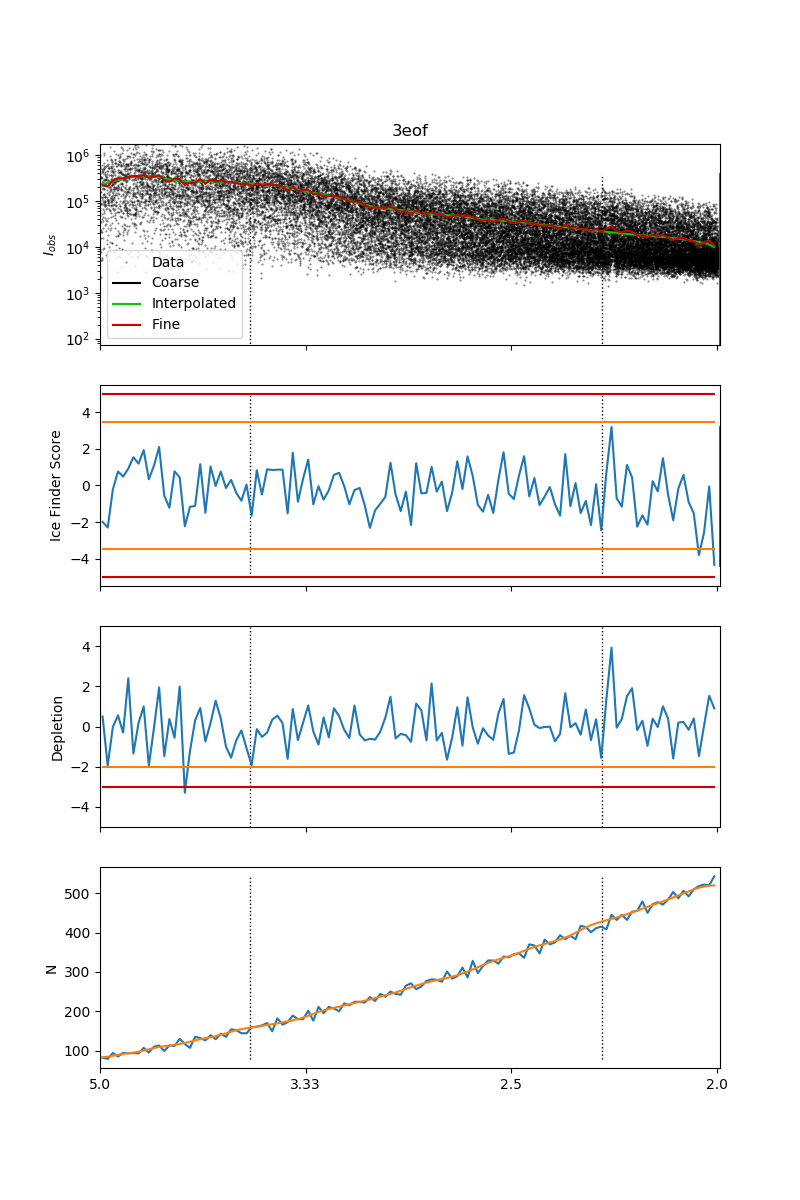

Supplement: Supplementary file 3 [file d-77-00540-sup3.zip › IceBiasingImages/3eof.png]

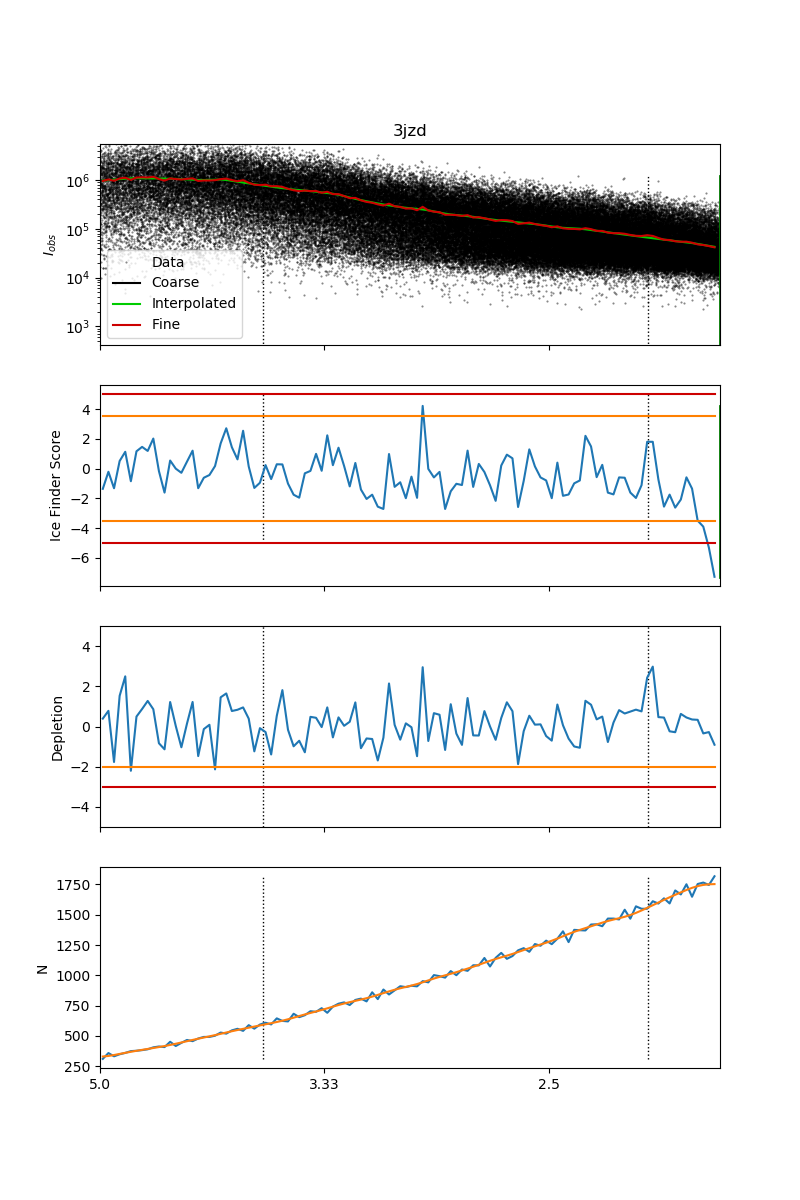

Supplement: Supplementary file 3 [file d-77-00540-sup3.zip › IceBiasingImages/3jzd.png]

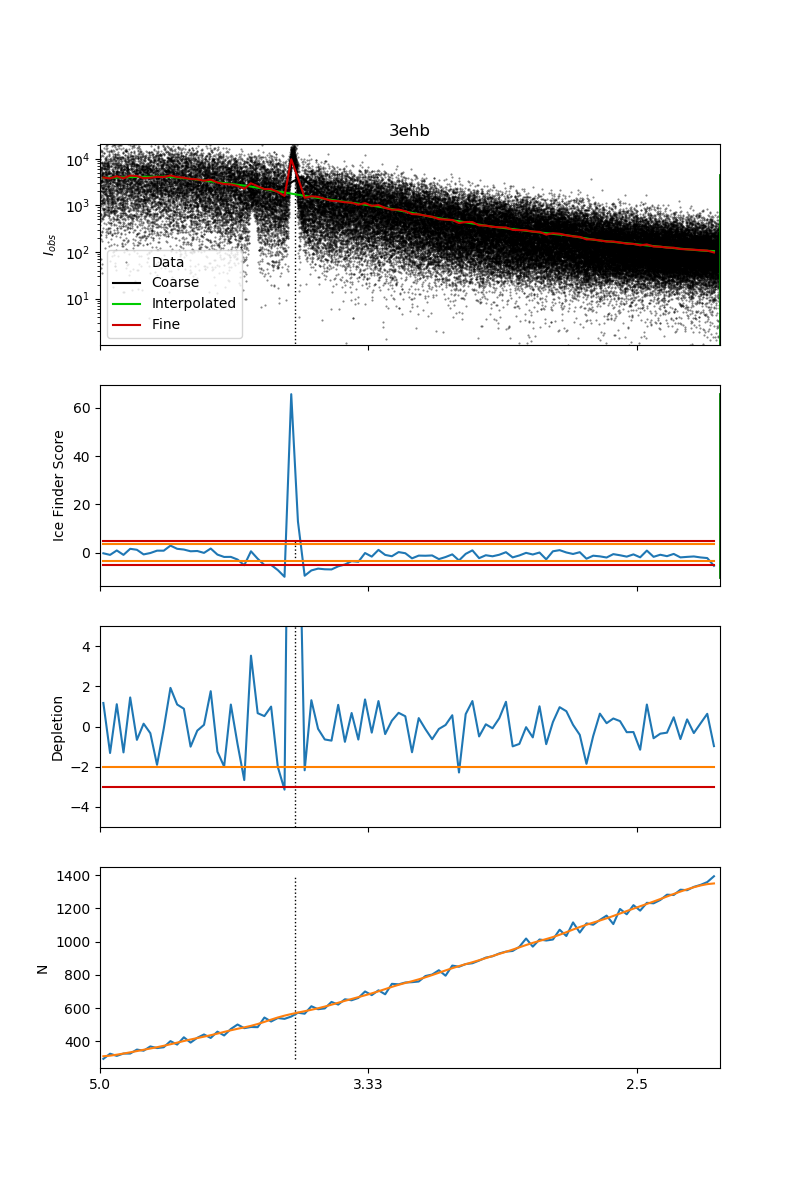

Supplement: Supplementary file 3 [file d-77-00540-sup3.zip › IceBiasingImages/3ehb.png]

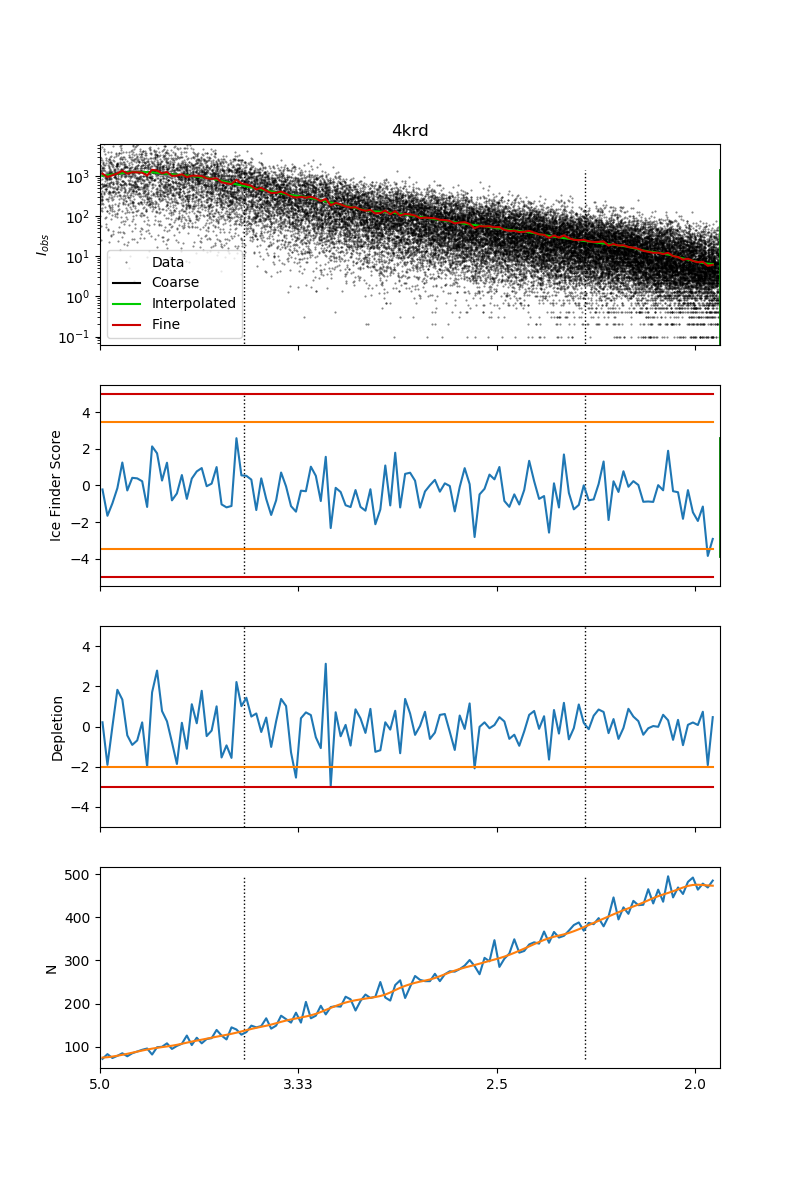

Supplement: Supplementary file 3 [file d-77-00540-sup3.zip › IceBiasingImages/4krd.png]

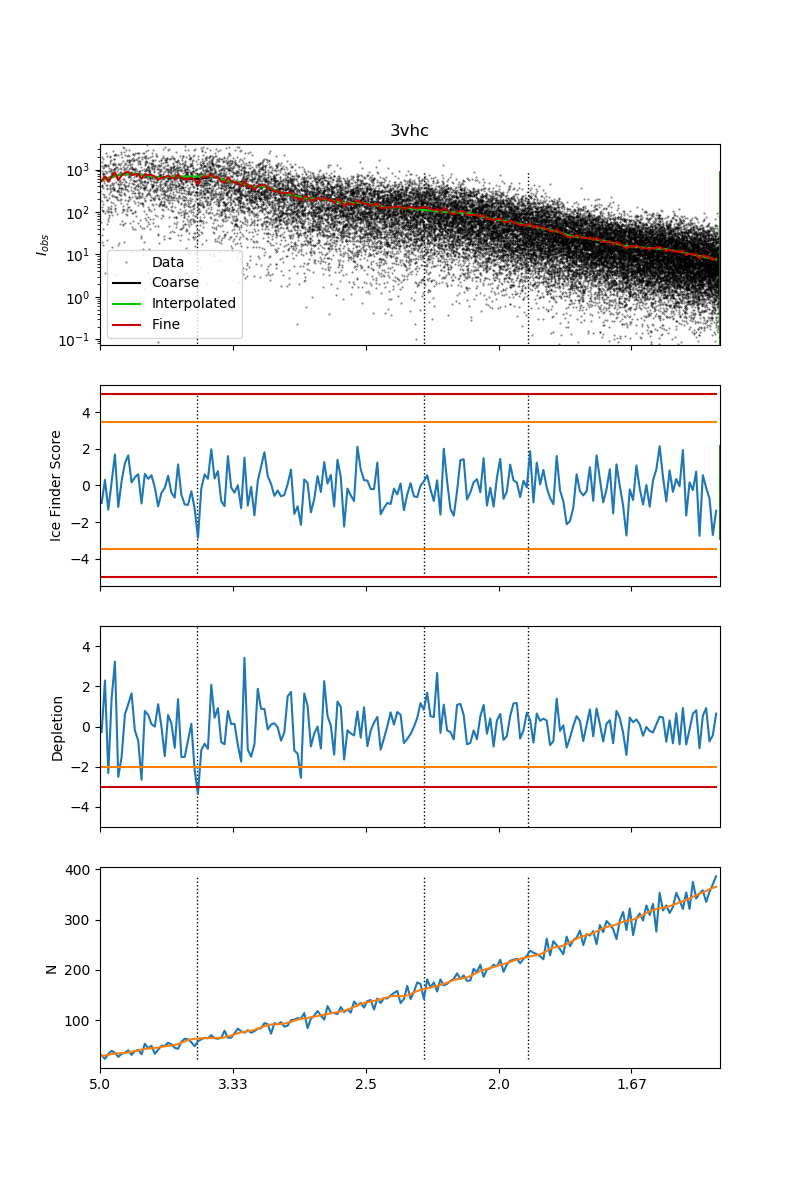

Supplement: Supplementary file 3 [file d-77-00540-sup3.zip › IceBiasingImages/3vhc.png]

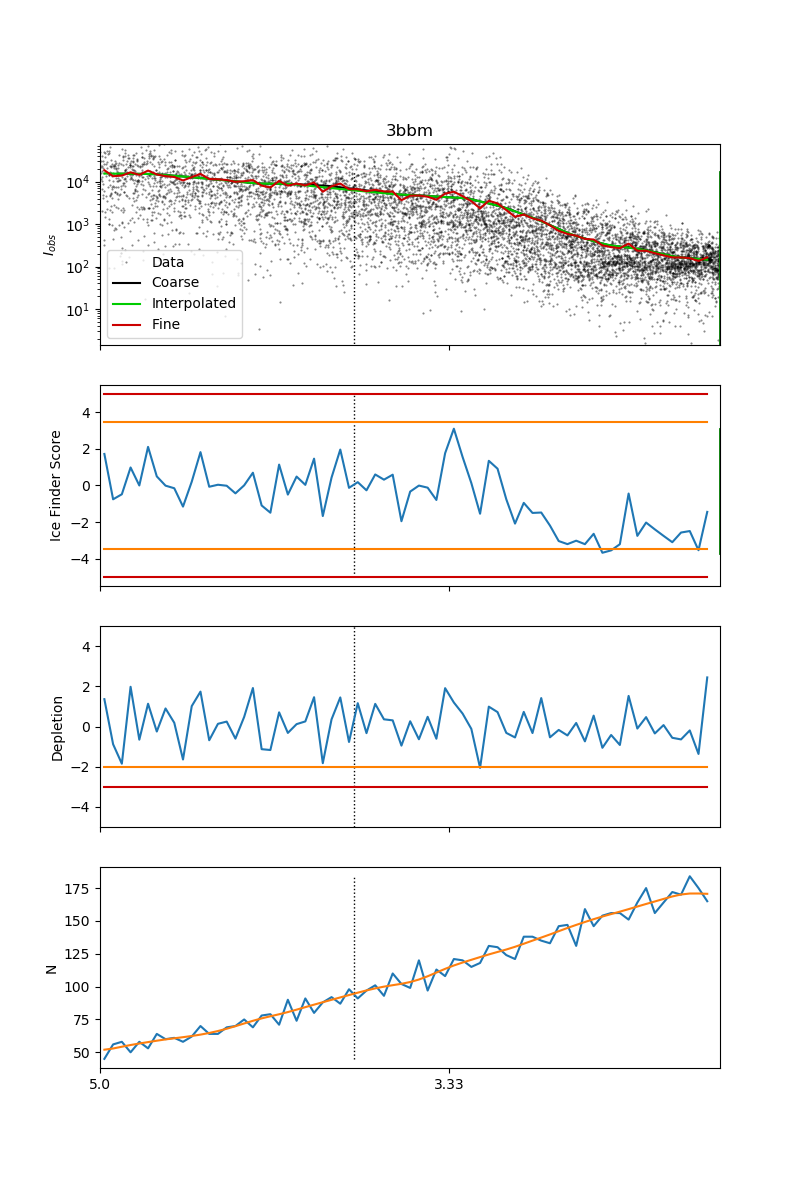

Supplement: Supplementary file 3 [file d-77-00540-sup3.zip › IceBiasingImages/3bbm.png]

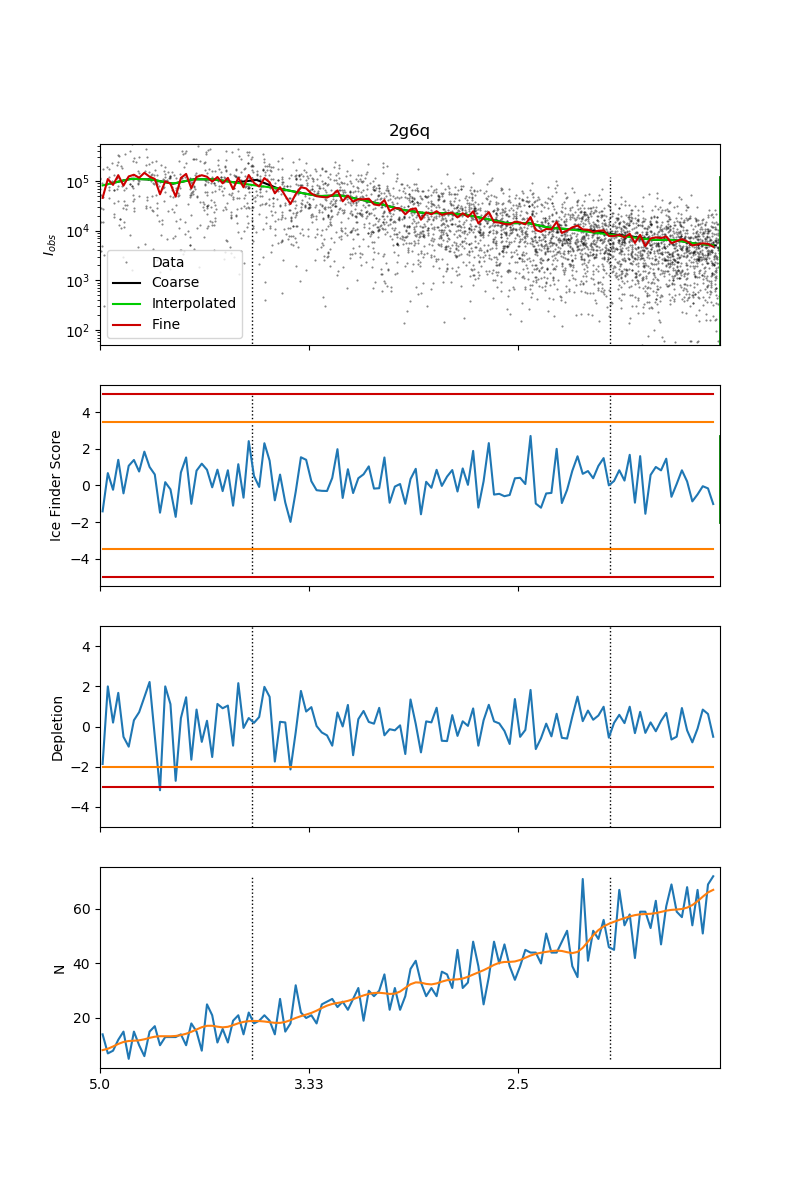

Supplement: Supplementary file 3 [file d-77-00540-sup3.zip › IceBiasingImages/2g6q.png]

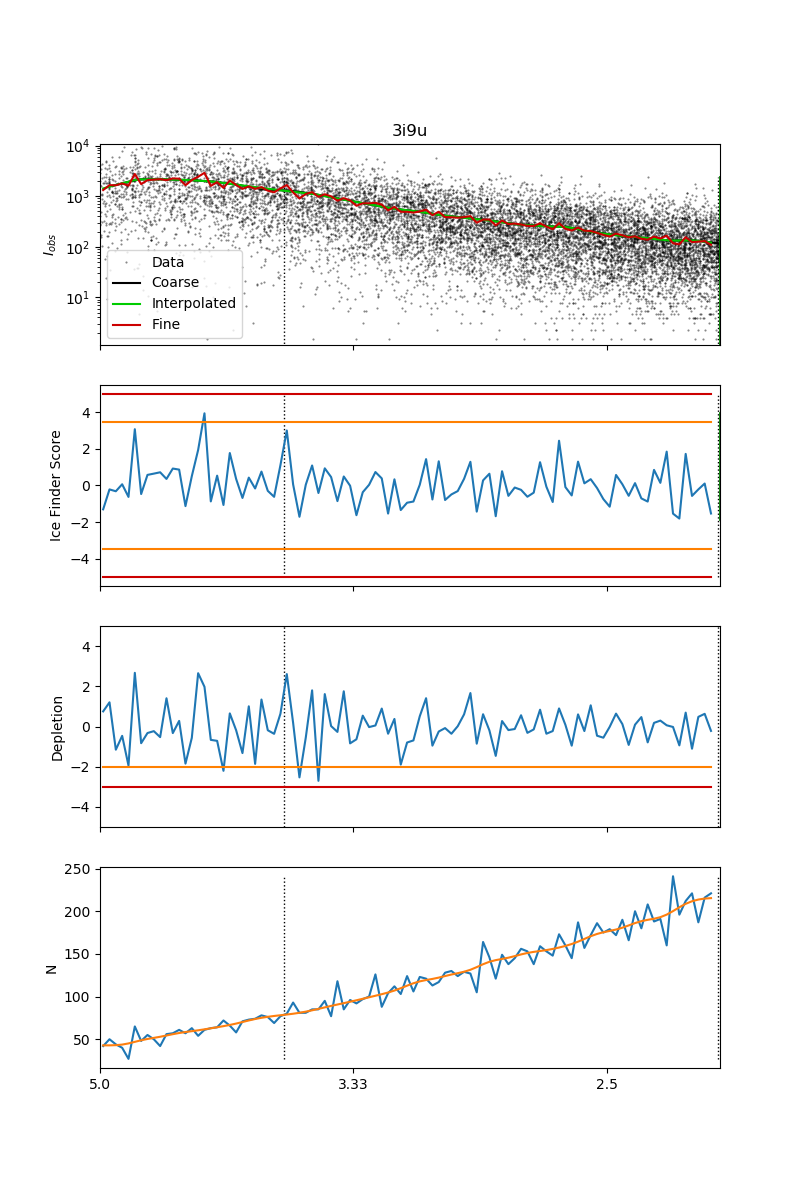

Supplement: Supplementary file 3 [file d-77-00540-sup3.zip › IceBiasingImages/3i9u.png]

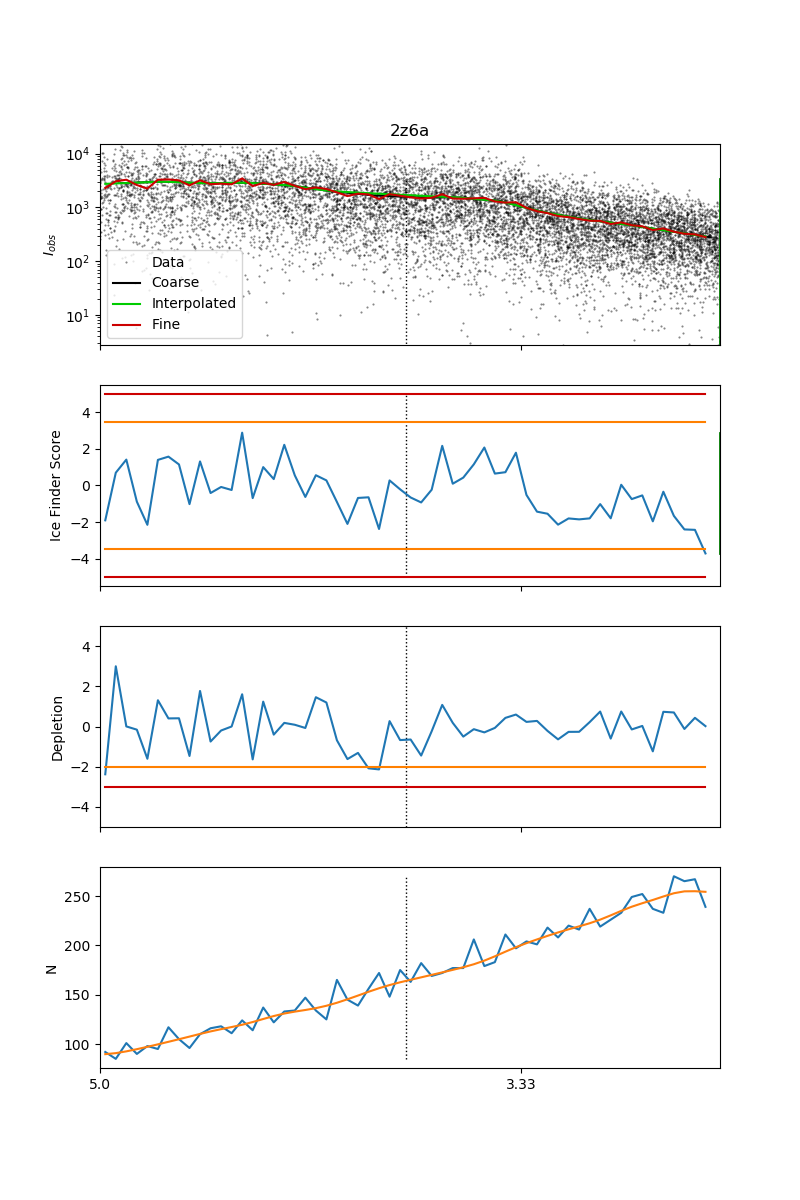

Supplement: Supplementary file 3 [file d-77-00540-sup3.zip › IceBiasingImages/2z6a.png]

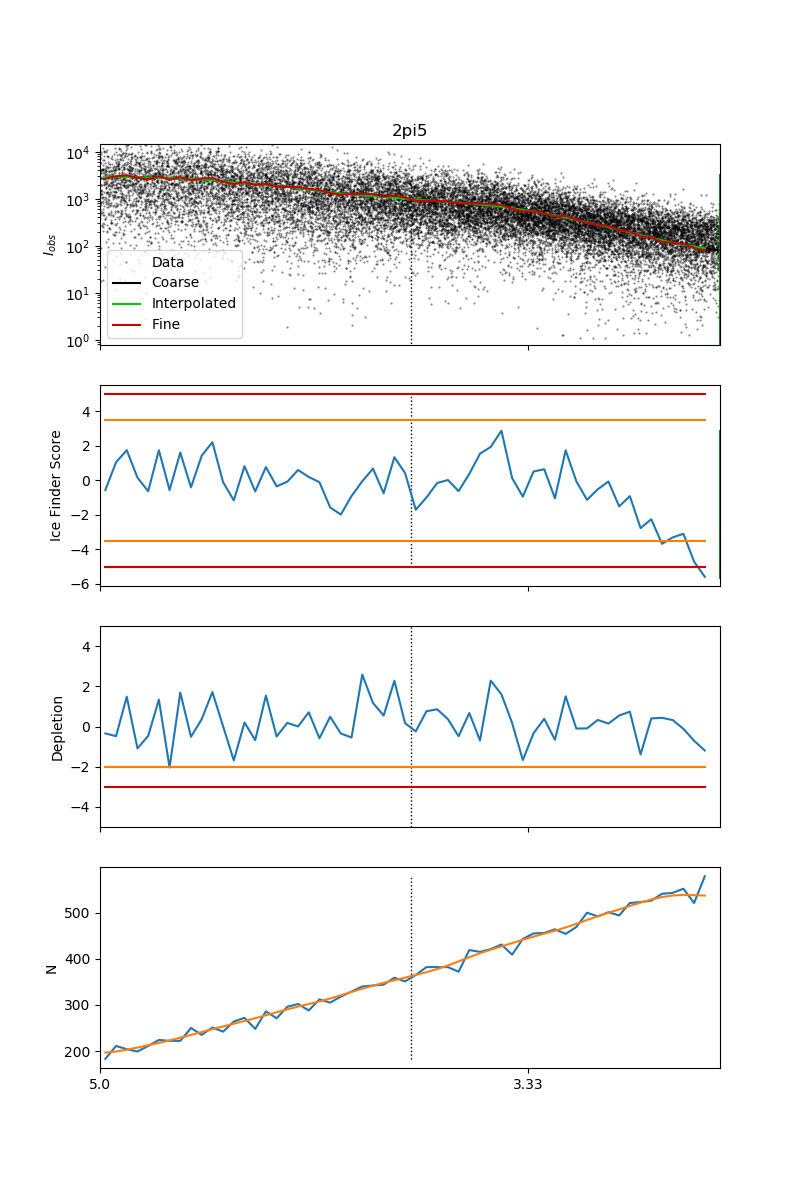

Supplement: Supplementary file 3 [file d-77-00540-sup3.zip › IceBiasingImages/2pi5.png]

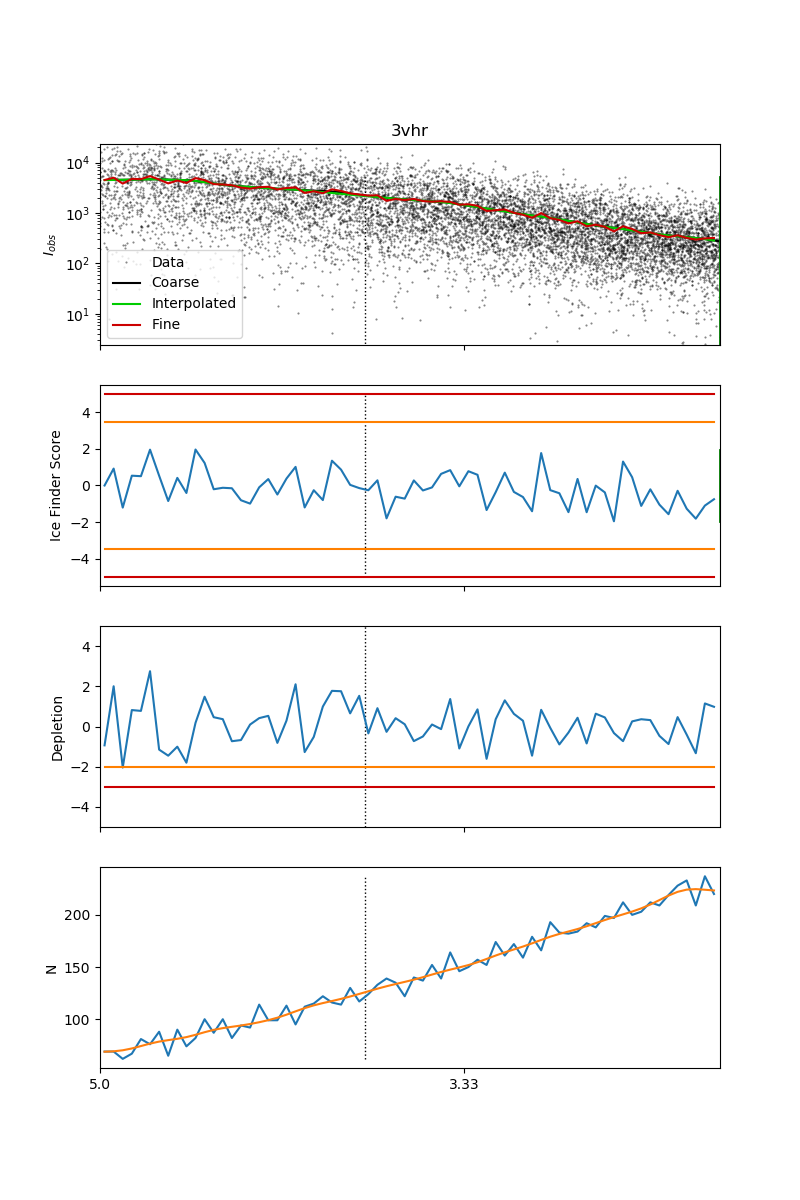

Supplement: Supplementary file 3 [file d-77-00540-sup3.zip › IceBiasingImages/3vhr.png]

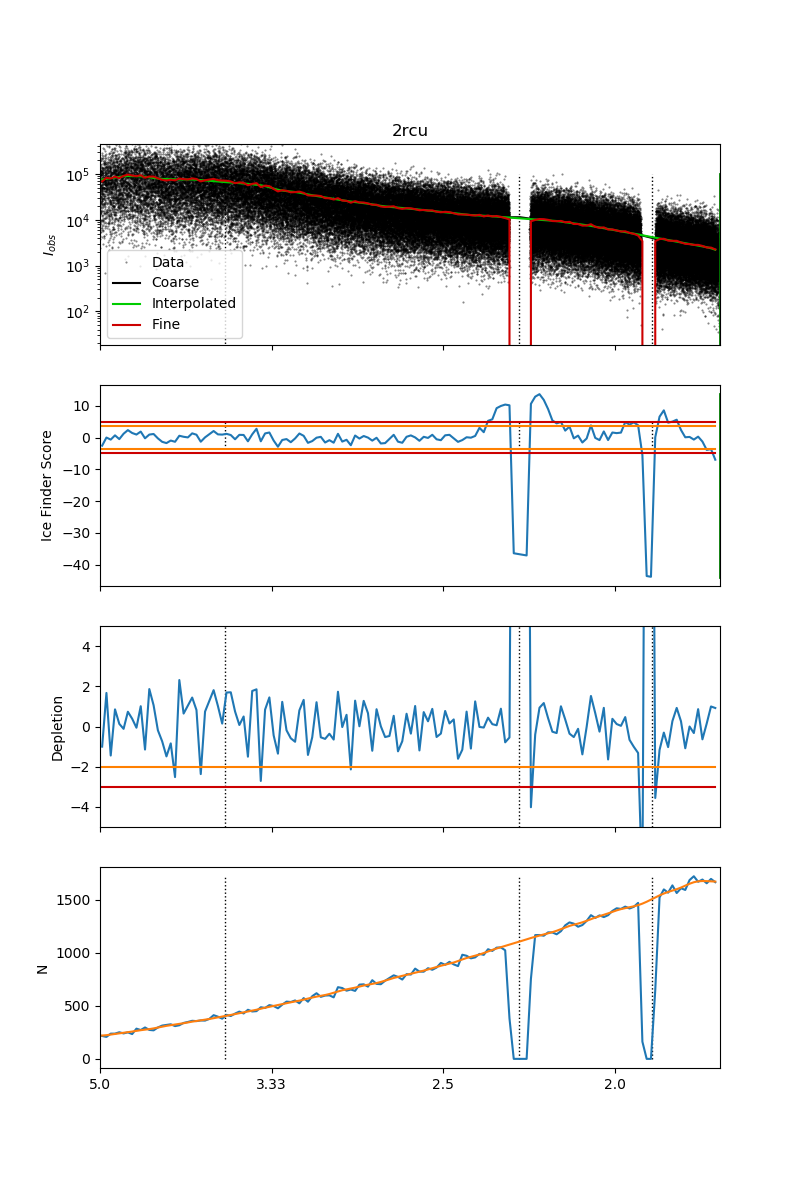

Supplement: Supplementary file 3 [file d-77-00540-sup3.zip › IceBiasingImages/2rcu.png]

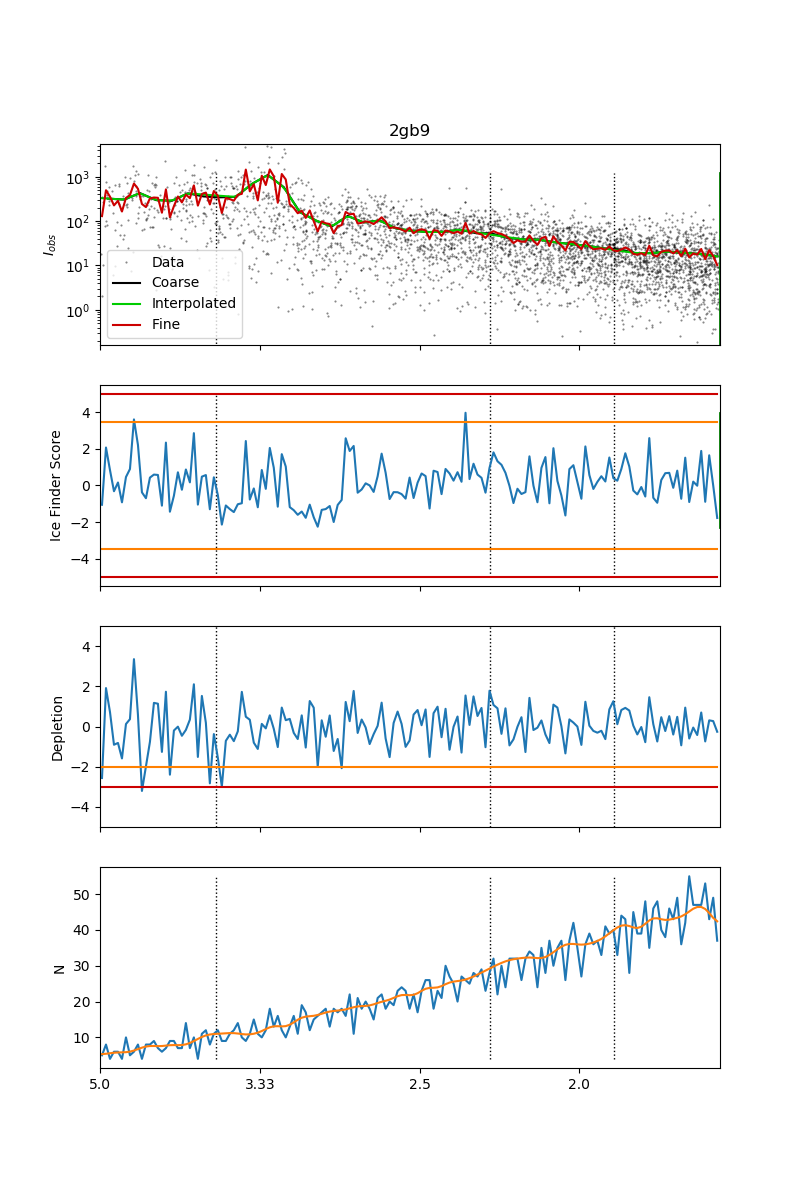

Supplement: Supplementary file 3 [file d-77-00540-sup3.zip › IceBiasingImages/2gb9.png]

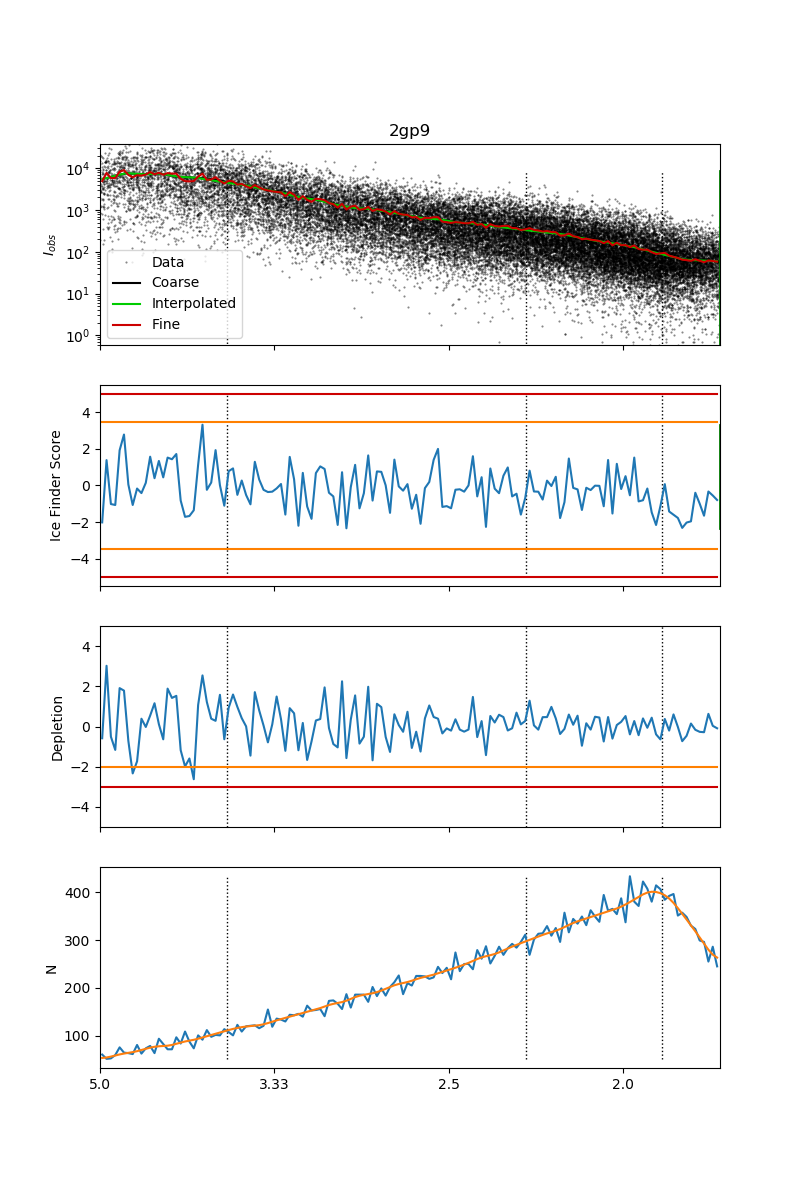

Supplement: Supplementary file 3 [file d-77-00540-sup3.zip › IceBiasingImages/2gp9.png]

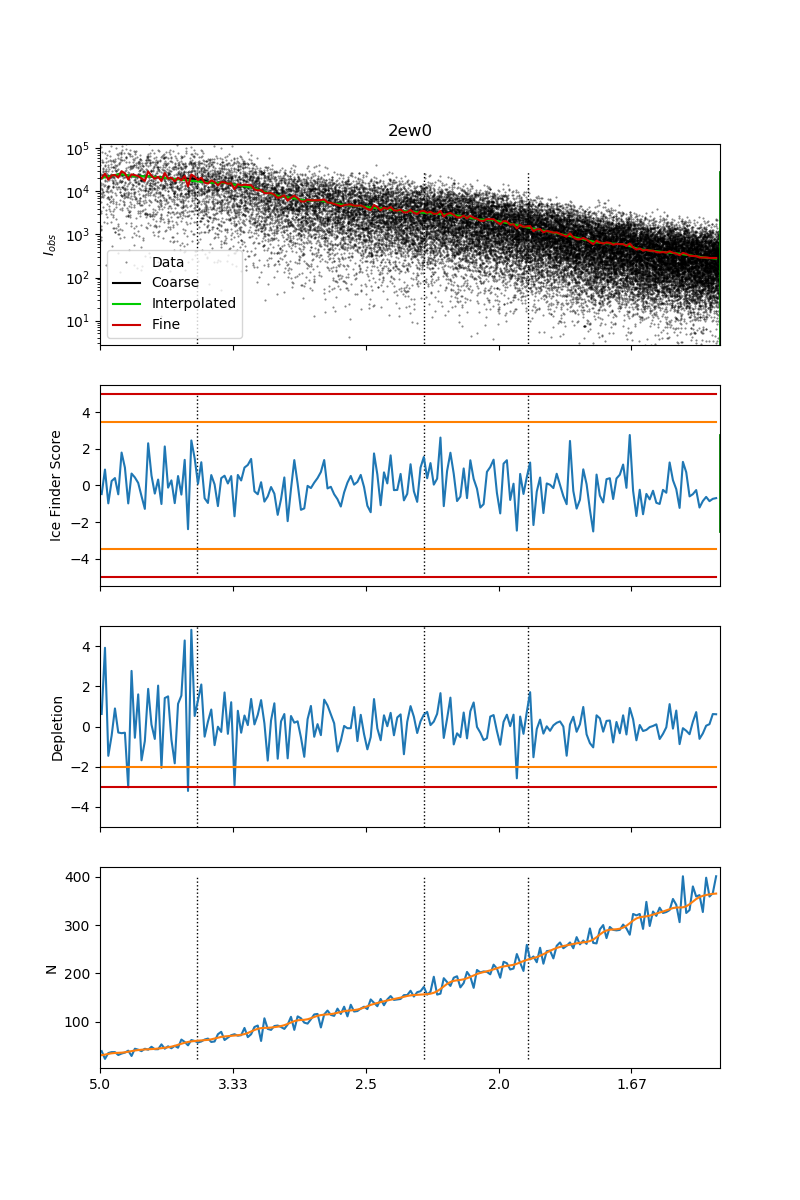

Supplement: Supplementary file 3 [file d-77-00540-sup3.zip › IceBiasingImages/2ew0.png]

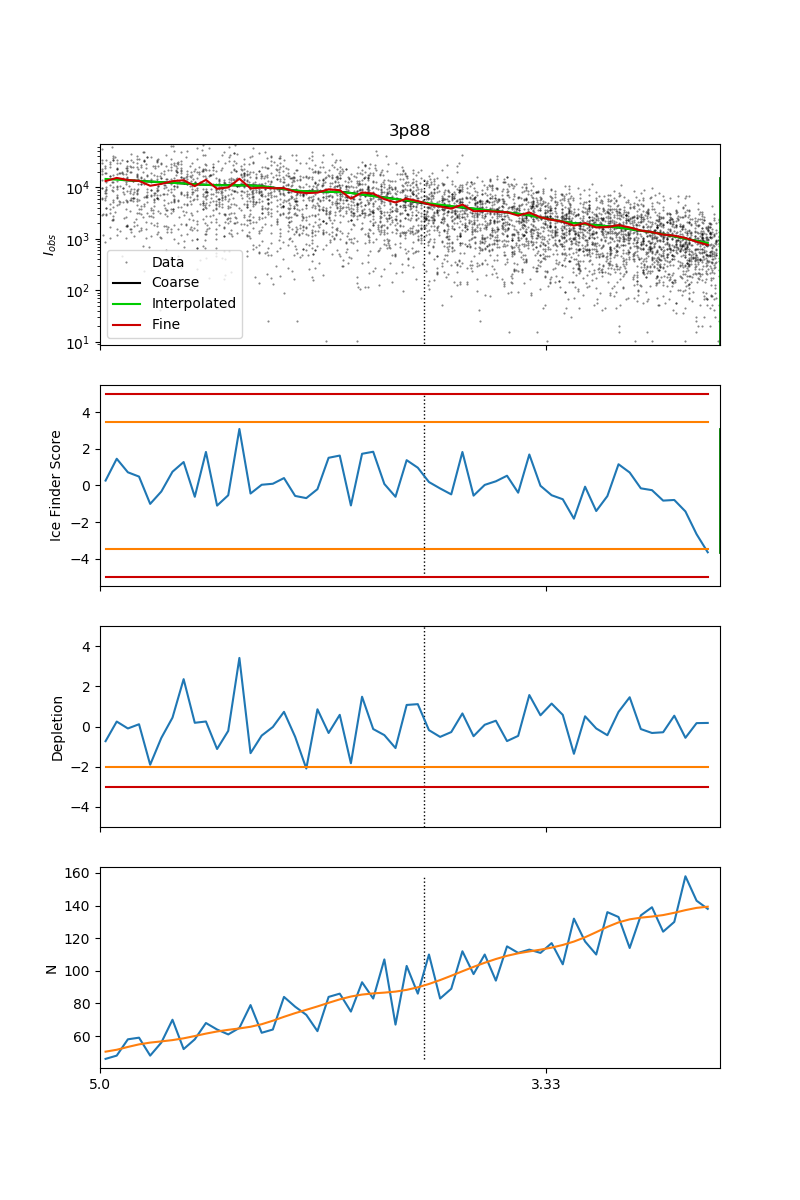

Supplement: Supplementary file 3 [file d-77-00540-sup3.zip › IceBiasingImages/3p88.png]

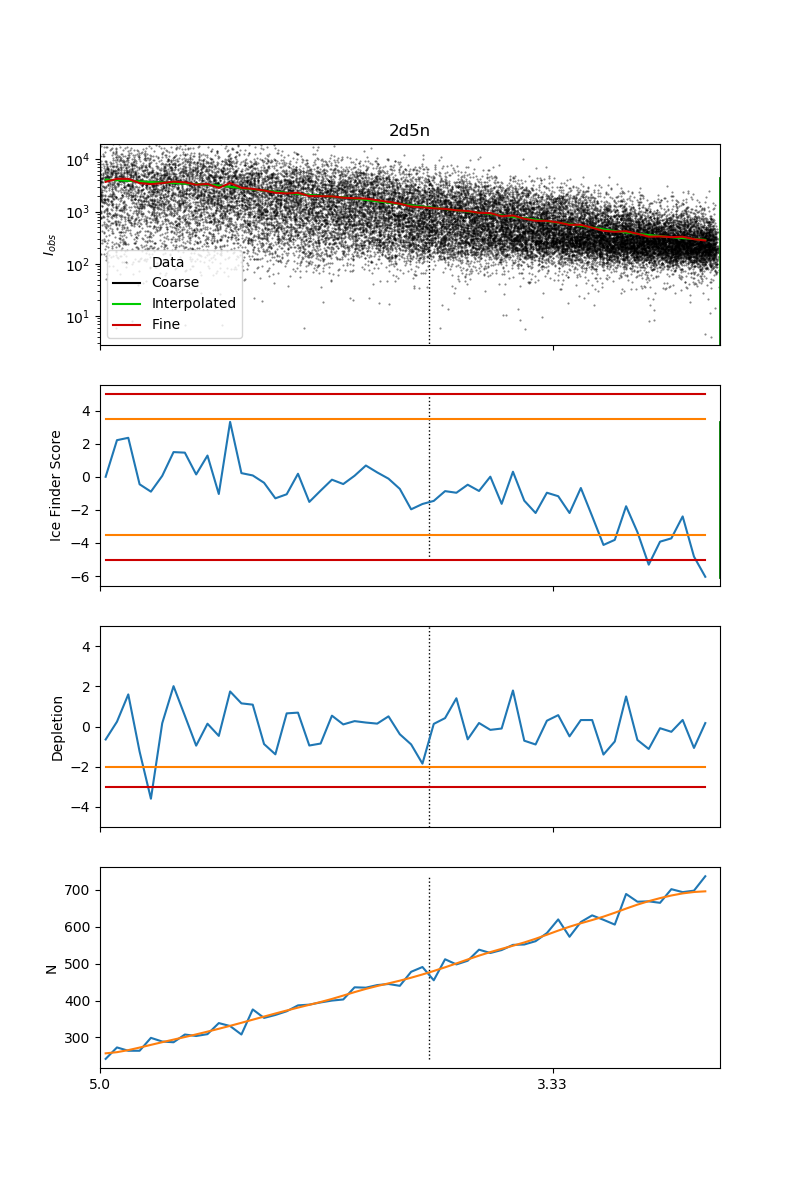

Supplement: Supplementary file 3 [file d-77-00540-sup3.zip › IceBiasingImages/2d5n.png]

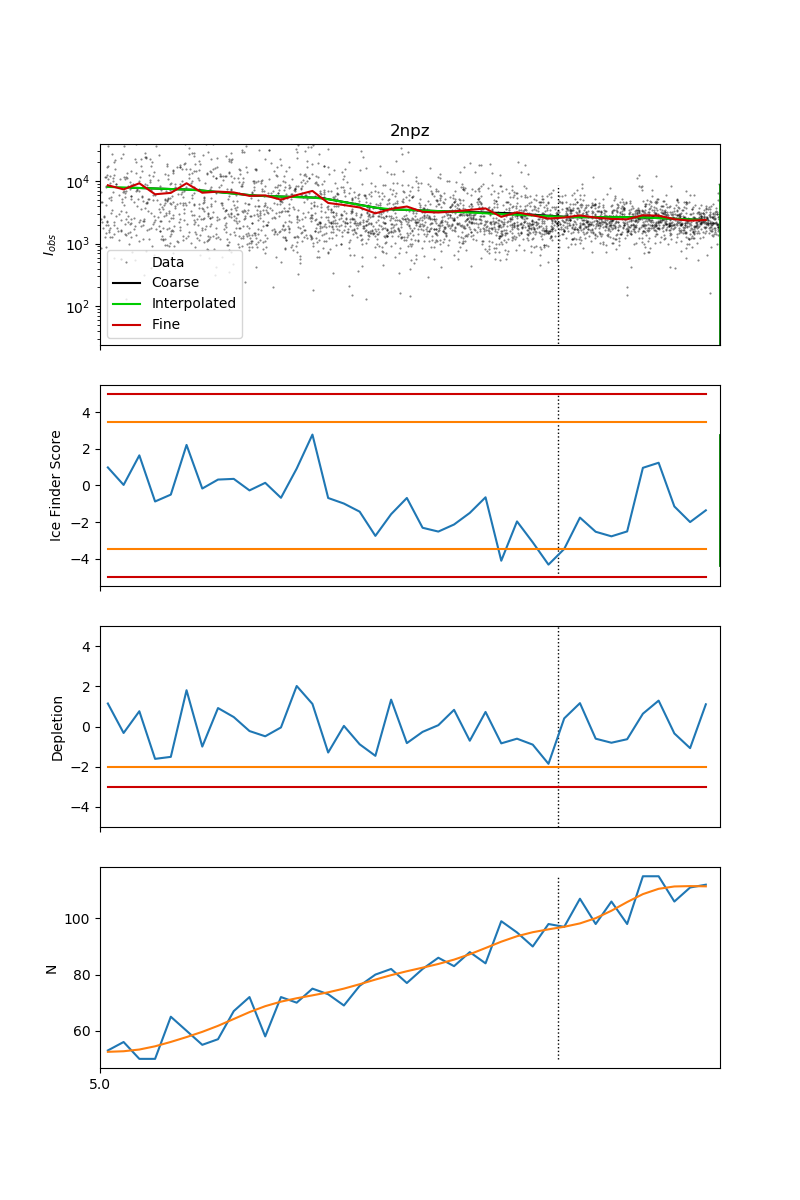

Supplement: Supplementary file 3 [file d-77-00540-sup3.zip › IceBiasingImages/2npz.png]

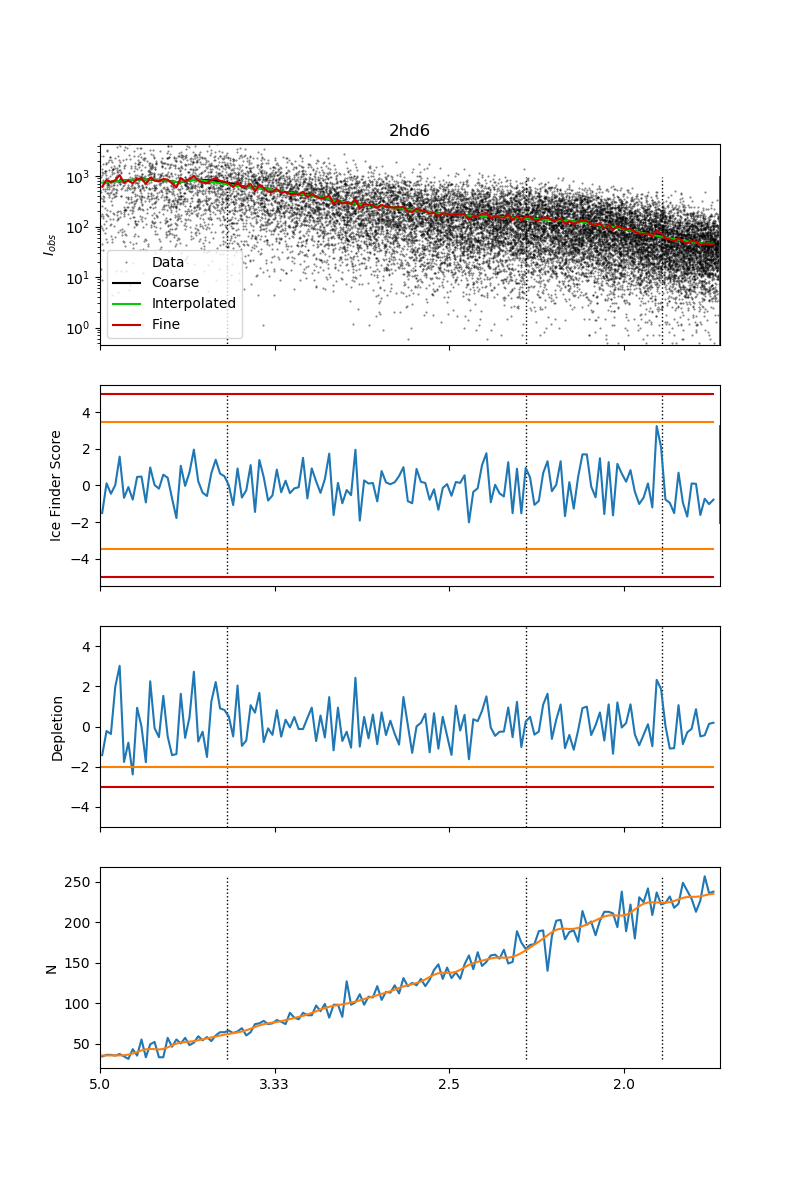

Supplement: Supplementary file 3 [file d-77-00540-sup3.zip › IceBiasingImages/2hd6.png]

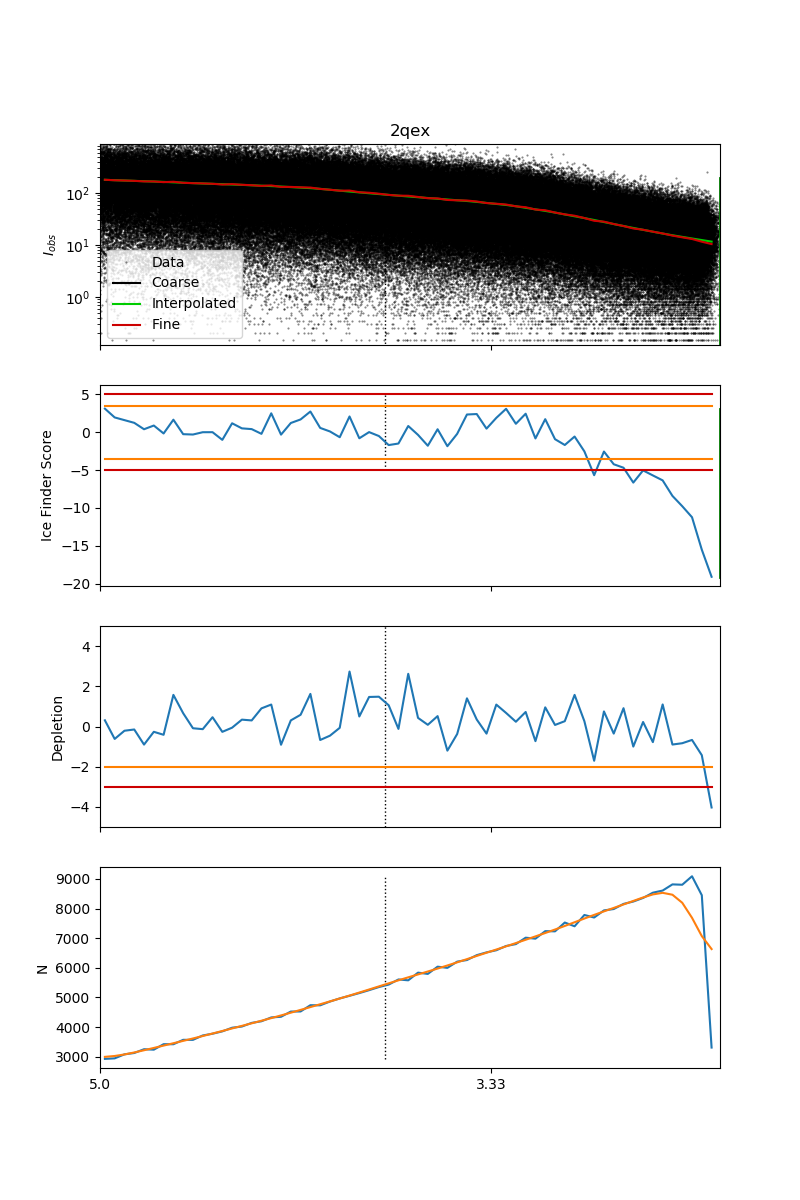

Supplement: Supplementary file 3 [file d-77-00540-sup3.zip › IceBiasingImages/2qex.png]

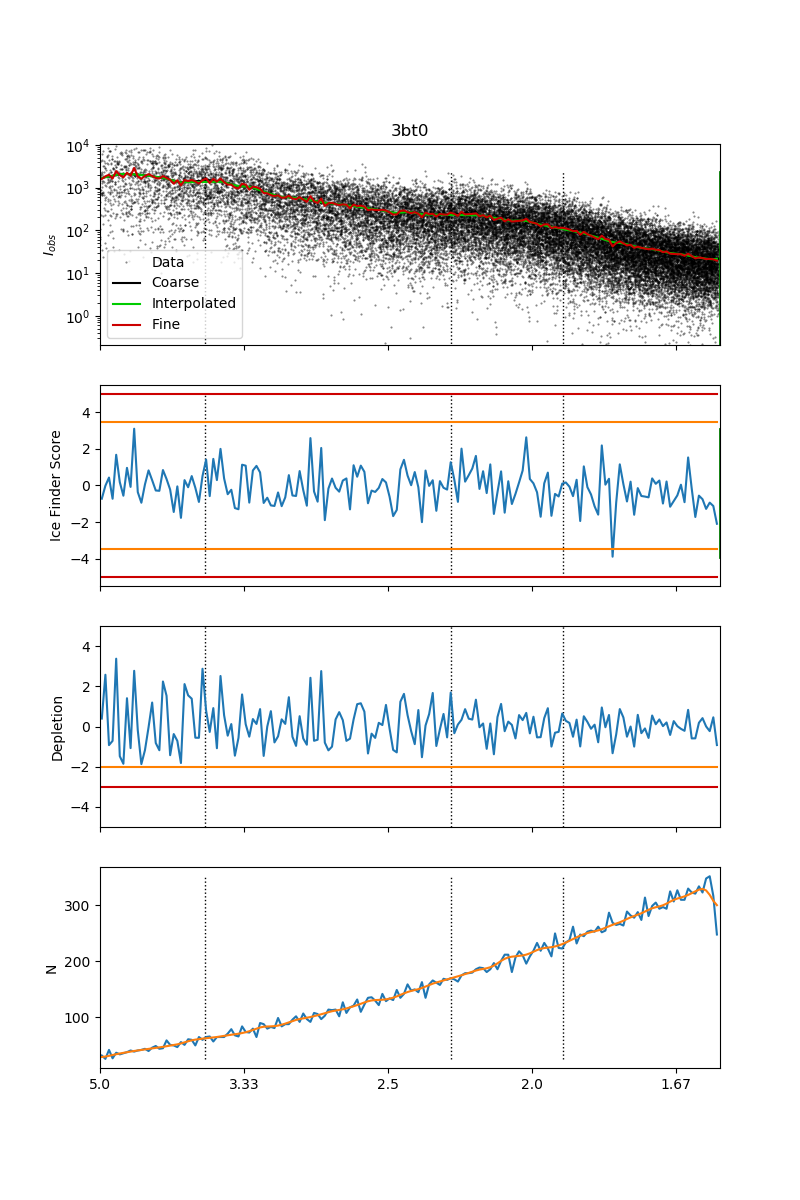

Supplement: Supplementary file 3 [file d-77-00540-sup3.zip › IceBiasingImages/3bt0.png]

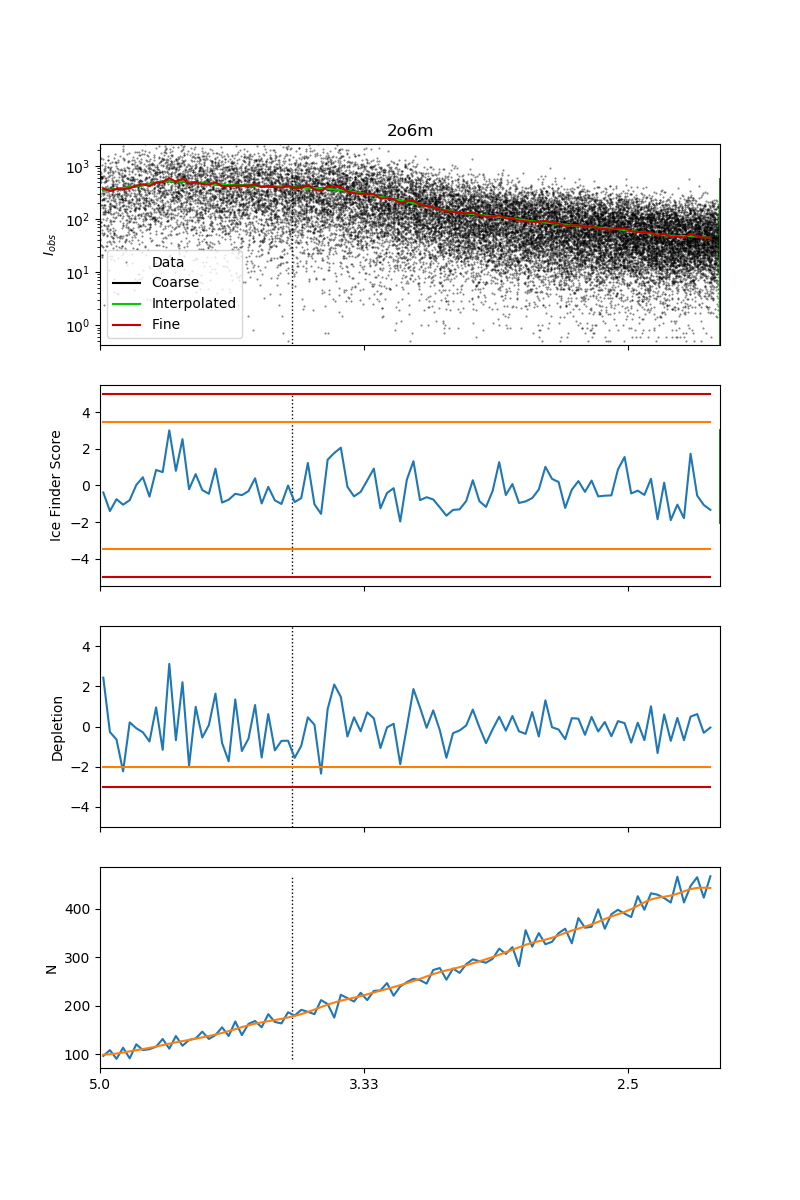

Supplement: Supplementary file 3 [file d-77-00540-sup3.zip › IceBiasingImages/2o6m.png]

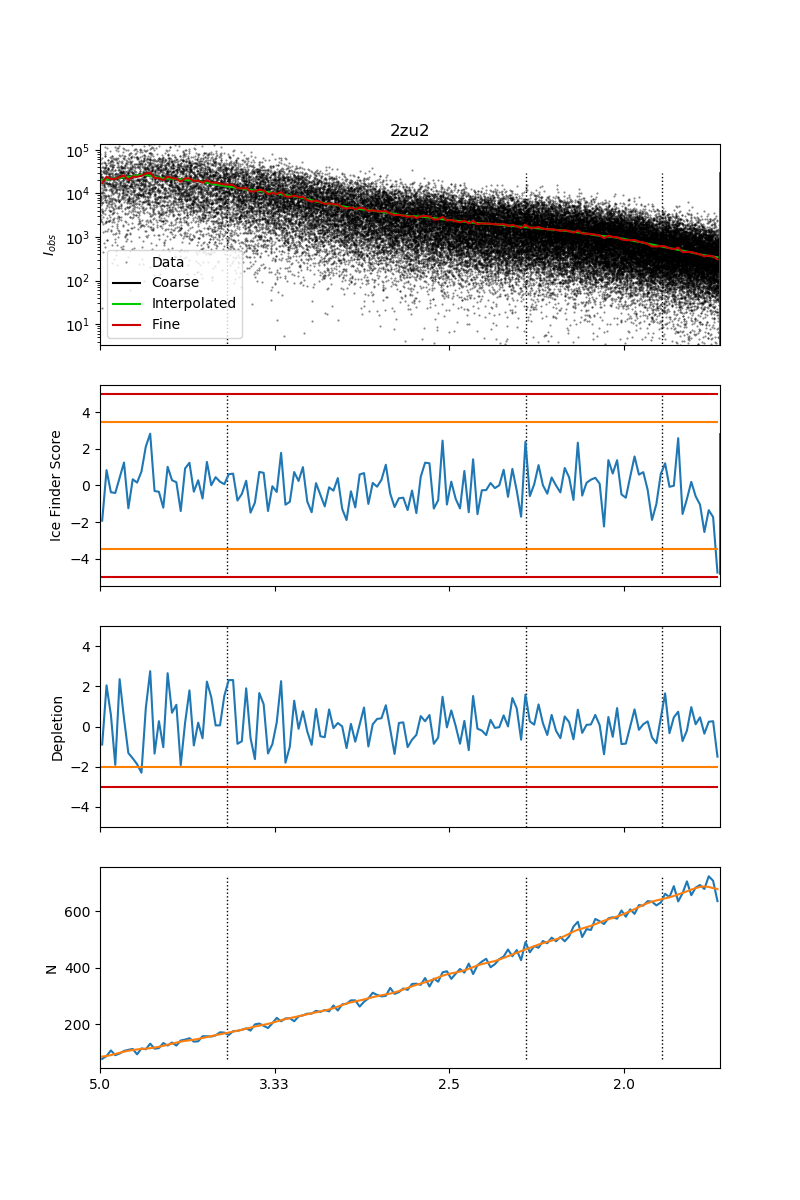

Supplement: Supplementary file 3 [file d-77-00540-sup3.zip › IceBiasingImages/2zu2.png]

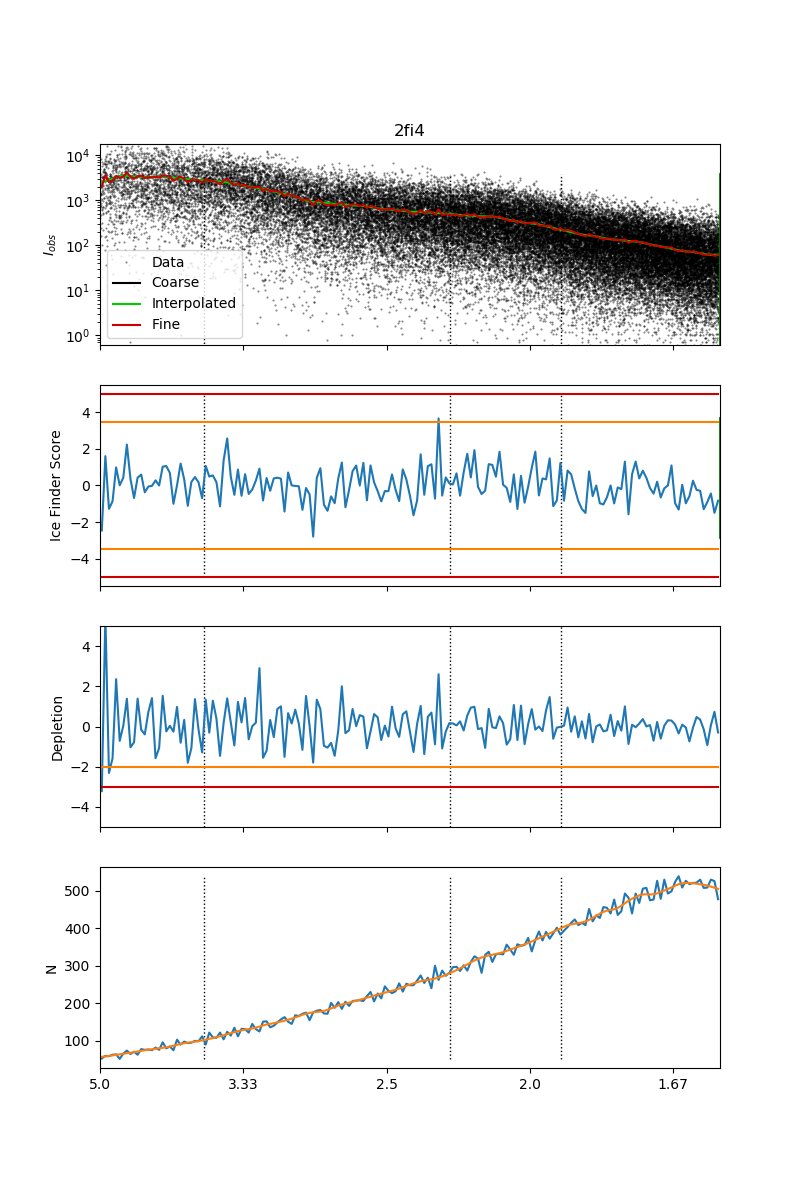

Supplement: Supplementary file 3 [file d-77-00540-sup3.zip › IceBiasingImages/2fi4.png]

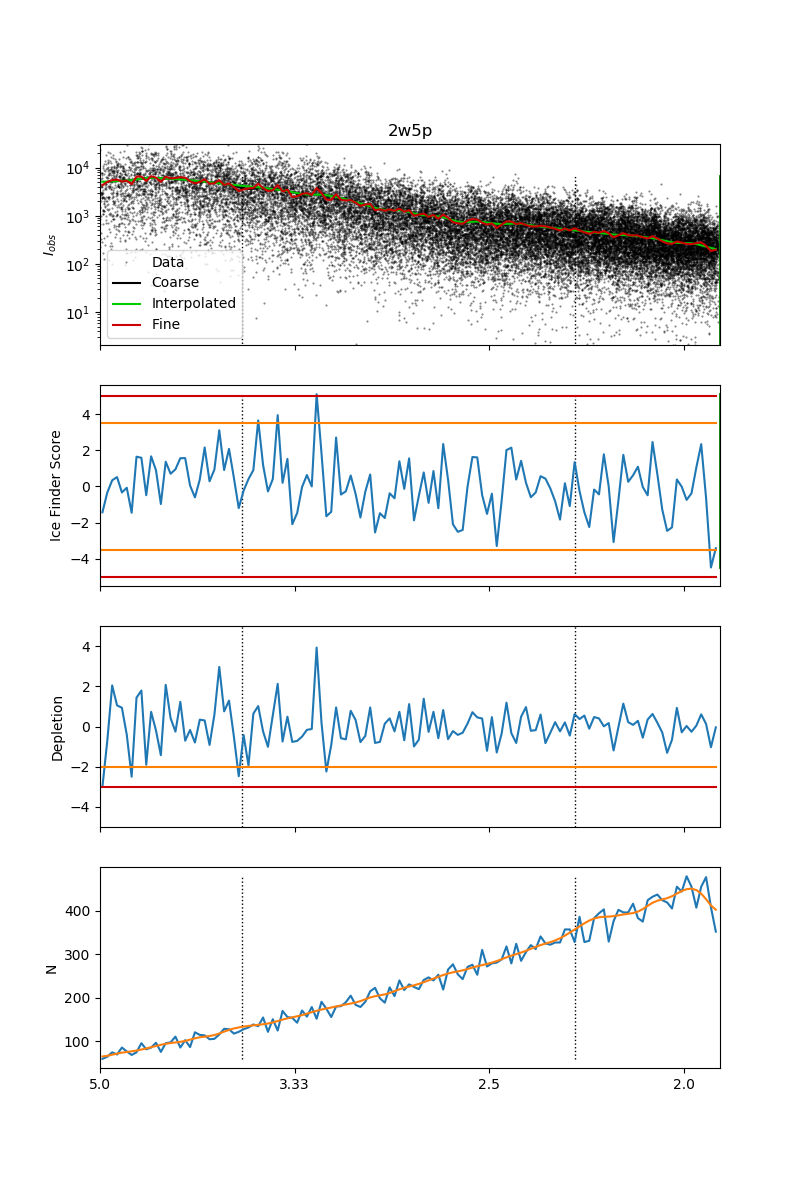

Supplement: Supplementary file 3 [file d-77-00540-sup3.zip › IceBiasingImages/2w5p.png]

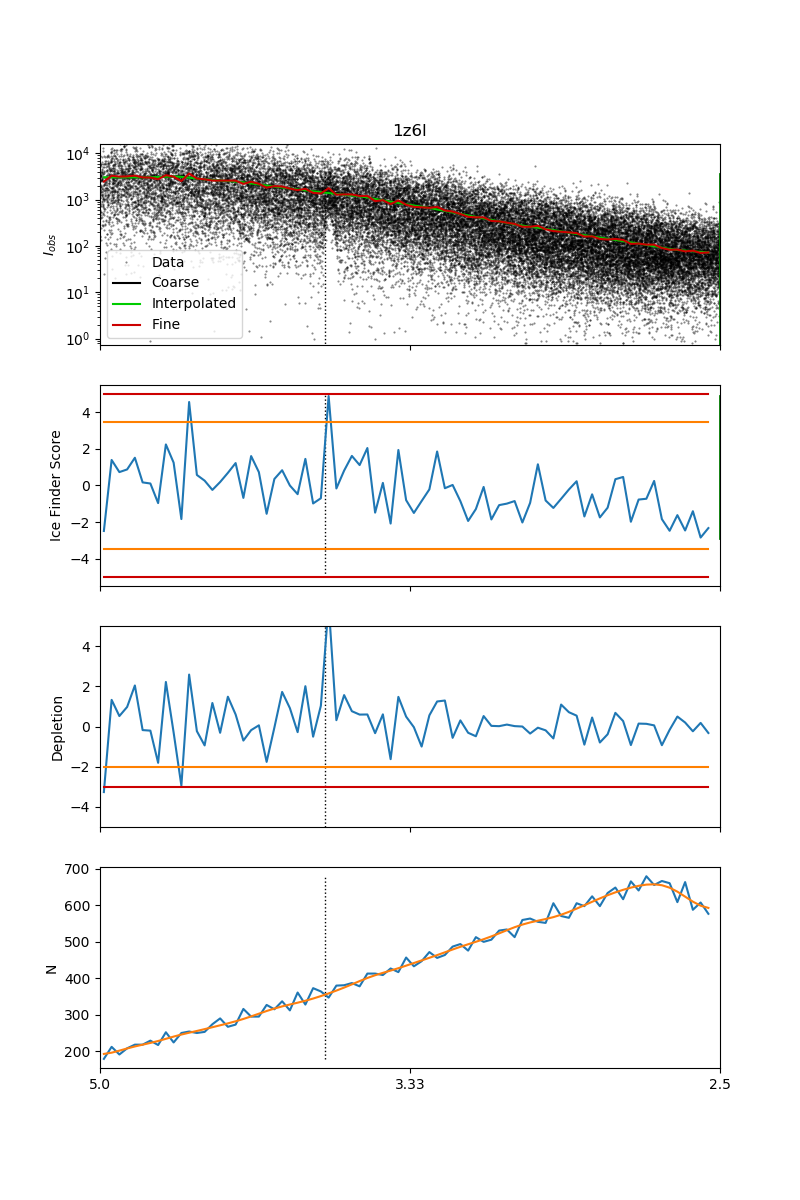

Supplement: Supplementary file 3 [file d-77-00540-sup3.zip › IceBiasingImages/1z6l.png]

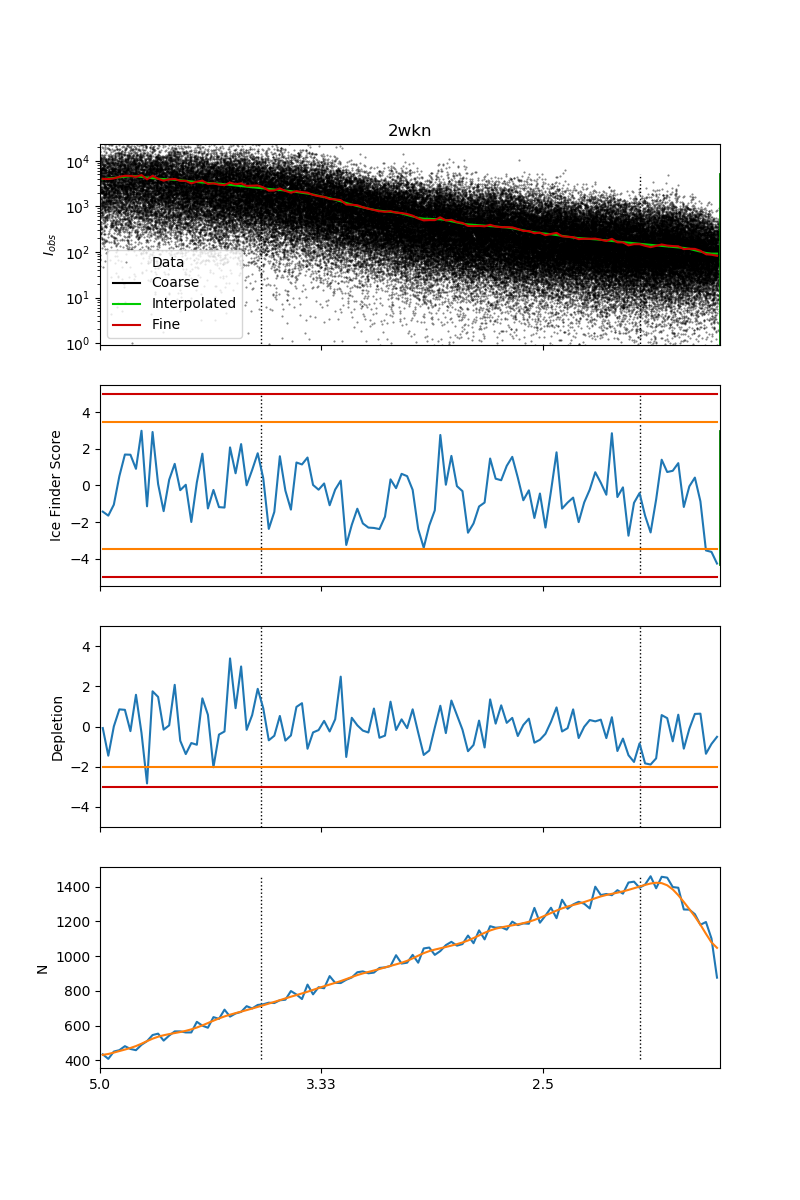

Supplement: Supplementary file 3 [file d-77-00540-sup3.zip › IceBiasingImages/2wkn.png]

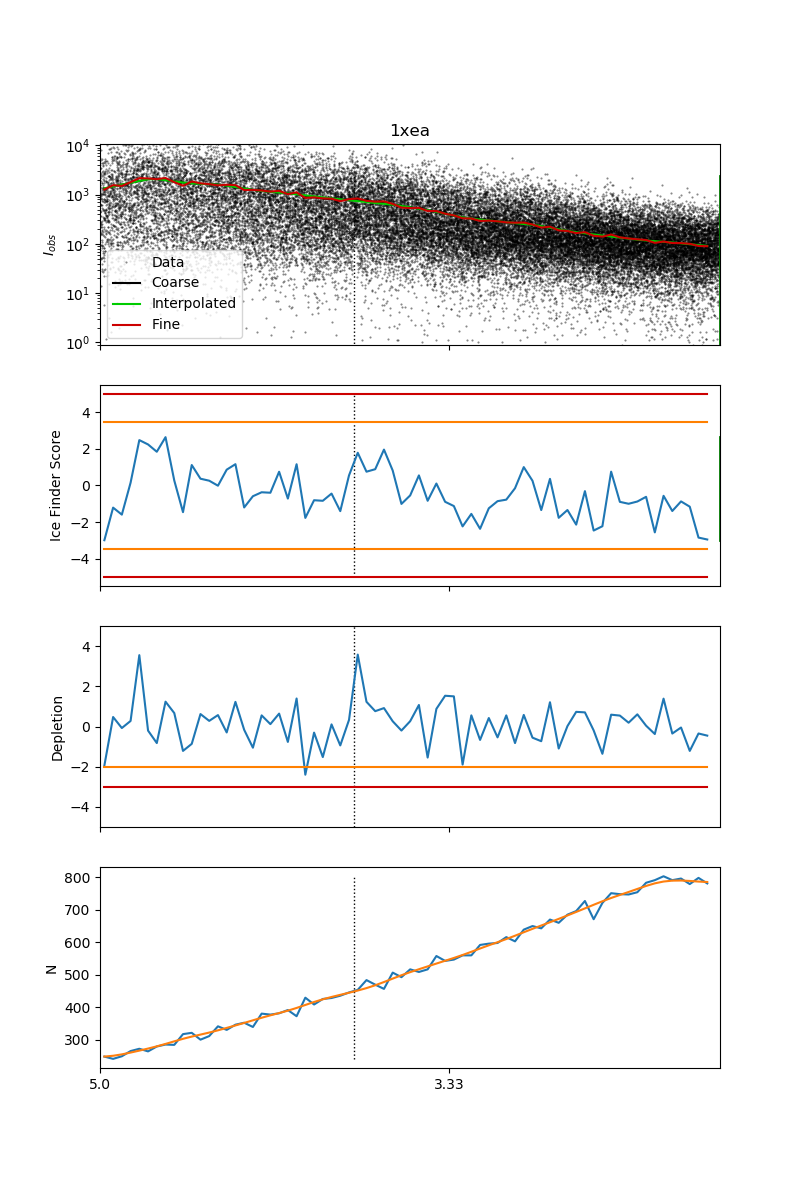

Supplement: Supplementary file 3 [file d-77-00540-sup3.zip › IceBiasingImages/1xea.png]

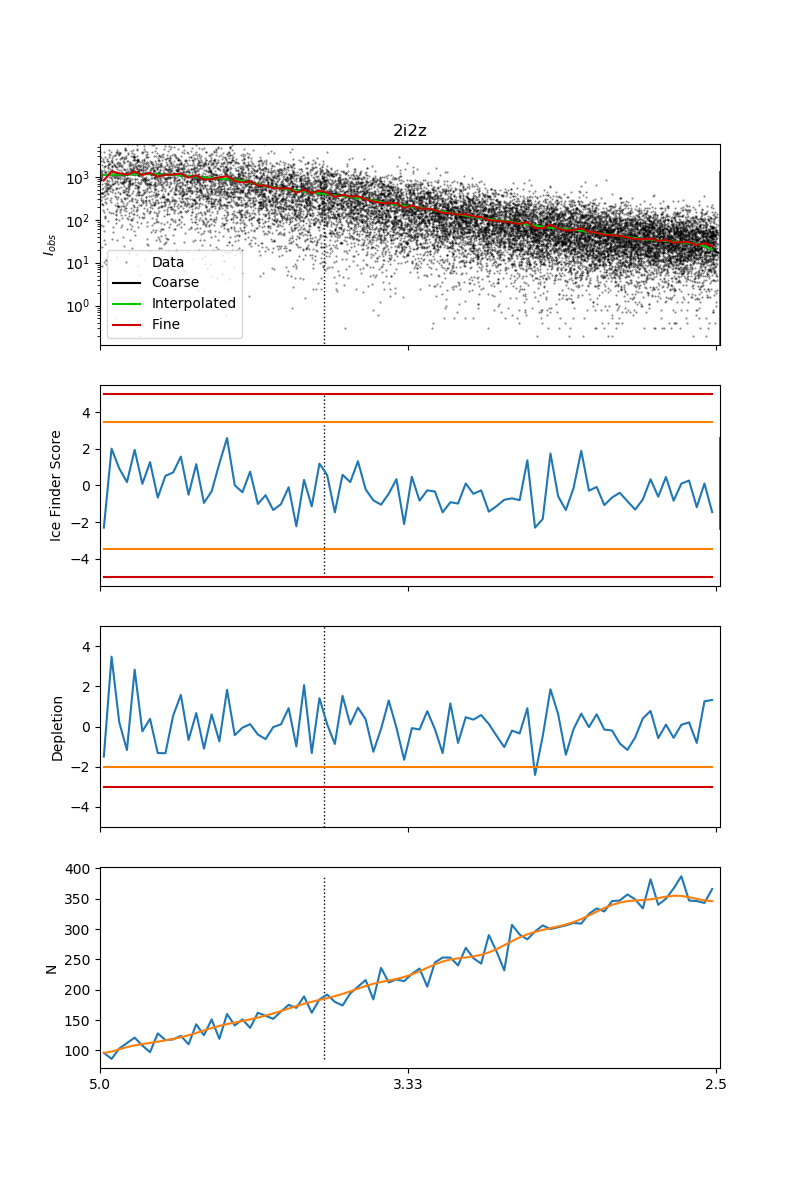

Supplement: Supplementary file 3 [file d-77-00540-sup3.zip › IceBiasingImages/2i2z.png]

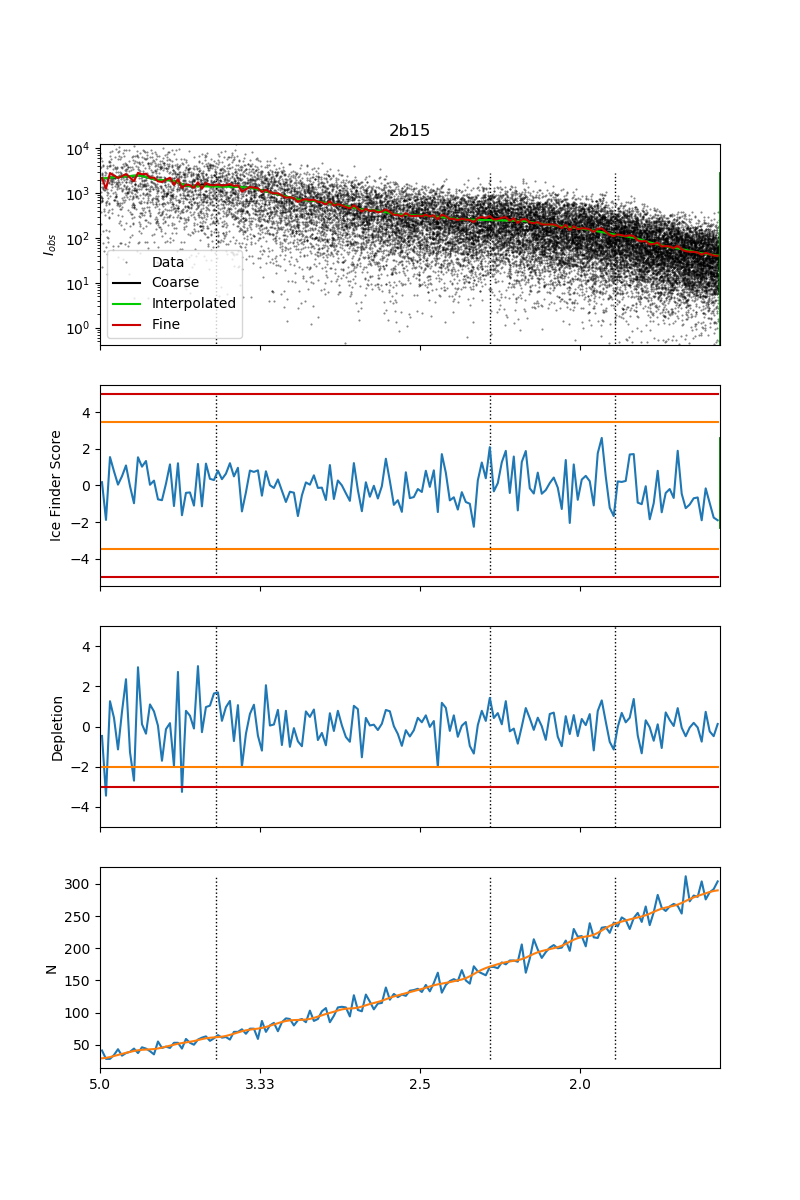

Supplement: Supplementary file 3 [file d-77-00540-sup3.zip › IceBiasingImages/2b15.png]

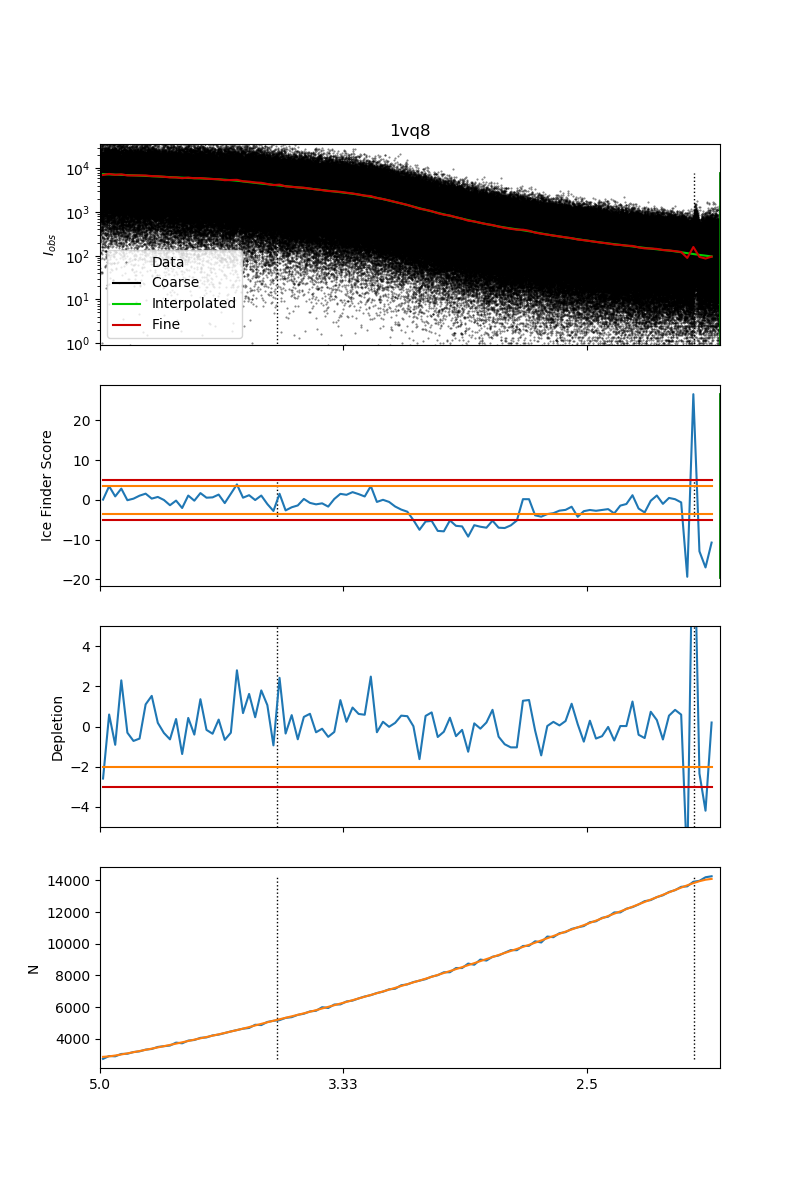

Supplement: Supplementary file 3 [file d-77-00540-sup3.zip › IceBiasingImages/1vq8.png]

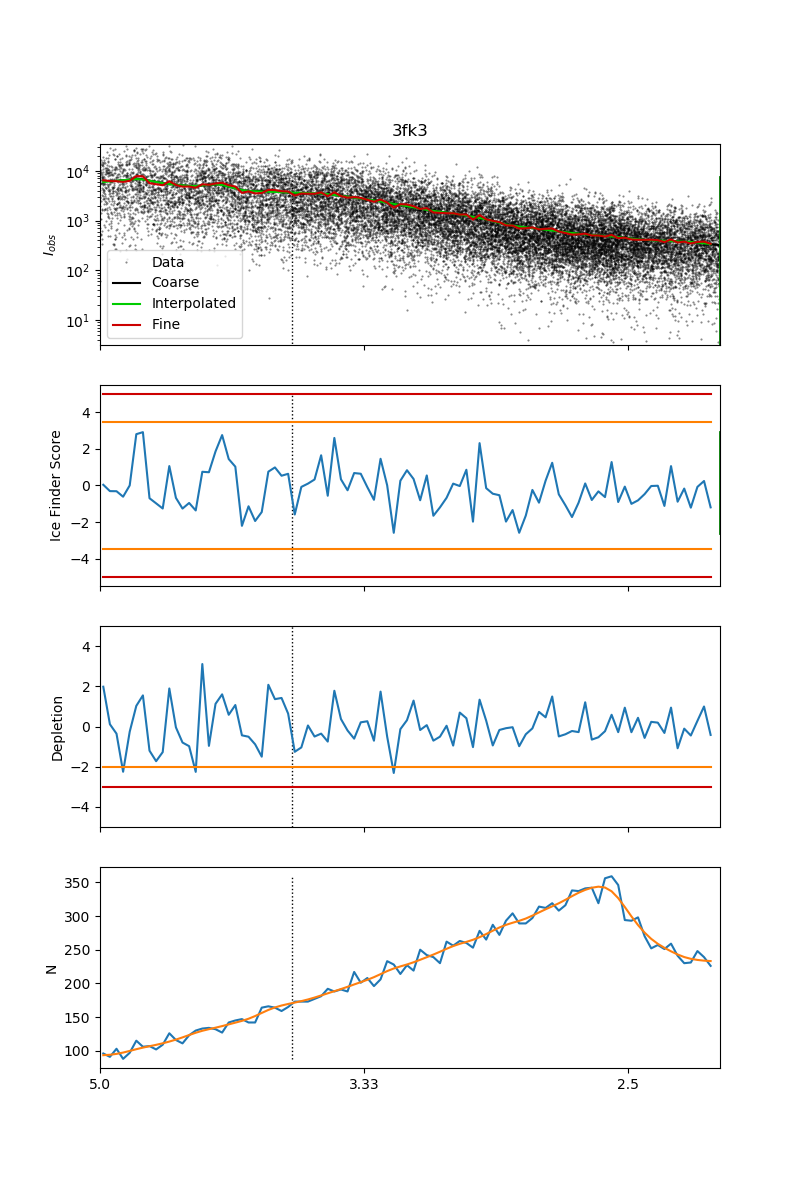

Supplement: Supplementary file 3 [file d-77-00540-sup3.zip › IceBiasingImages/3fk3.png]

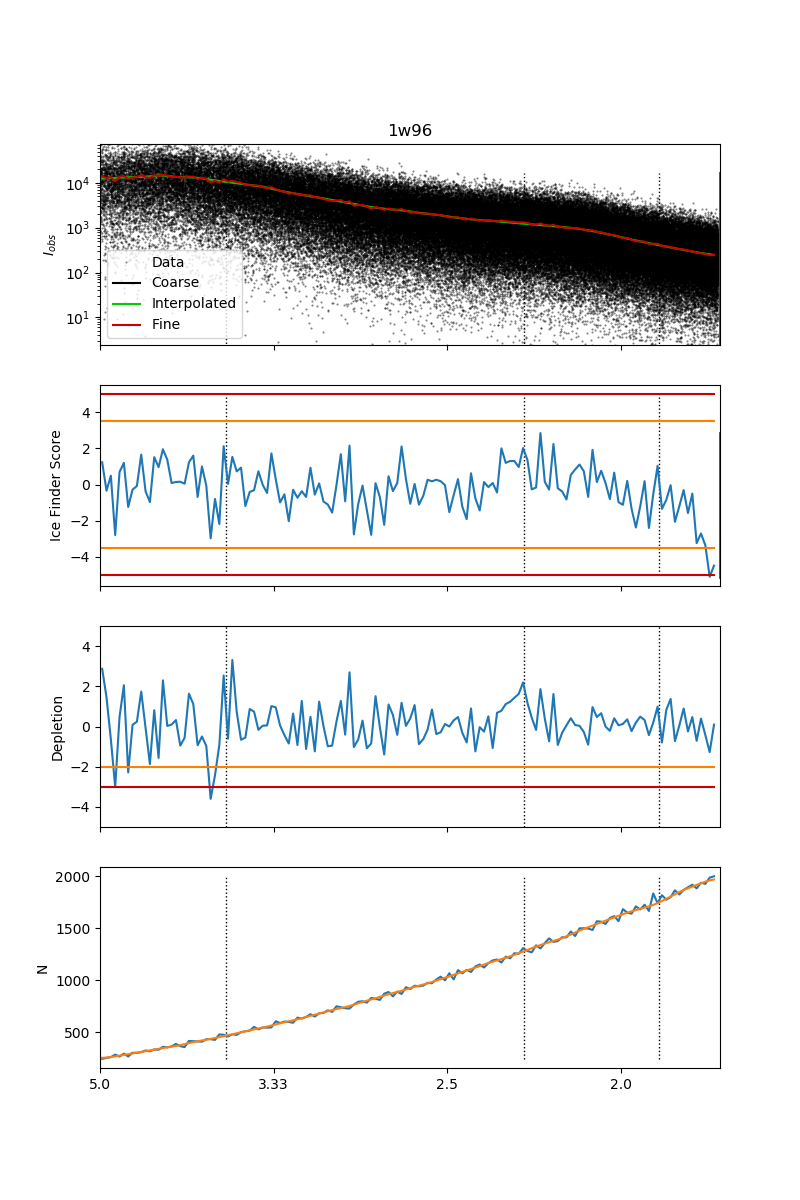

Supplement: Supplementary file 3 [file d-77-00540-sup3.zip › IceBiasingImages/1w96.png]

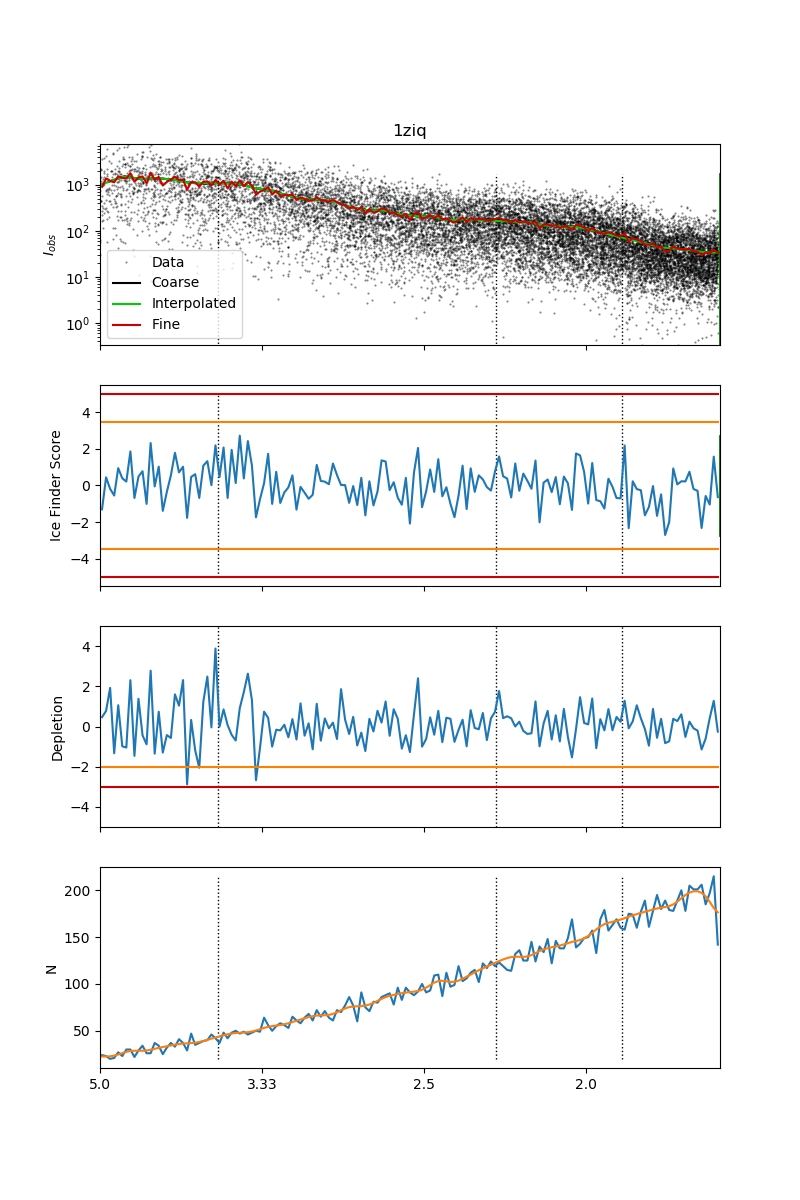

Supplement: Supplementary file 3 [file d-77-00540-sup3.zip › IceBiasingImages/1ziq.png]

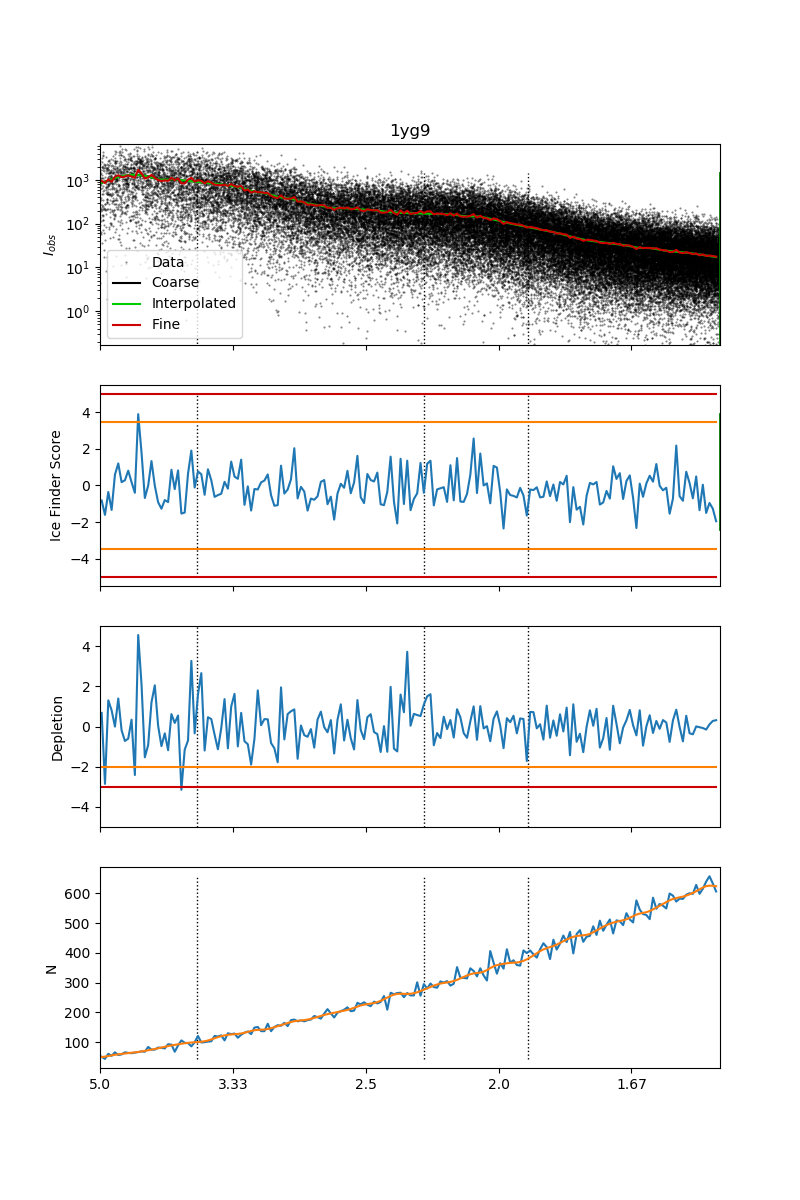

Supplement: Supplementary file 3 [file d-77-00540-sup3.zip › IceBiasingImages/1yg9.png]

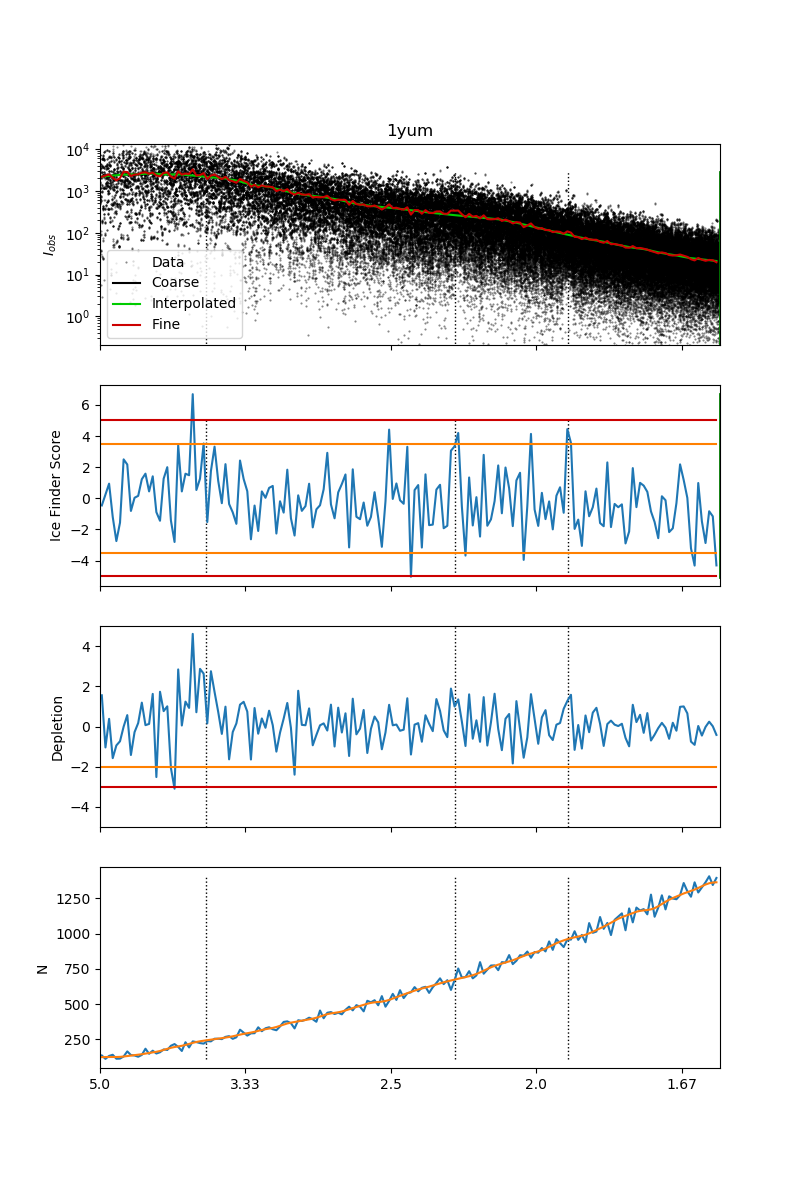

Supplement: Supplementary file 3 [file d-77-00540-sup3.zip › IceBiasingImages/1yum.png]

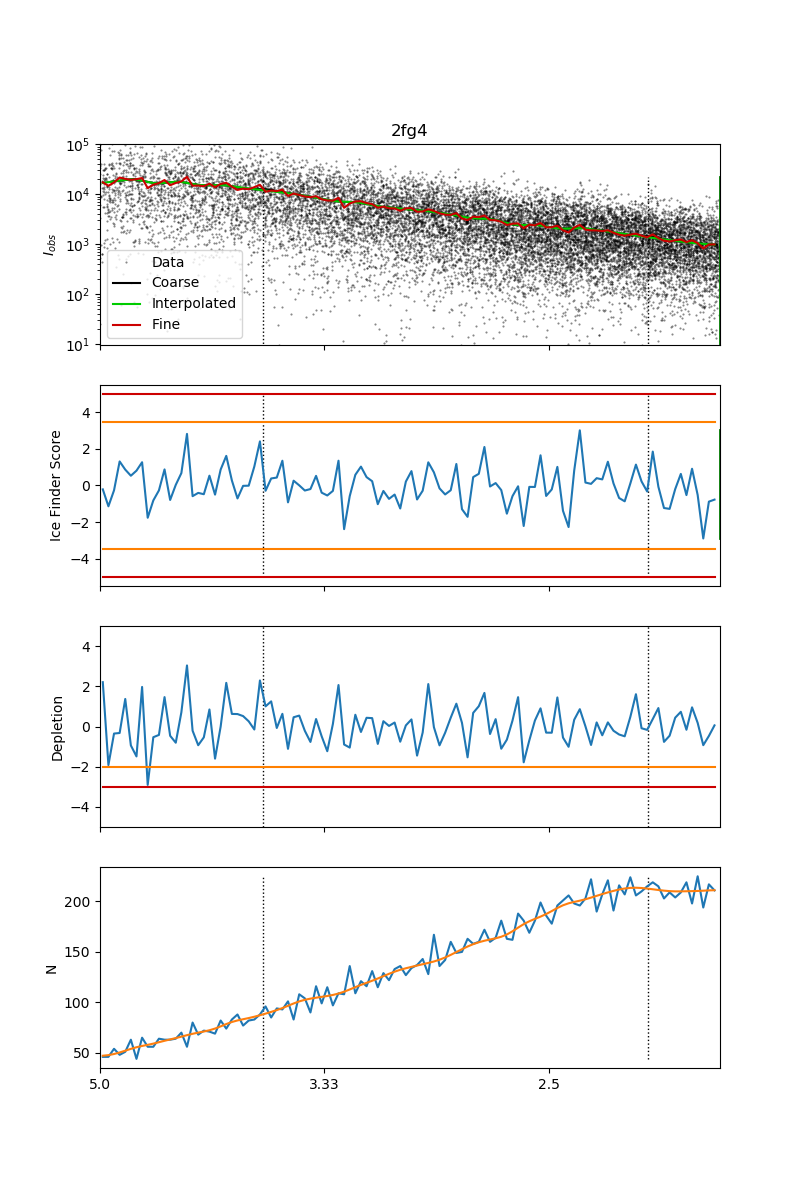

Supplement: Supplementary file 3 [file d-77-00540-sup3.zip › IceBiasingImages/2fg4.png]

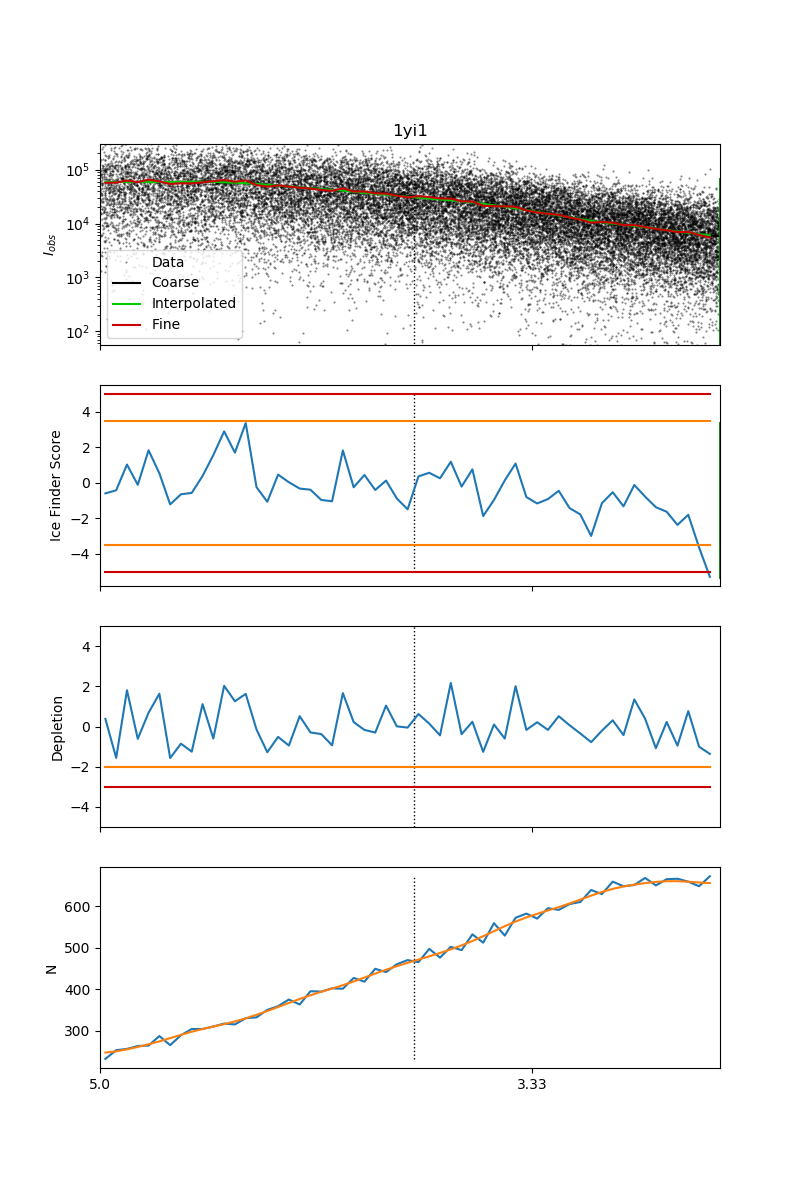

Supplement: Supplementary file 3 [file d-77-00540-sup3.zip › IceBiasingImages/1yi1.png]

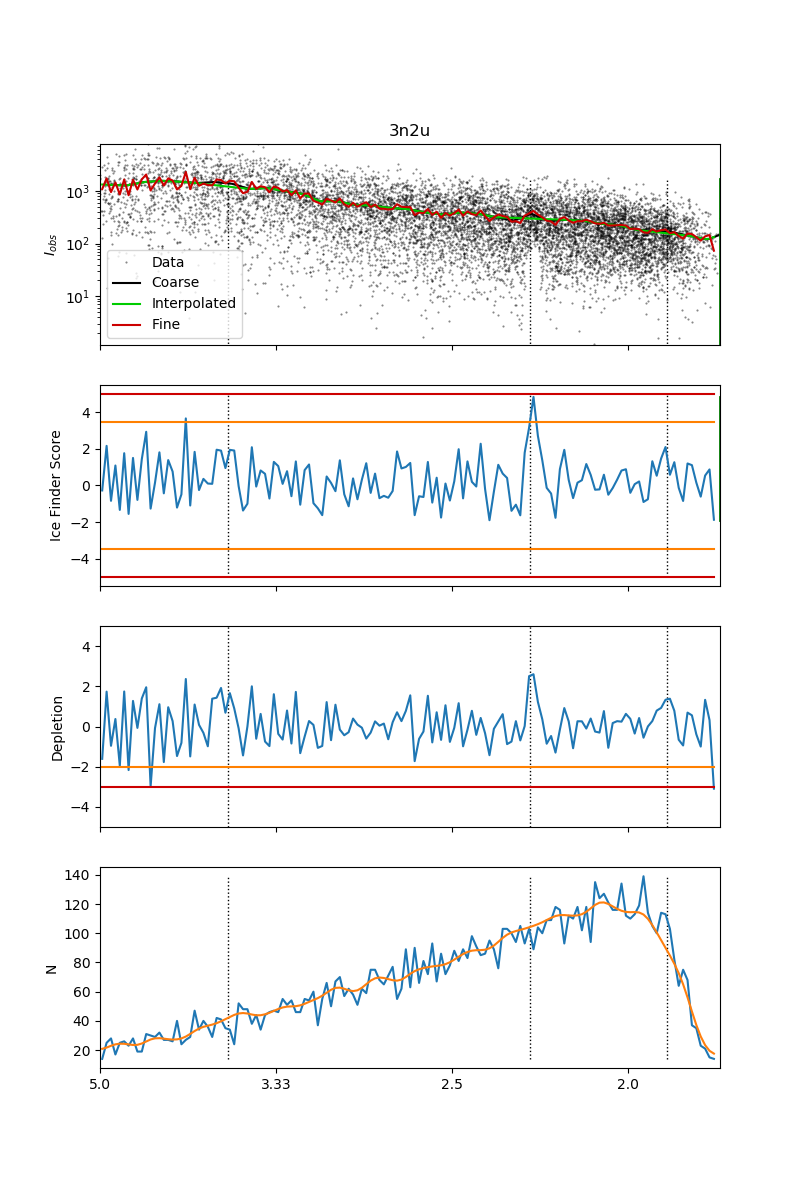

Supplement: Supplementary file 3 [file d-77-00540-sup3.zip › IceBiasingImages/3n2u.png]

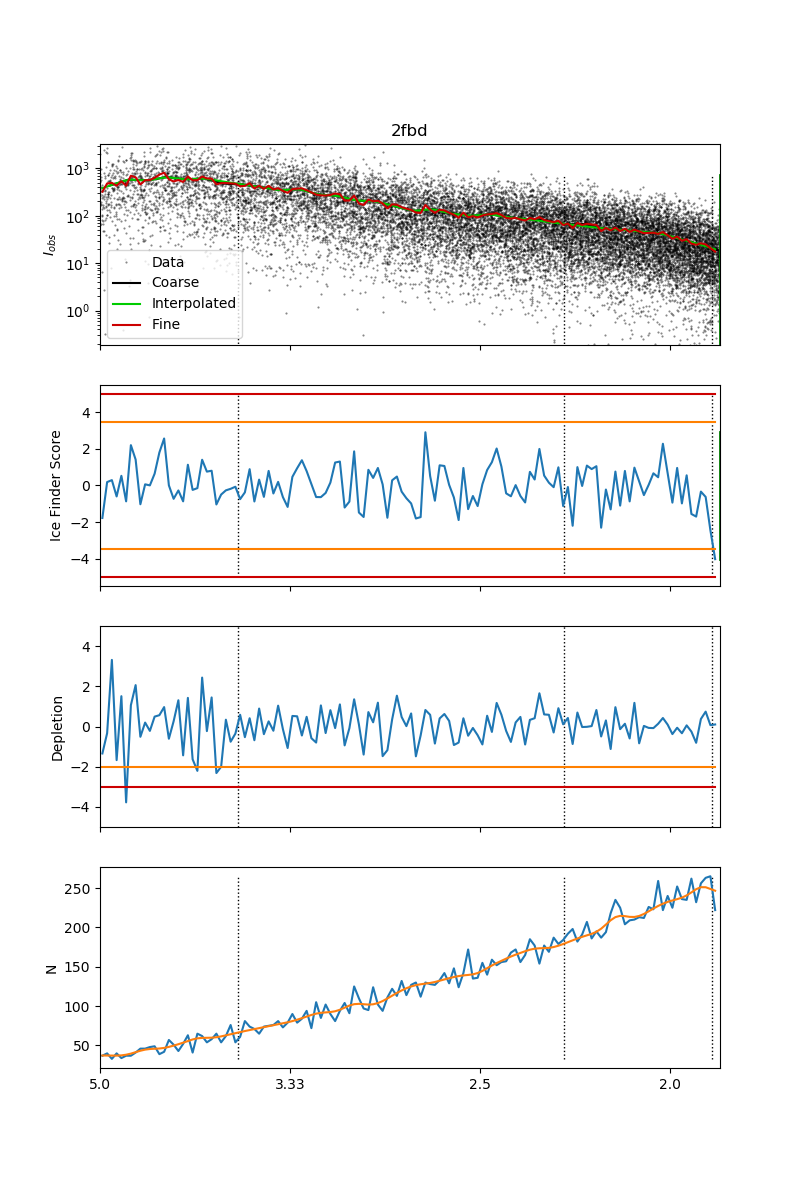

Supplement: Supplementary file 3 [file d-77-00540-sup3.zip › IceBiasingImages/2fbd.png]

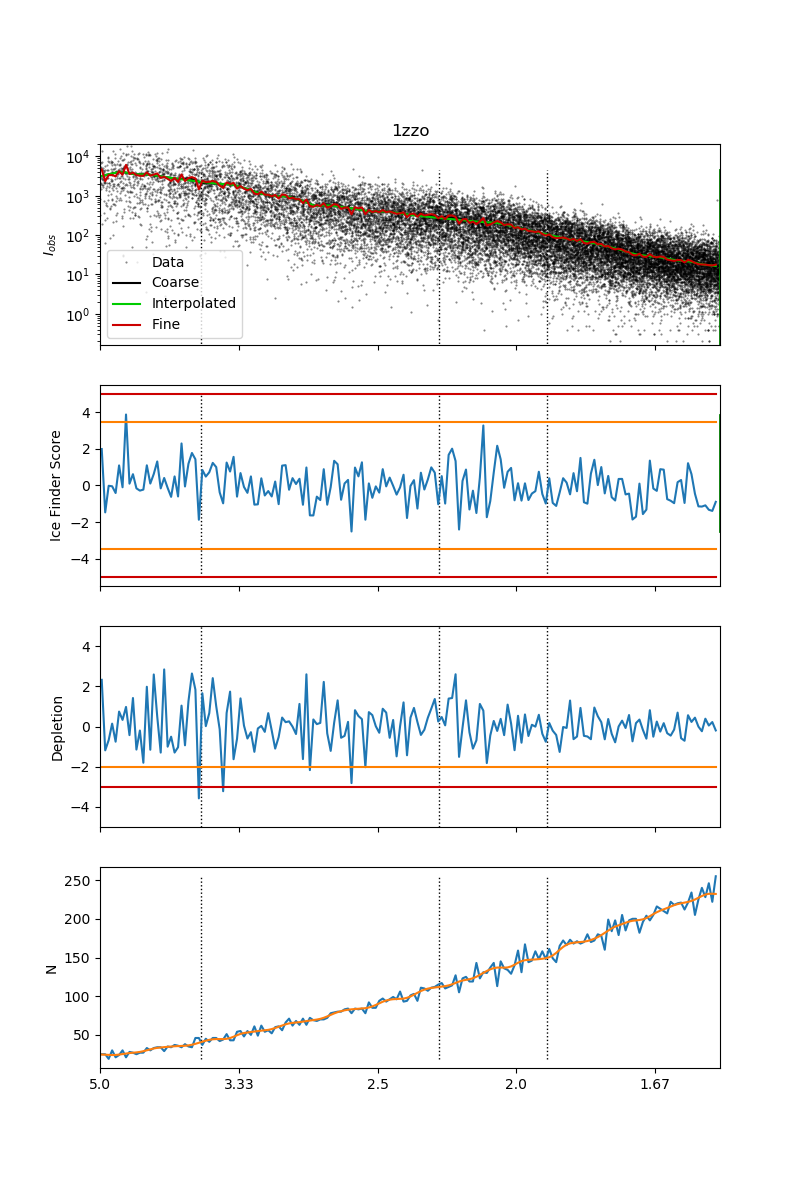

Supplement: Supplementary file 3 [file d-77-00540-sup3.zip › IceBiasingImages/1zzo.png]

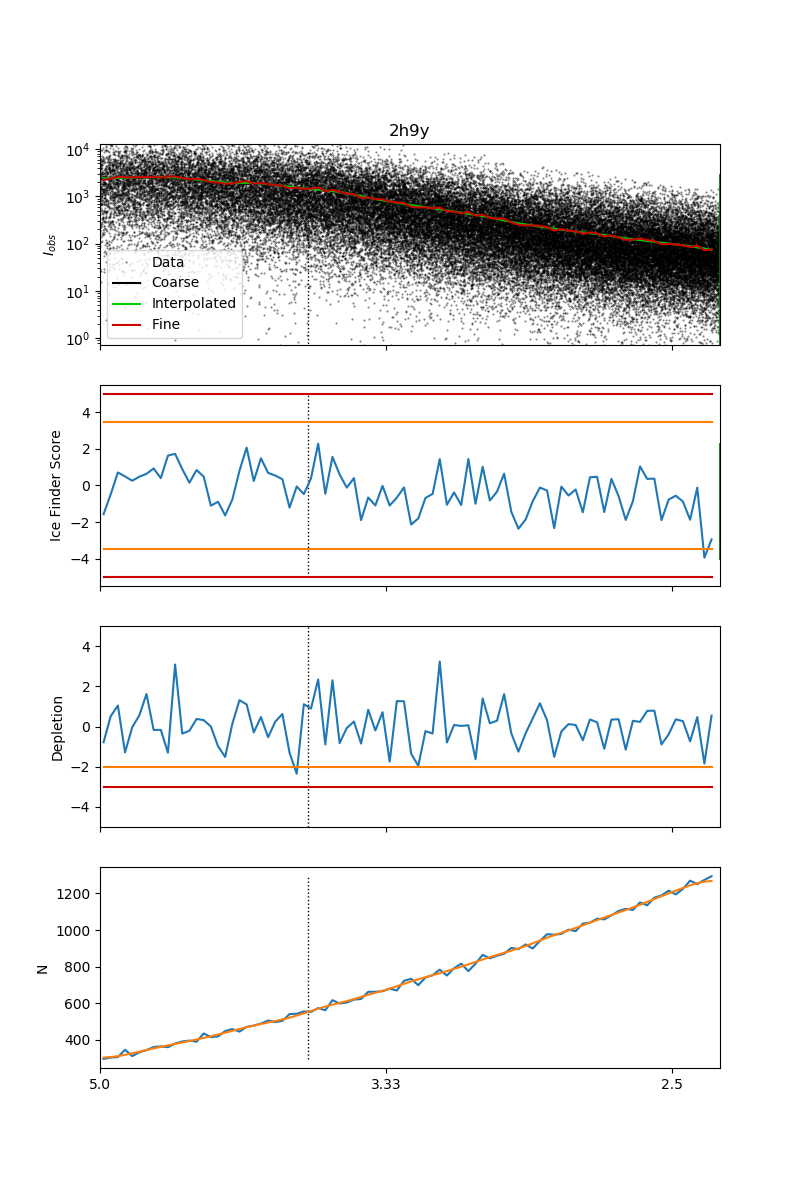

Supplement: Supplementary file 3 [file d-77-00540-sup3.zip › IceBiasingImages/2h9y.png]

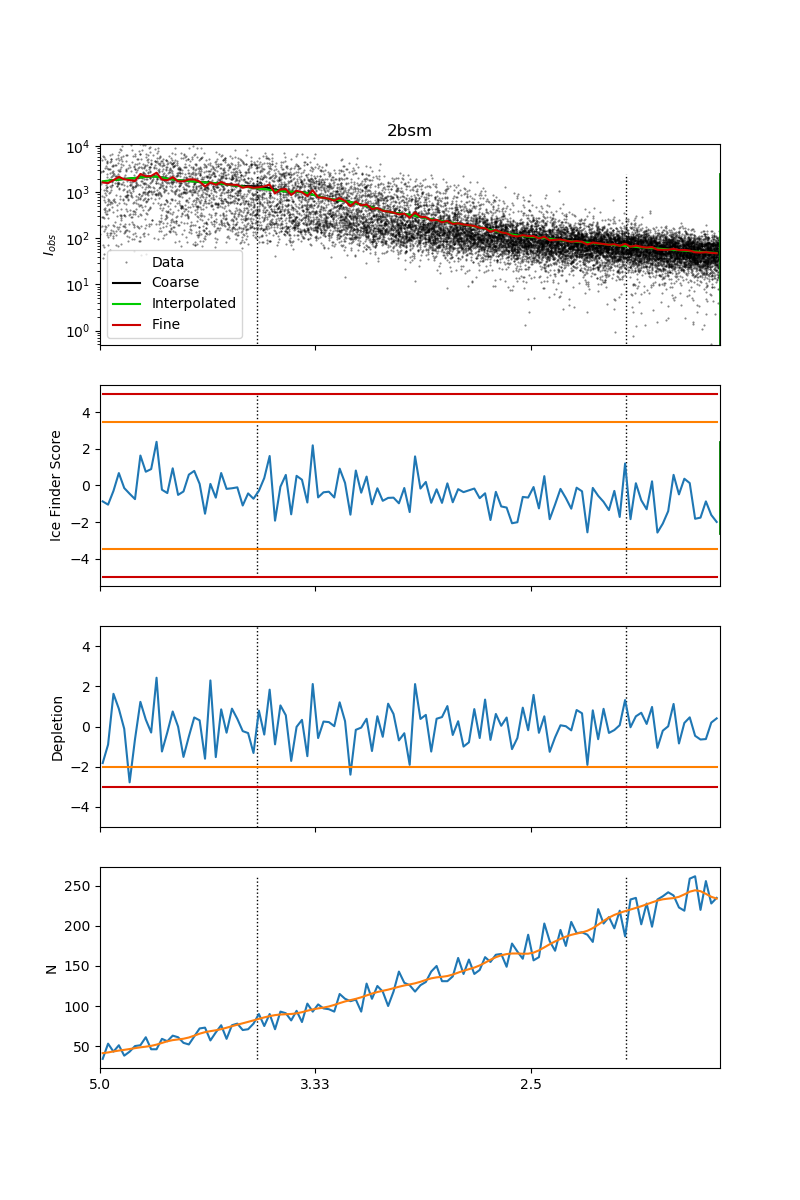

Supplement: Supplementary file 3 [file d-77-00540-sup3.zip › IceBiasingImages/2bsm.png]

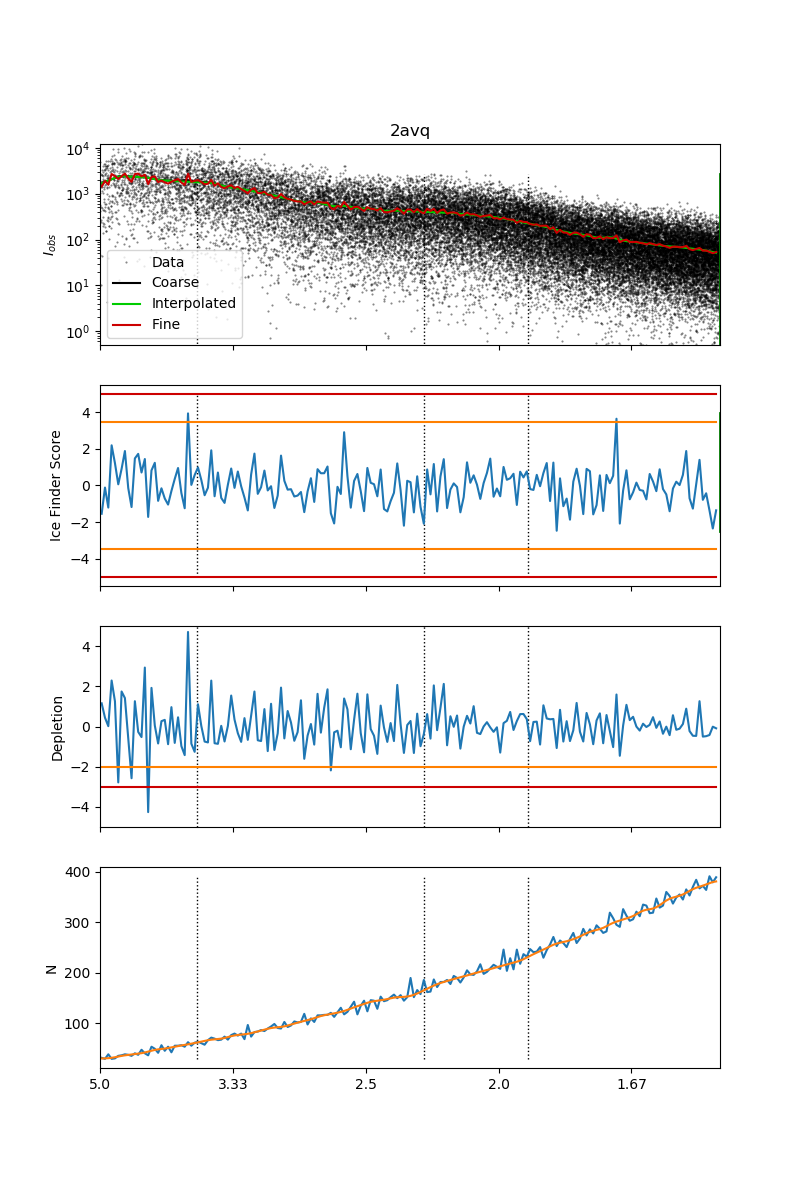

Supplement: Supplementary file 3 [file d-77-00540-sup3.zip › IceBiasingImages/2avq.png]

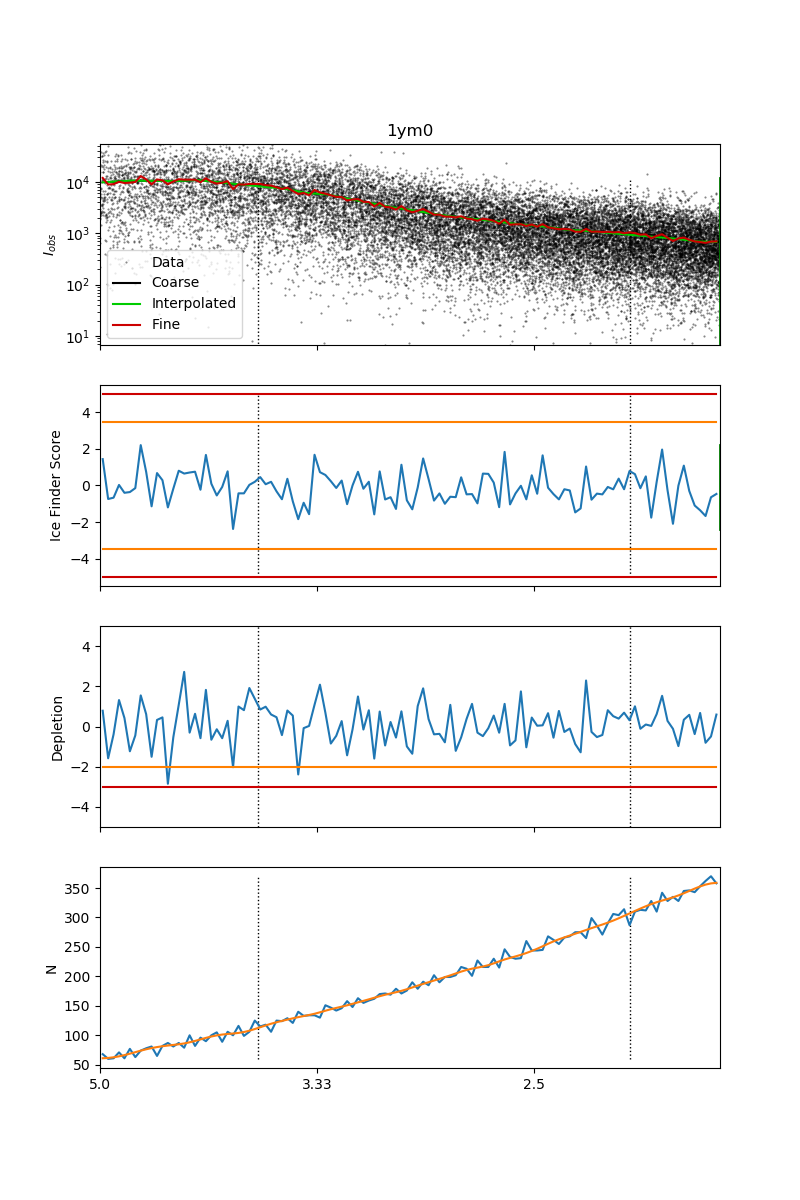

Supplement: Supplementary file 3 [file d-77-00540-sup3.zip › IceBiasingImages/1ym0.png]

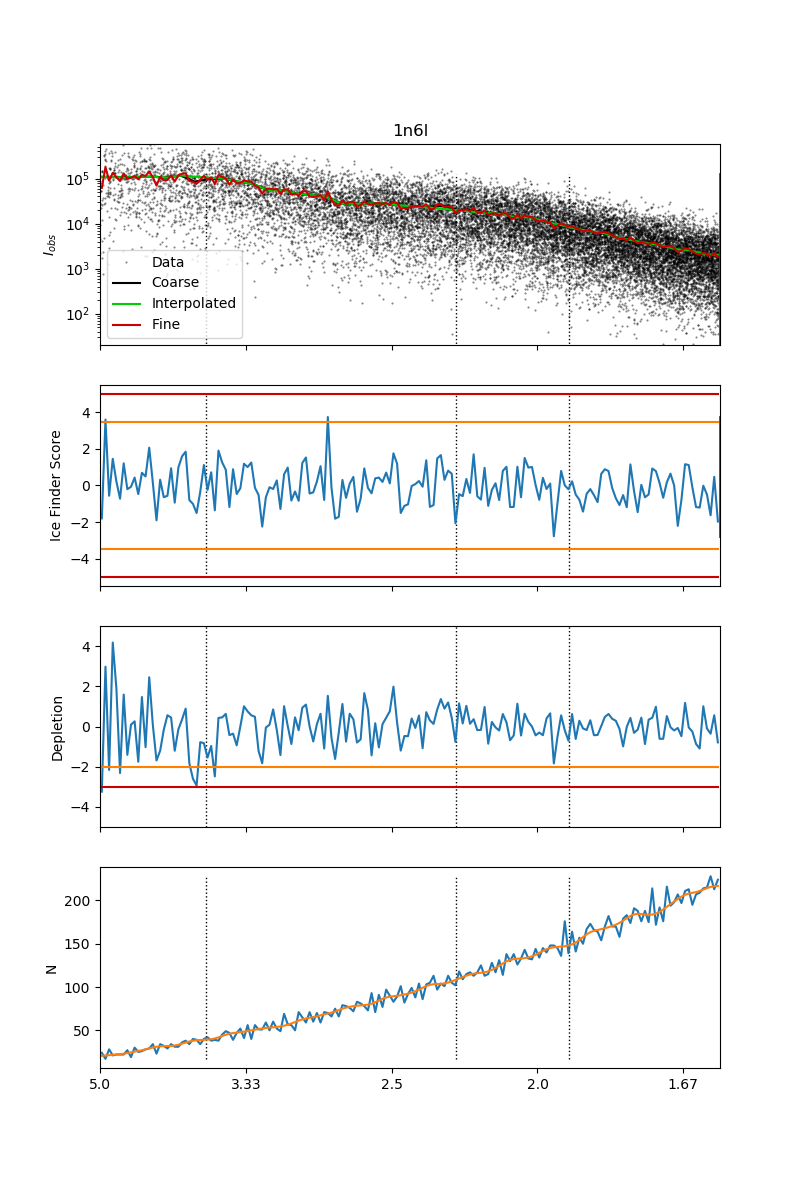

Supplement: Supplementary file 3 [file d-77-00540-sup3.zip › IceBiasingImages/1n6l.png]

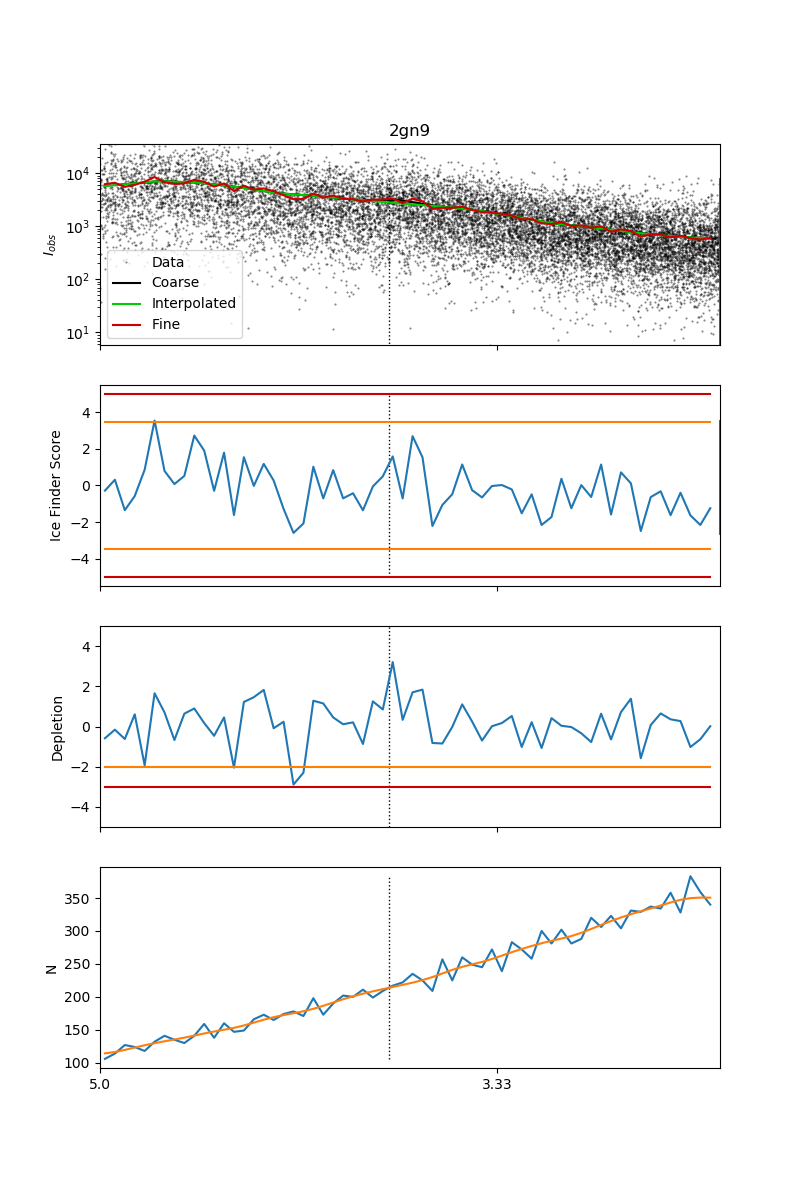

Supplement: Supplementary file 3 [file d-77-00540-sup3.zip › IceBiasingImages/2gn9.png]

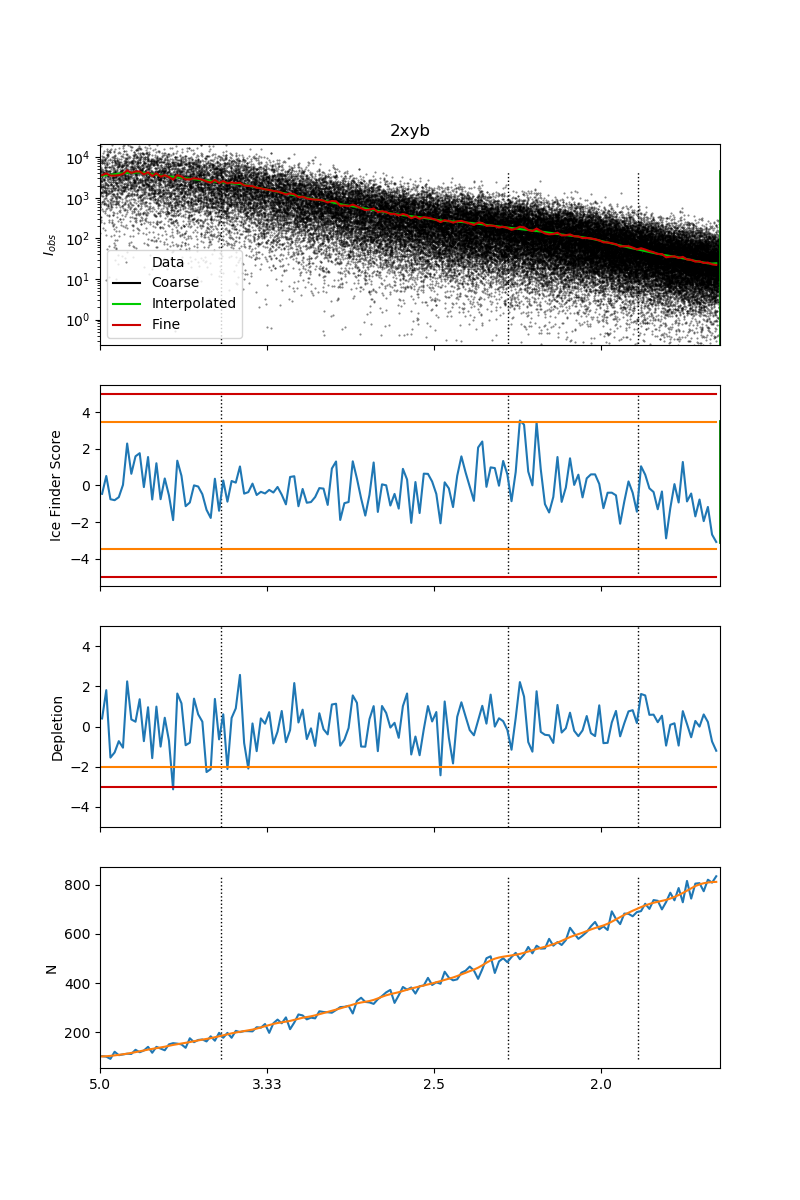

Supplement: Supplementary file 3 [file d-77-00540-sup3.zip › IceBiasingImages/2xyb.png]

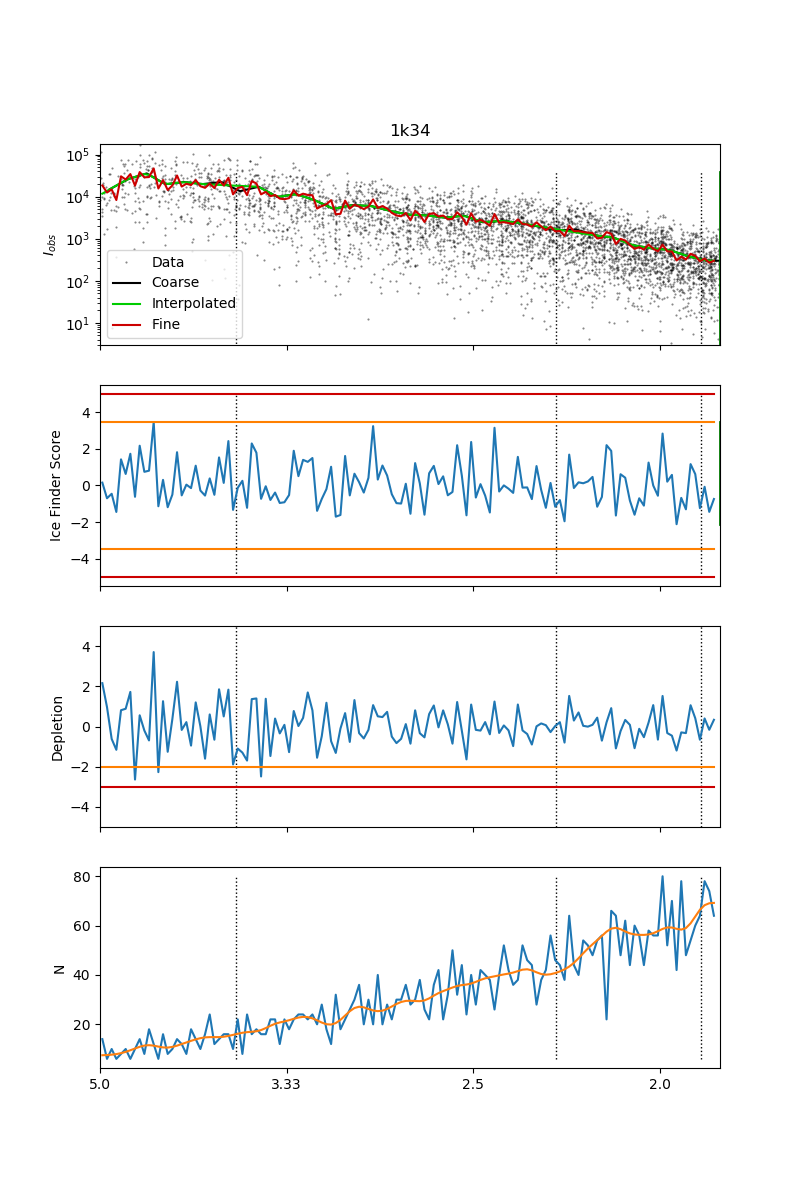

Supplement: Supplementary file 3 [file d-77-00540-sup3.zip › IceBiasingImages/1k34.png]

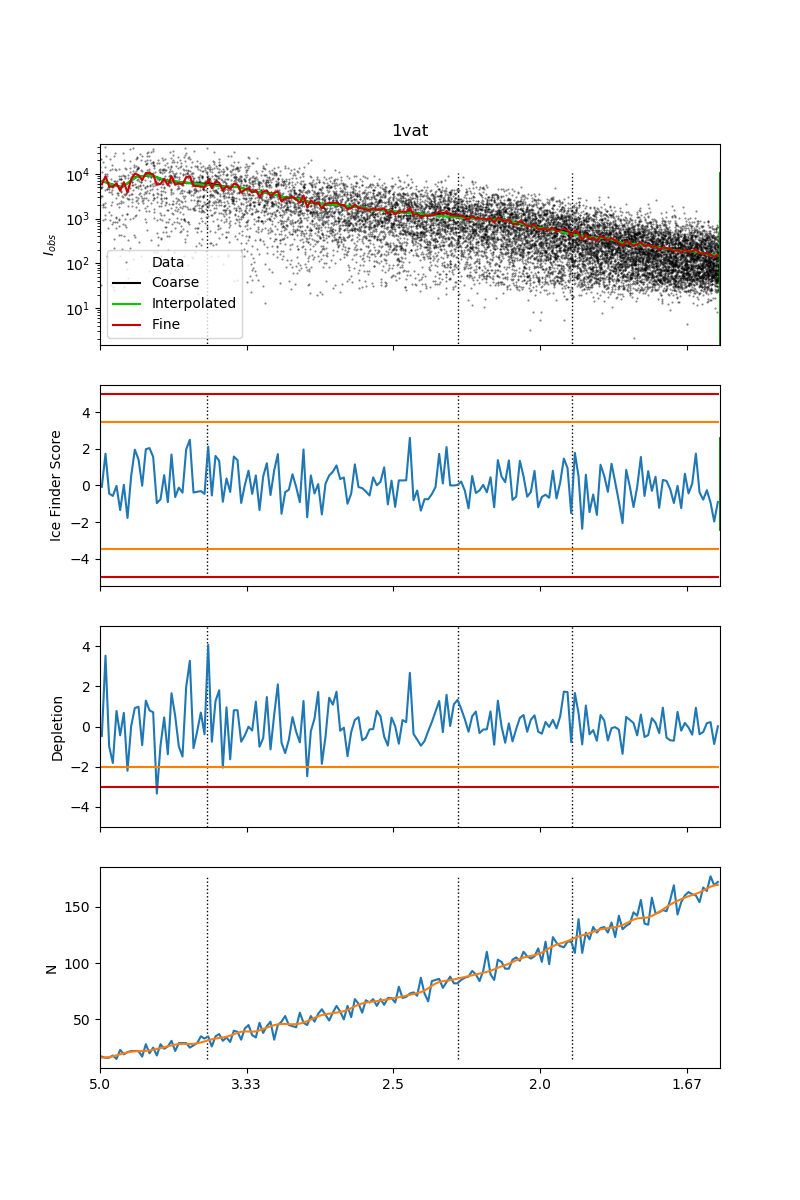

Supplement: Supplementary file 3 [file d-77-00540-sup3.zip › IceBiasingImages/1vat.png]

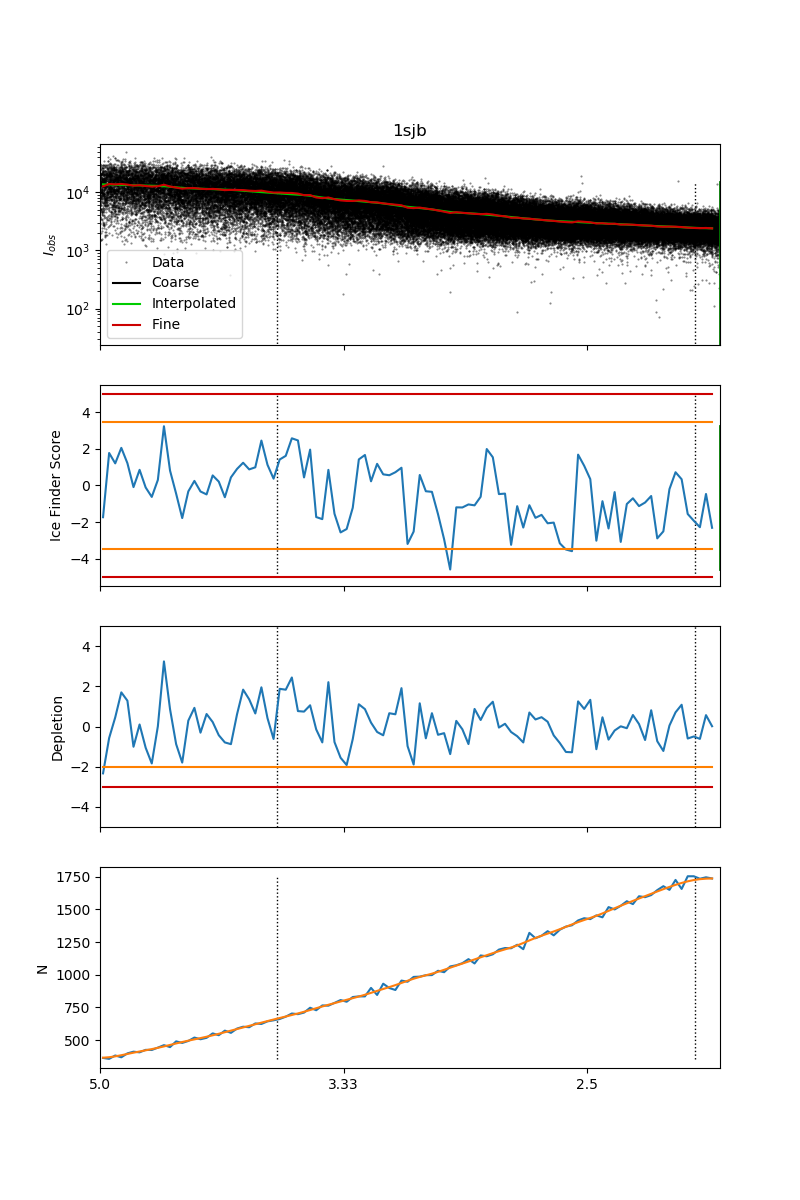

Supplement: Supplementary file 3 [file d-77-00540-sup3.zip › IceBiasingImages/1sjb.png]

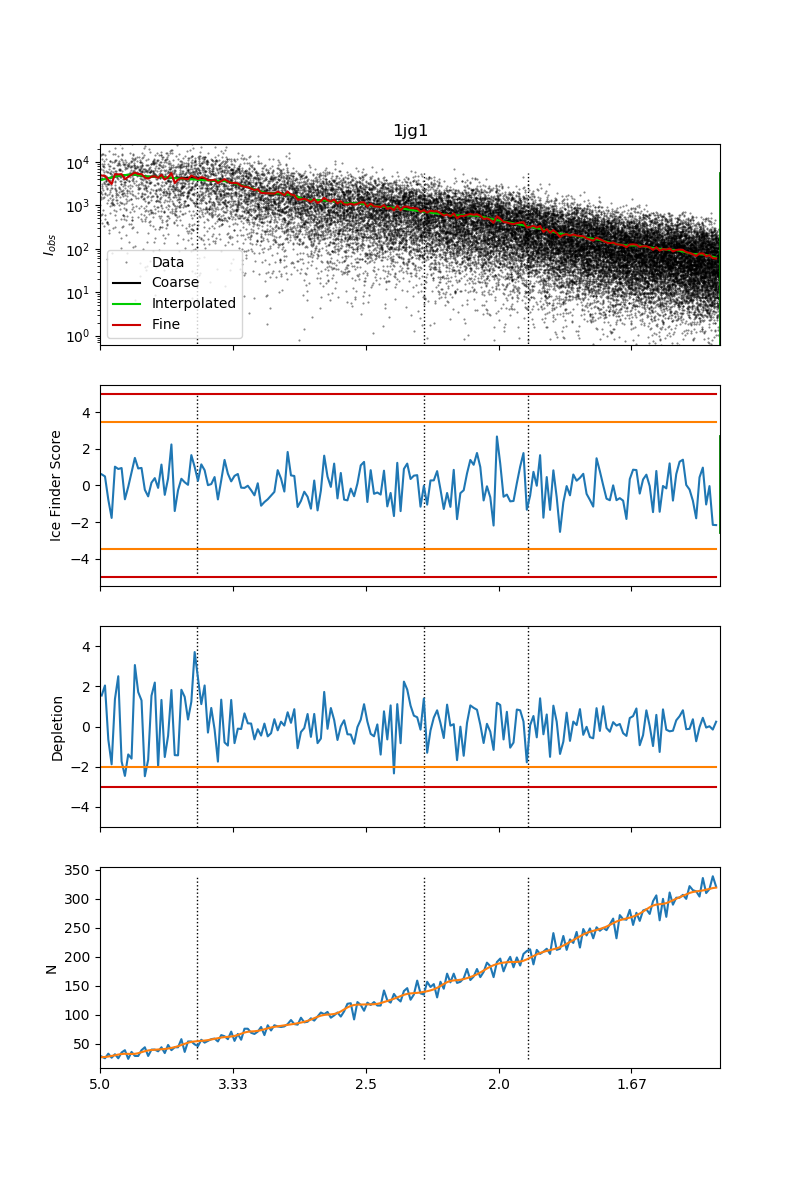

Supplement: Supplementary file 3 [file d-77-00540-sup3.zip › IceBiasingImages/1jg1.png]

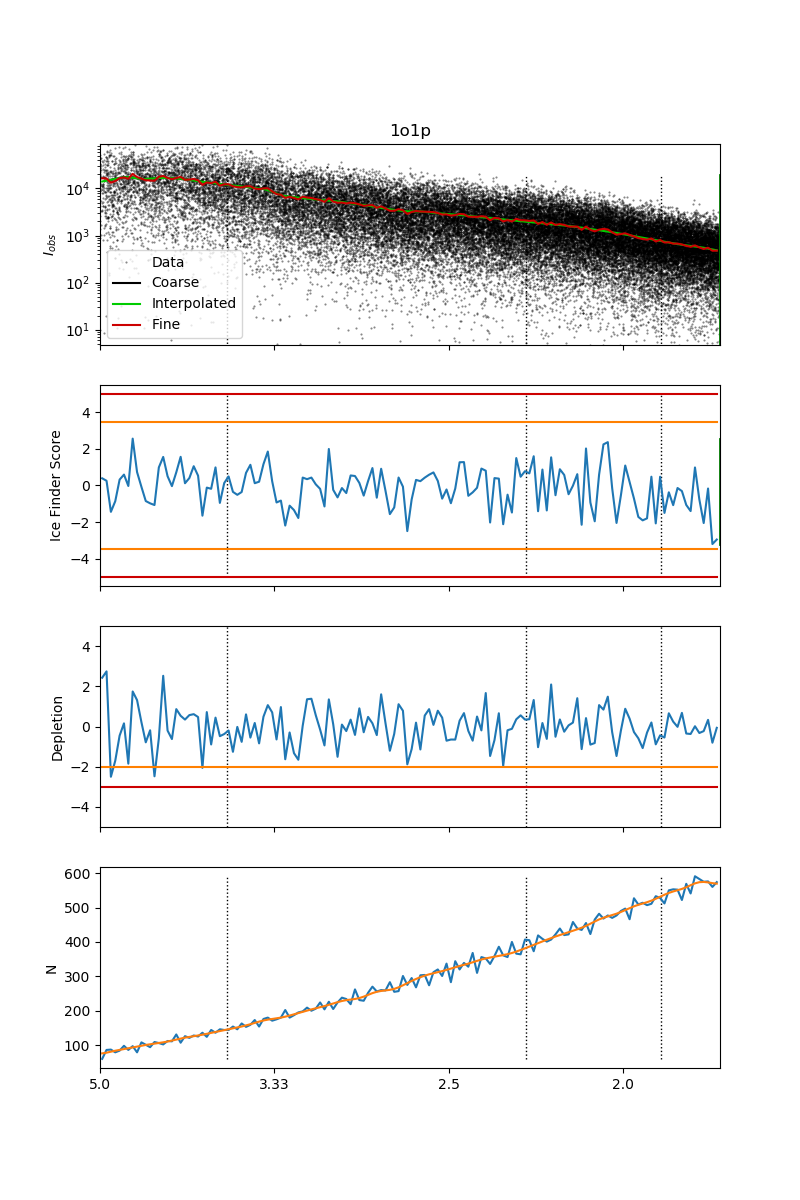

Supplement: Supplementary file 3 [file d-77-00540-sup3.zip › IceBiasingImages/1o1p.png]

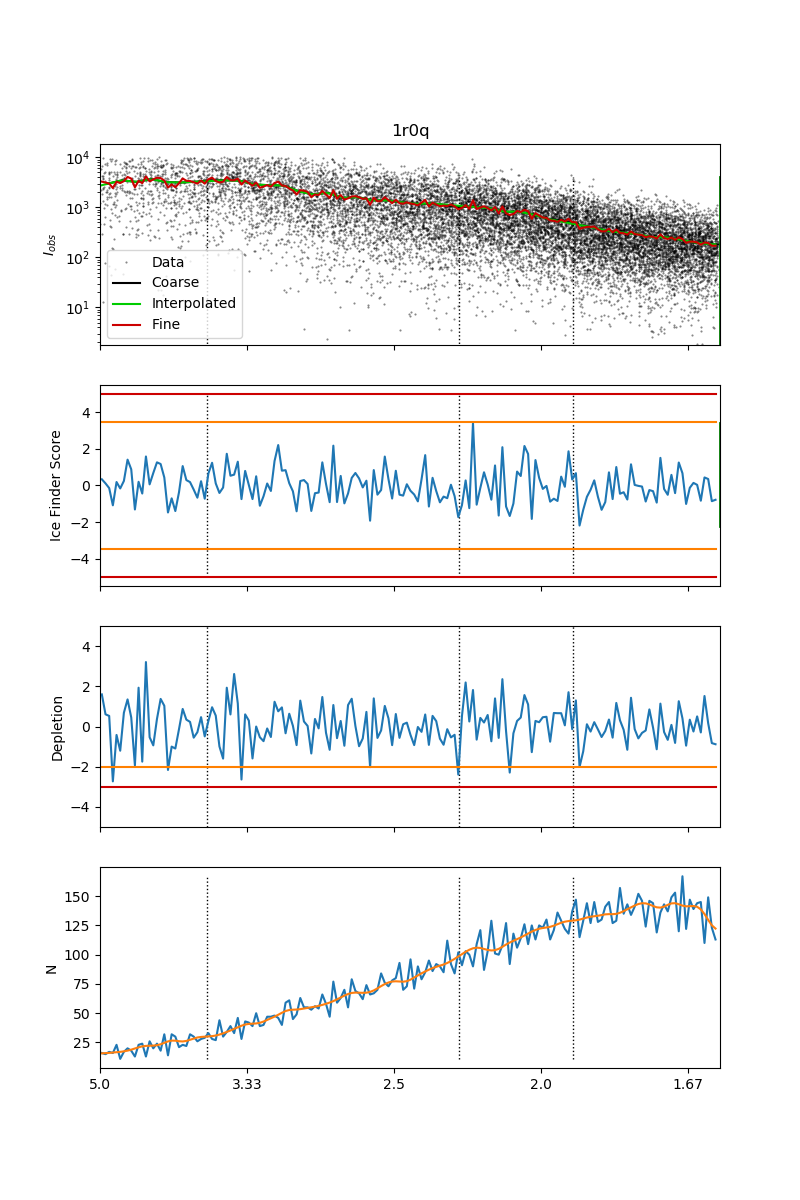

Supplement: Supplementary file 3 [file d-77-00540-sup3.zip › IceBiasingImages/1r0q.png]

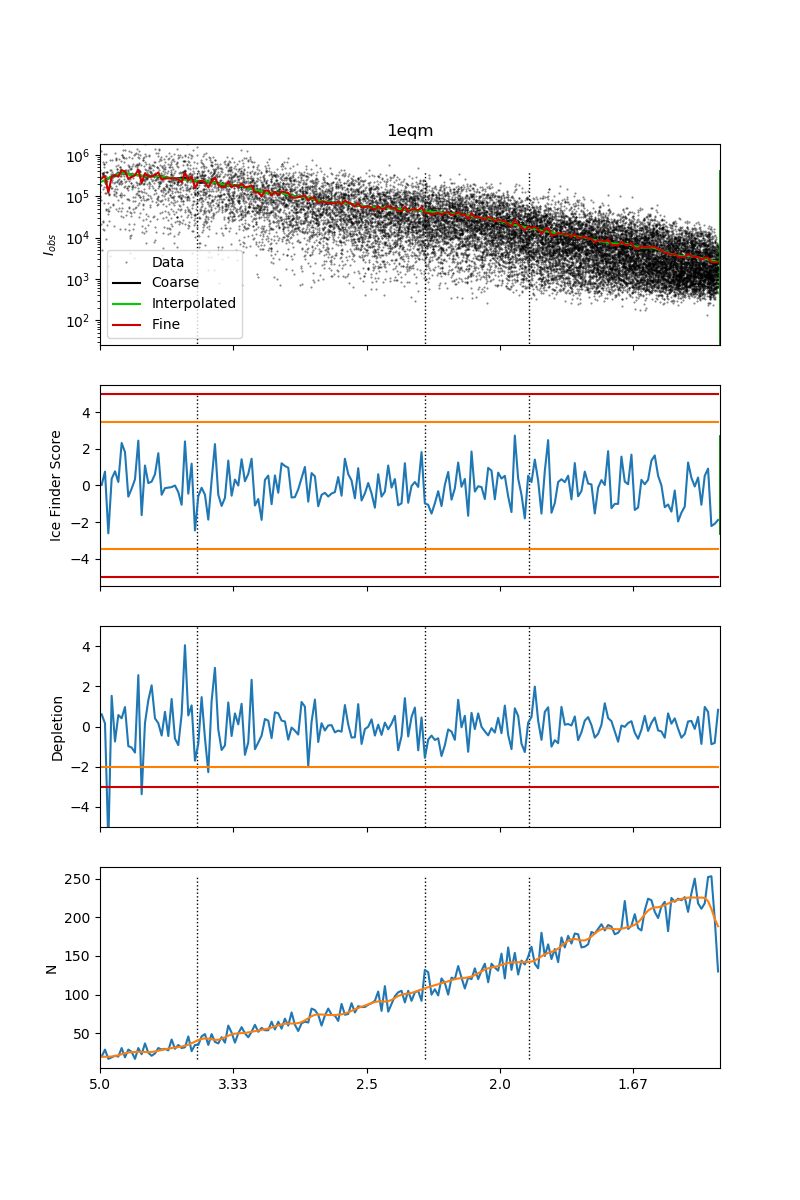

Supplement: Supplementary file 3 [file d-77-00540-sup3.zip › IceBiasingImages/1eqm.png]

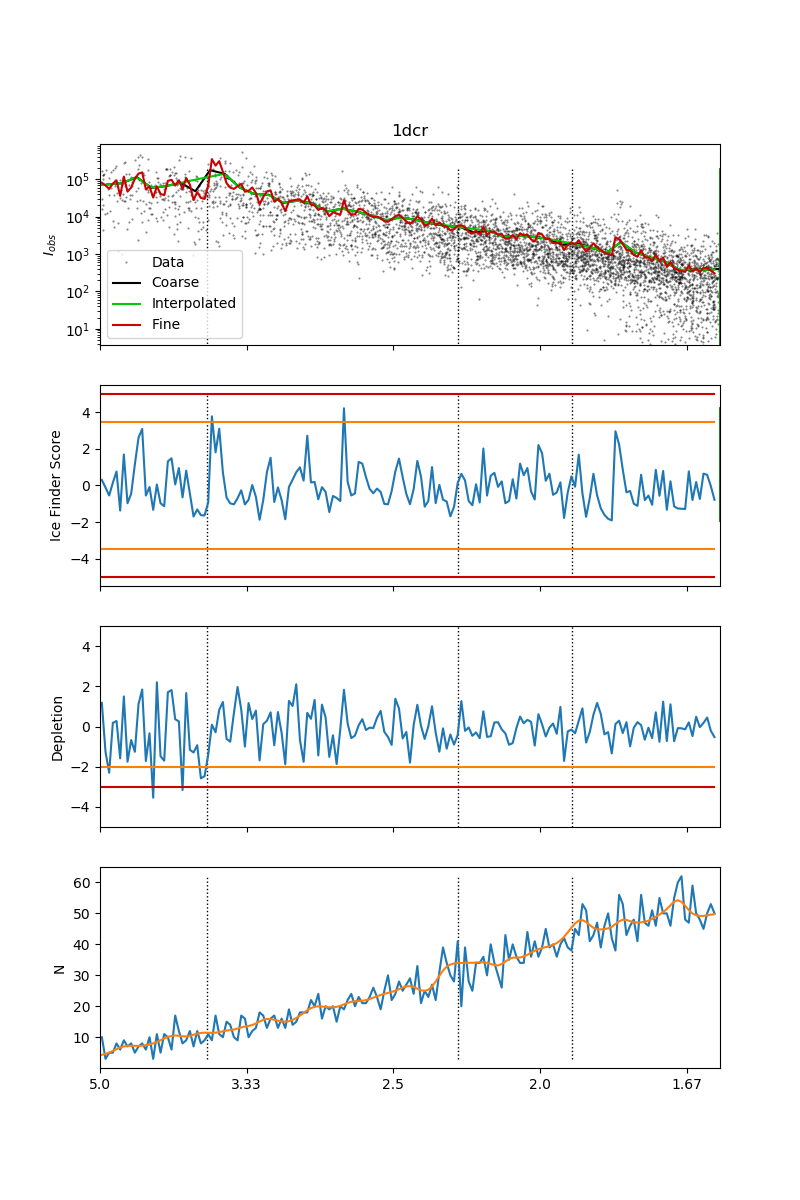

Supplement: Supplementary file 3 [file d-77-00540-sup3.zip › IceBiasingImages/1dcr.png]

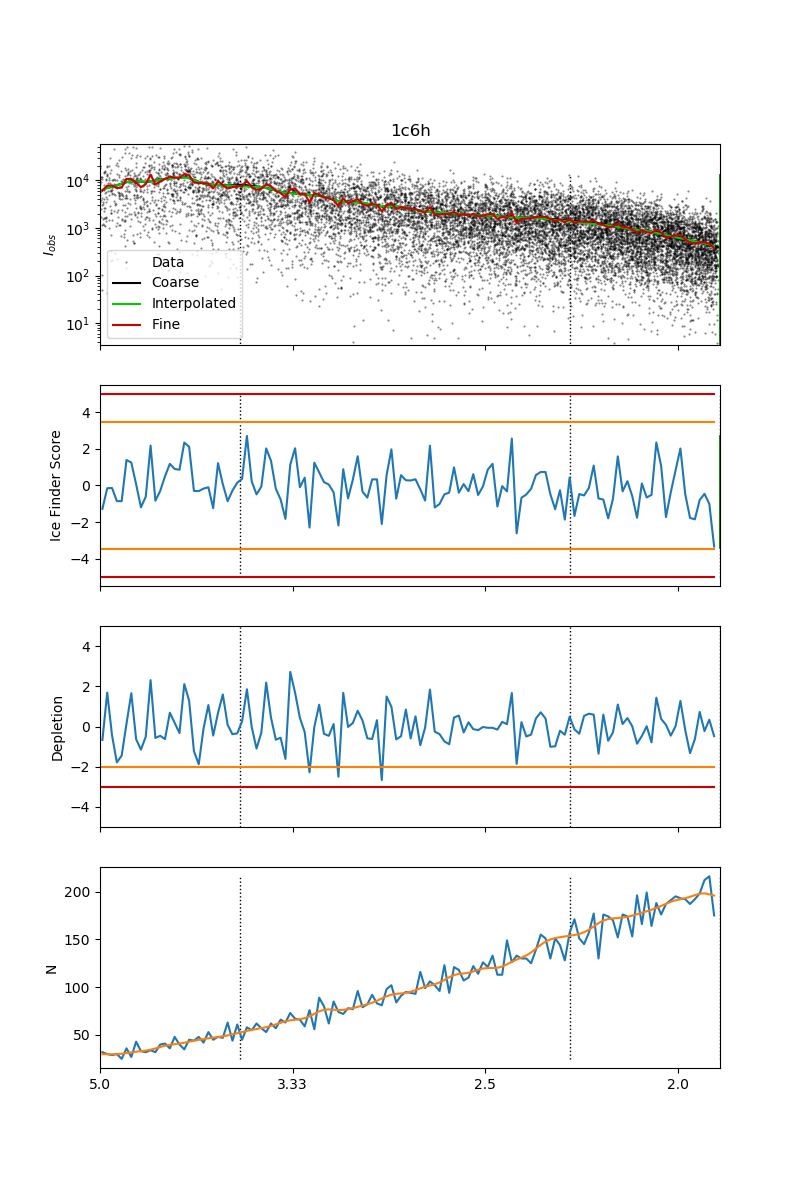

Supplement: Supplementary file 3 [file d-77-00540-sup3.zip › IceBiasingImages/1c6h.png]

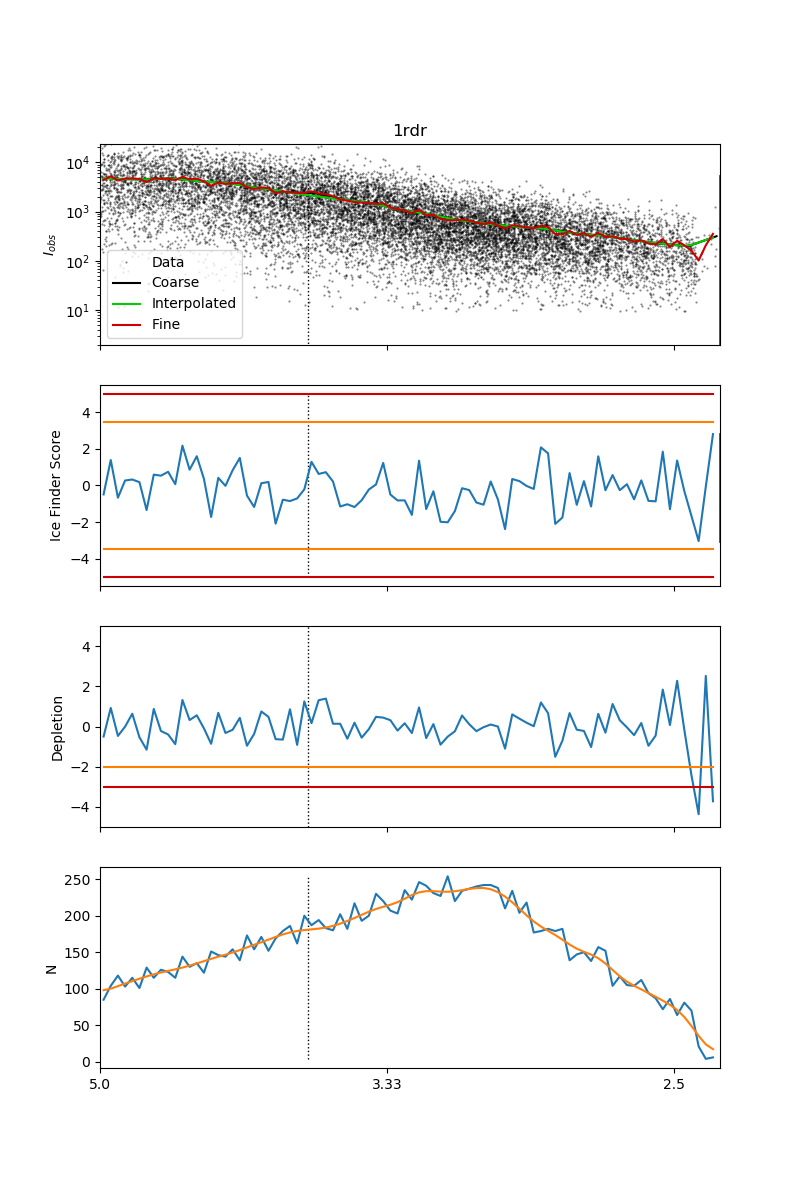

Supplement: Supplementary file 3 [file d-77-00540-sup3.zip › IceBiasingImages/1rdr.png]

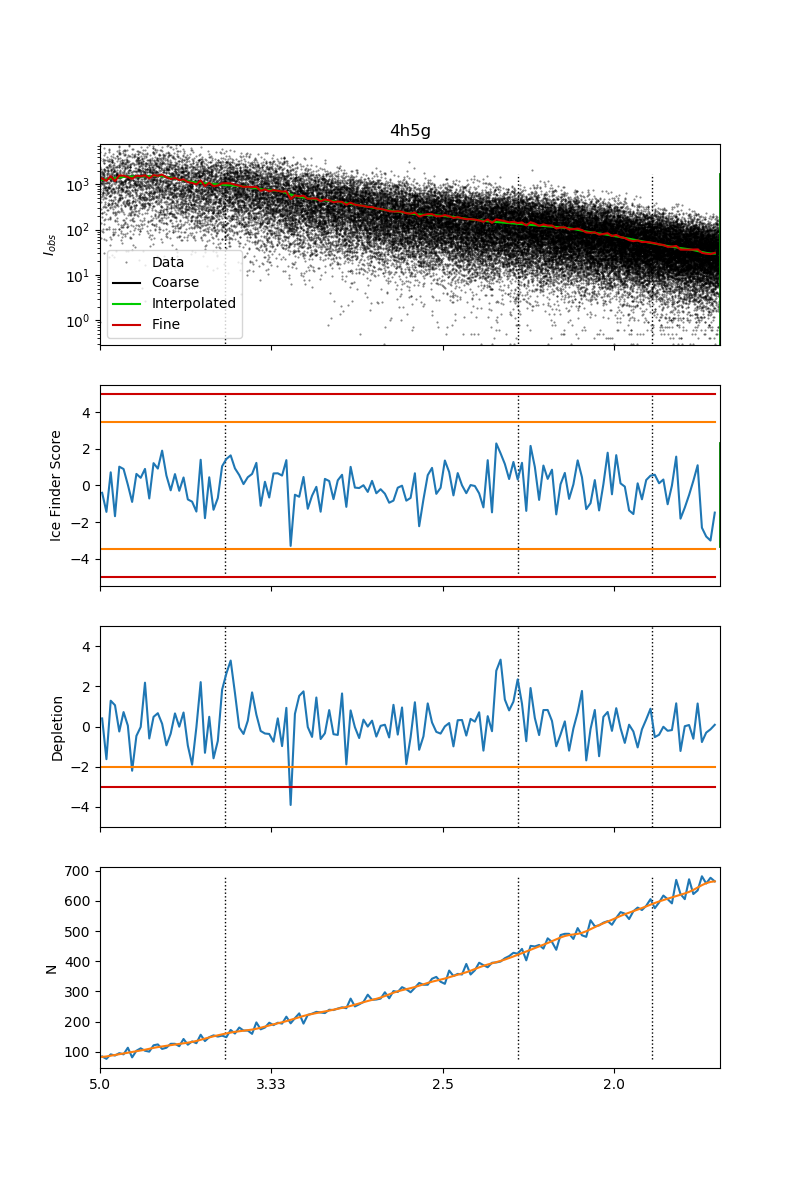

Supplement: Supplementary file 3 [file d-77-00540-sup3.zip › IceBiasingImages/4h5g.png]

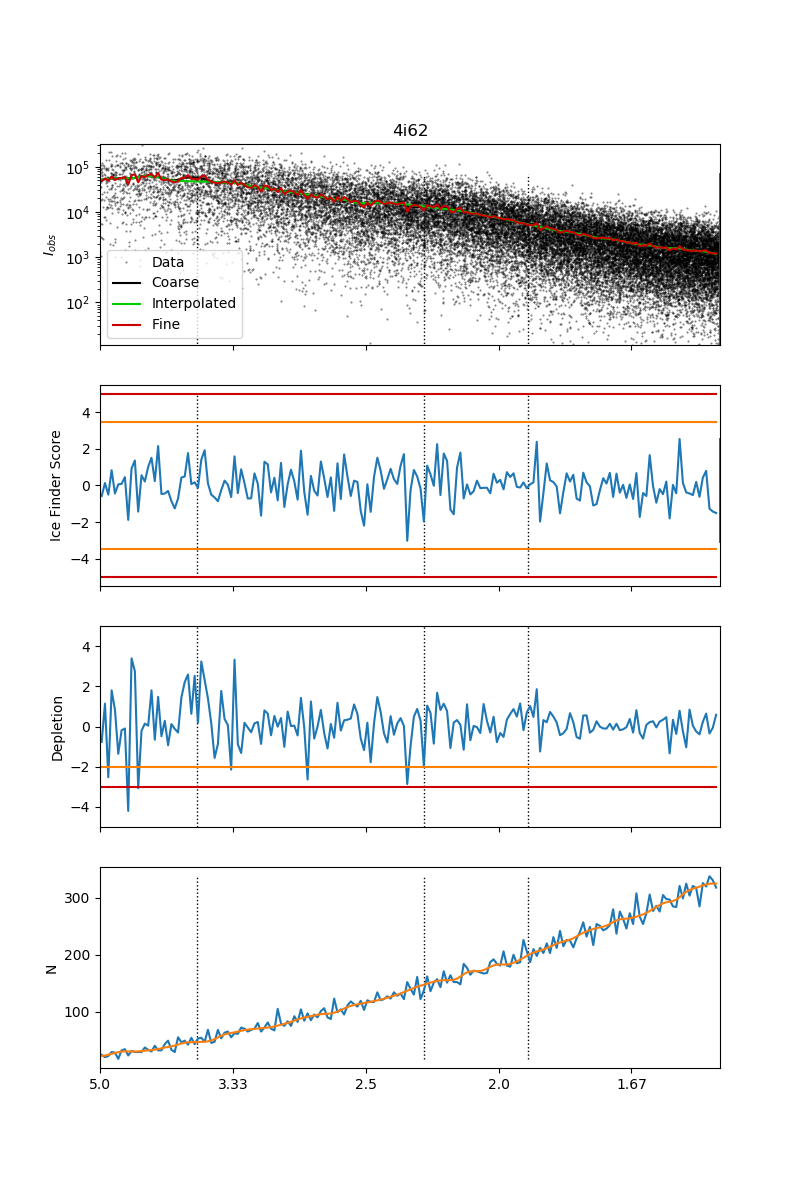

Supplement: Supplementary file 3 [file d-77-00540-sup3.zip › IceBiasingImages/4i62.png]

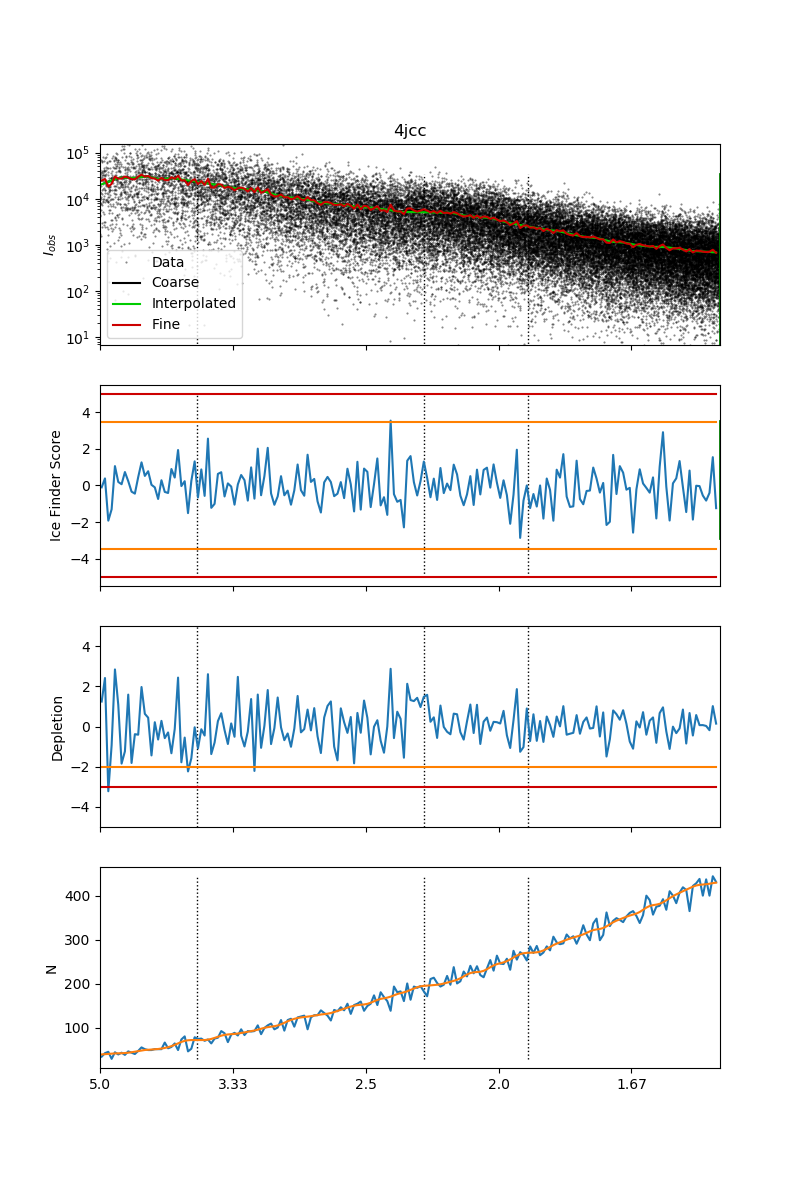

Supplement: Supplementary file 3 [file d-77-00540-sup3.zip › IceBiasingImages/4jcc.png]

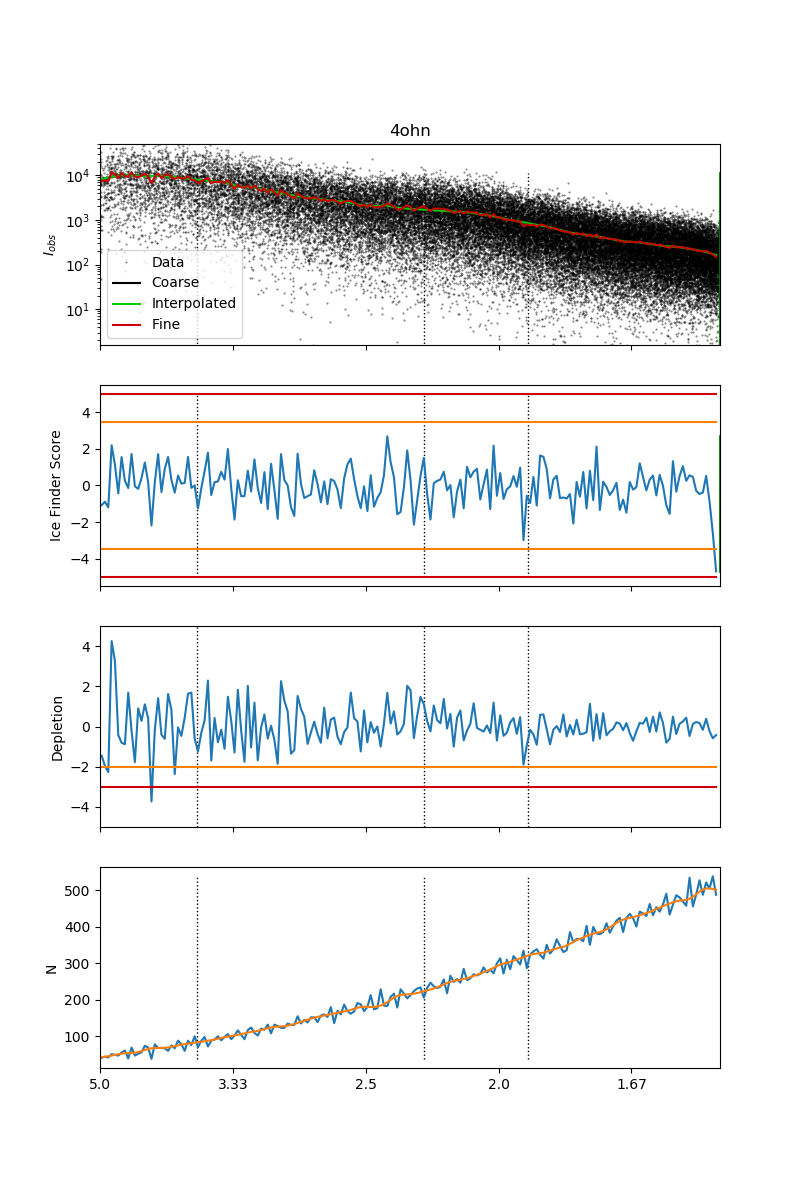

Supplement: Supplementary file 3 [file d-77-00540-sup3.zip › IceBiasingImages/4ohn.png]

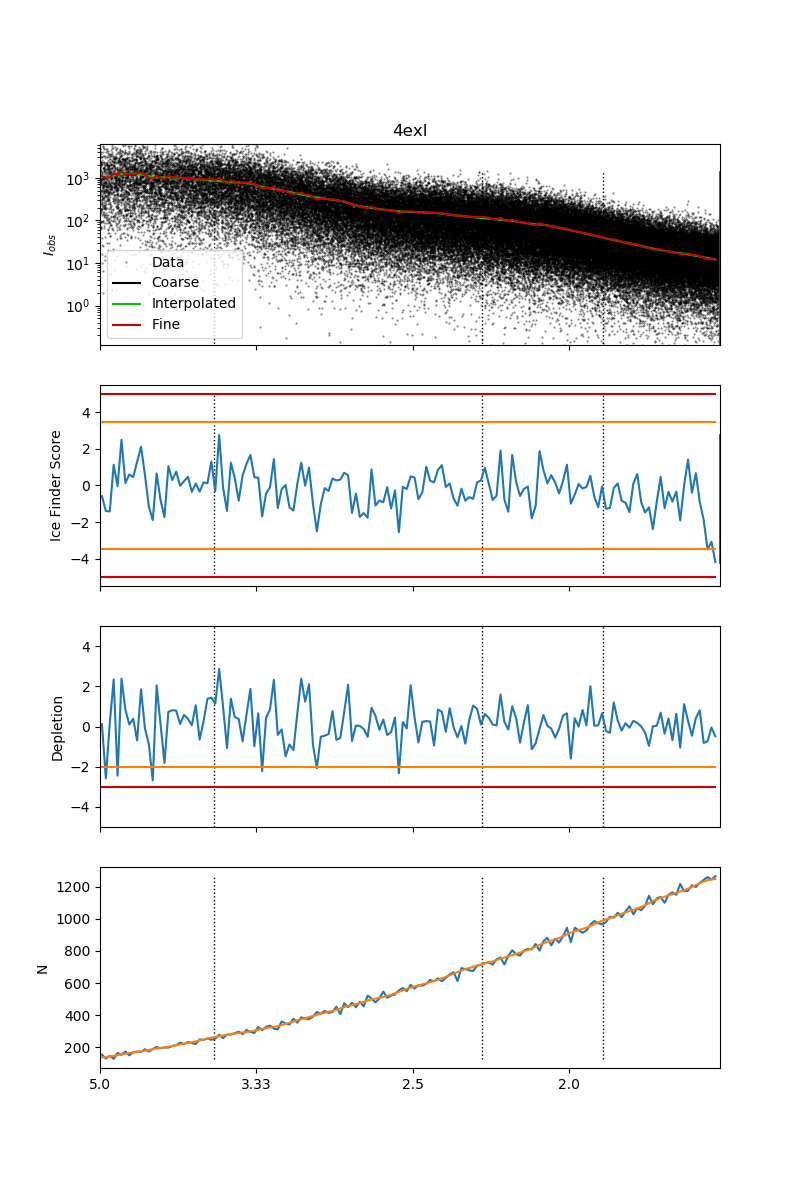

Supplement: Supplementary file 3 [file d-77-00540-sup3.zip › IceBiasingImages/4exl.png]

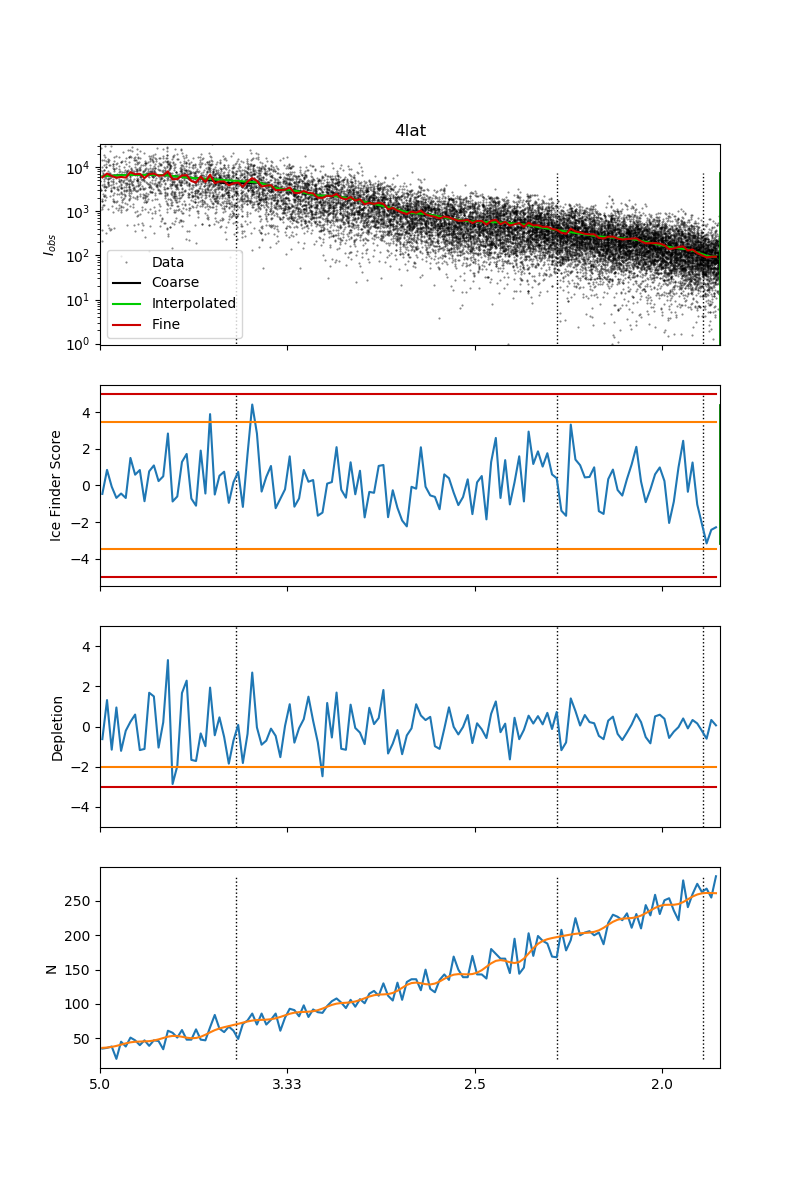

Supplement: Supplementary file 3 [file d-77-00540-sup3.zip › IceBiasingImages/4lat.png]

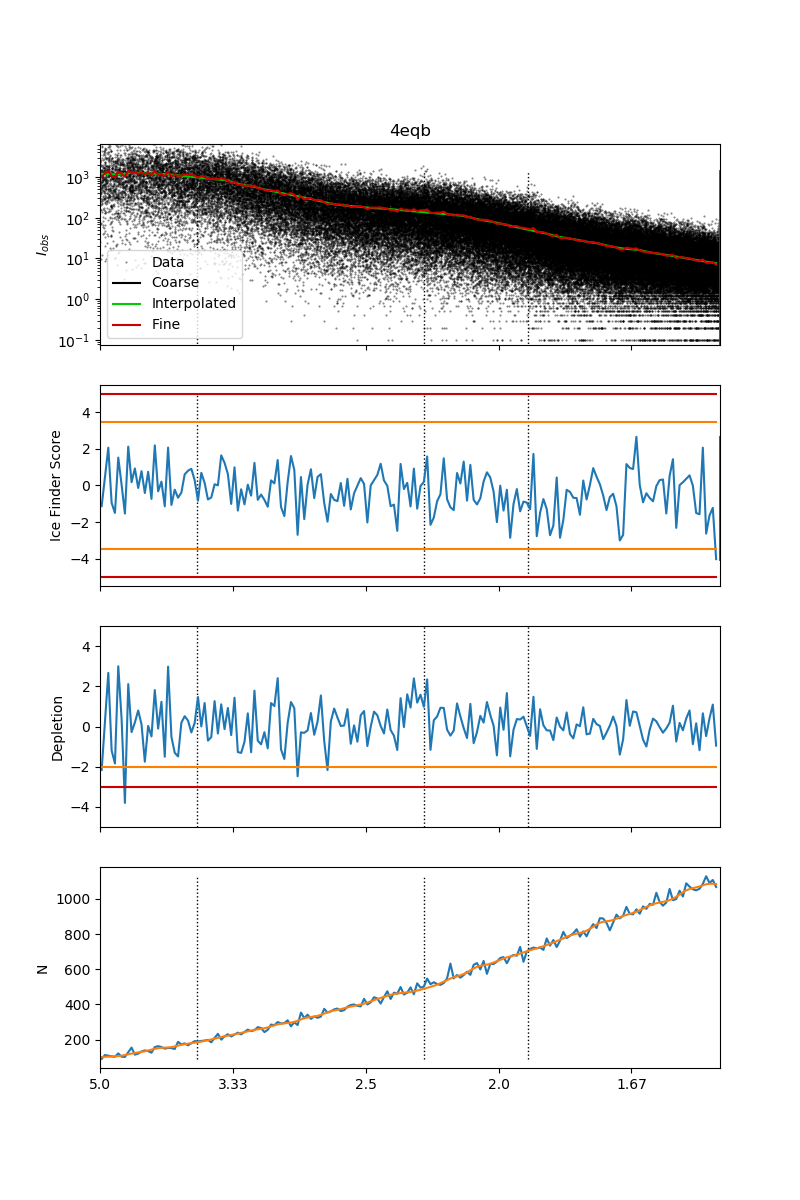

Supplement: Supplementary file 3 [file d-77-00540-sup3.zip › IceBiasingImages/4eqb.png]

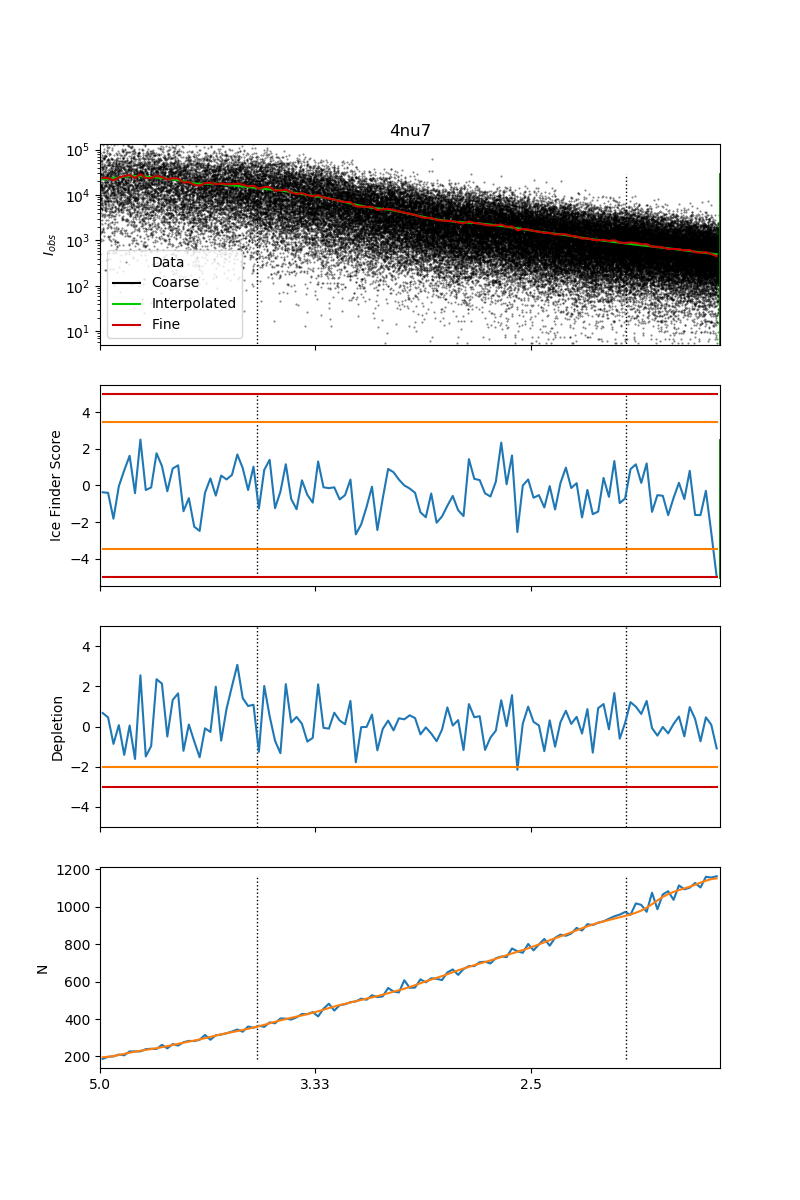

Supplement: Supplementary file 3 [file d-77-00540-sup3.zip › IceBiasingImages/4nu7.png]

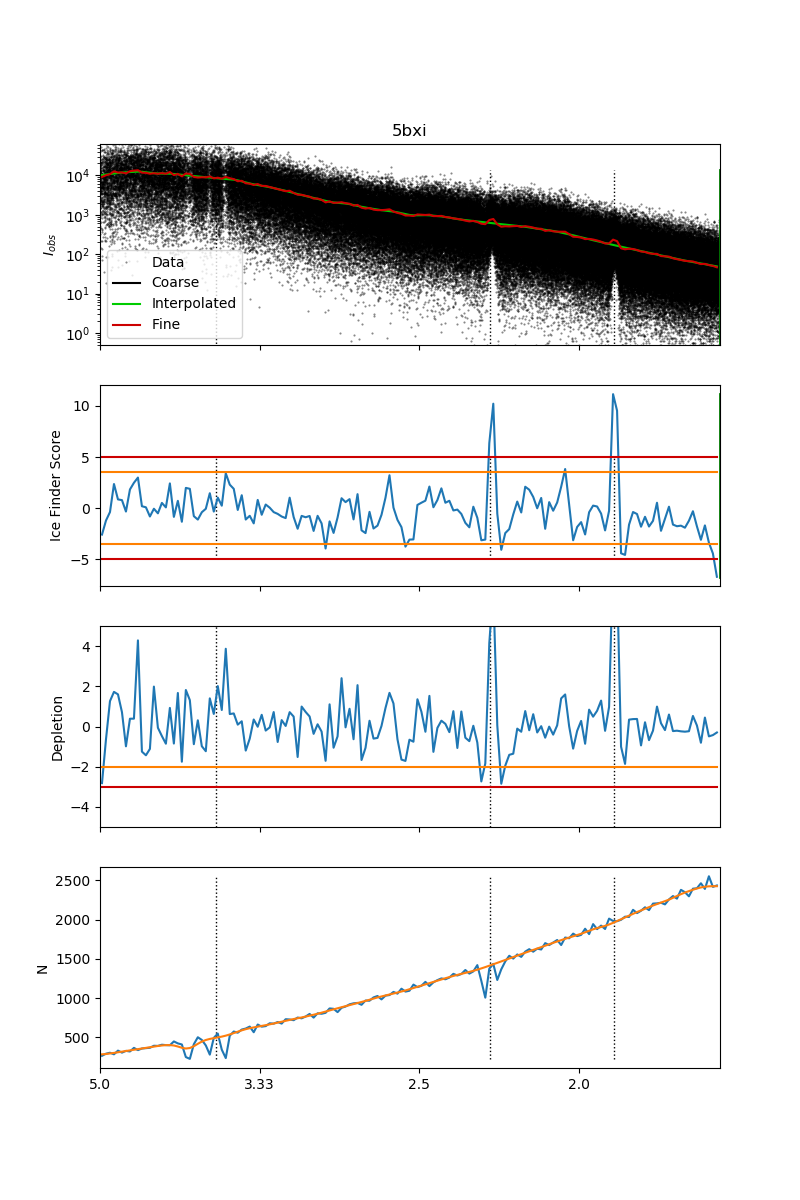

Supplement: Supplementary file 3 [file d-77-00540-sup3.zip › IceBiasingImages/5bxi.png]

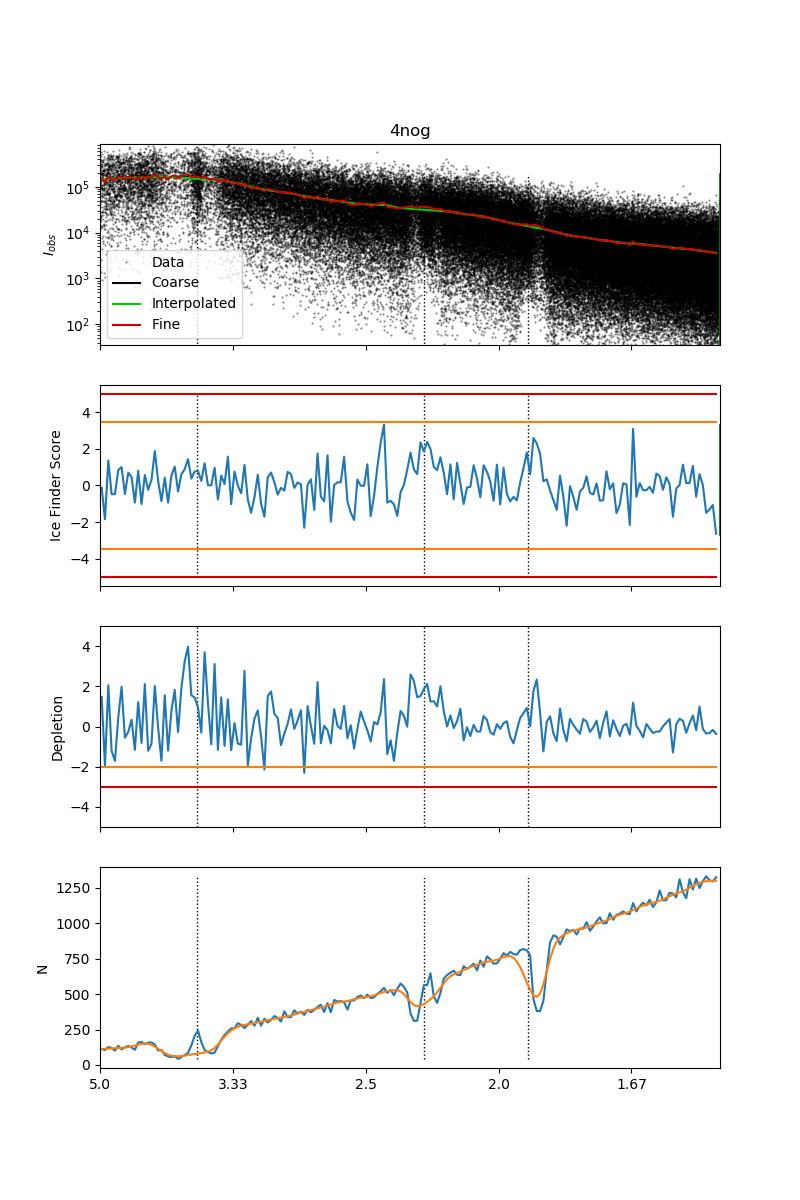

Supplement: Supplementary file 3 [file d-77-00540-sup3.zip › IceBiasingImages/4nog.png]

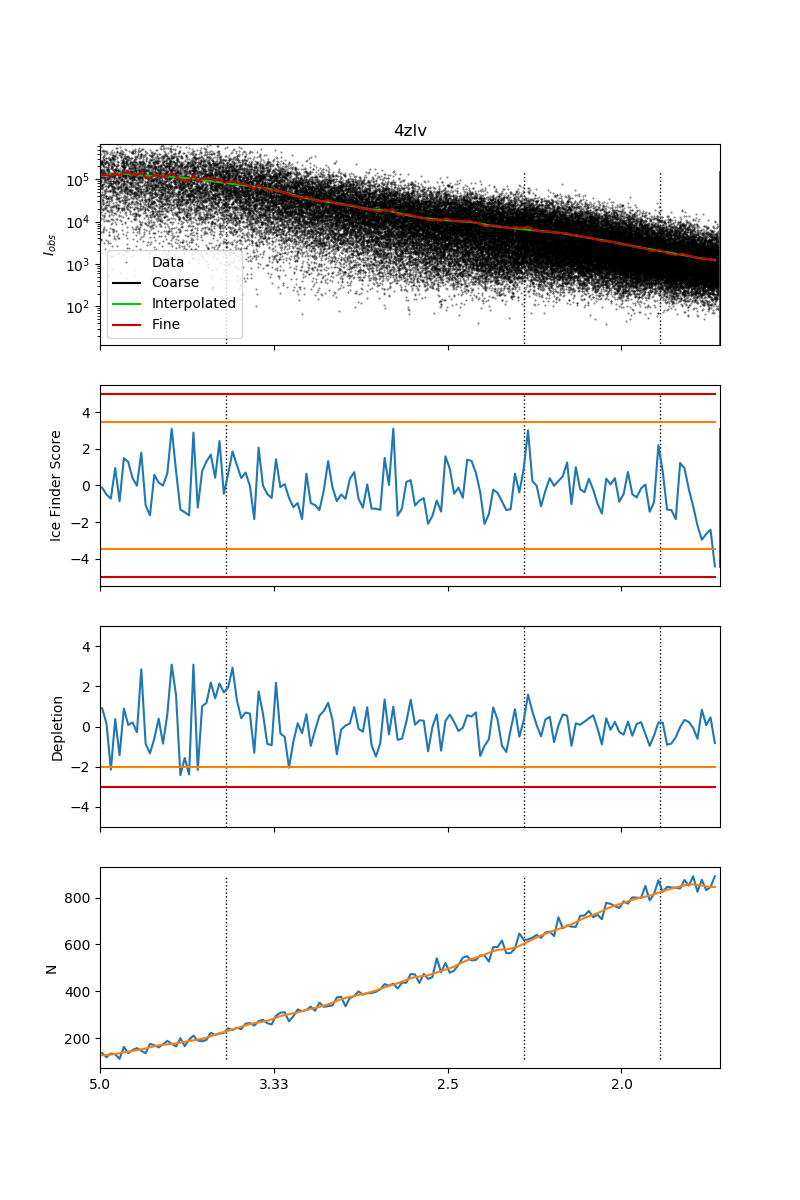

Supplement: Supplementary file 3 [file d-77-00540-sup3.zip › IceBiasingImages/4zlv.png]

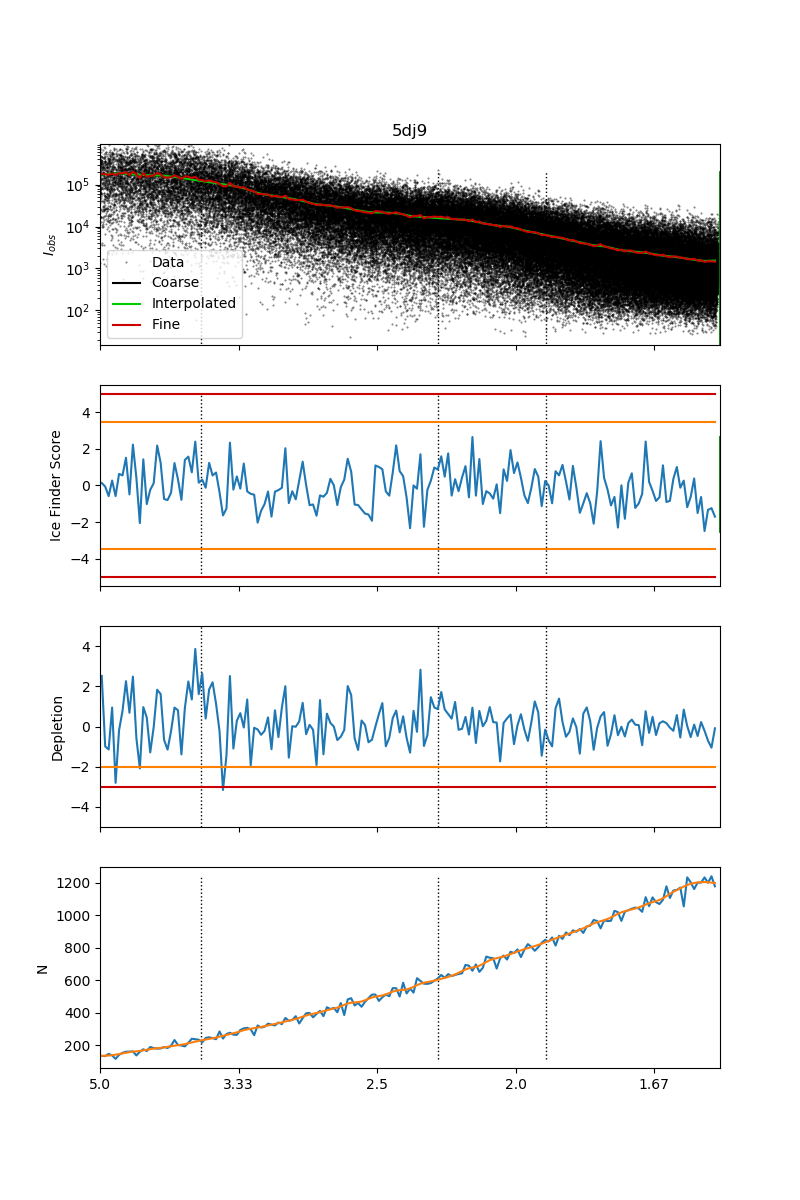

Supplement: Supplementary file 3 [file d-77-00540-sup3.zip › IceBiasingImages/5dj9.png]

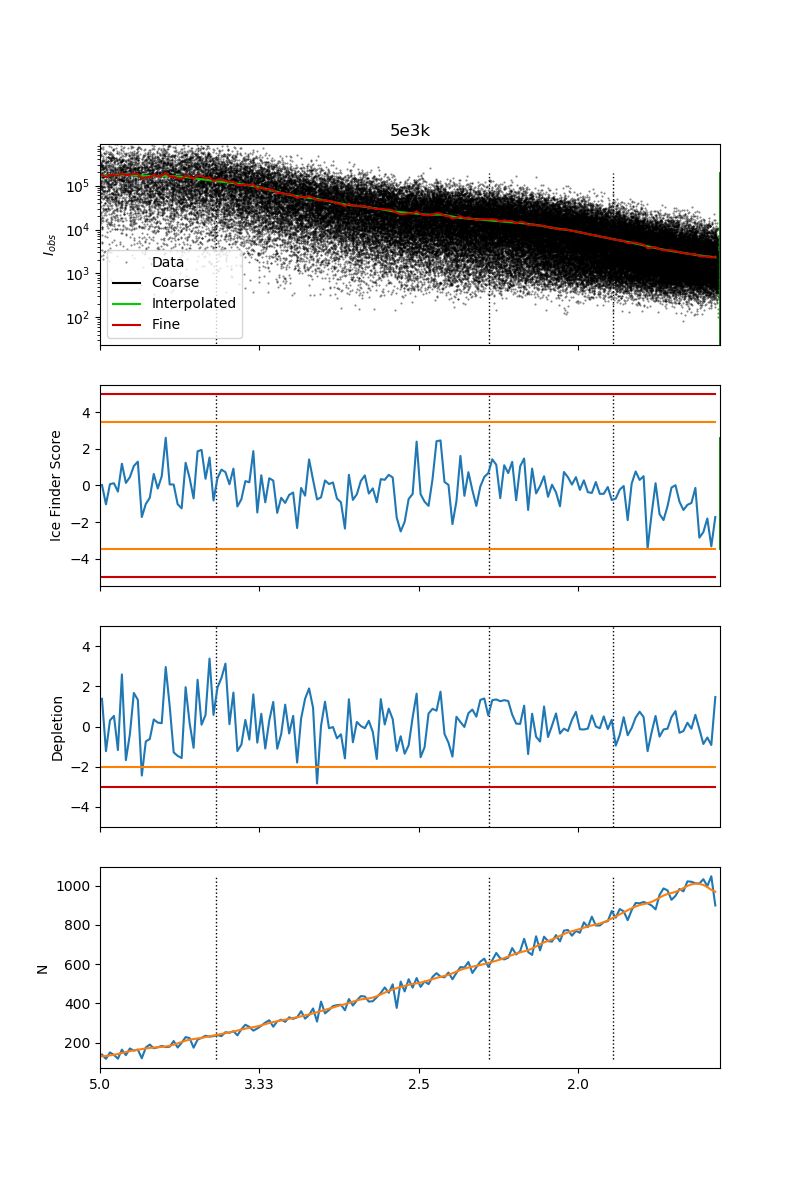

Supplement: Supplementary file 3 [file d-77-00540-sup3.zip › IceBiasingImages/5e3k.png]

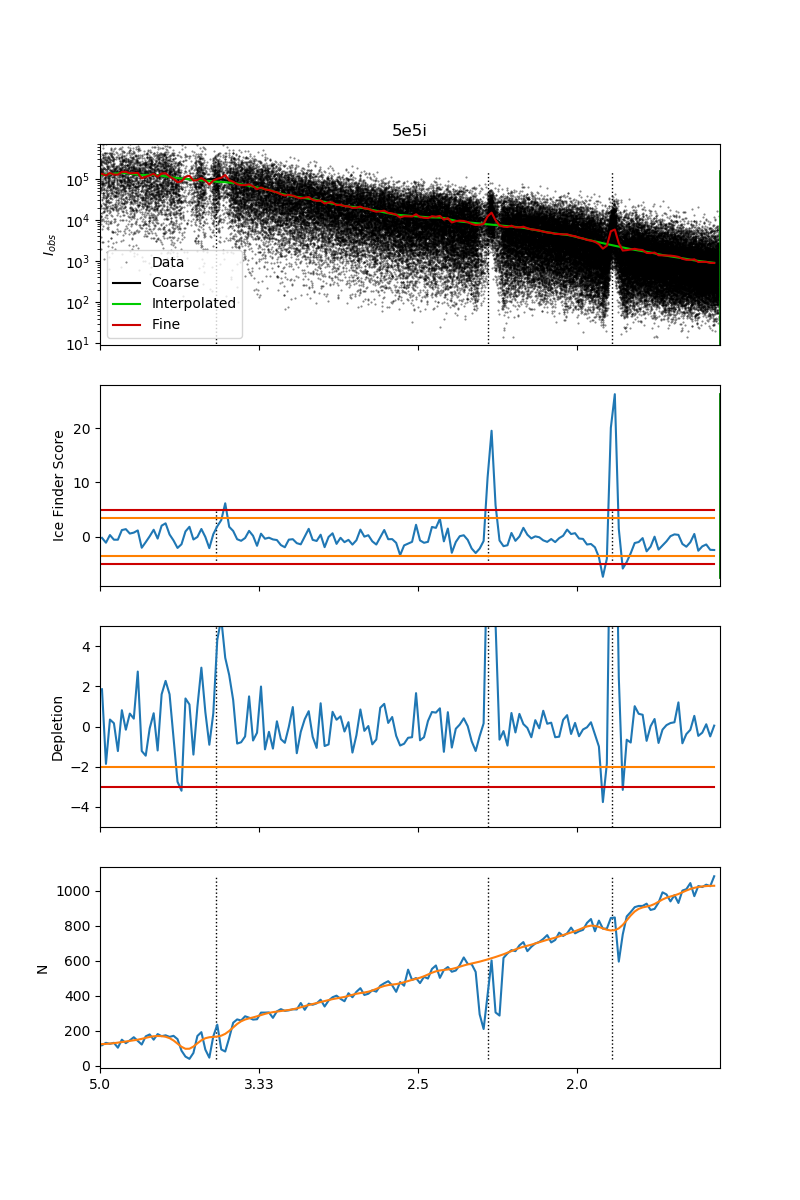

Supplement: Supplementary file 3 [file d-77-00540-sup3.zip › IceBiasingImages/5e5i.png]

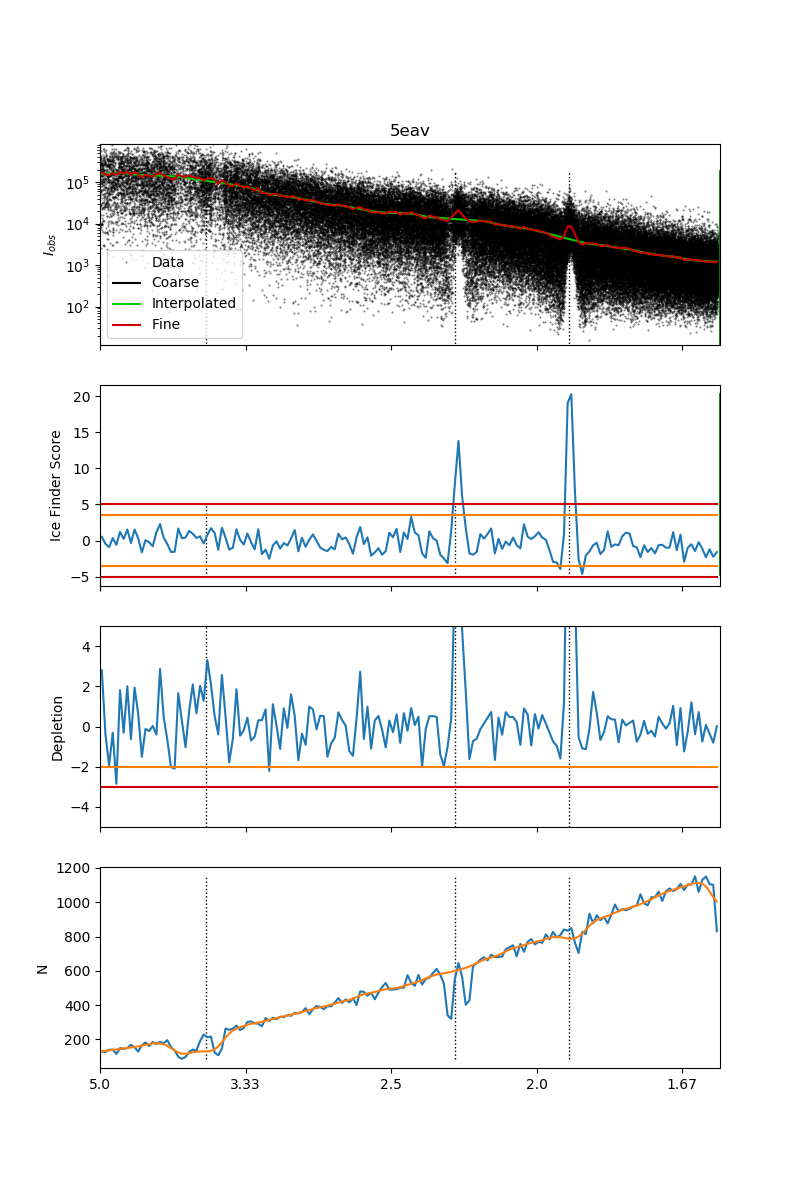

Supplement: Supplementary file 3 [file d-77-00540-sup3.zip › IceBiasingImages/5eav.png]

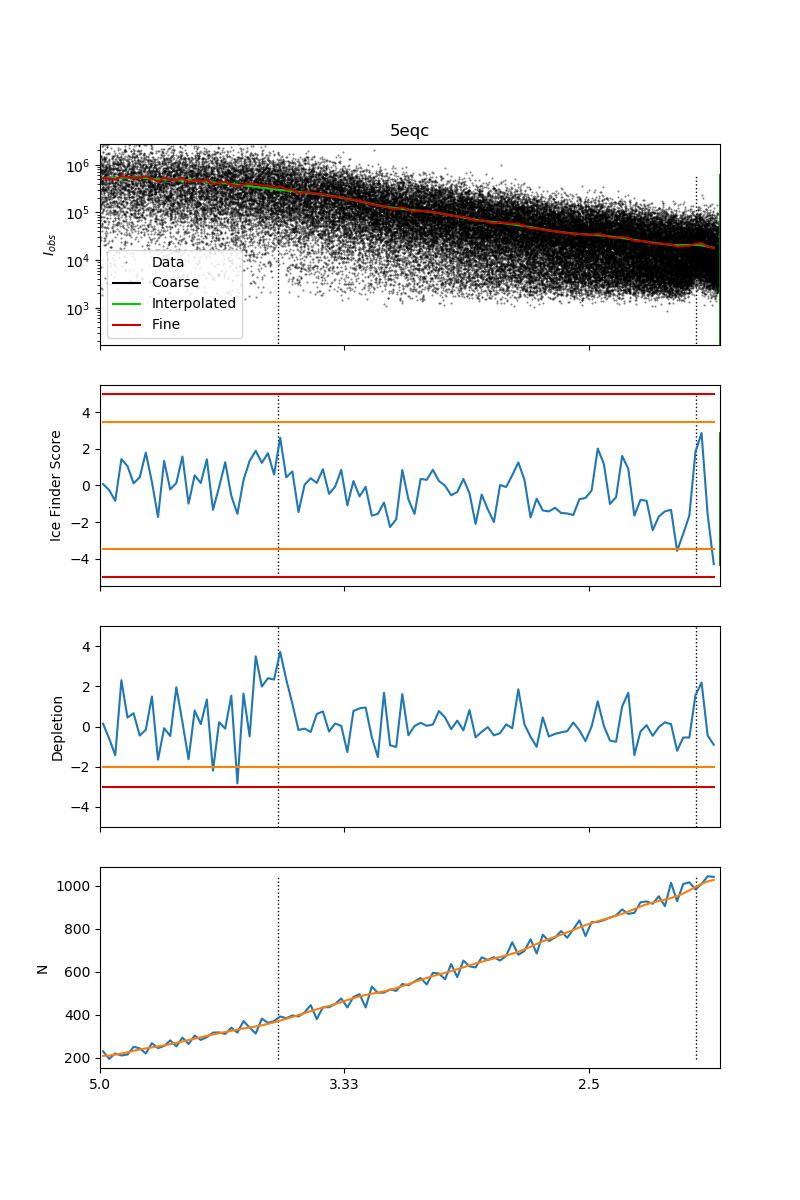

Supplement: Supplementary file 3 [file d-77-00540-sup3.zip › IceBiasingImages/5eqc.png]
