# Supplementary figures and images for: Ice in biomolecular cryocrystallography (part 1 of 2)
Source: Acta Crystallogr D Struct Biol. 2021 Mar 30;77(Pt 4):540–54. doi: 10.1107/S2059798321001170 (PMC8025888; doi:10.1107/S2059798321001170)

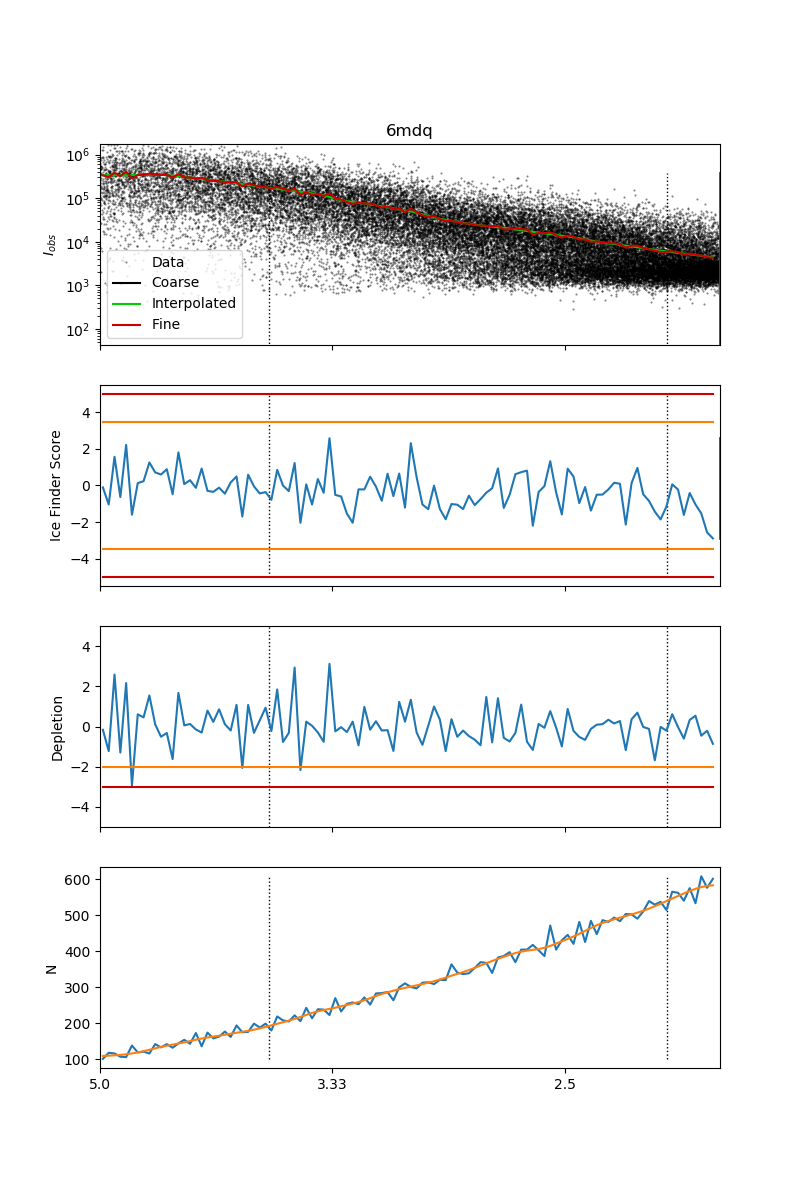

Supplement: Supplementary file 3 [file d-77-00540-sup3.zip › IceBiasingImages/6mdq.png]

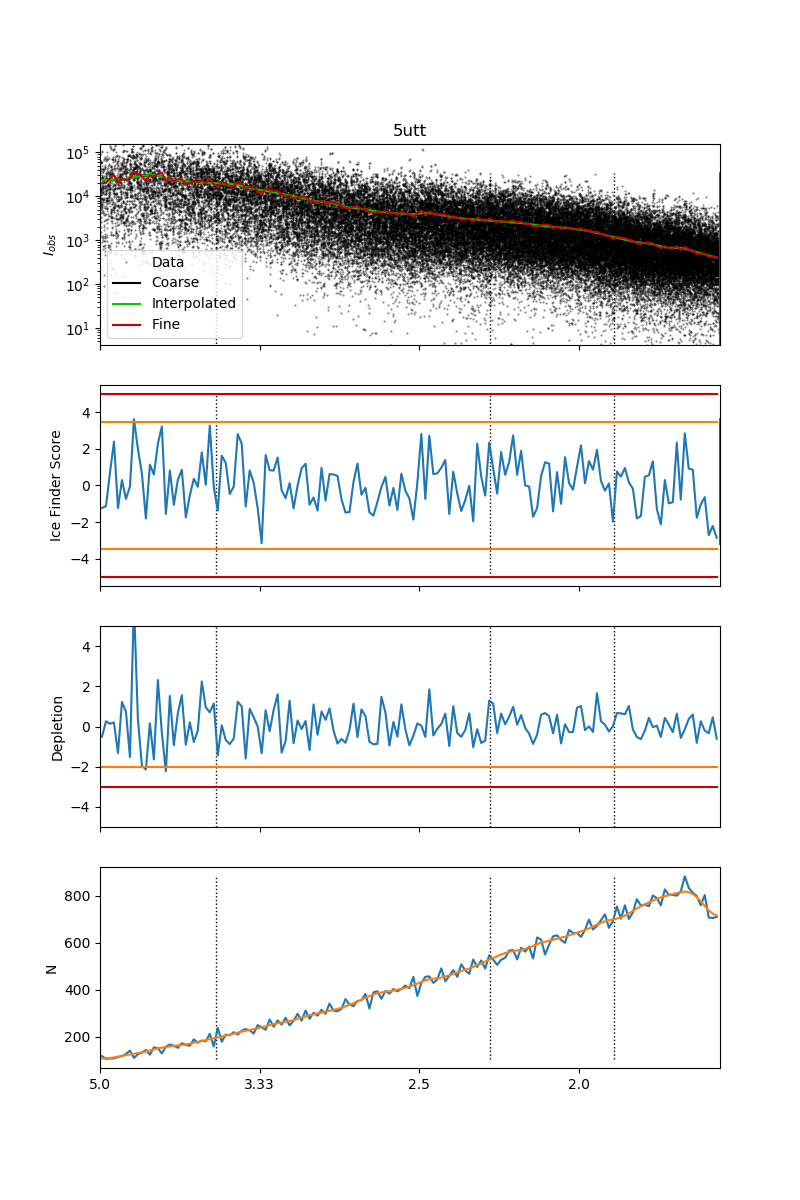

Supplement: Supplementary file 3 [file d-77-00540-sup3.zip › IceBiasingImages/5utt.png]

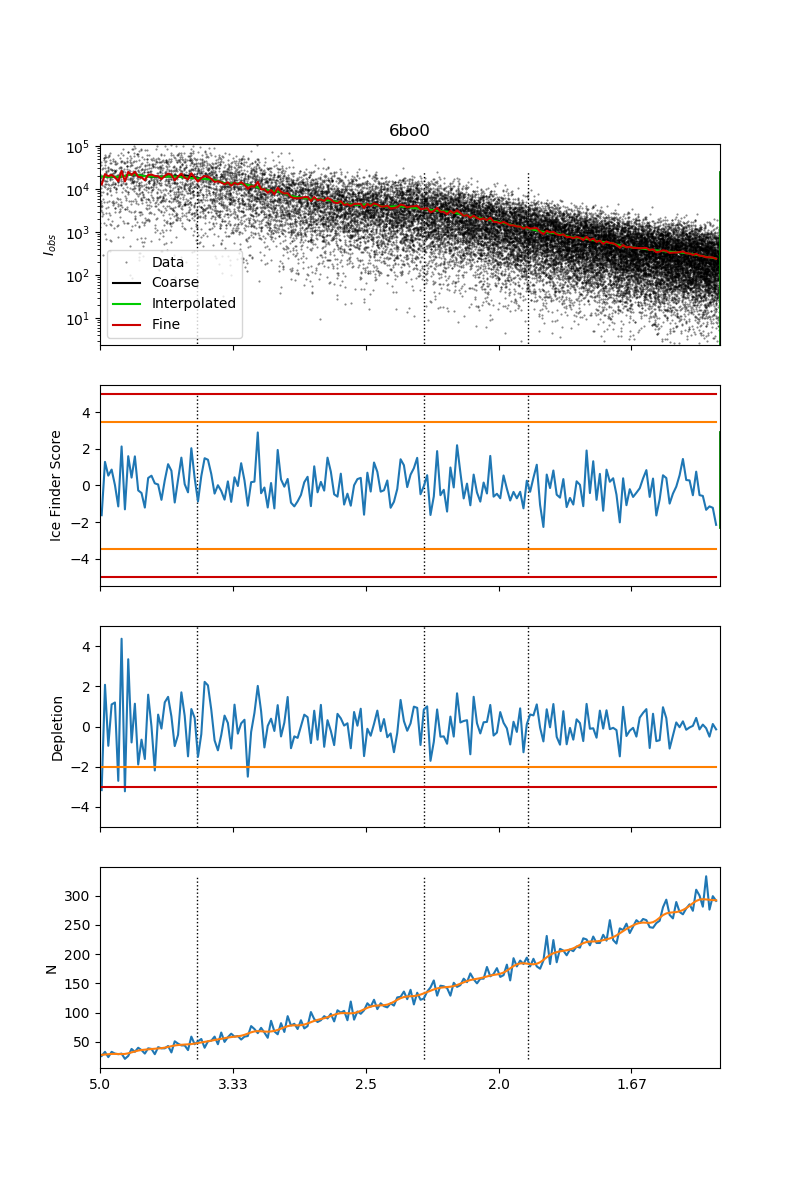

Supplement: Supplementary file 3 [file d-77-00540-sup3.zip › IceBiasingImages/6bo0.png]

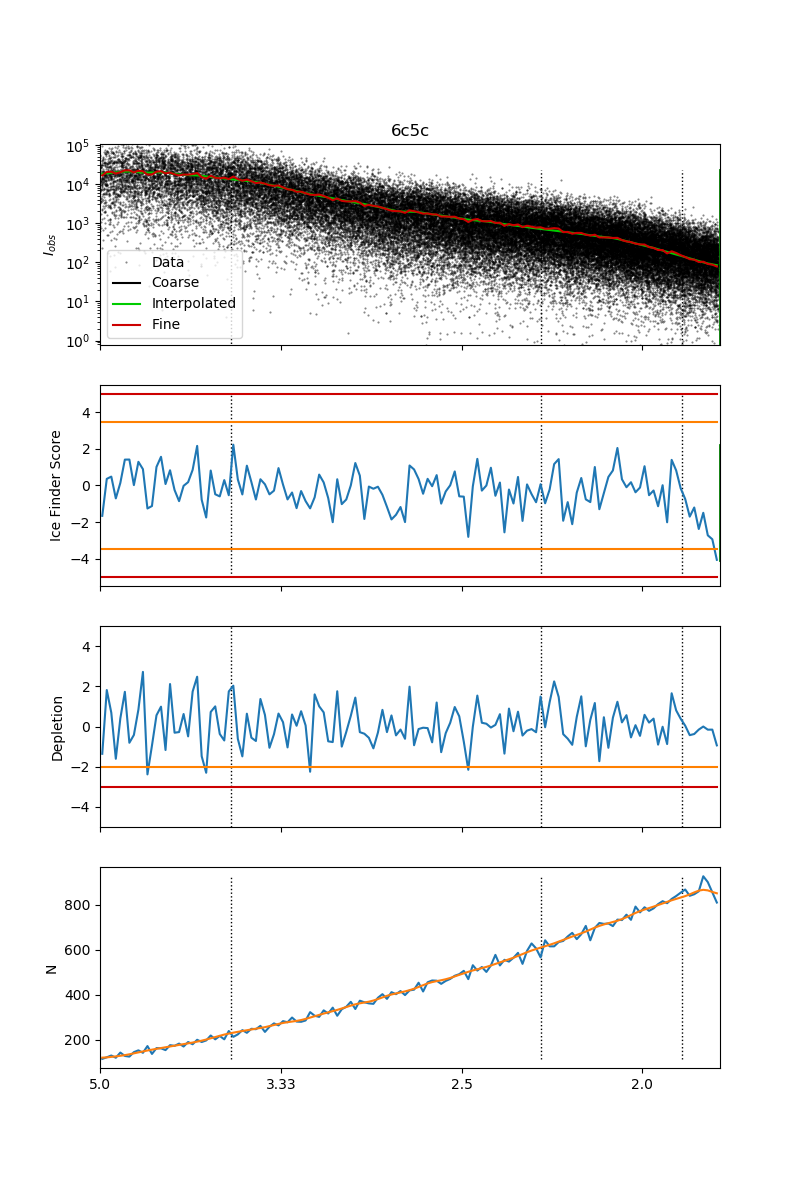

Supplement: Supplementary file 3 [file d-77-00540-sup3.zip › IceBiasingImages/6c5c.png]

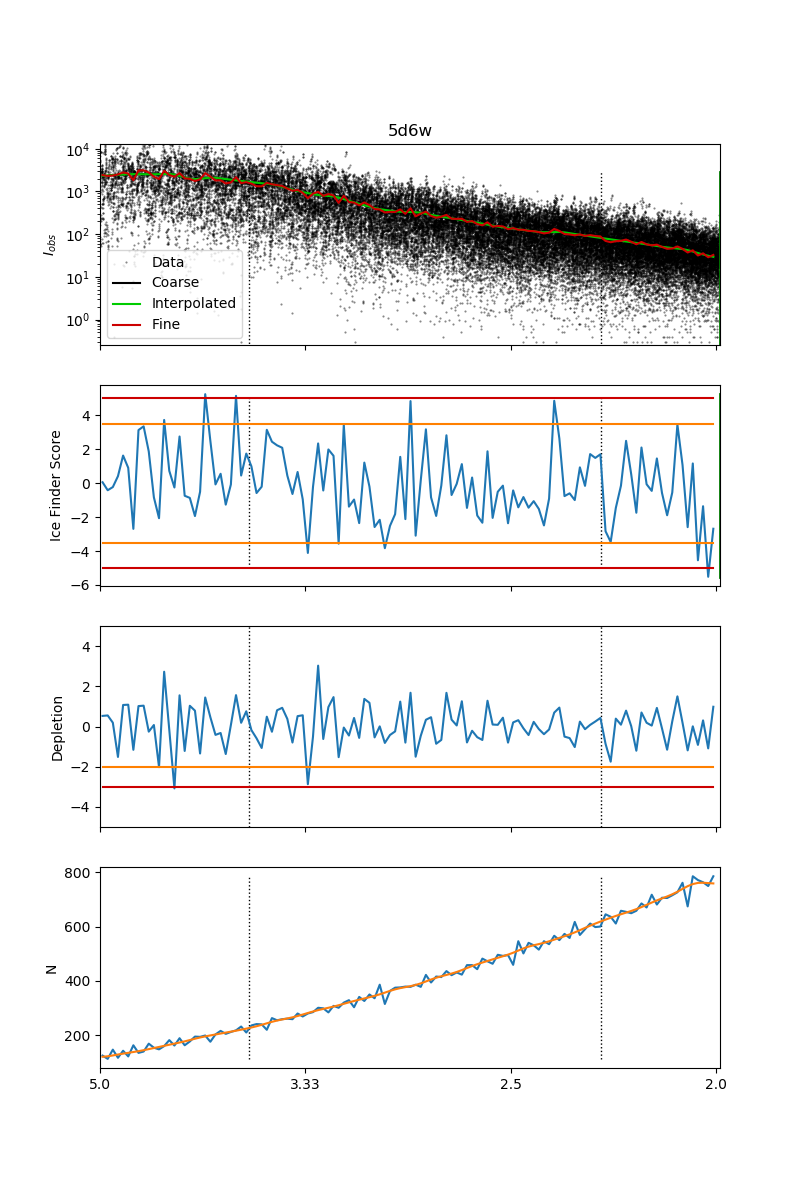

Supplement: Supplementary file 3 [file d-77-00540-sup3.zip › IceBiasingImages/5d6w.png]

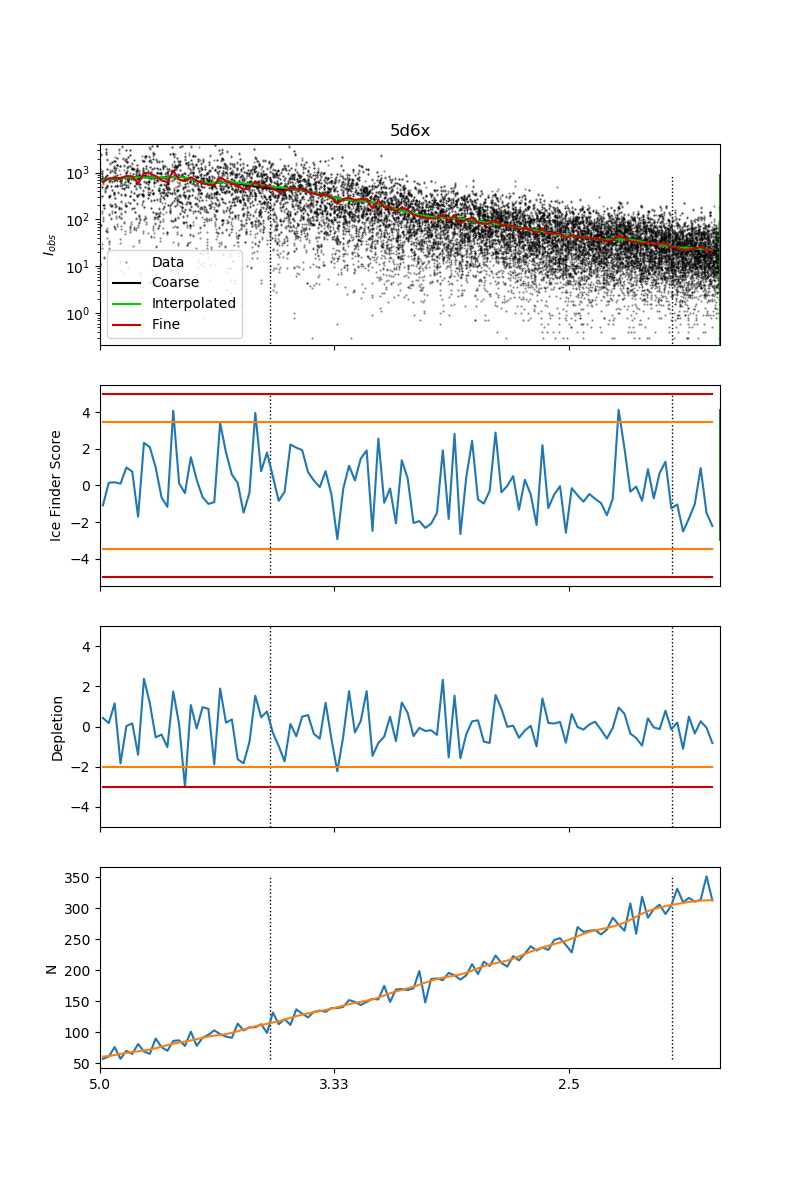

Supplement: Supplementary file 3 [file d-77-00540-sup3.zip › IceBiasingImages/5d6x.png]

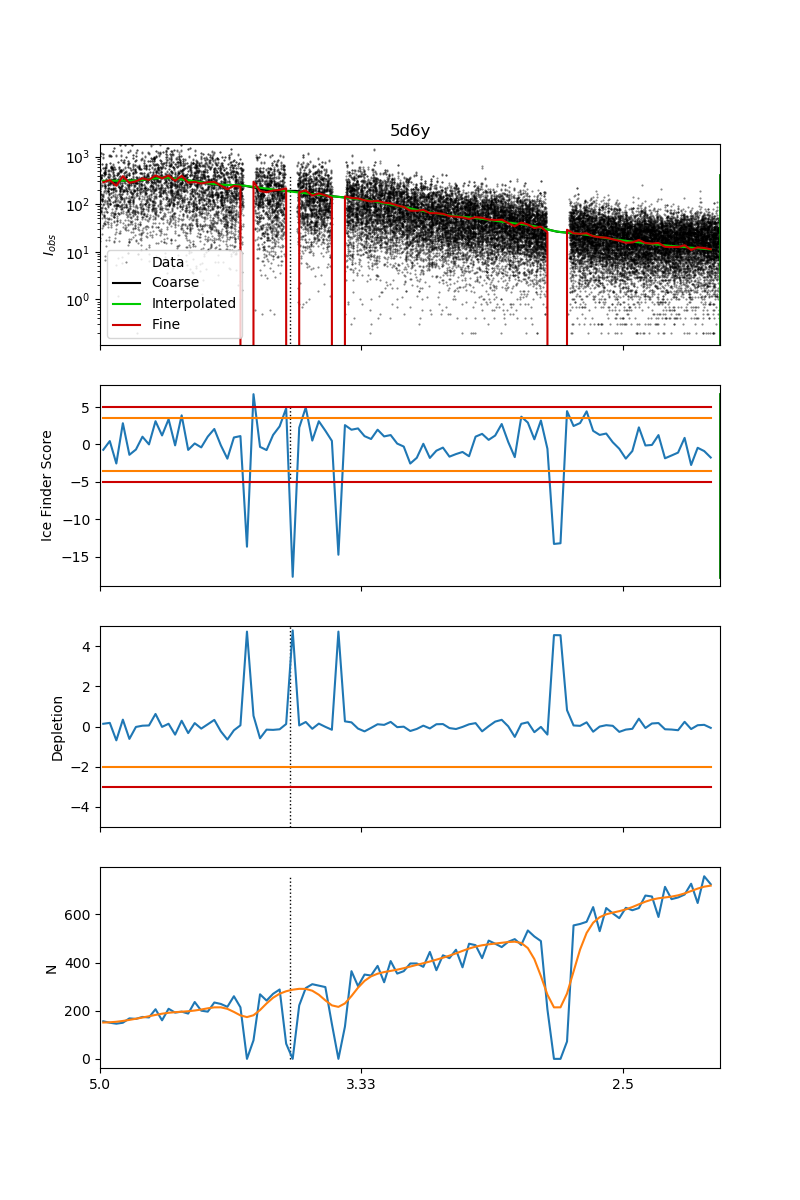

Supplement: Supplementary file 3 [file d-77-00540-sup3.zip › IceBiasingImages/5d6y.png]

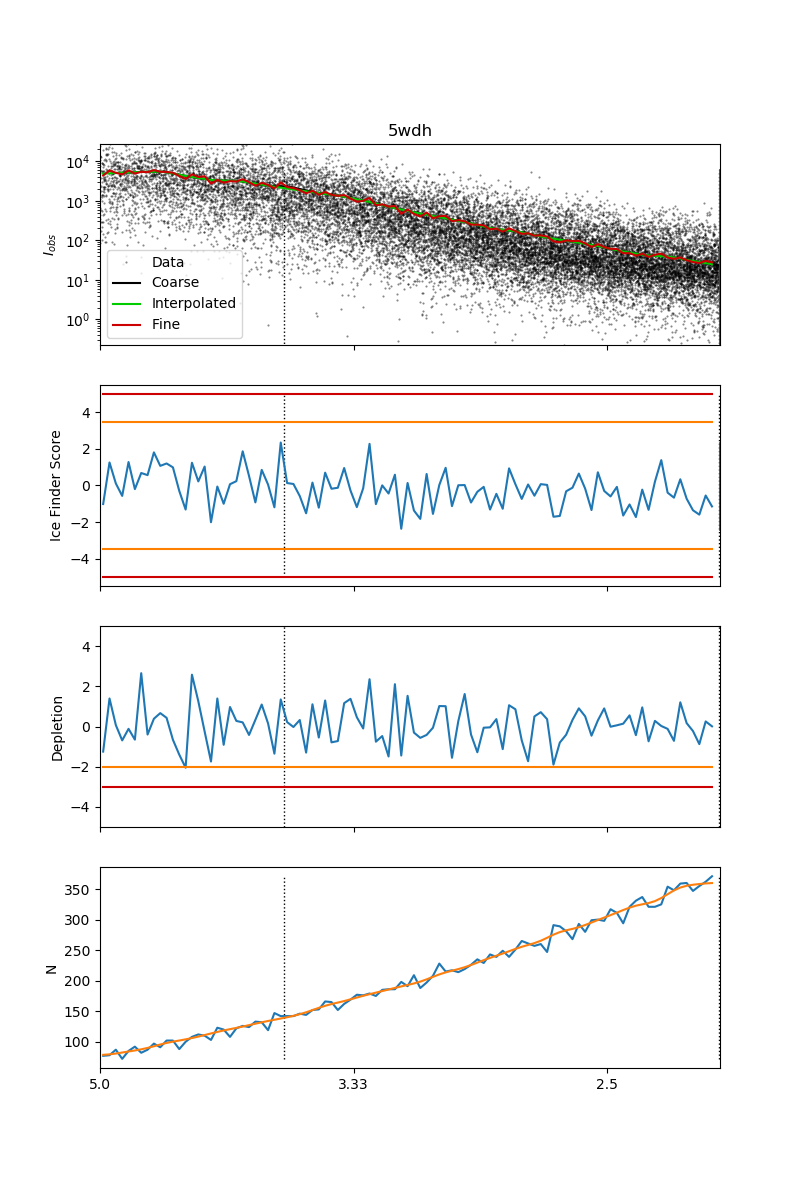

Supplement: Supplementary file 3 [file d-77-00540-sup3.zip › IceBiasingImages/5wdh.png]

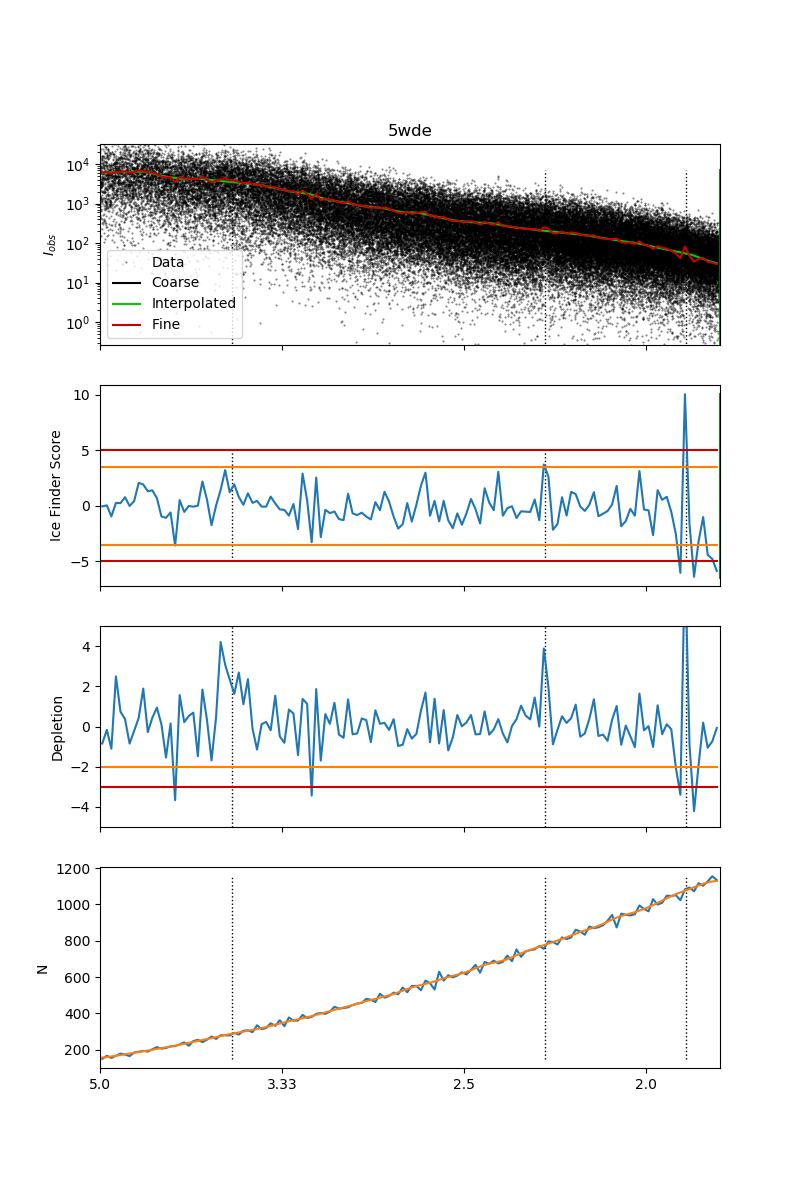

Supplement: Supplementary file 3 [file d-77-00540-sup3.zip › IceBiasingImages/5wde.png]

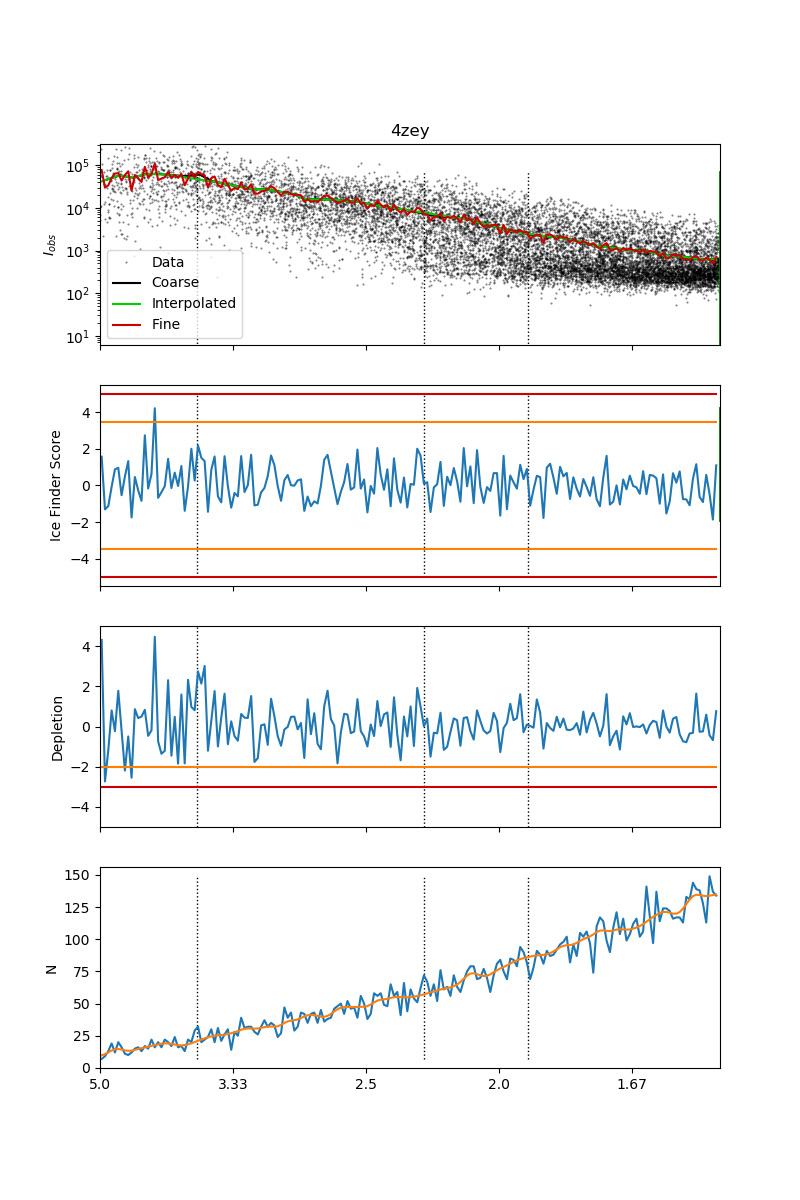

Supplement: Supplementary file 3 [file d-77-00540-sup3.zip › IceBiasingImages/4zey.png]

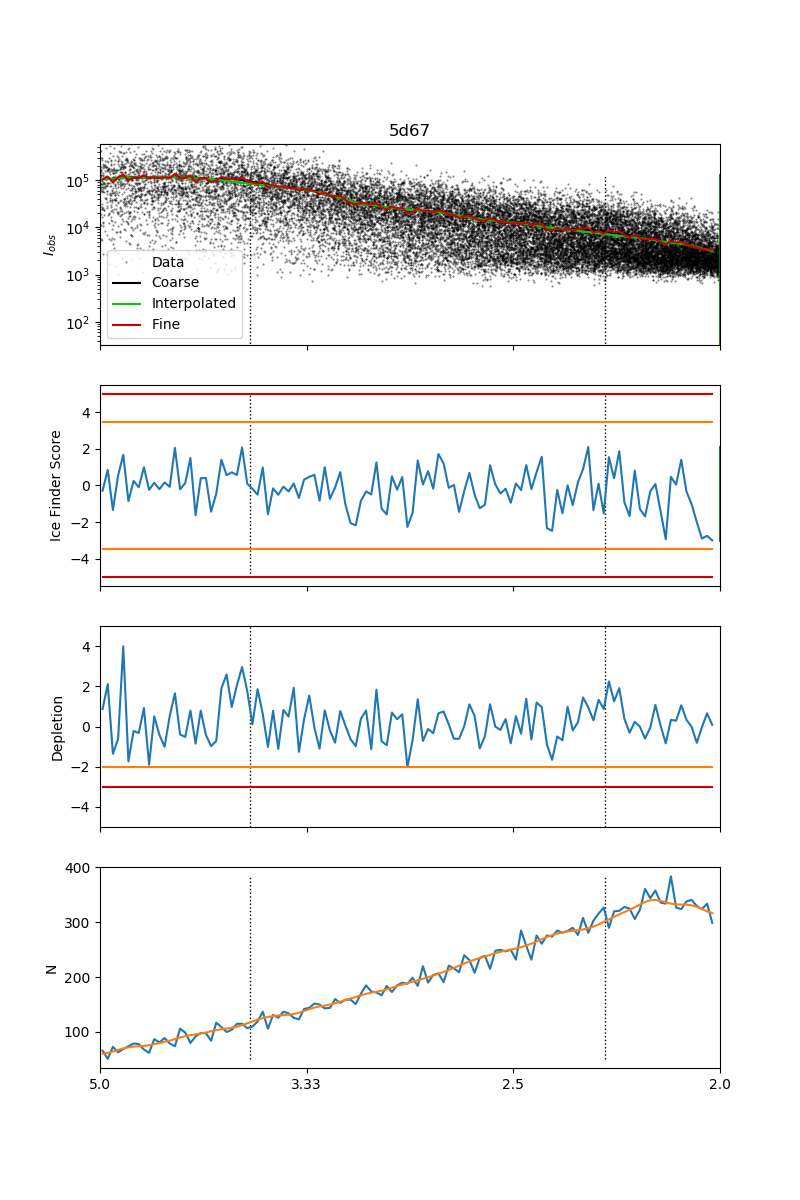

Supplement: Supplementary file 3 [file d-77-00540-sup3.zip › IceBiasingImages/5d67.png]

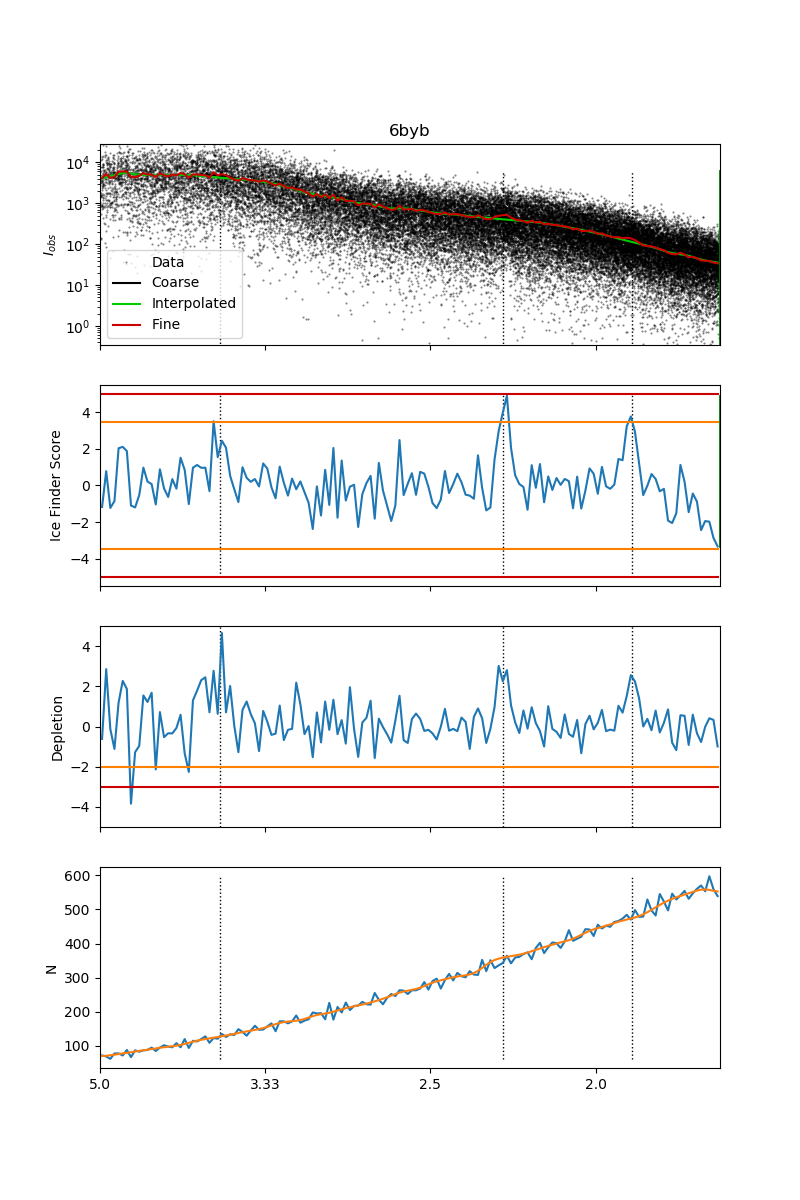

Supplement: Supplementary file 3 [file d-77-00540-sup3.zip › IceBiasingImages/6byb.png]

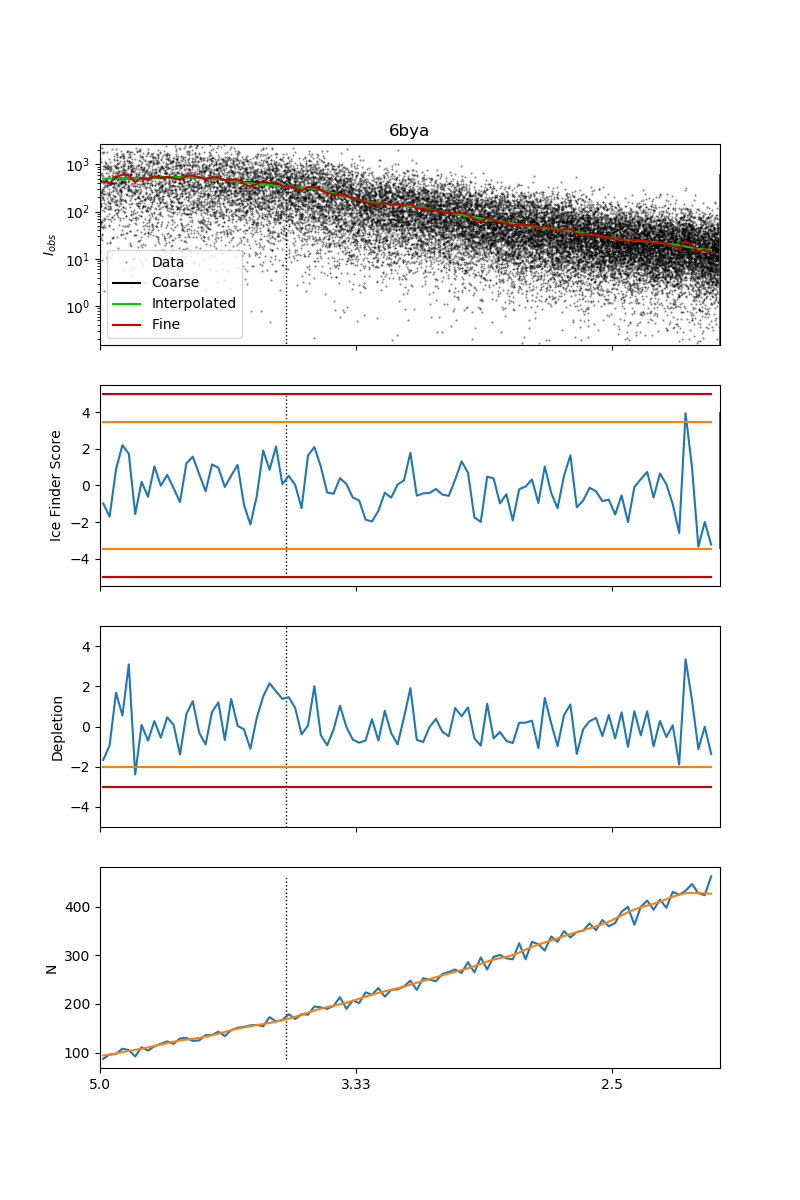

Supplement: Supplementary file 3 [file d-77-00540-sup3.zip › IceBiasingImages/6bya.png]

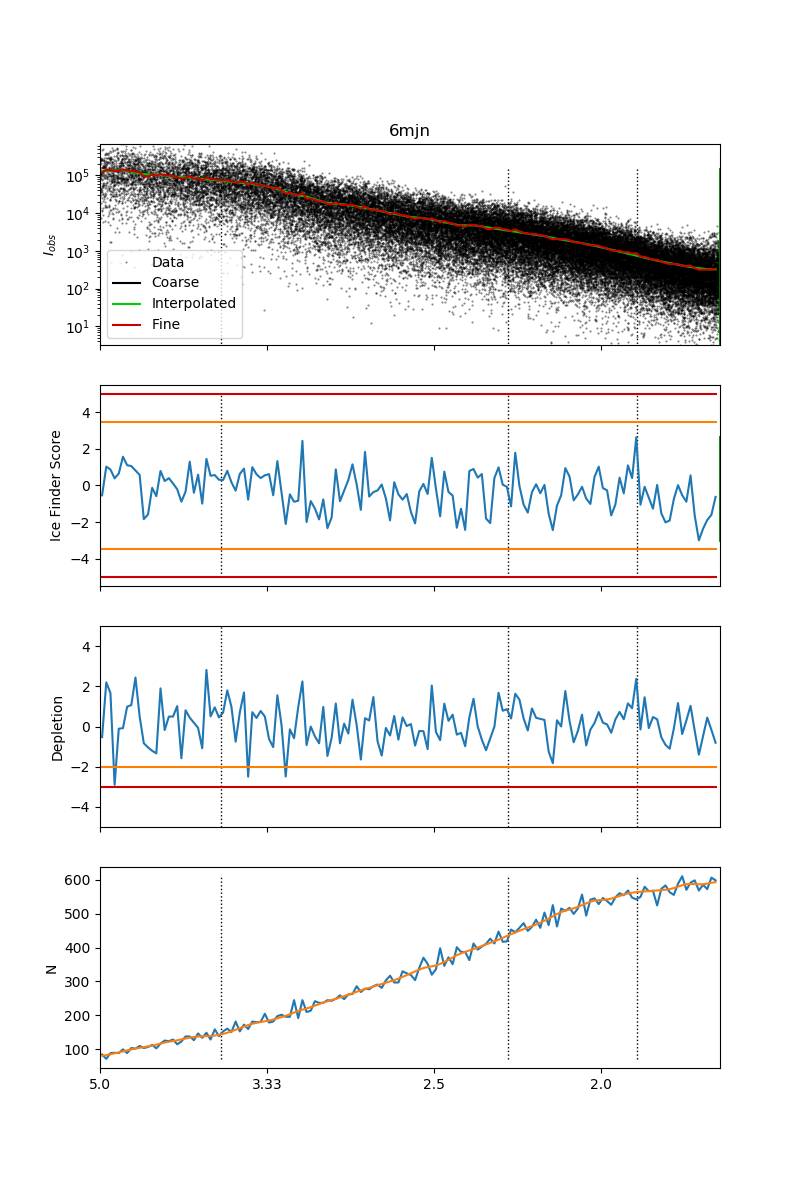

Supplement: Supplementary file 3 [file d-77-00540-sup3.zip › IceBiasingImages/6mjn.png]

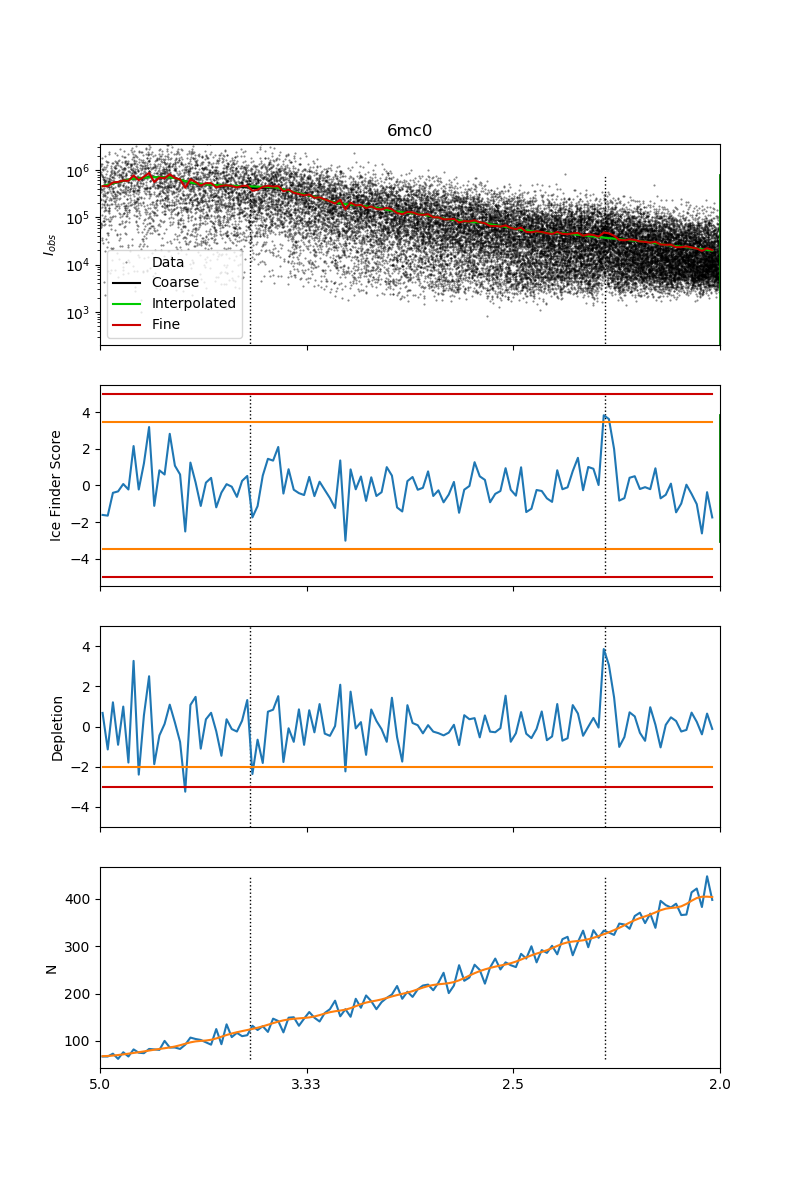

Supplement: Supplementary file 3 [file d-77-00540-sup3.zip › IceBiasingImages/6mc0.png]

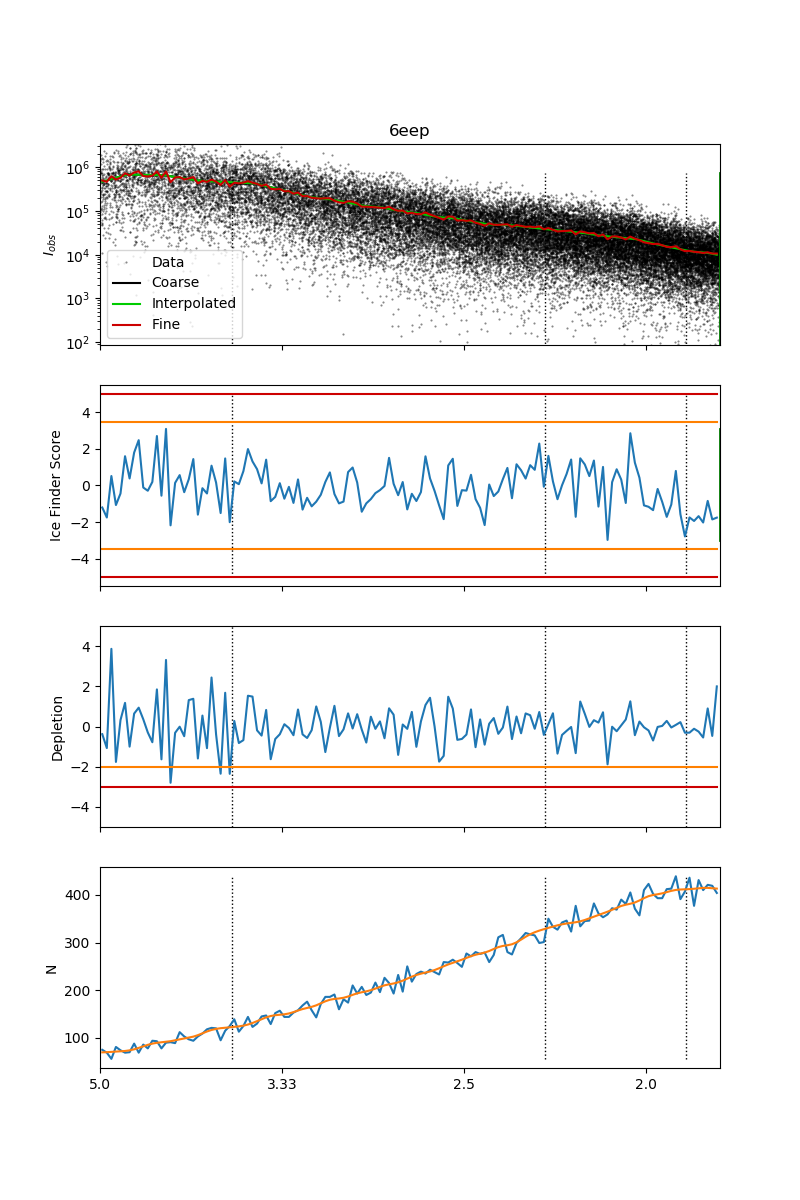

Supplement: Supplementary file 3 [file d-77-00540-sup3.zip › IceBiasingImages/6eep.png]

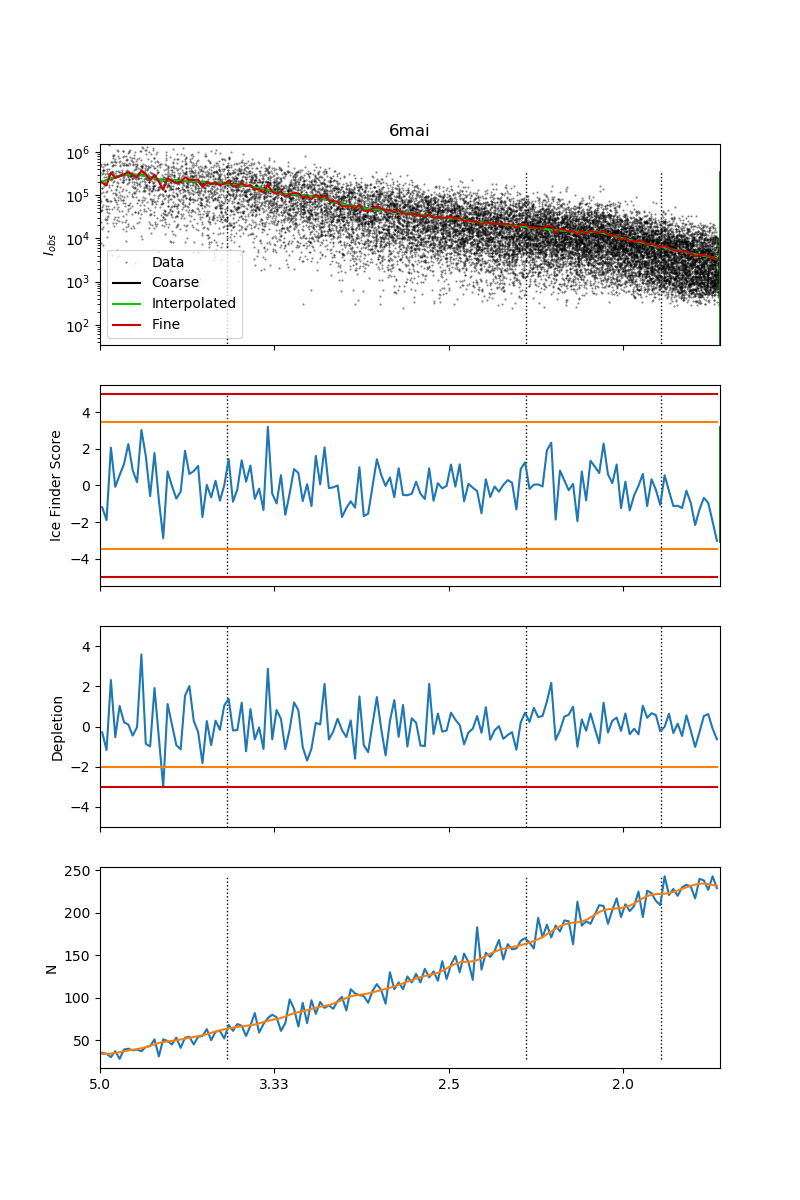

Supplement: Supplementary file 3 [file d-77-00540-sup3.zip › IceBiasingImages/6mai.png]

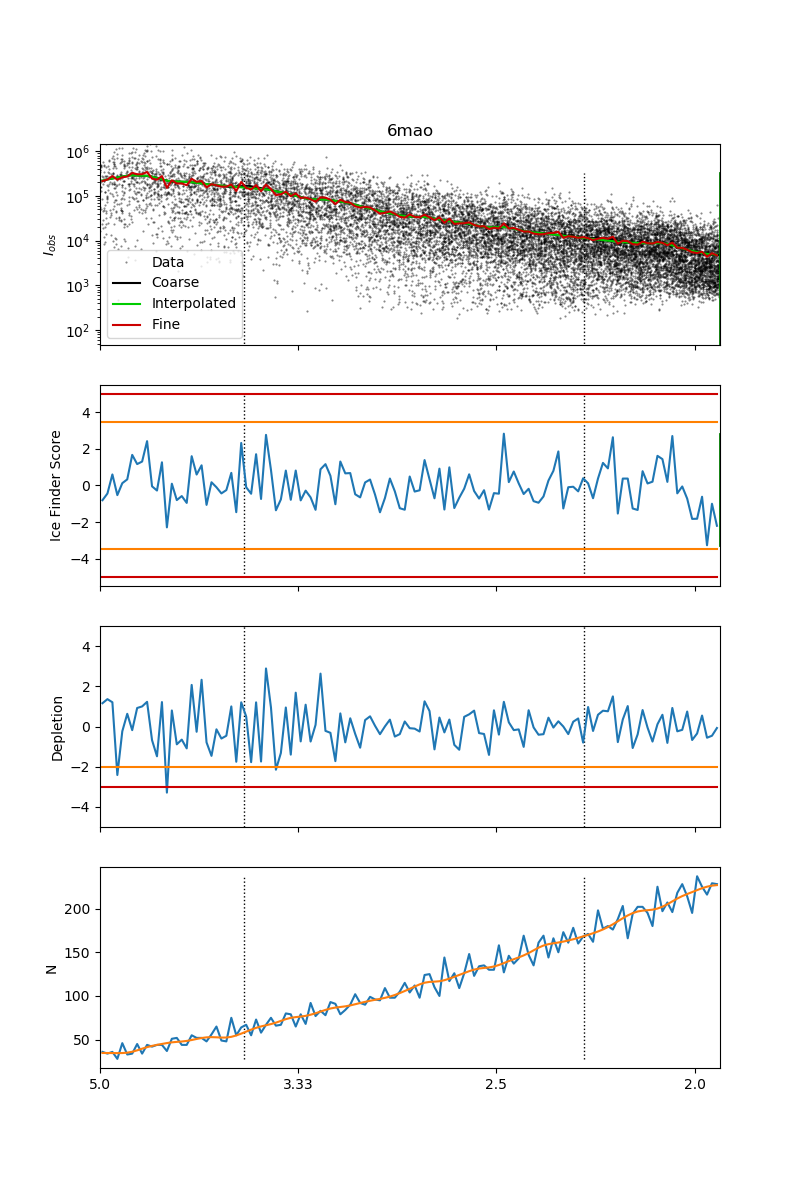

Supplement: Supplementary file 3 [file d-77-00540-sup3.zip › IceBiasingImages/6mao.png]

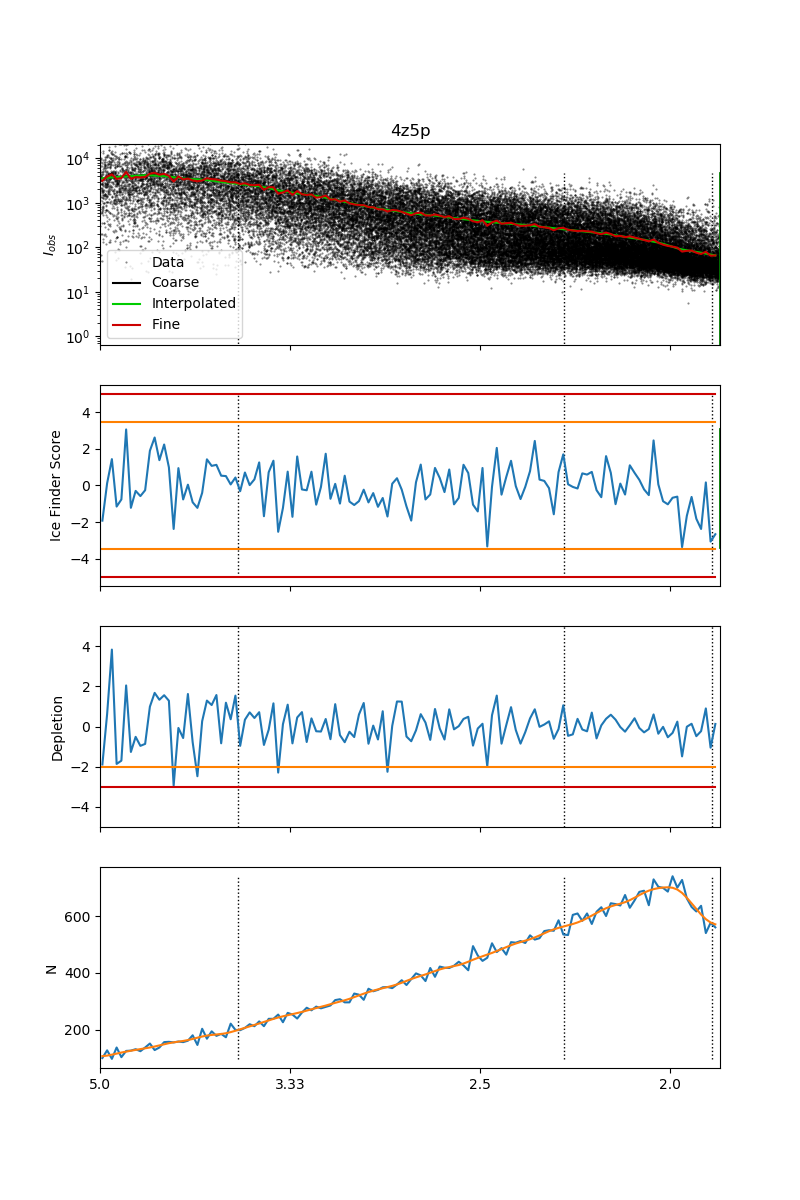

Supplement: Supplementary file 3 [file d-77-00540-sup3.zip › IceBiasingImages/4z5p.png]

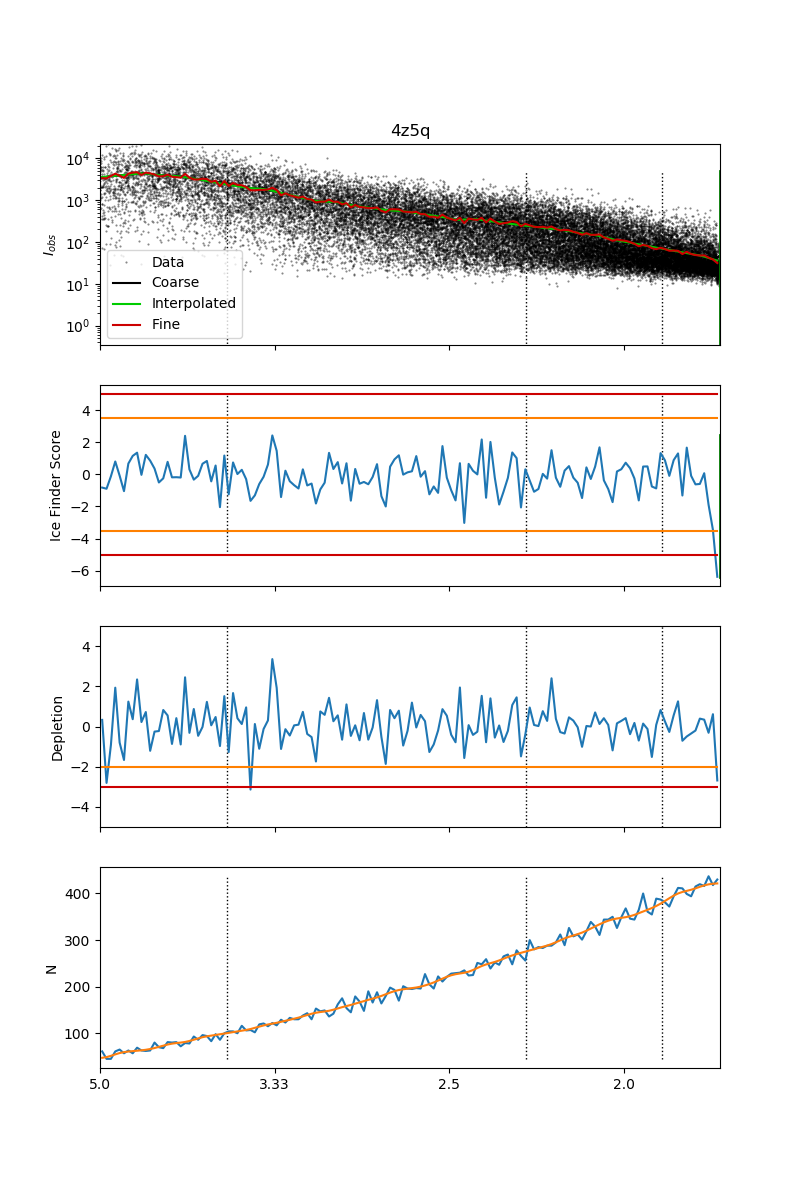

Supplement: Supplementary file 3 [file d-77-00540-sup3.zip › IceBiasingImages/4z5q.png]

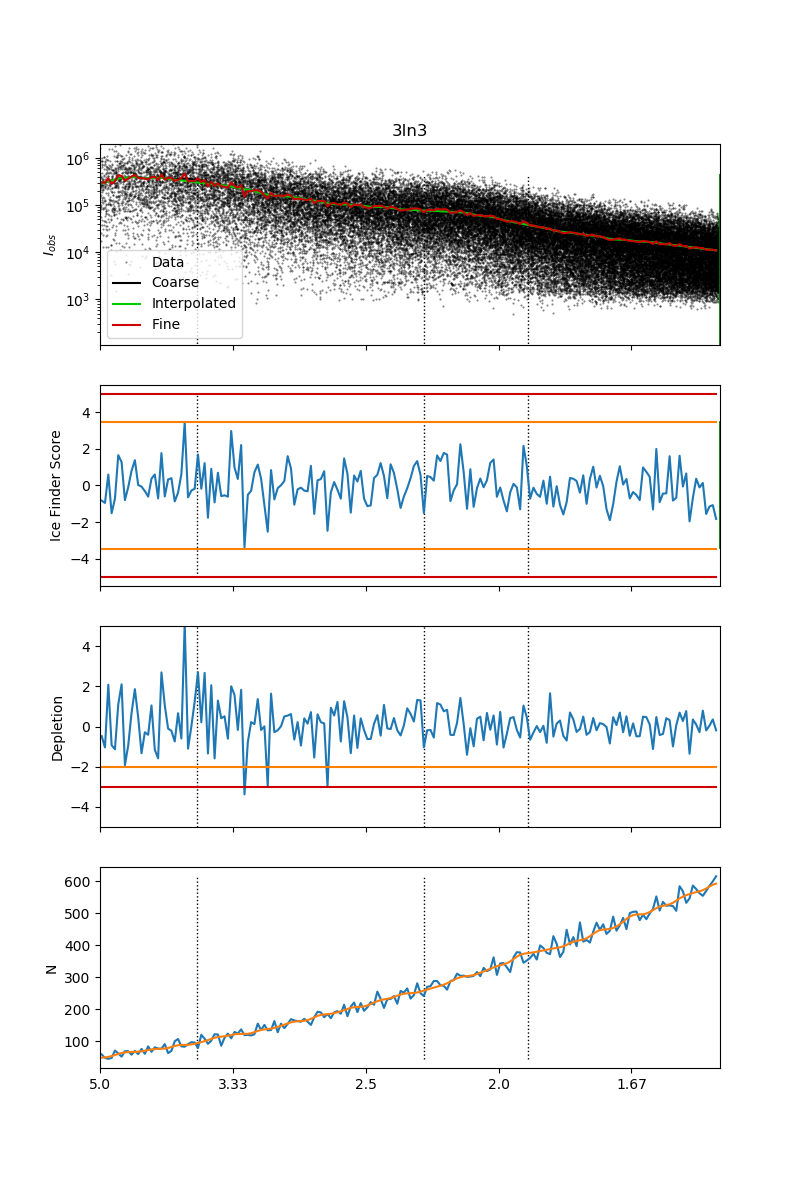

Supplement: Supplementary file 3 [file d-77-00540-sup3.zip › IceBiasingImages/3ln3.png]

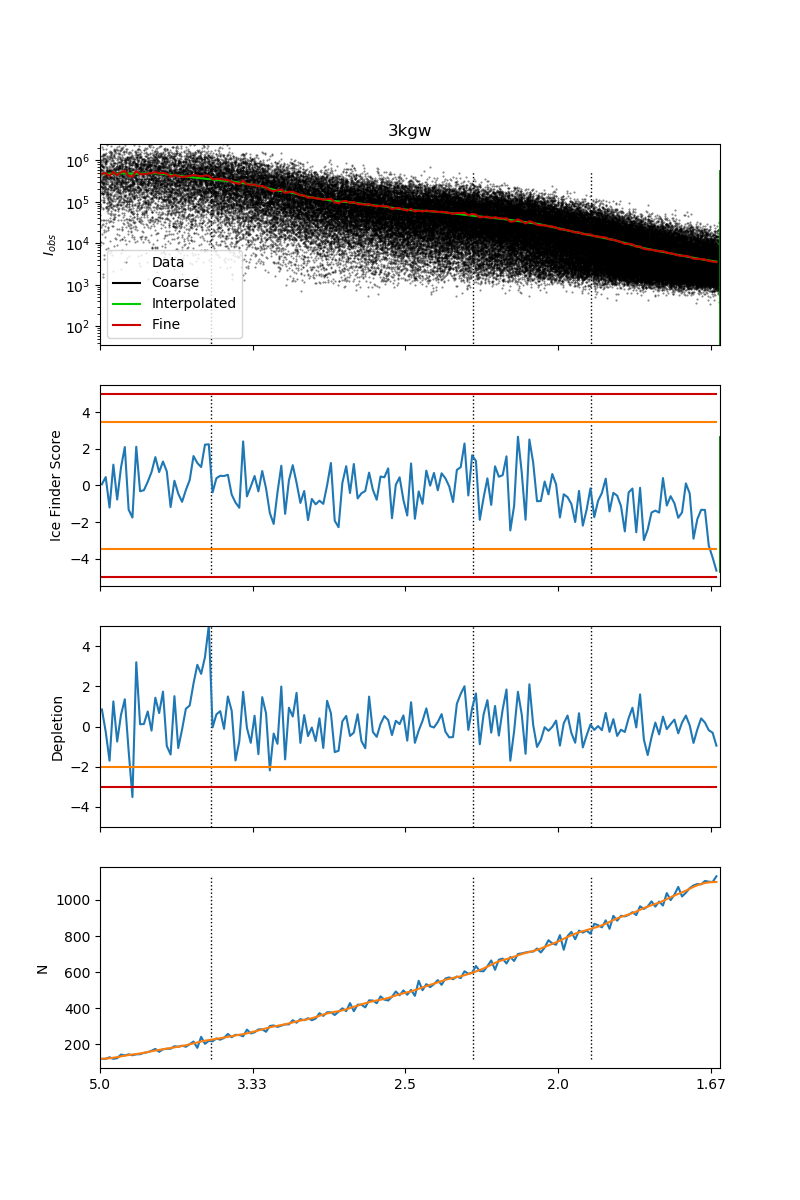

Supplement: Supplementary file 3 [file d-77-00540-sup3.zip › IceBiasingImages/3kgw.png]

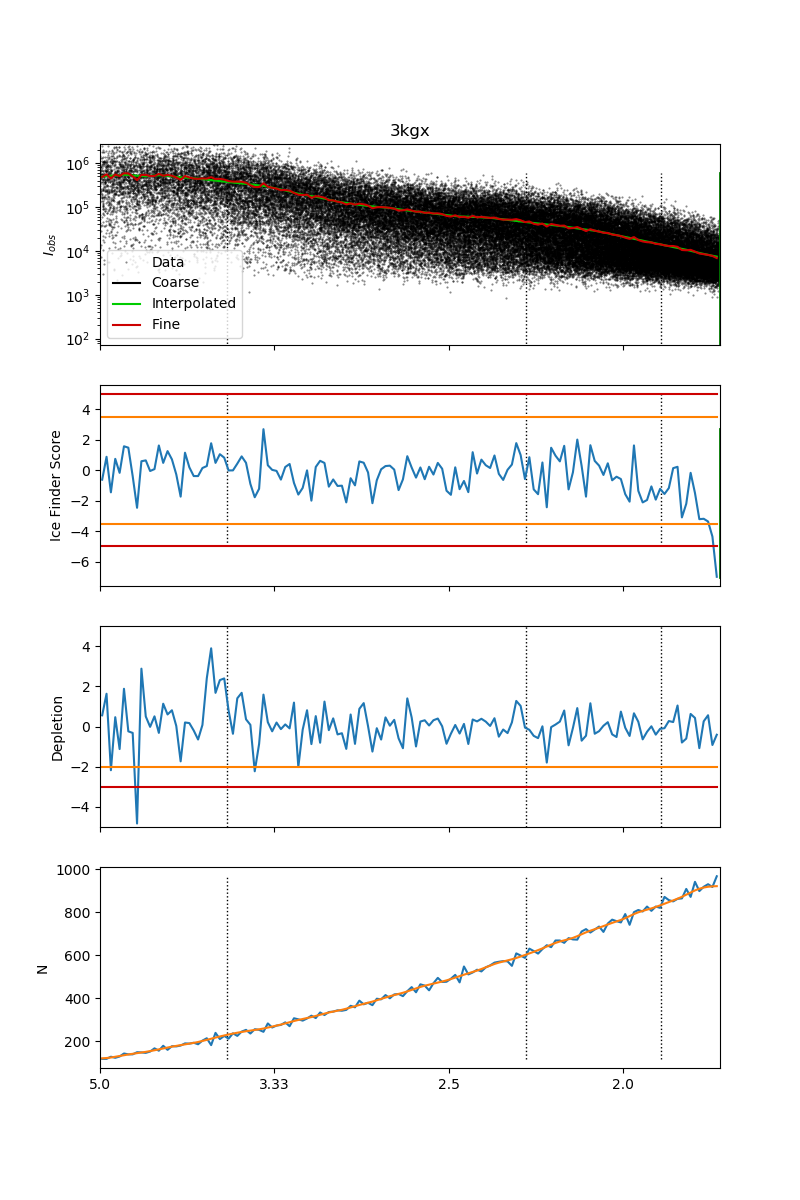

Supplement: Supplementary file 3 [file d-77-00540-sup3.zip › IceBiasingImages/3kgx.png]

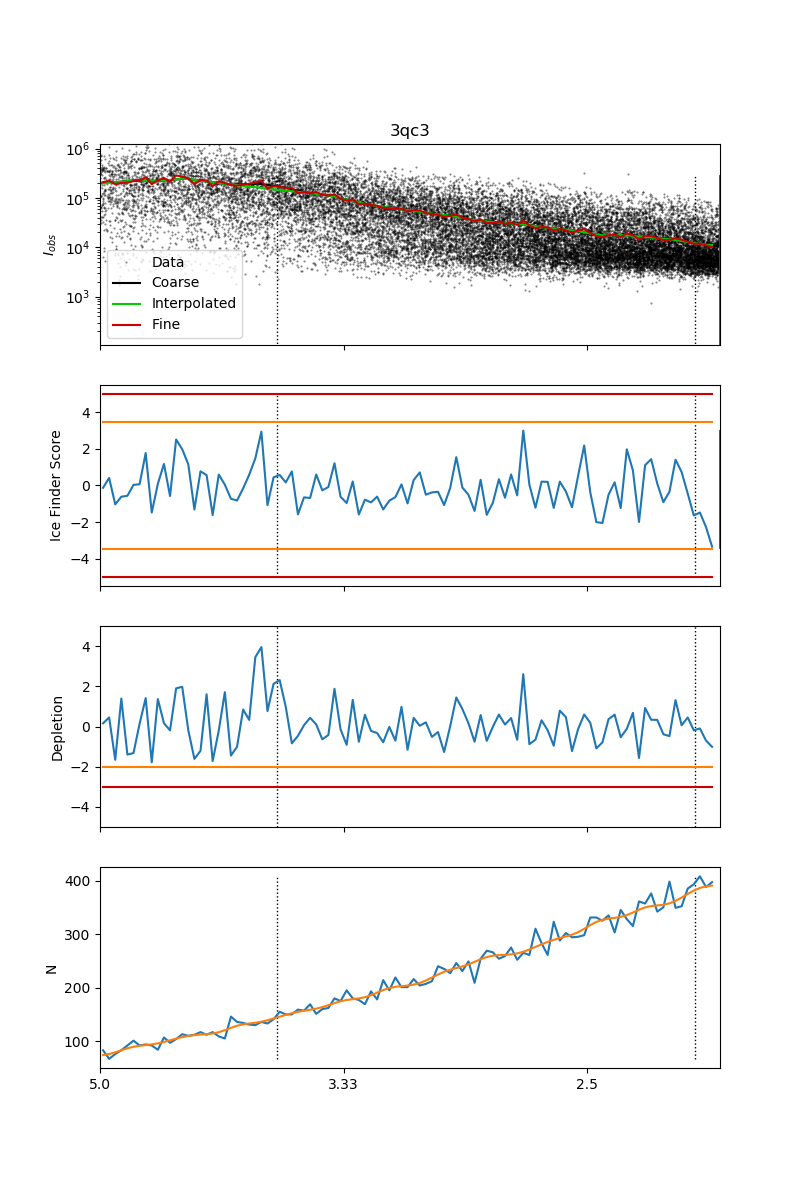

Supplement: Supplementary file 3 [file d-77-00540-sup3.zip › IceBiasingImages/3qc3.png]

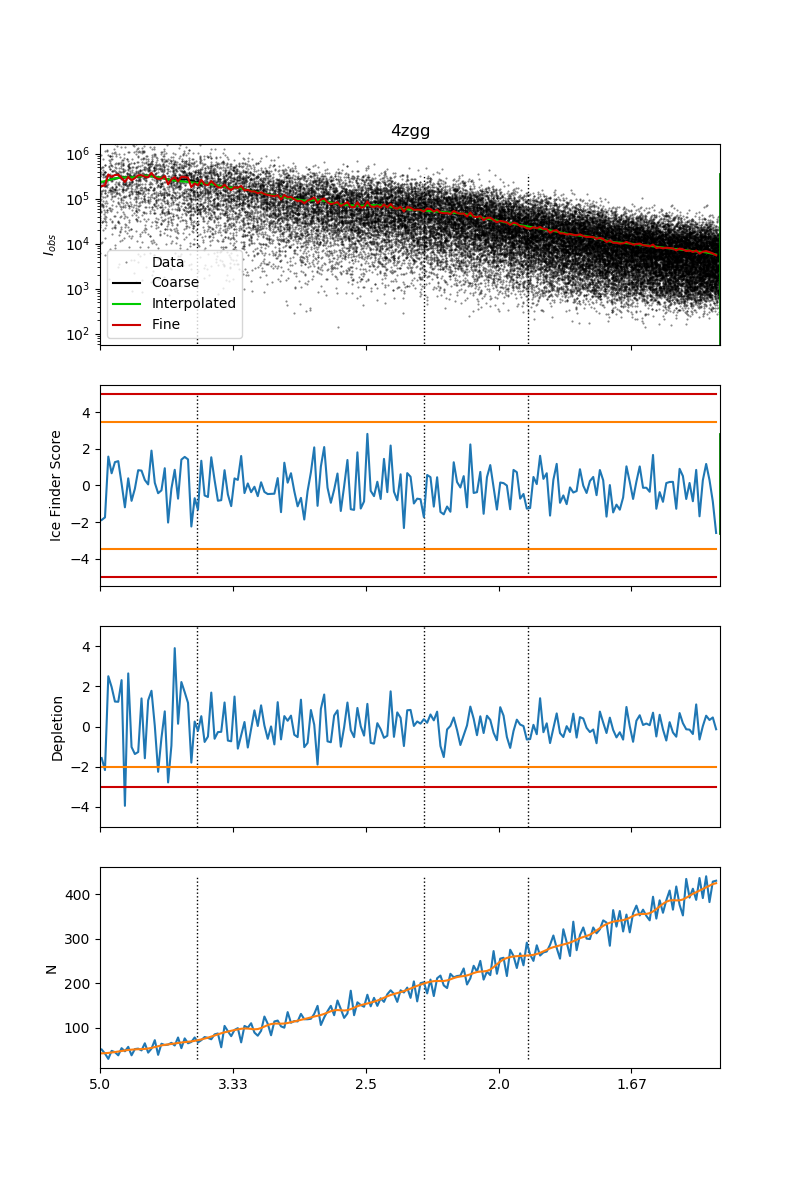

Supplement: Supplementary file 3 [file d-77-00540-sup3.zip › IceBiasingImages/4zgg.png]

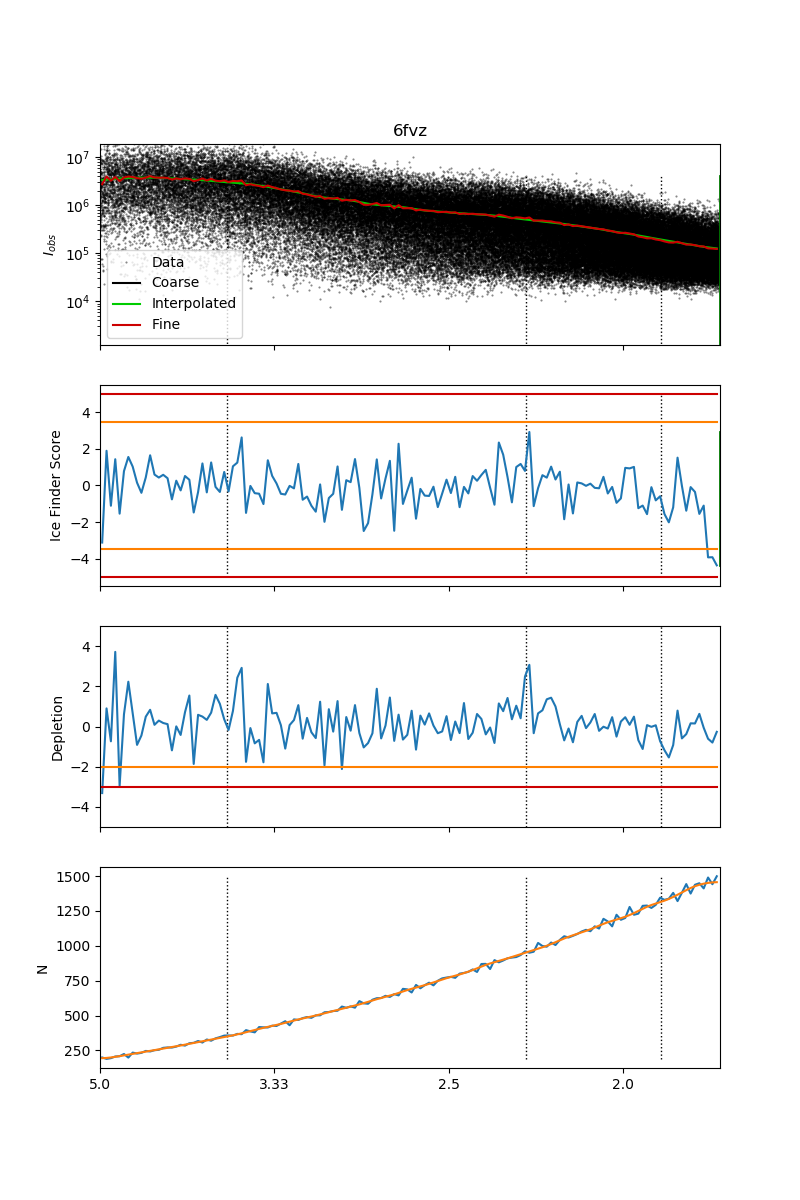

Supplement: Supplementary file 3 [file d-77-00540-sup3.zip › IceBiasingImages/6fvz.png]

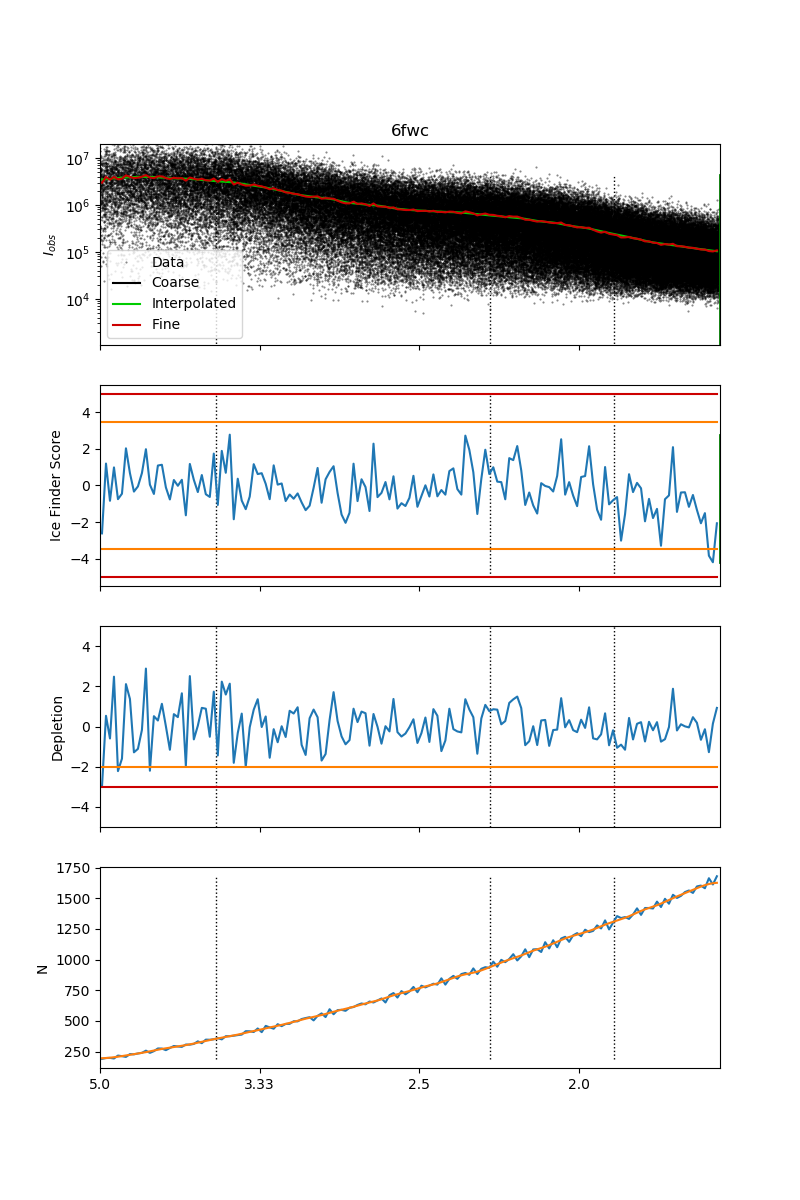

Supplement: Supplementary file 3 [file d-77-00540-sup3.zip › IceBiasingImages/6fwc.png]

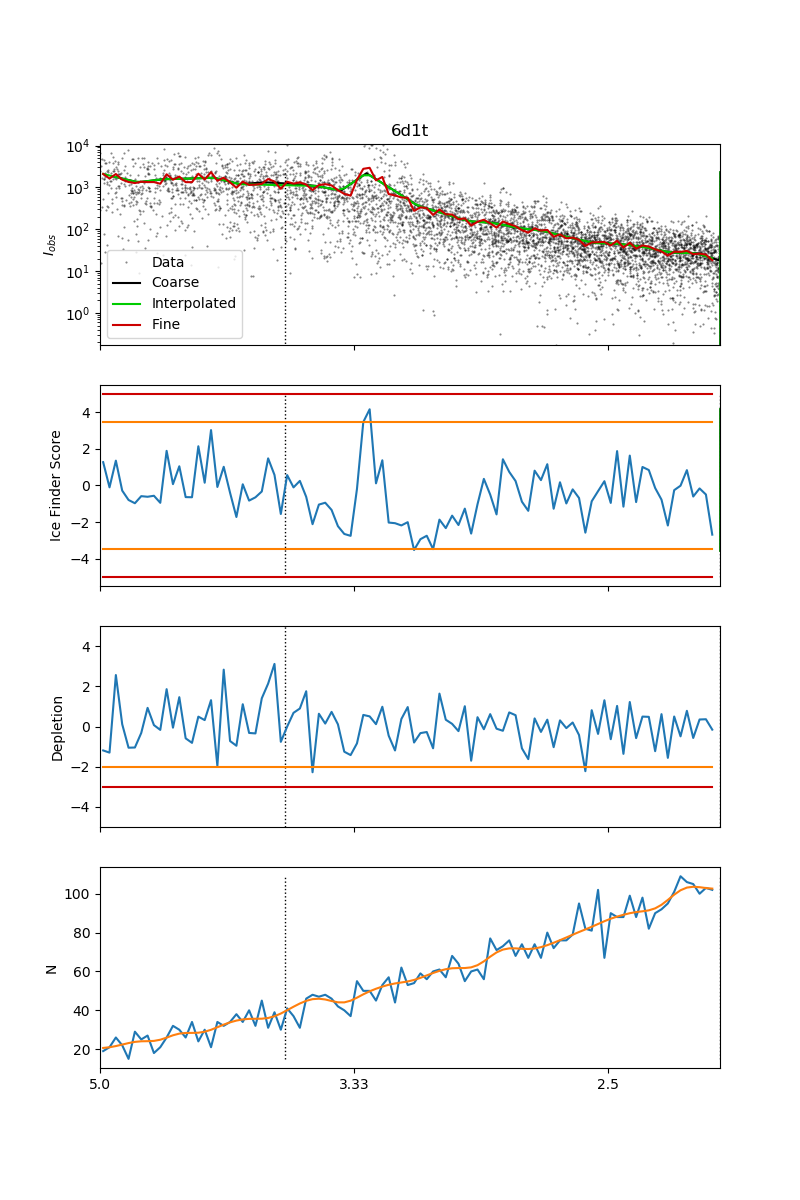

Supplement: Supplementary file 3 [file d-77-00540-sup3.zip › IceBiasingImages/6d1t.png]

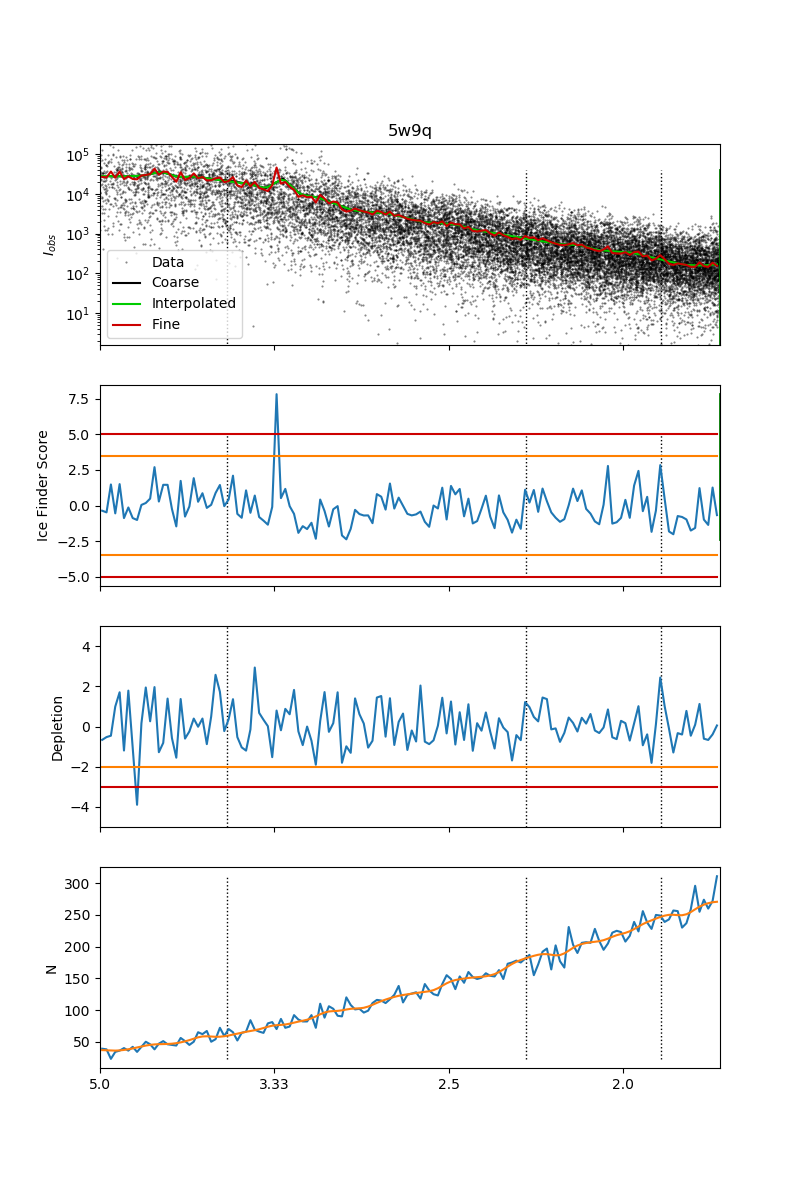

Supplement: Supplementary file 3 [file d-77-00540-sup3.zip › IceBiasingImages/5w9q.png]

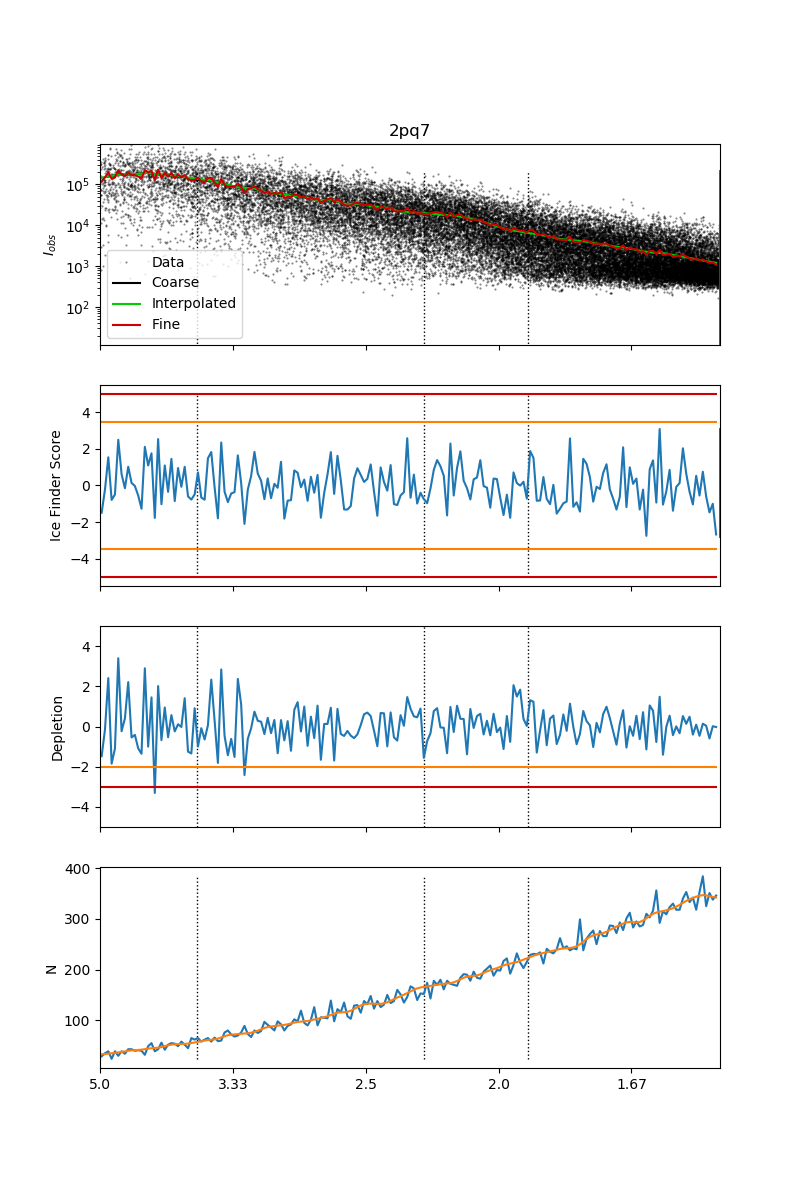

Supplement: Supplementary file 3 [file d-77-00540-sup3.zip › IceBiasingImages/2pq7.png]

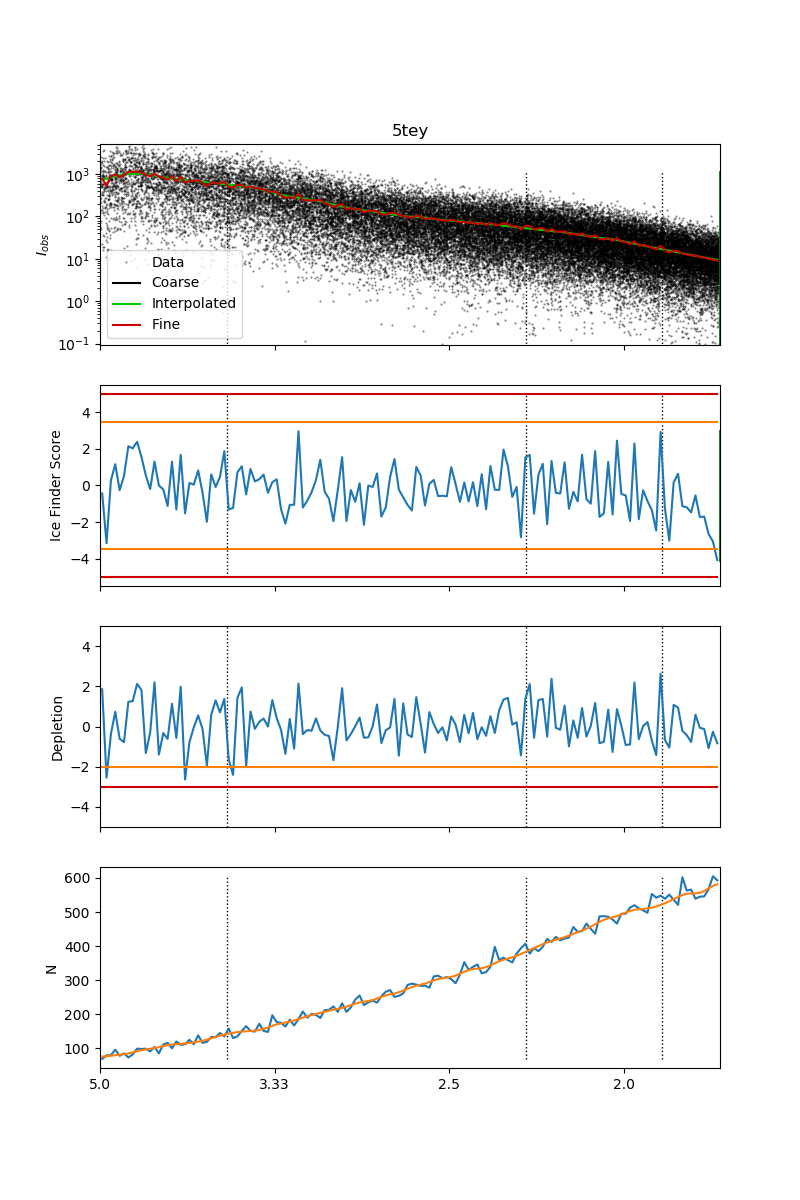

Supplement: Supplementary file 3 [file d-77-00540-sup3.zip › IceBiasingImages/5tey.png]

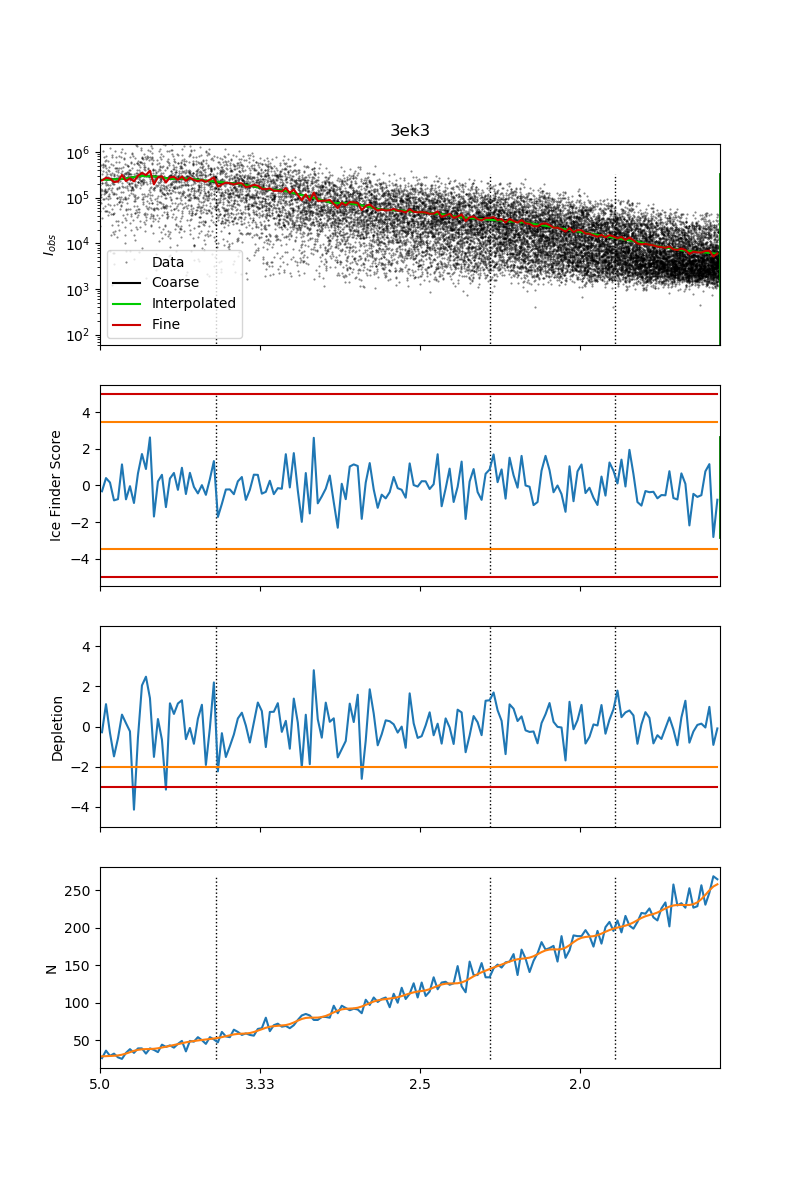

Supplement: Supplementary file 3 [file d-77-00540-sup3.zip › IceBiasingImages/3ek3.png]

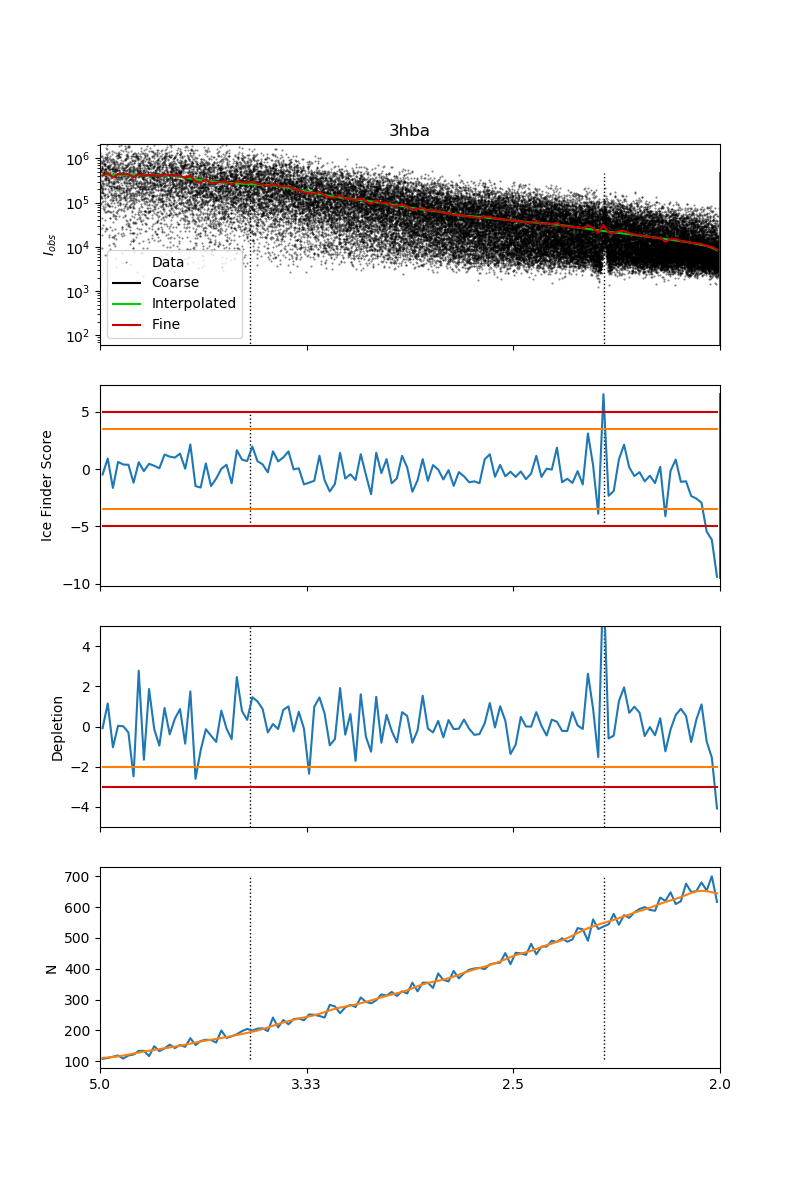

Supplement: Supplementary file 3 [file d-77-00540-sup3.zip › IceBiasingImages/3hba.png]

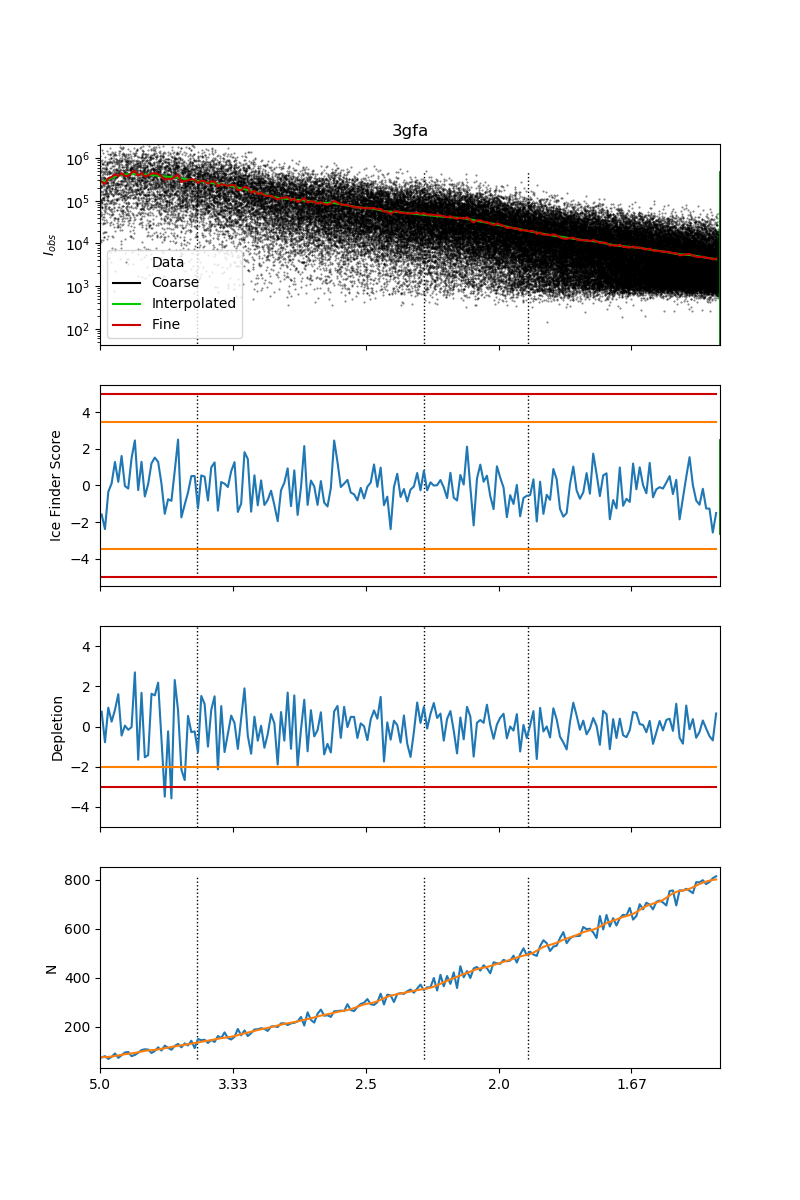

Supplement: Supplementary file 3 [file d-77-00540-sup3.zip › IceBiasingImages/3gfa.png]

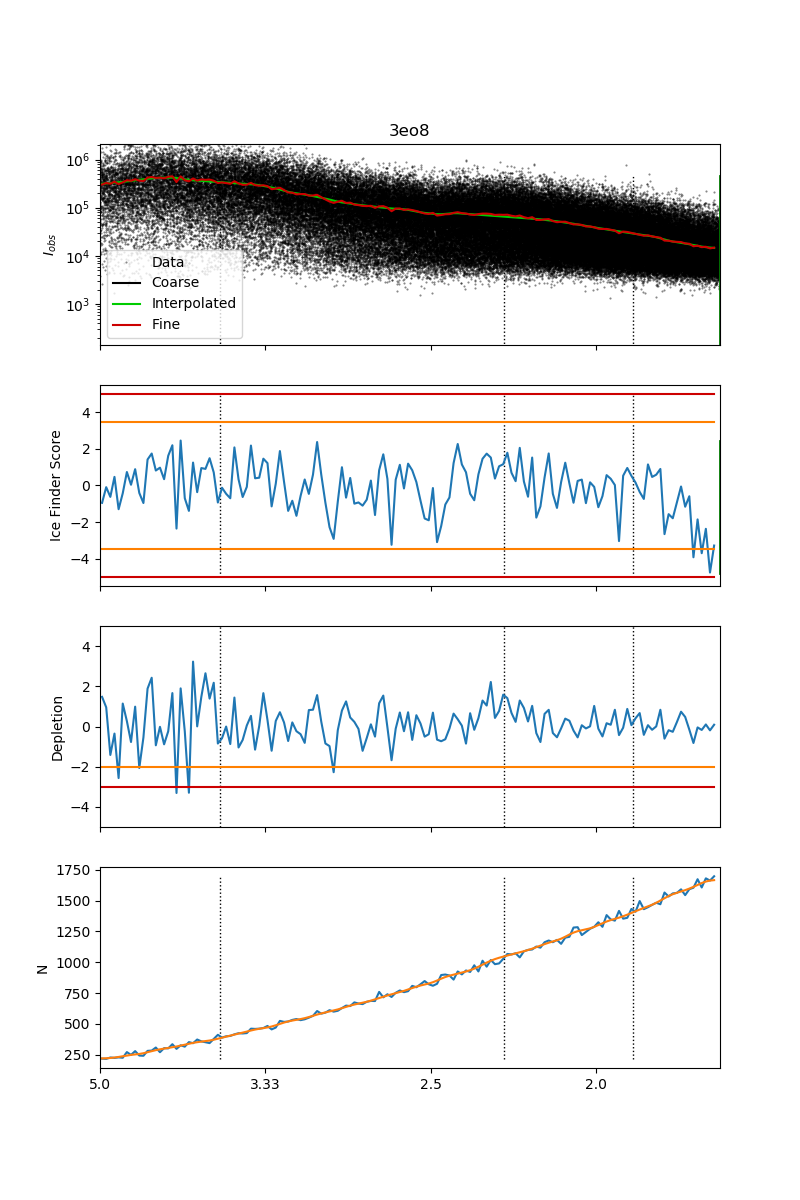

Supplement: Supplementary file 3 [file d-77-00540-sup3.zip › IceBiasingImages/3eo8.png]

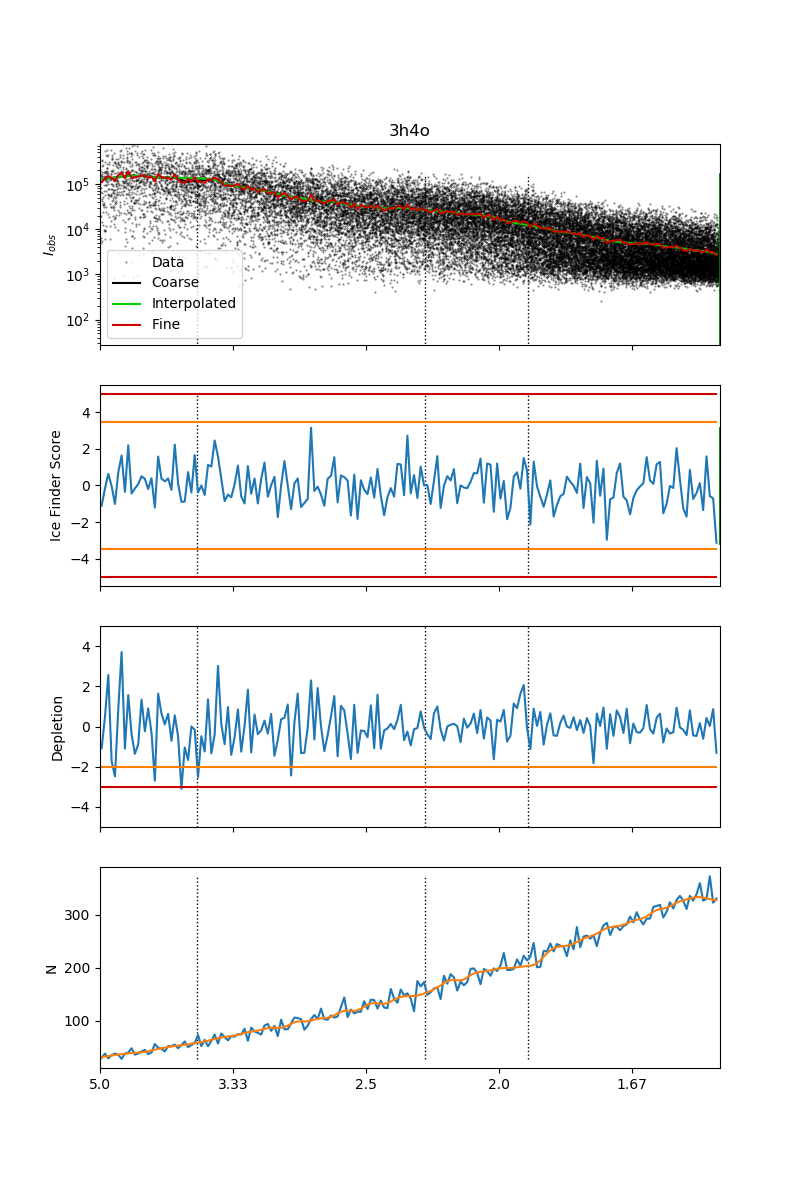

Supplement: Supplementary file 3 [file d-77-00540-sup3.zip › IceBiasingImages/3h4o.png]

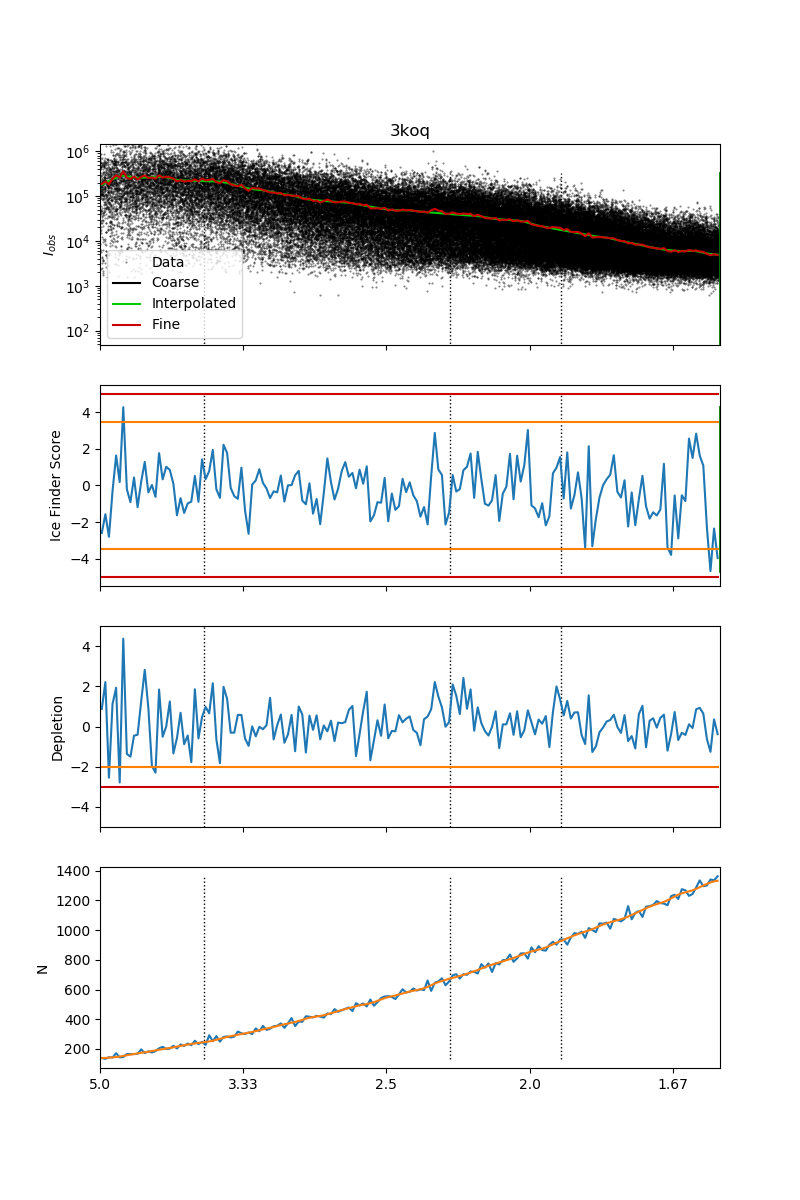

Supplement: Supplementary file 3 [file d-77-00540-sup3.zip › IceBiasingImages/3koq.png]

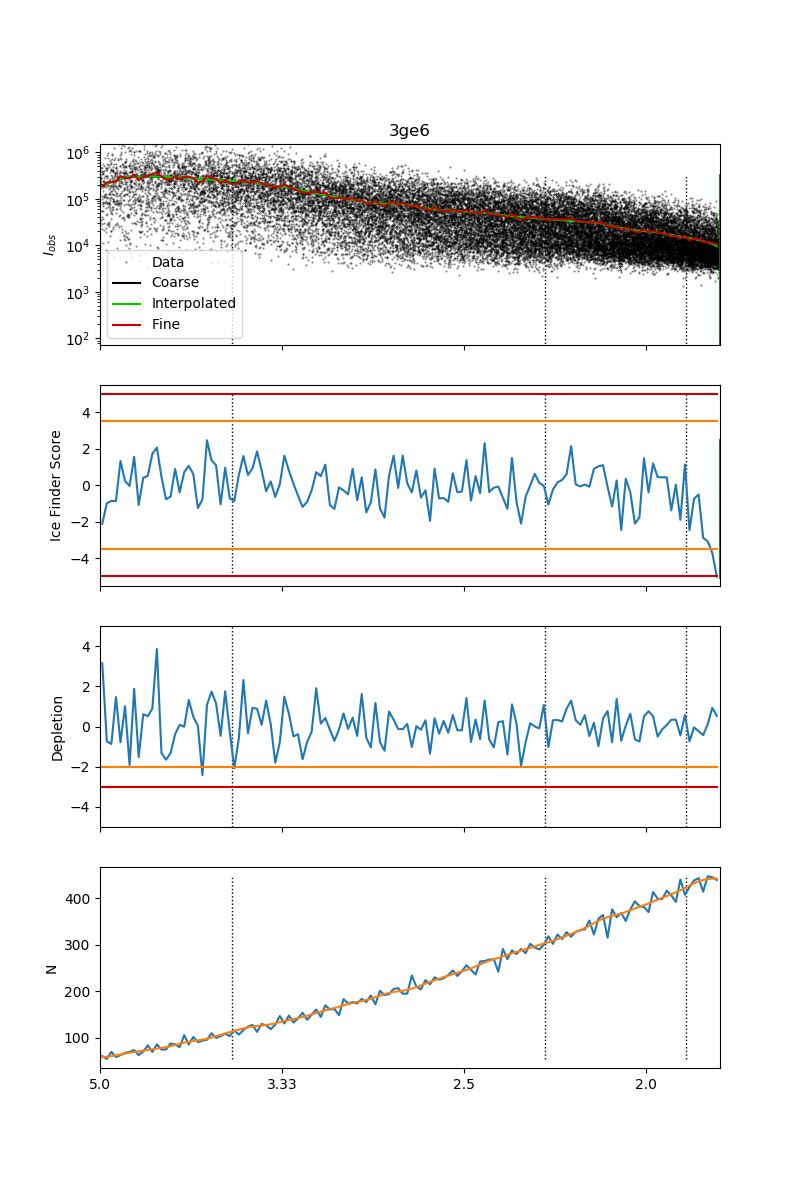

Supplement: Supplementary file 3 [file d-77-00540-sup3.zip › IceBiasingImages/3ge6.png]

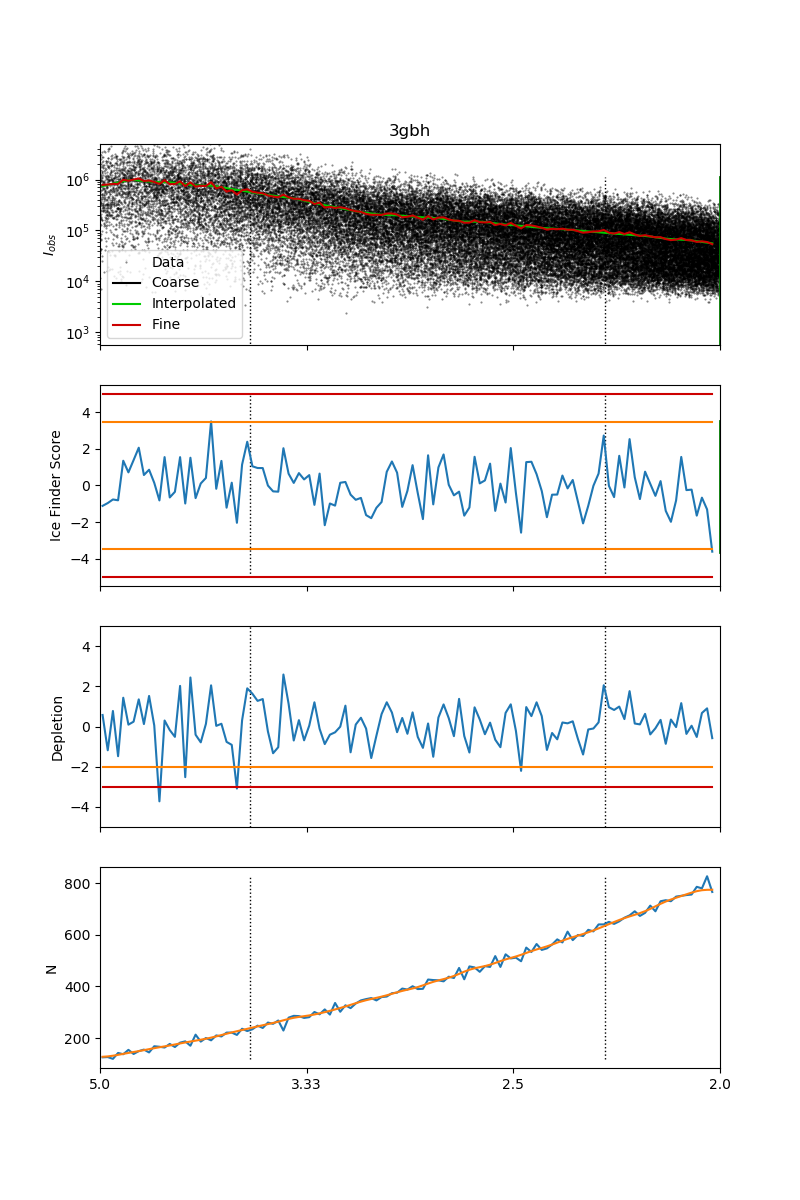

Supplement: Supplementary file 3 [file d-77-00540-sup3.zip › IceBiasingImages/3gbh.png]

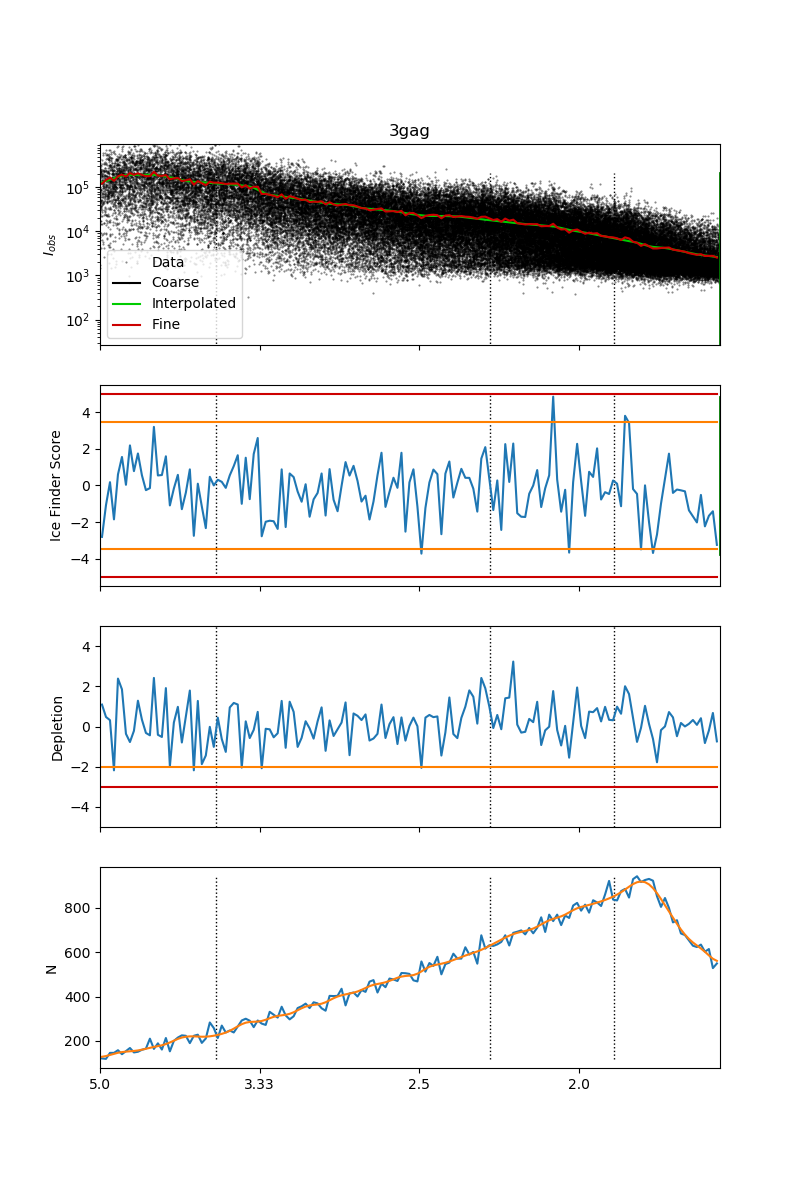

Supplement: Supplementary file 3 [file d-77-00540-sup3.zip › IceBiasingImages/3gag.png]

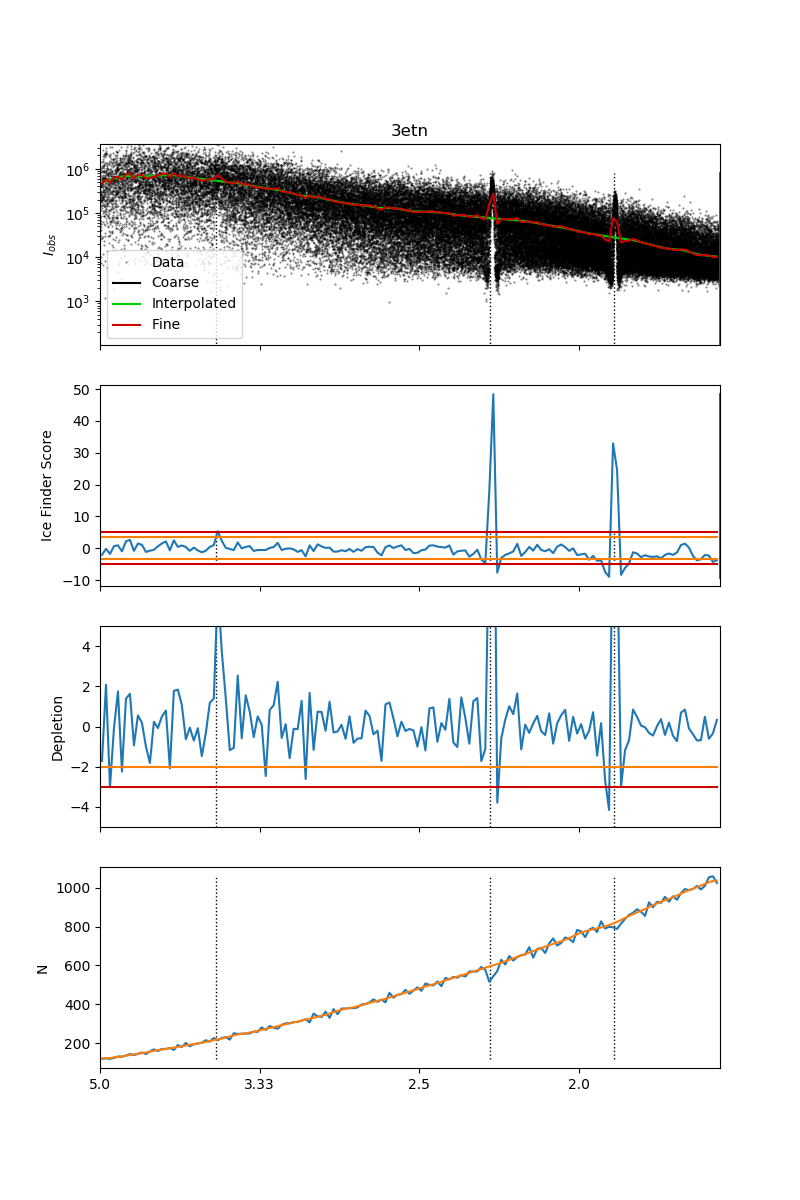

Supplement: Supplementary file 3 [file d-77-00540-sup3.zip › IceBiasingImages/3etn.png]

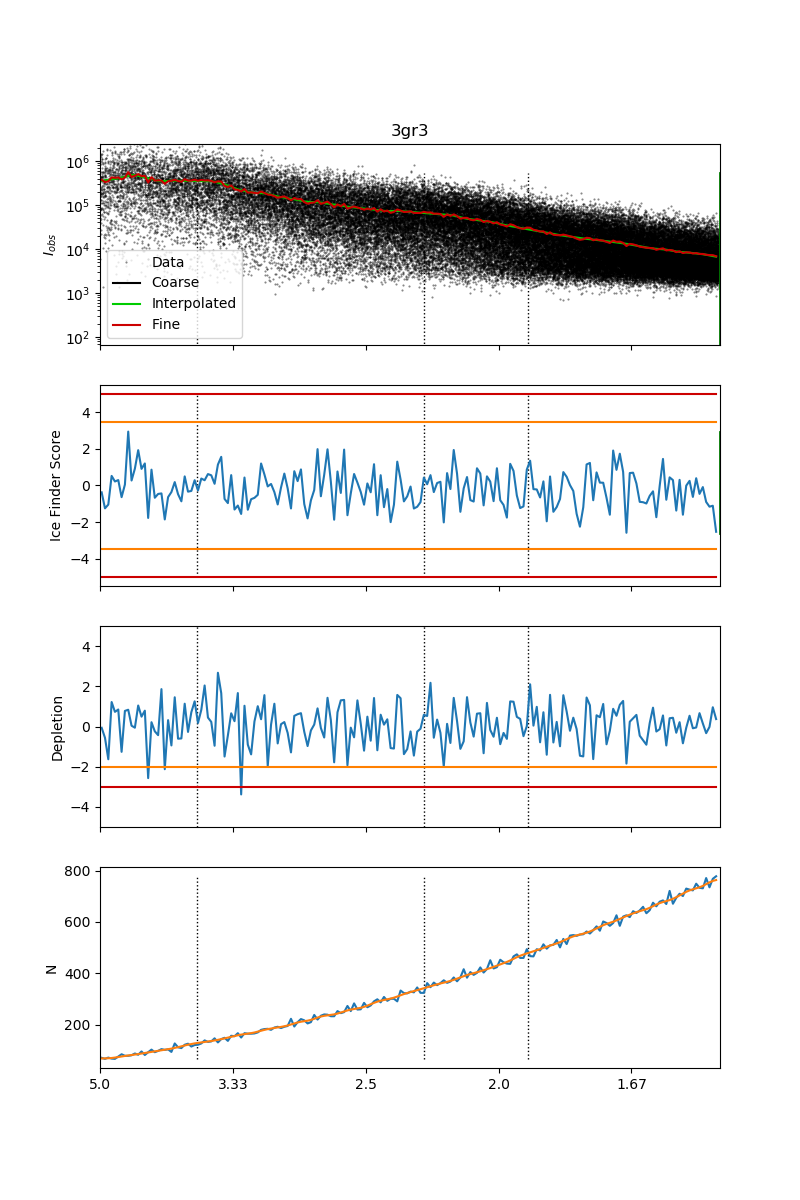

Supplement: Supplementary file 3 [file d-77-00540-sup3.zip › IceBiasingImages/3gr3.png]

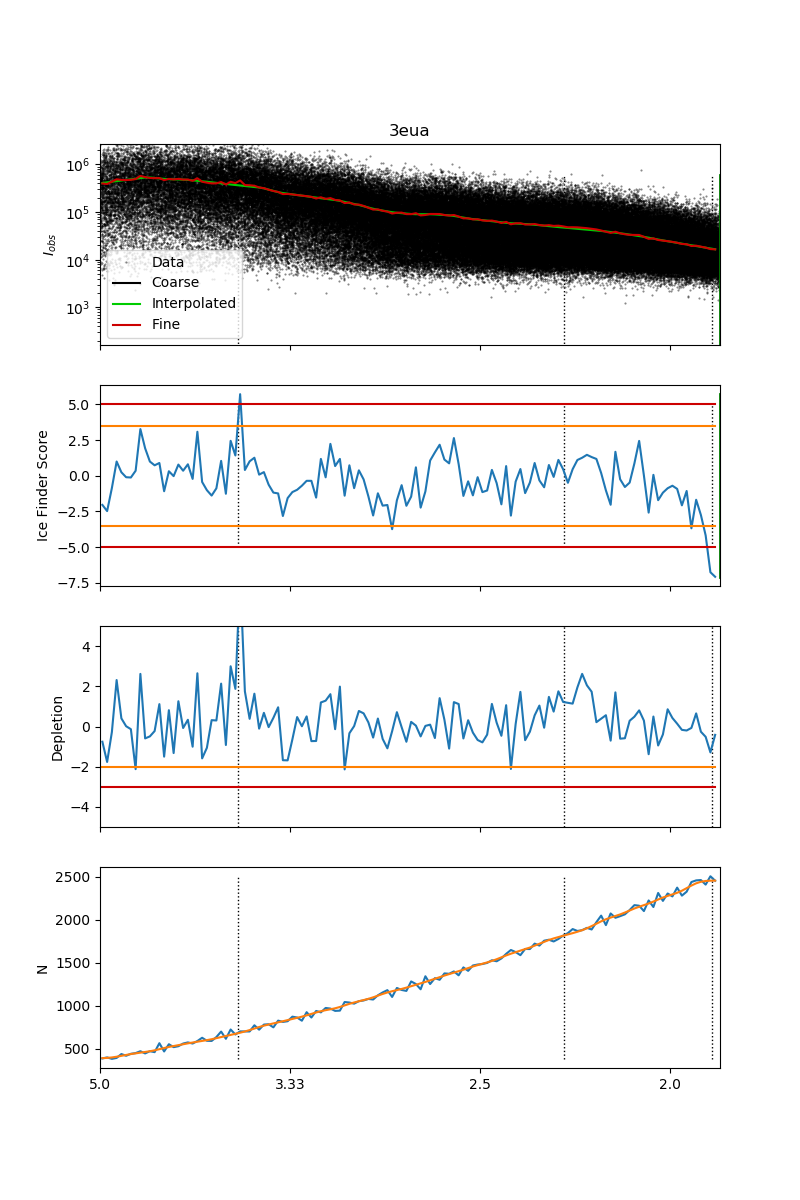

Supplement: Supplementary file 3 [file d-77-00540-sup3.zip › IceBiasingImages/3eua.png]

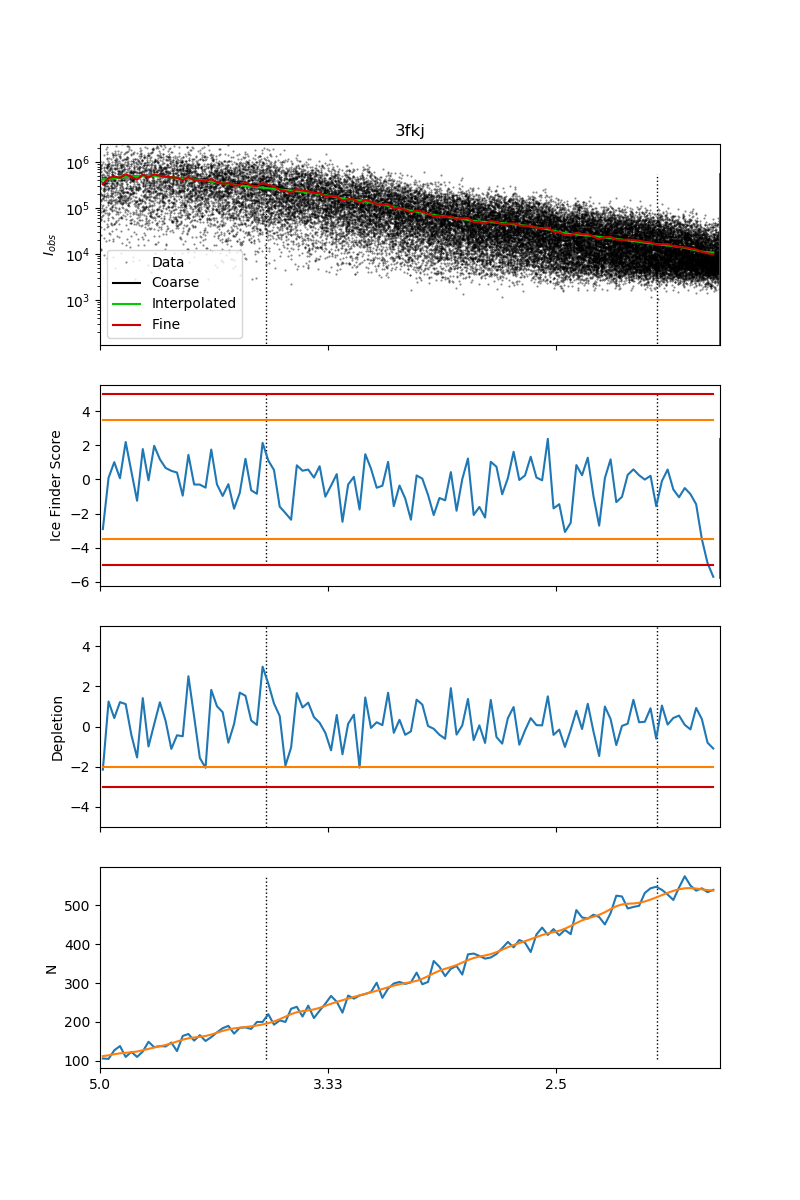

Supplement: Supplementary file 3 [file d-77-00540-sup3.zip › IceBiasingImages/3fkj.png]

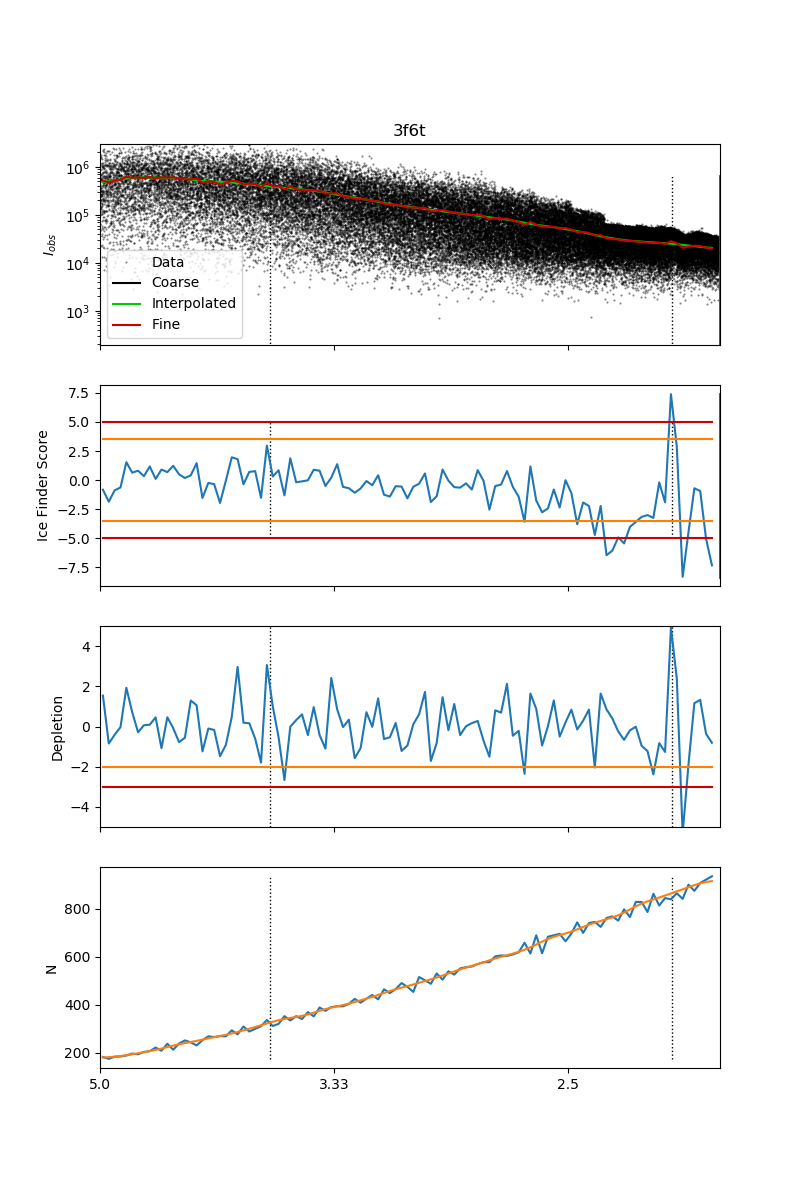

Supplement: Supplementary file 3 [file d-77-00540-sup3.zip › IceBiasingImages/3f6t.png]

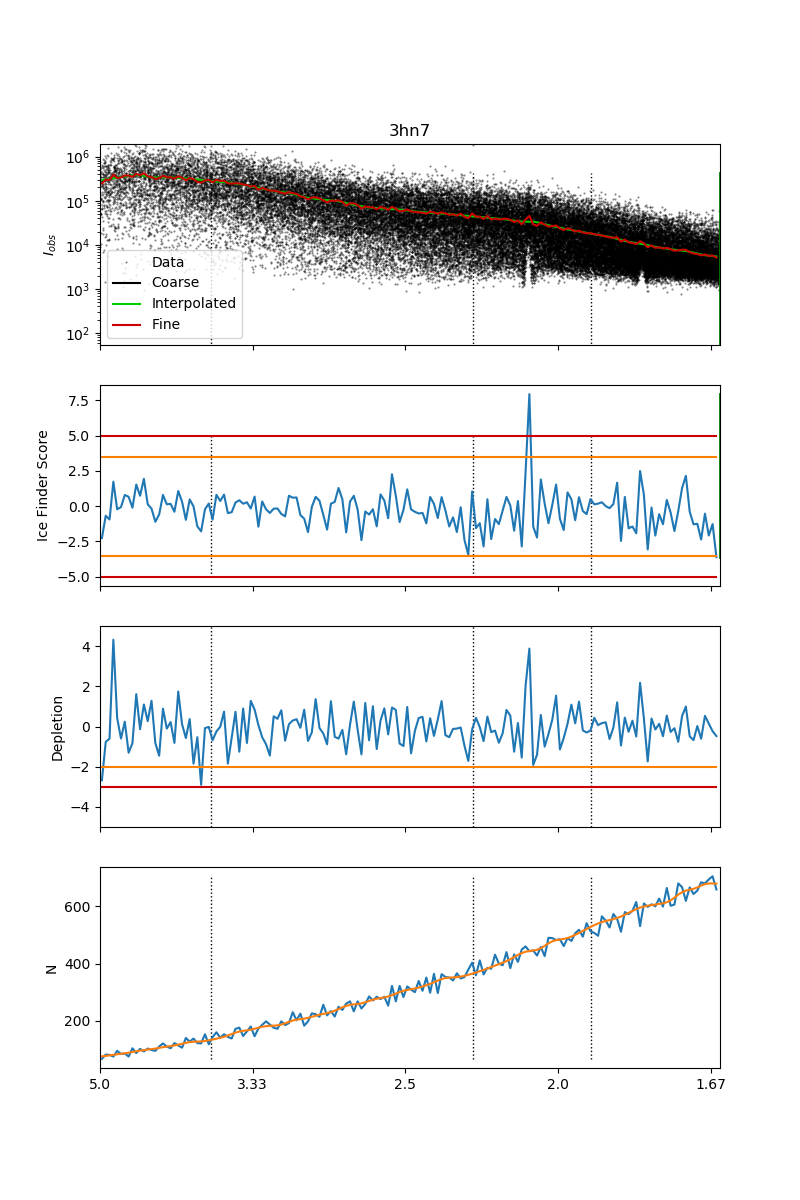

Supplement: Supplementary file 3 [file d-77-00540-sup3.zip › IceBiasingImages/3hn7.png]

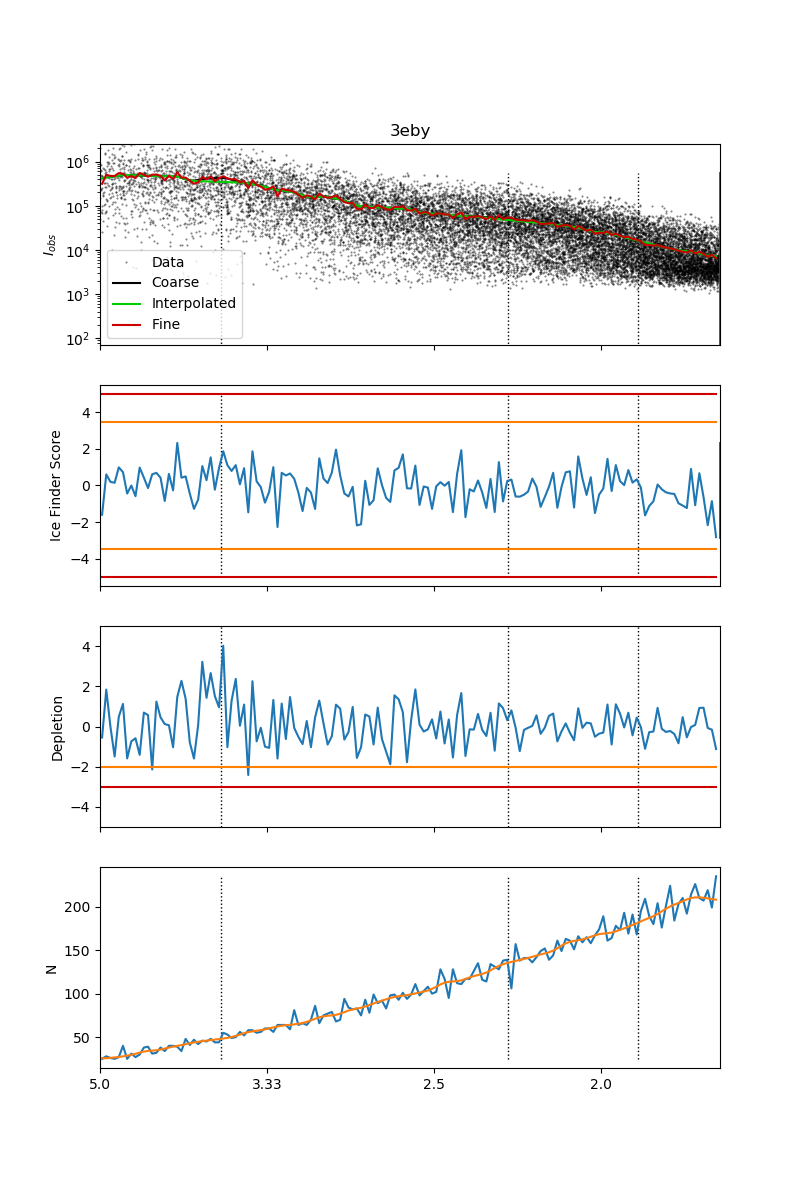

Supplement: Supplementary file 3 [file d-77-00540-sup3.zip › IceBiasingImages/3eby.png]

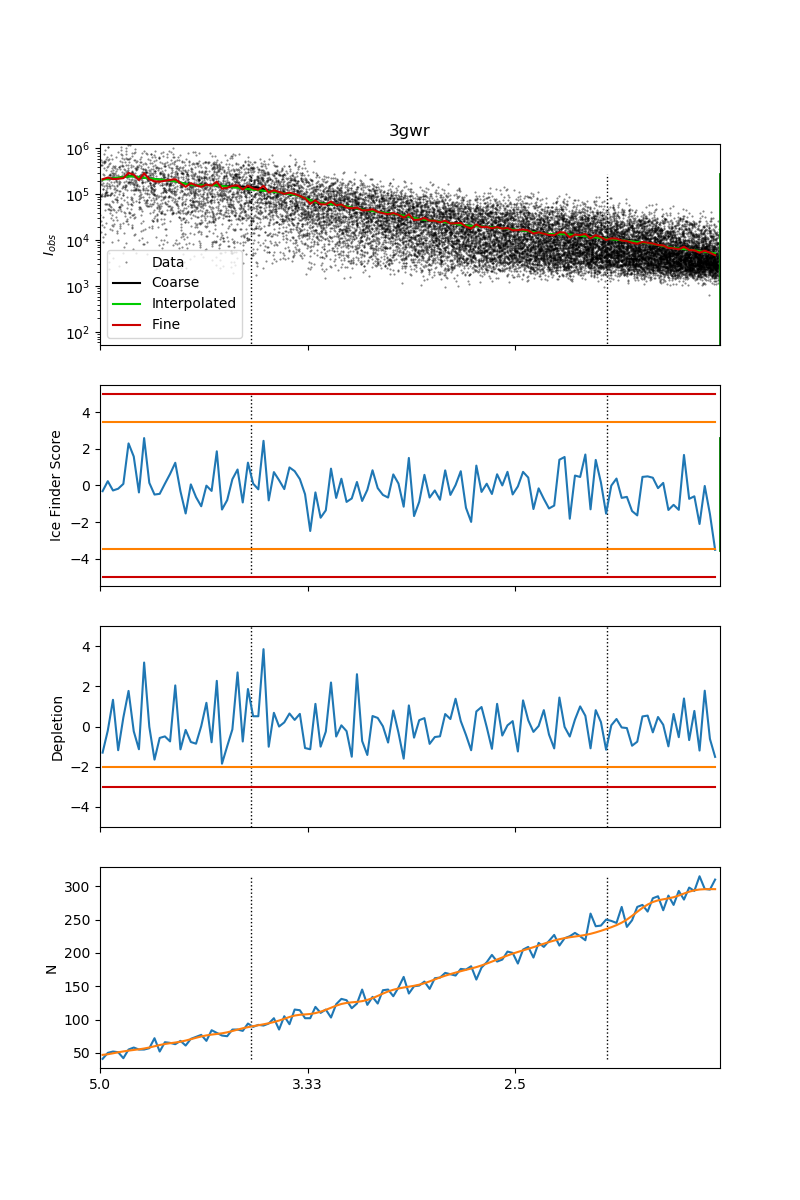

Supplement: Supplementary file 3 [file d-77-00540-sup3.zip › IceBiasingImages/3gwr.png]

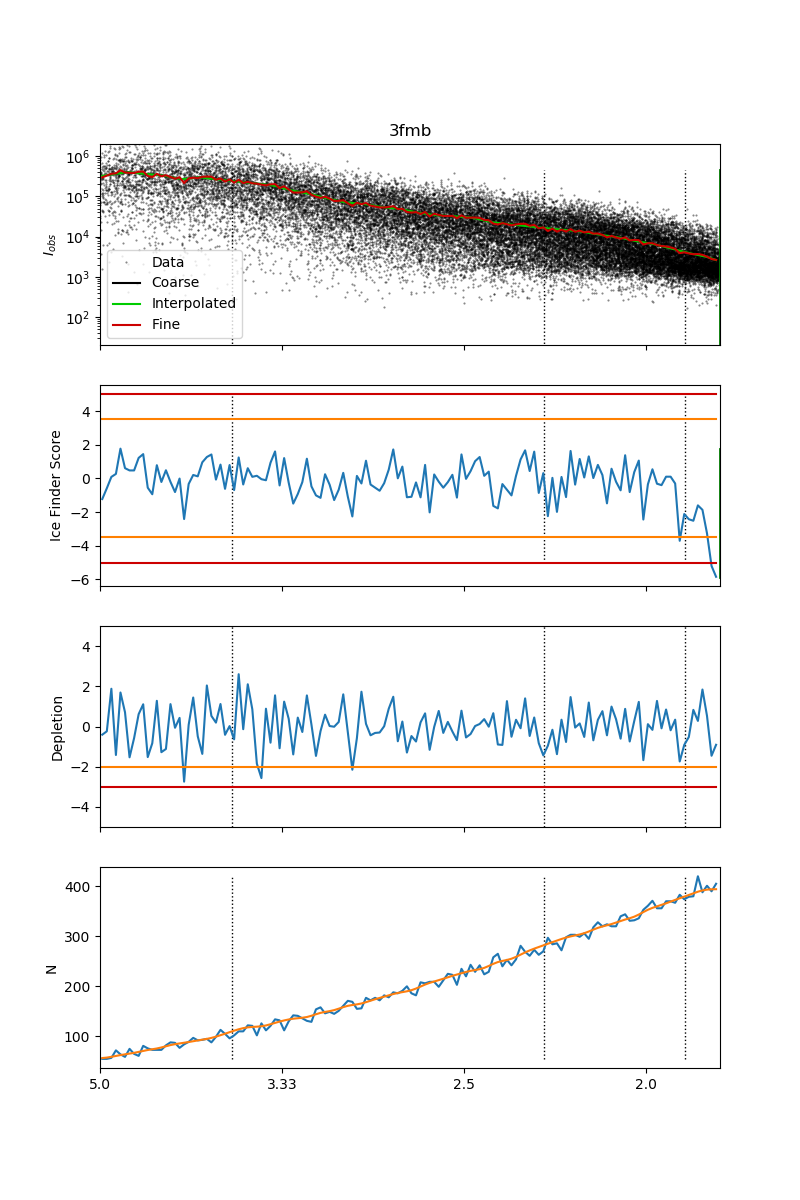

Supplement: Supplementary file 3 [file d-77-00540-sup3.zip › IceBiasingImages/3fmb.png]

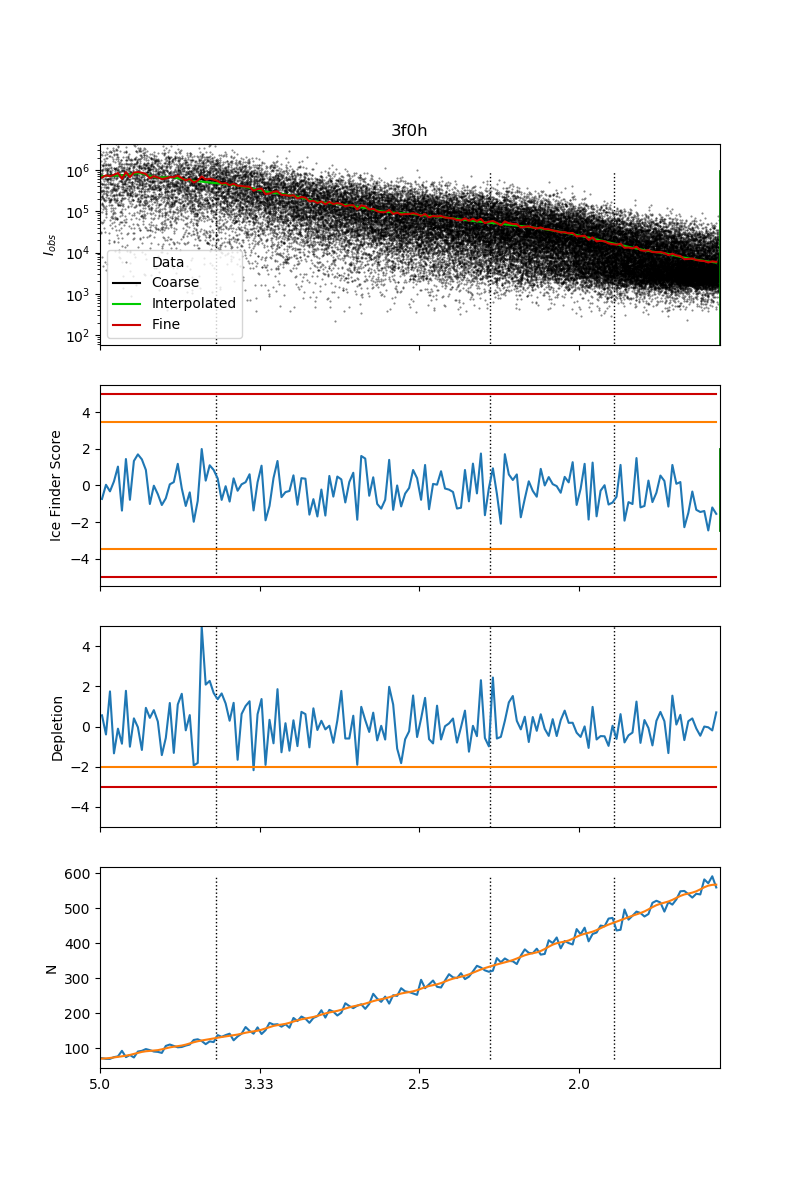

Supplement: Supplementary file 3 [file d-77-00540-sup3.zip › IceBiasingImages/3f0h.png]

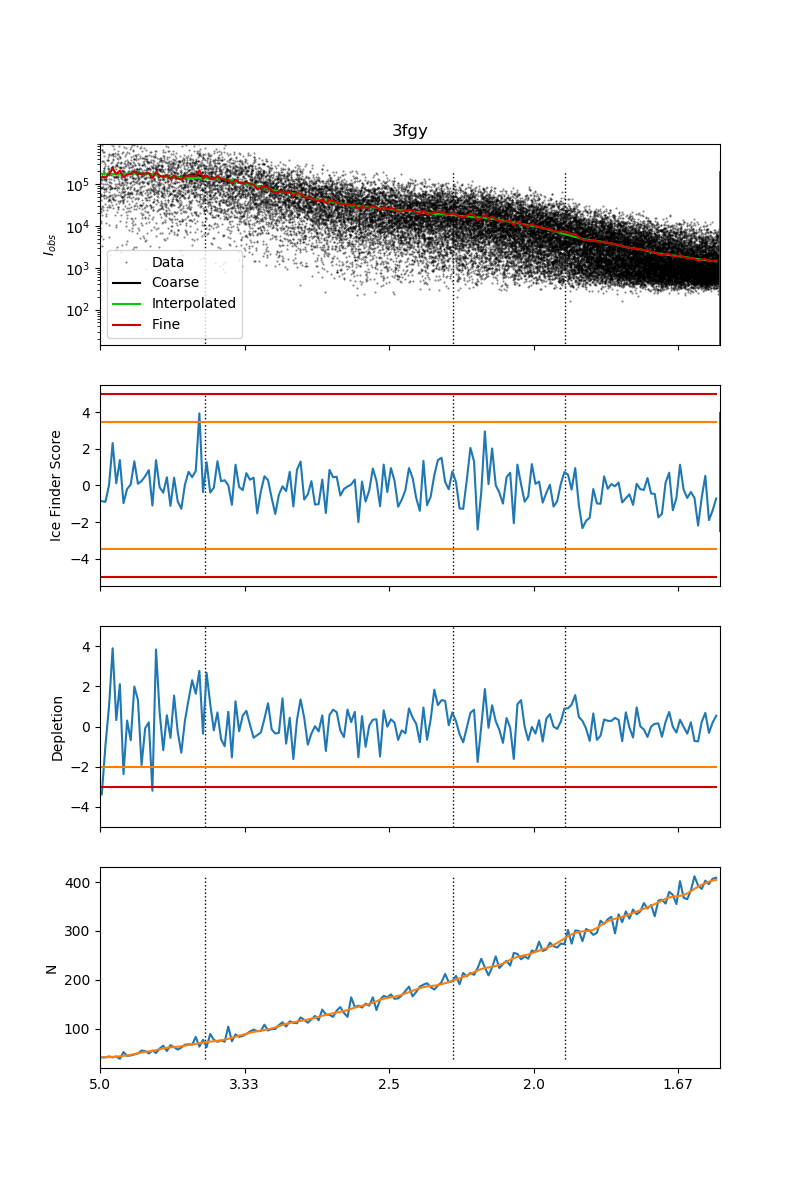

Supplement: Supplementary file 3 [file d-77-00540-sup3.zip › IceBiasingImages/3fgy.png]

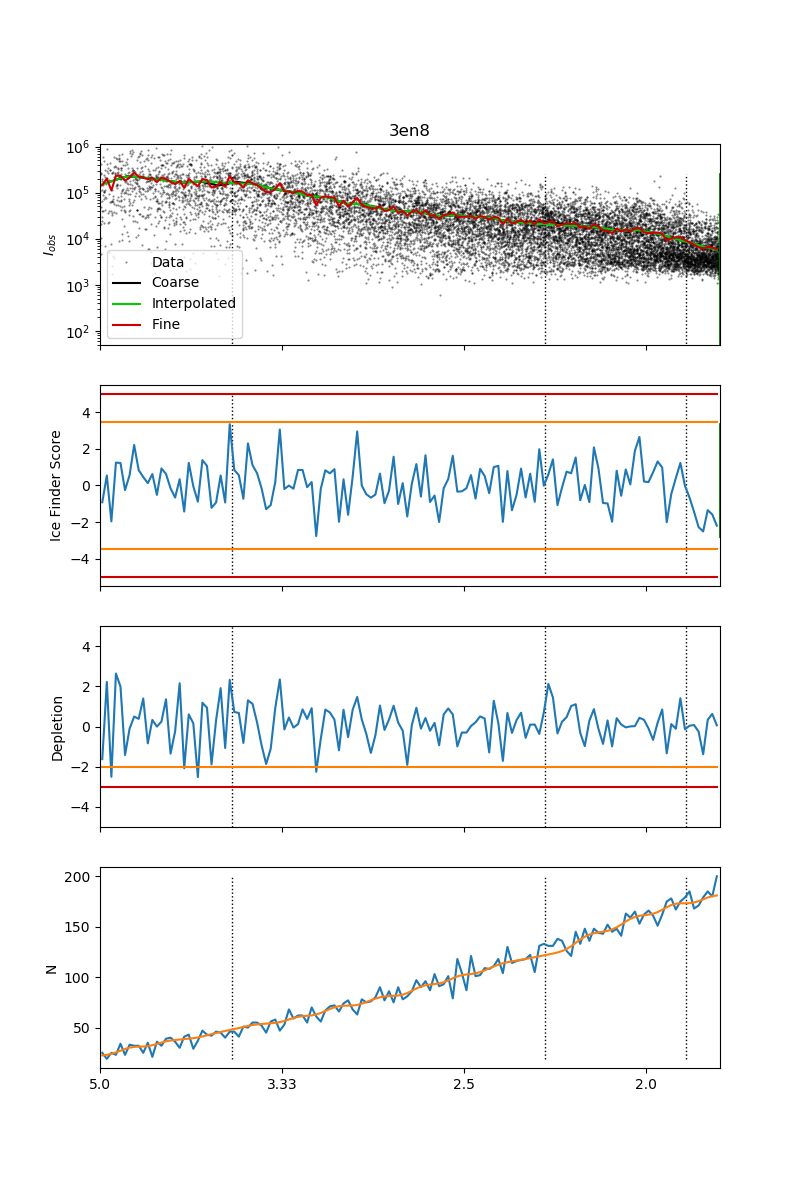

Supplement: Supplementary file 3 [file d-77-00540-sup3.zip › IceBiasingImages/3en8.png]

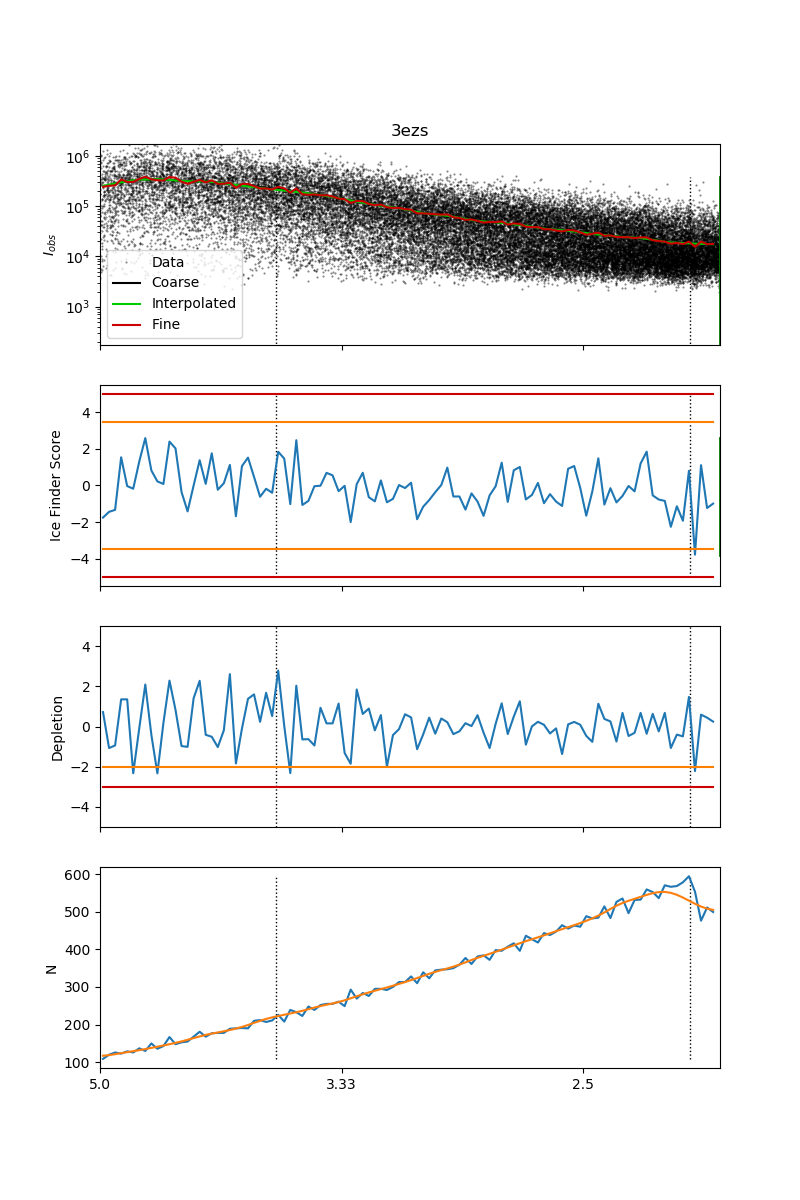

Supplement: Supplementary file 3 [file d-77-00540-sup3.zip › IceBiasingImages/3ezs.png]

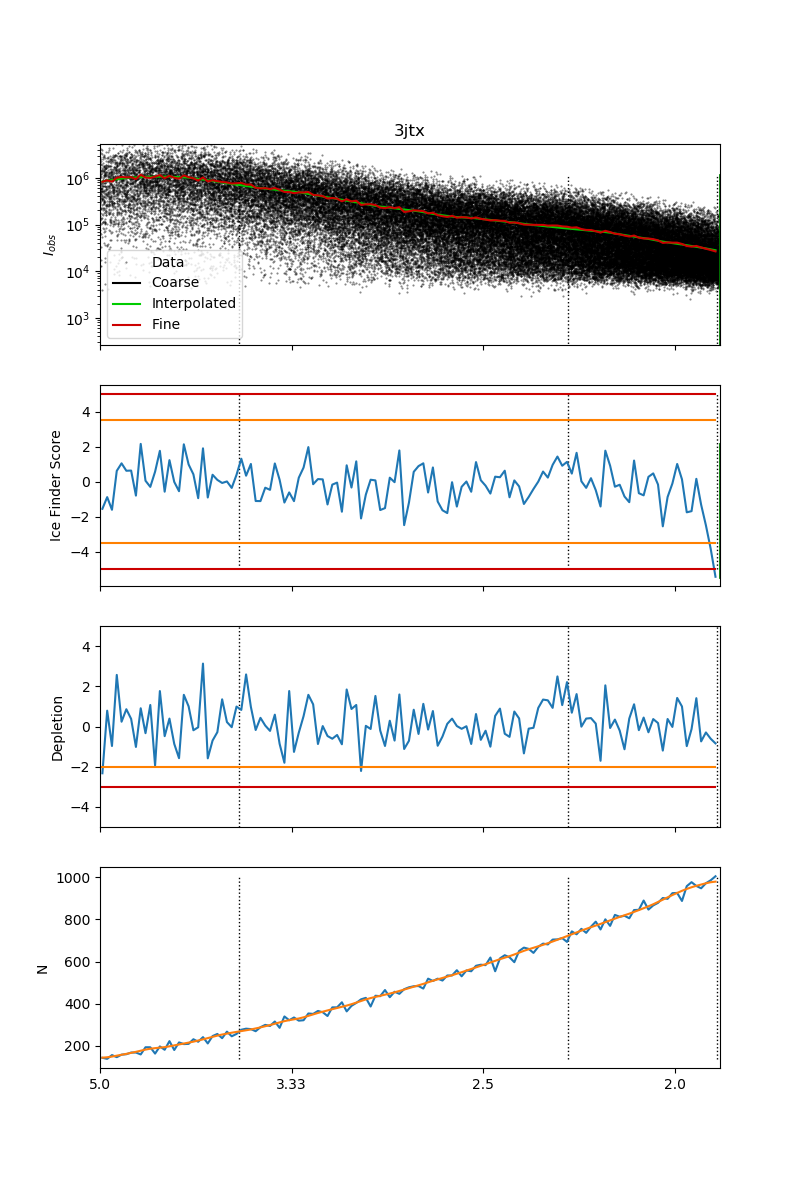

Supplement: Supplementary file 3 [file d-77-00540-sup3.zip › IceBiasingImages/3jtx.png]

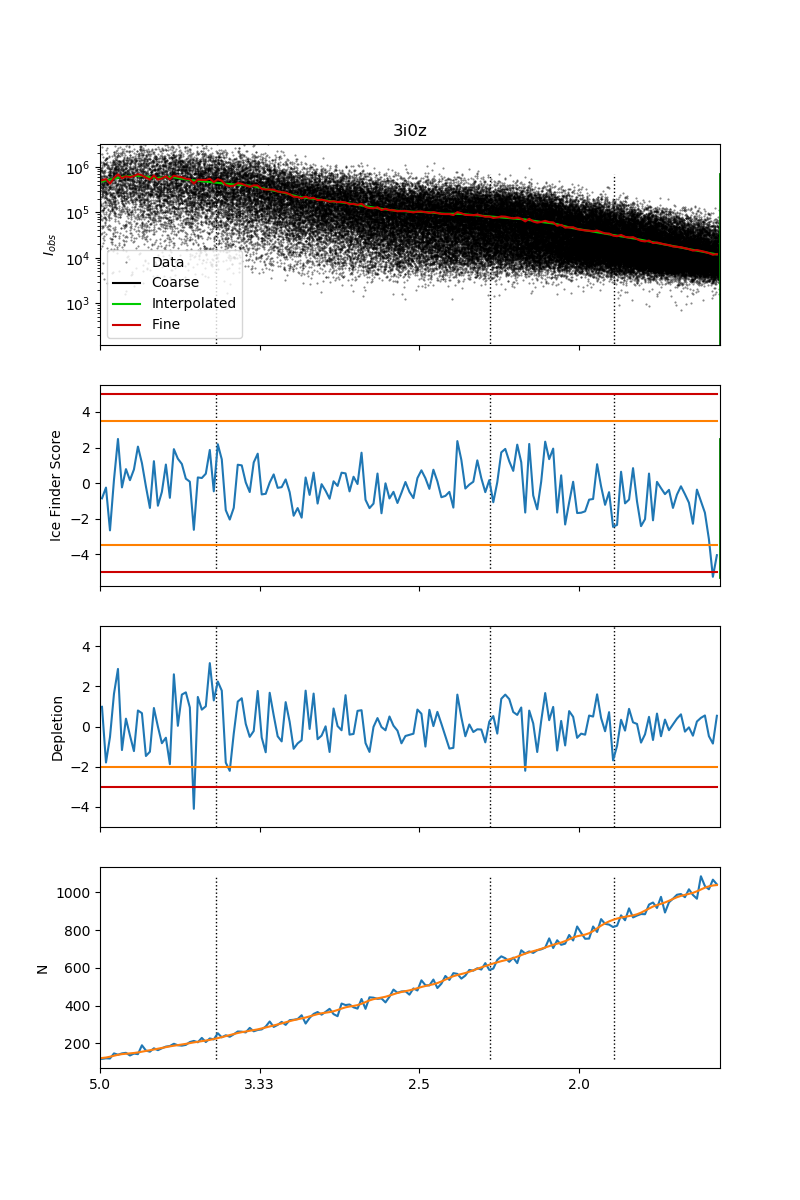

Supplement: Supplementary file 3 [file d-77-00540-sup3.zip › IceBiasingImages/3i0z.png]

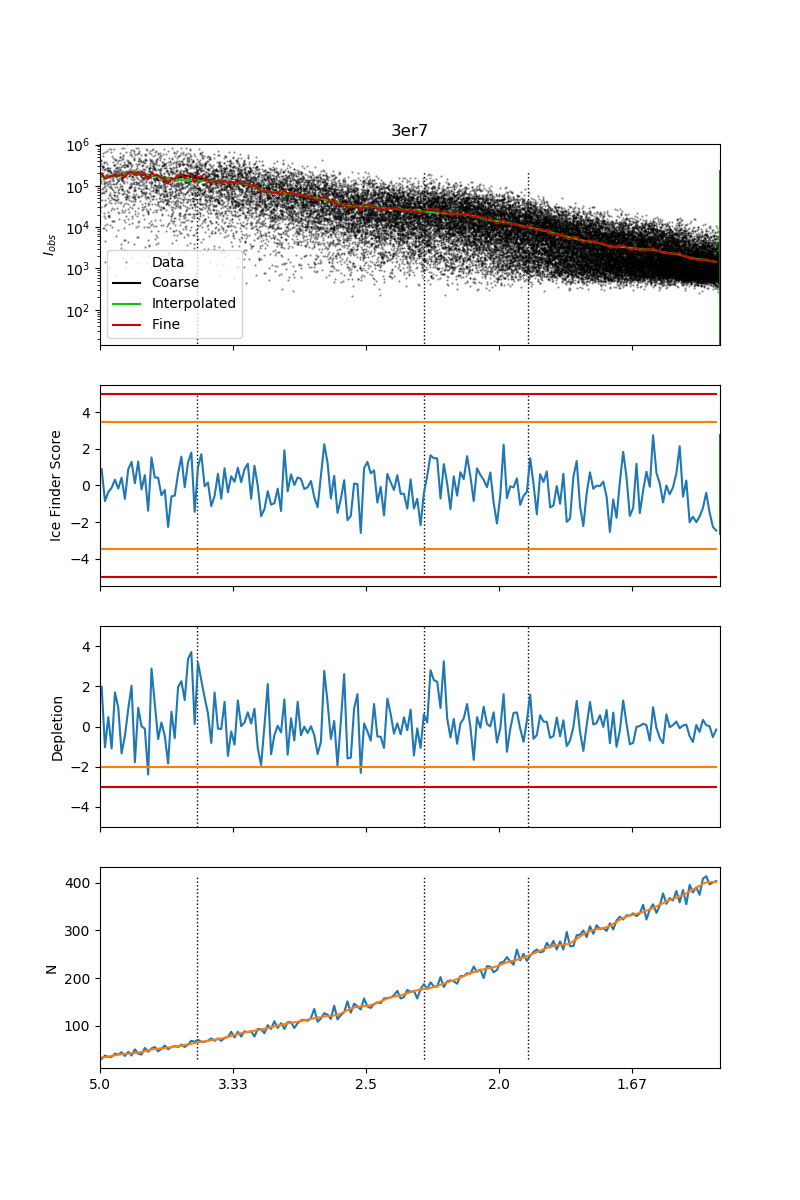

Supplement: Supplementary file 3 [file d-77-00540-sup3.zip › IceBiasingImages/3er7.png]

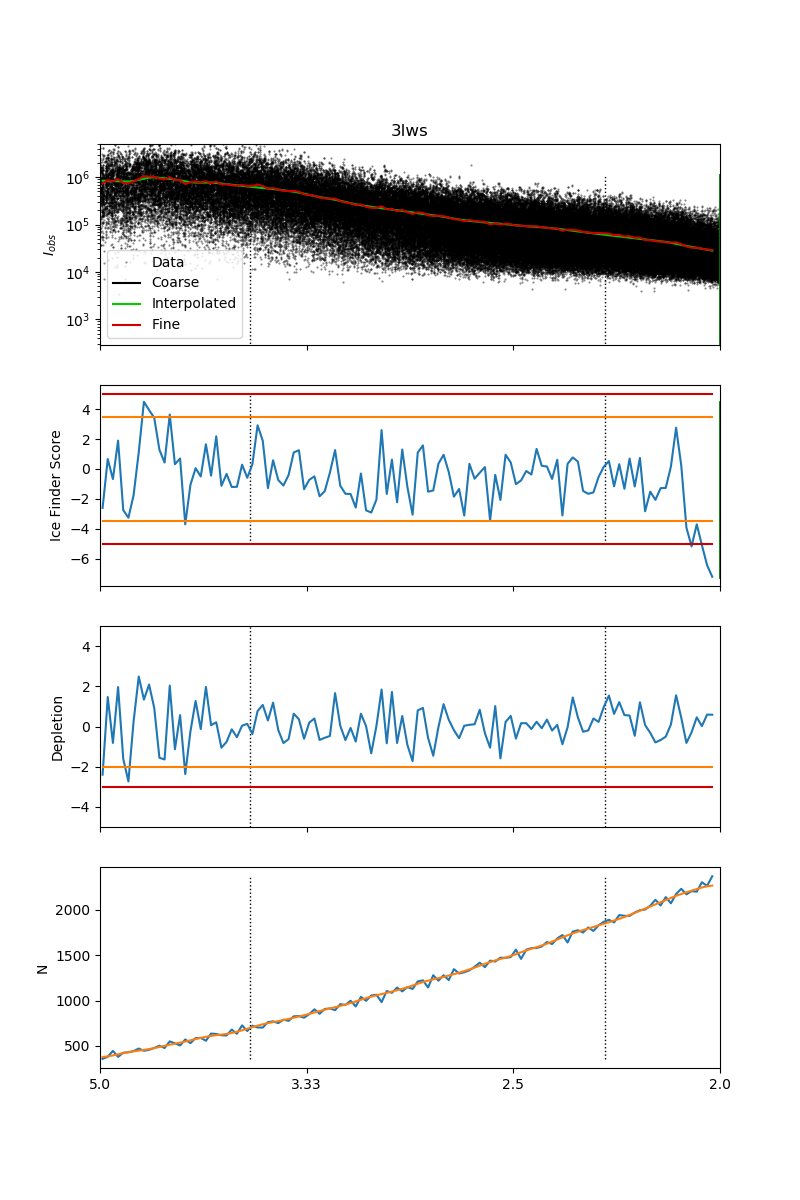

Supplement: Supplementary file 3 [file d-77-00540-sup3.zip › IceBiasingImages/3lws.png]

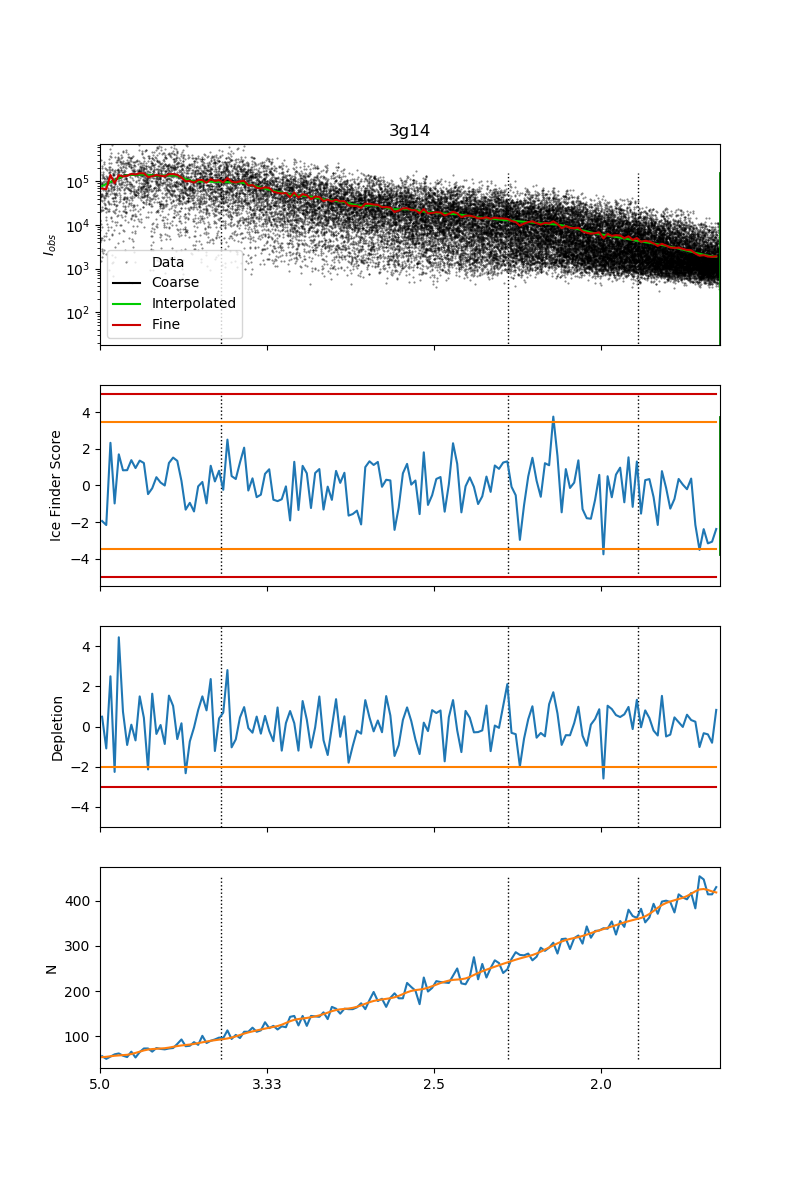

Supplement: Supplementary file 3 [file d-77-00540-sup3.zip › IceBiasingImages/3g14.png]

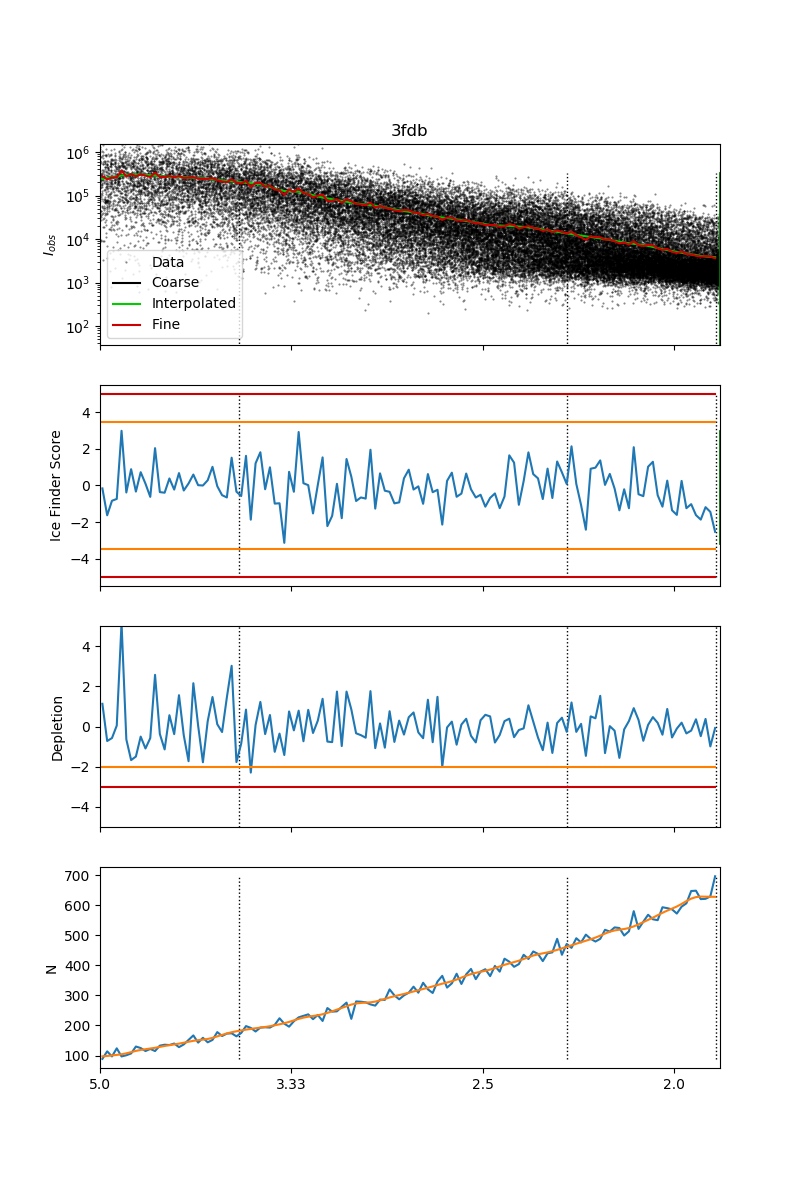

Supplement: Supplementary file 3 [file d-77-00540-sup3.zip › IceBiasingImages/3fdb.png]

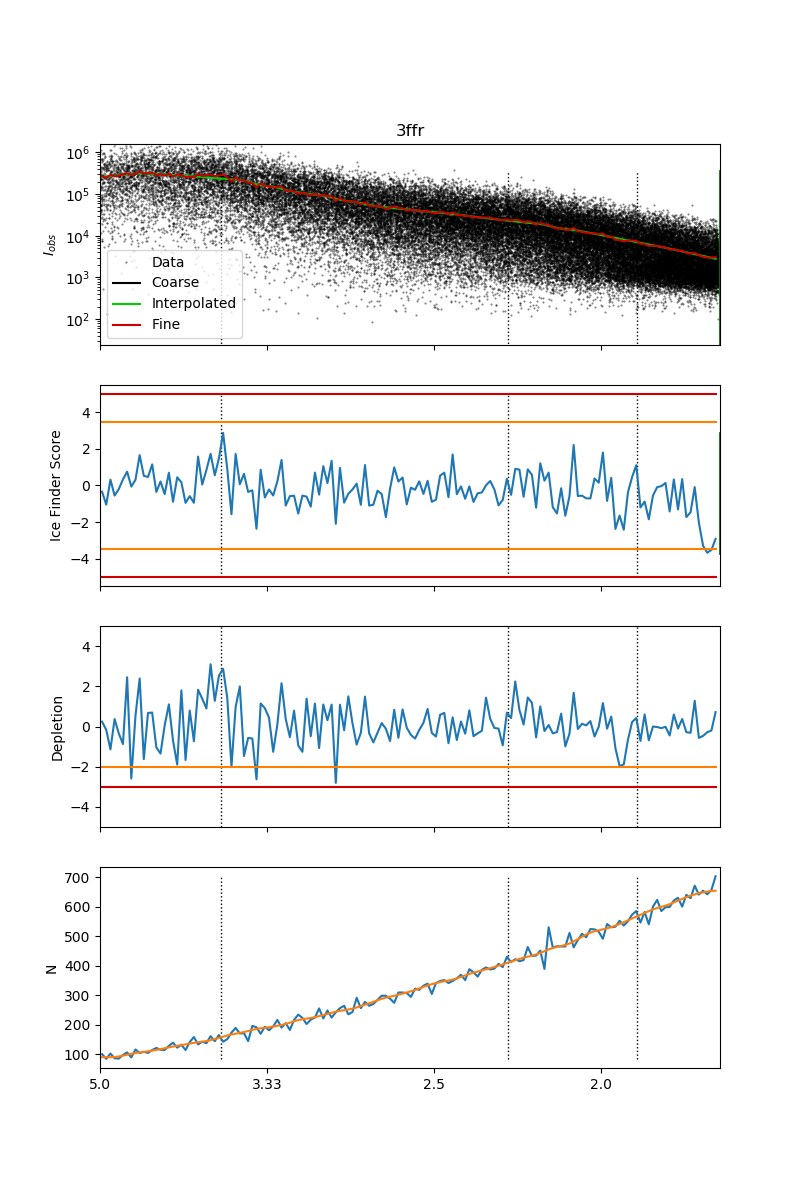

Supplement: Supplementary file 3 [file d-77-00540-sup3.zip › IceBiasingImages/3ffr.png]

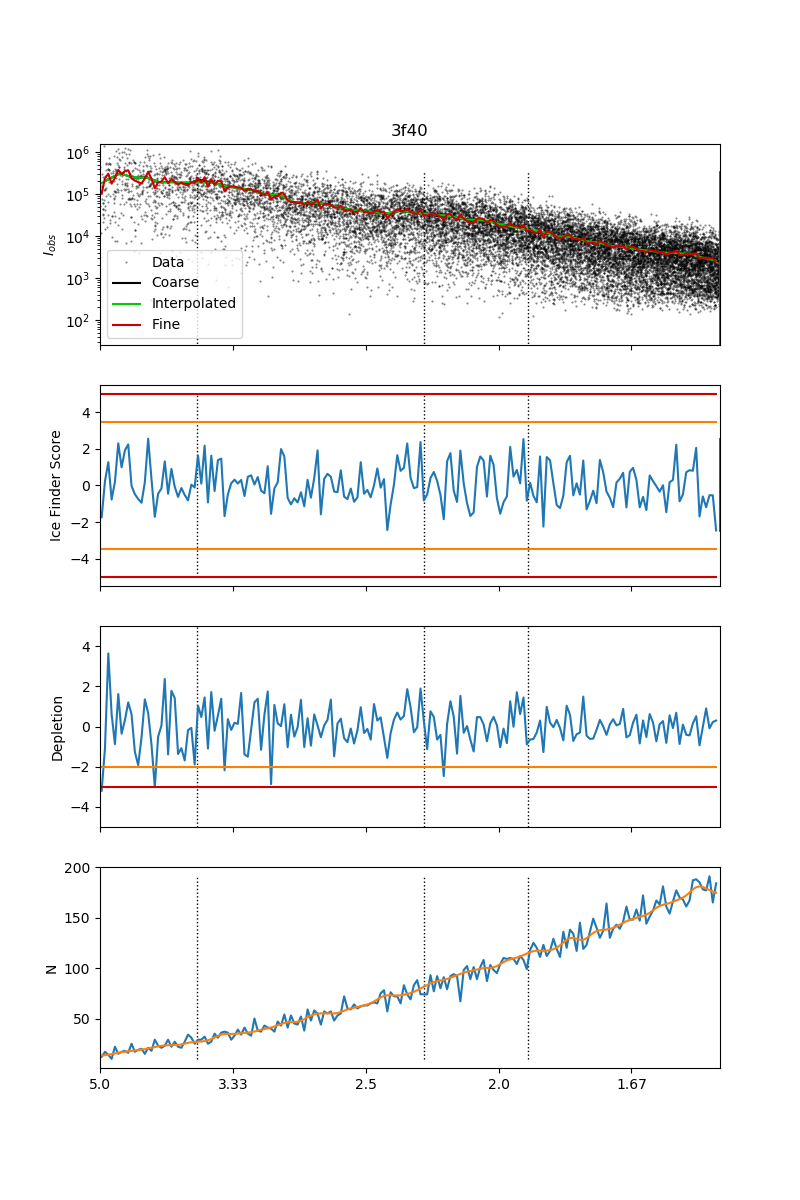

Supplement: Supplementary file 3 [file d-77-00540-sup3.zip › IceBiasingImages/3f40.png]

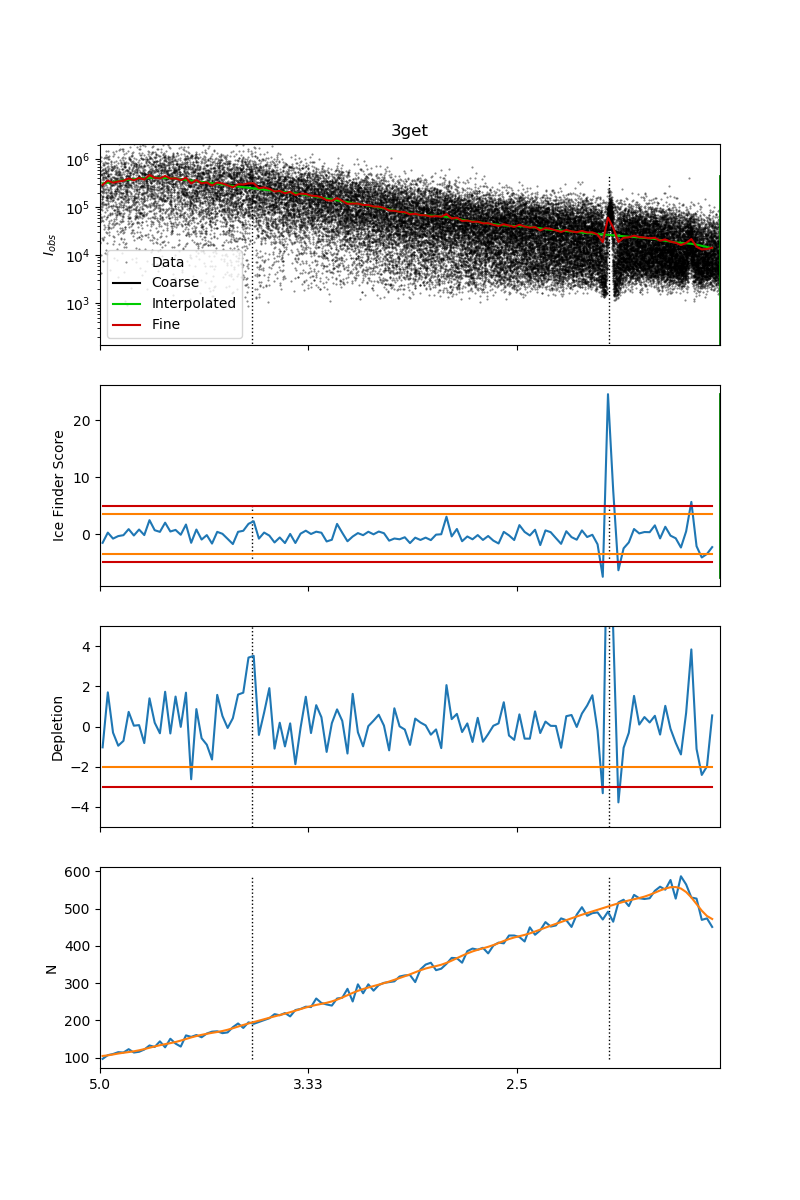

Supplement: Supplementary file 3 [file d-77-00540-sup3.zip › IceBiasingImages/3get.png]

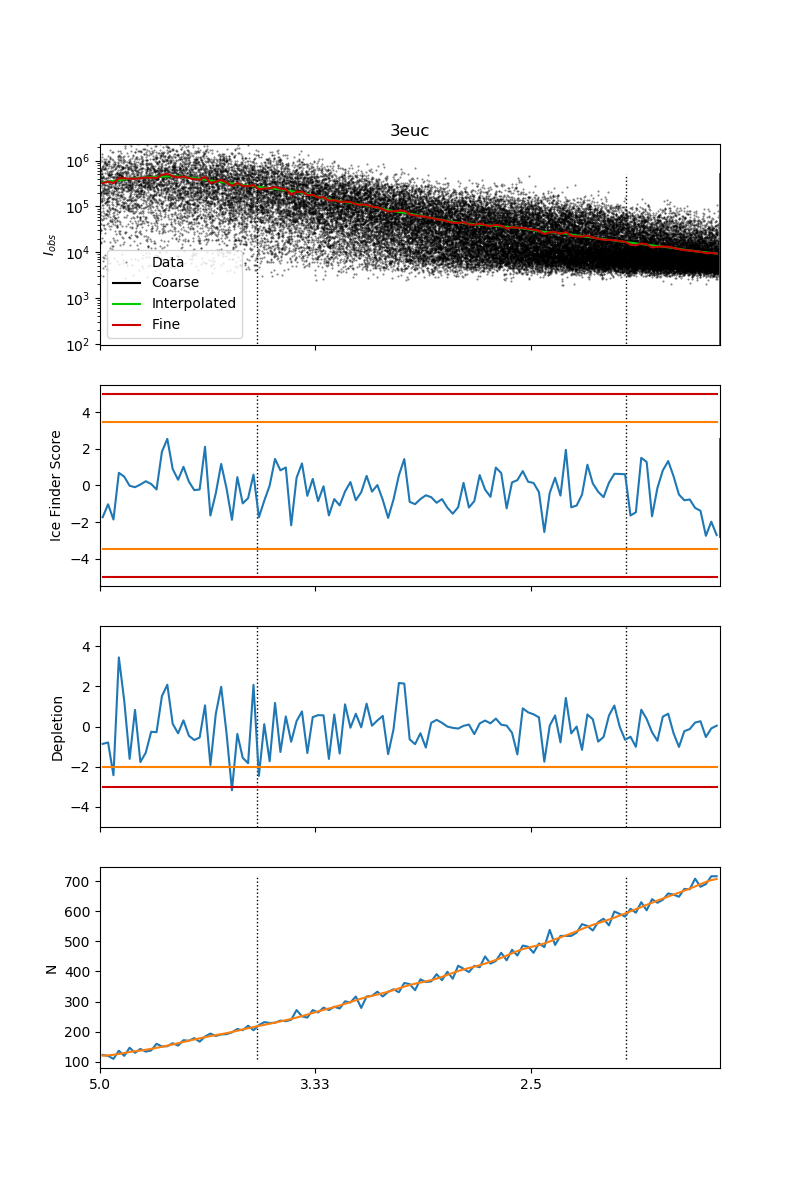

Supplement: Supplementary file 3 [file d-77-00540-sup3.zip › IceBiasingImages/3euc.png]

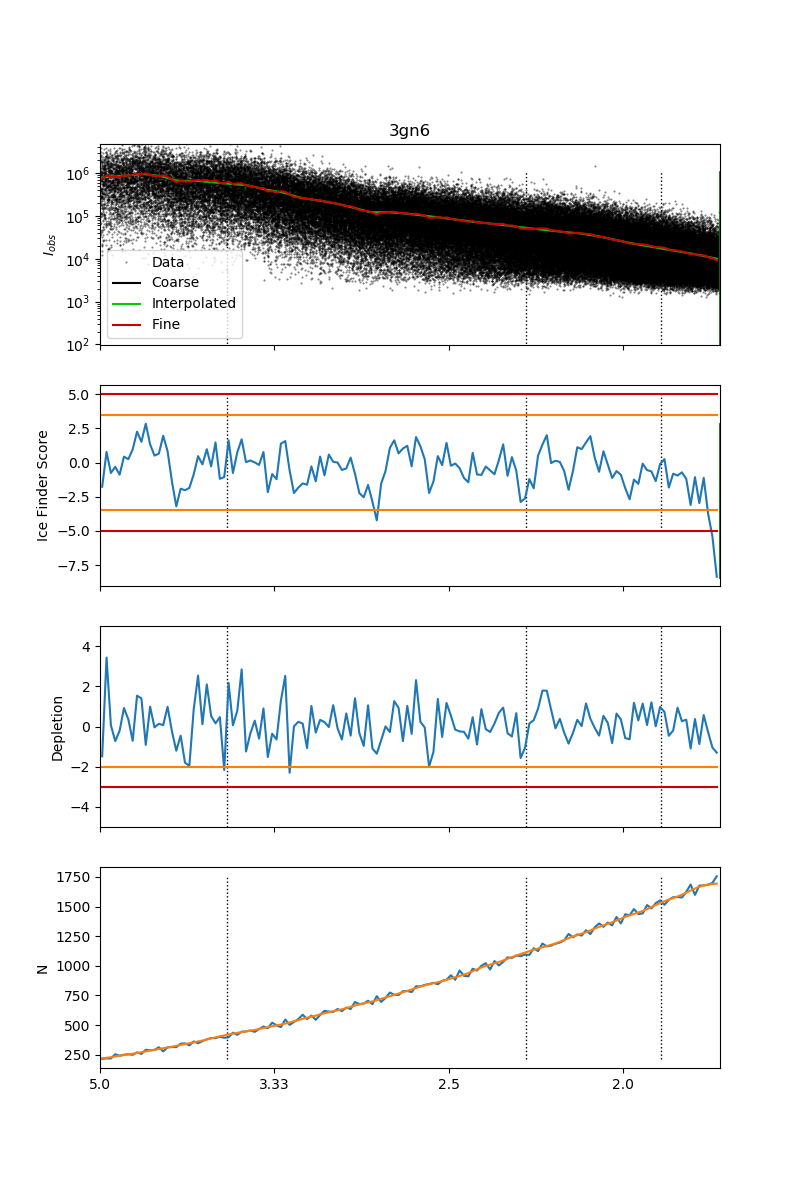

Supplement: Supplementary file 3 [file d-77-00540-sup3.zip › IceBiasingImages/3gn6.png]

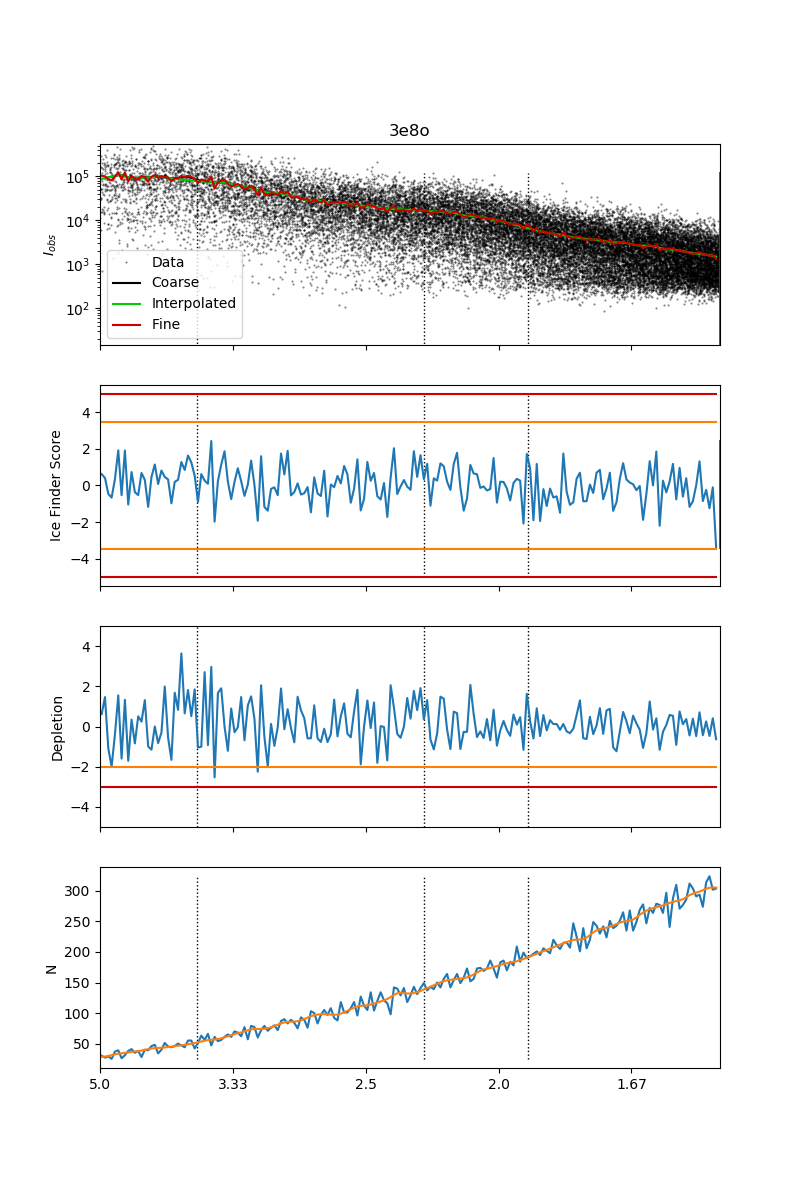

Supplement: Supplementary file 3 [file d-77-00540-sup3.zip › IceBiasingImages/3e8o.png]

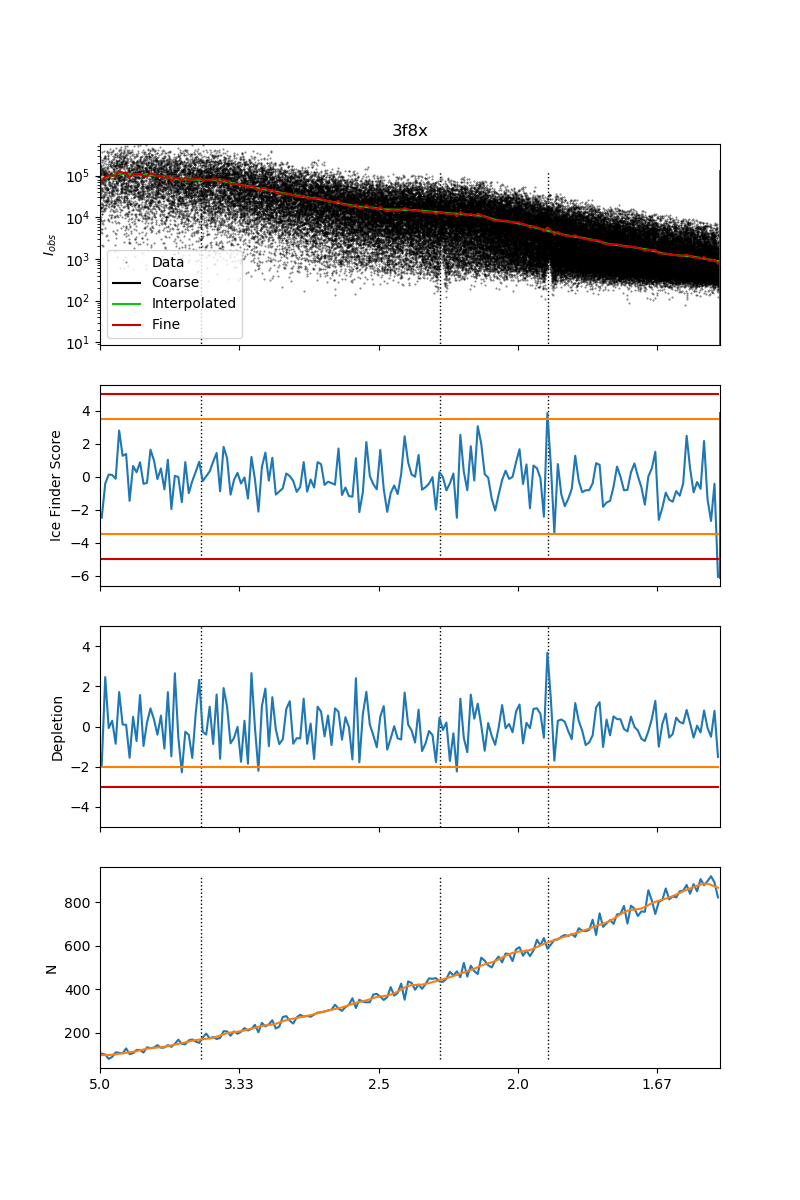

Supplement: Supplementary file 3 [file d-77-00540-sup3.zip › IceBiasingImages/3f8x.png]

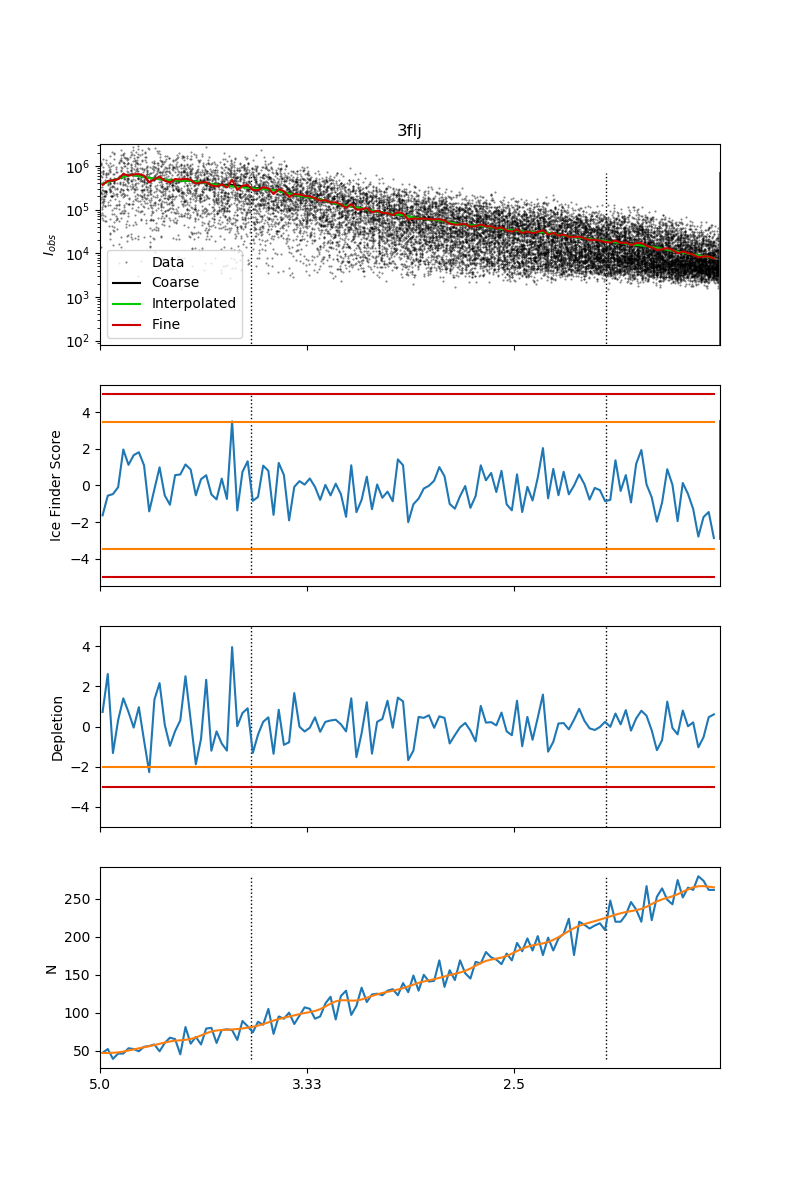

Supplement: Supplementary file 3 [file d-77-00540-sup3.zip › IceBiasingImages/3flj.png]

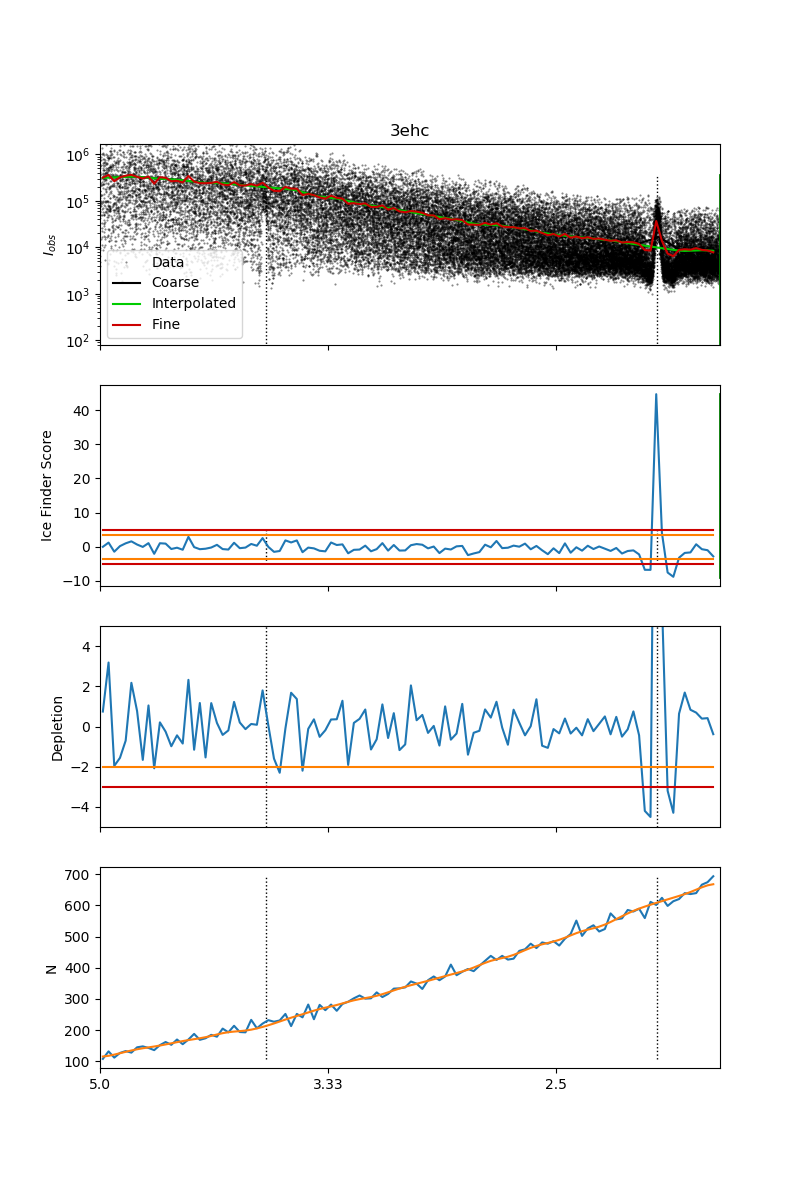

Supplement: Supplementary file 3 [file d-77-00540-sup3.zip › IceBiasingImages/3ehc.png]

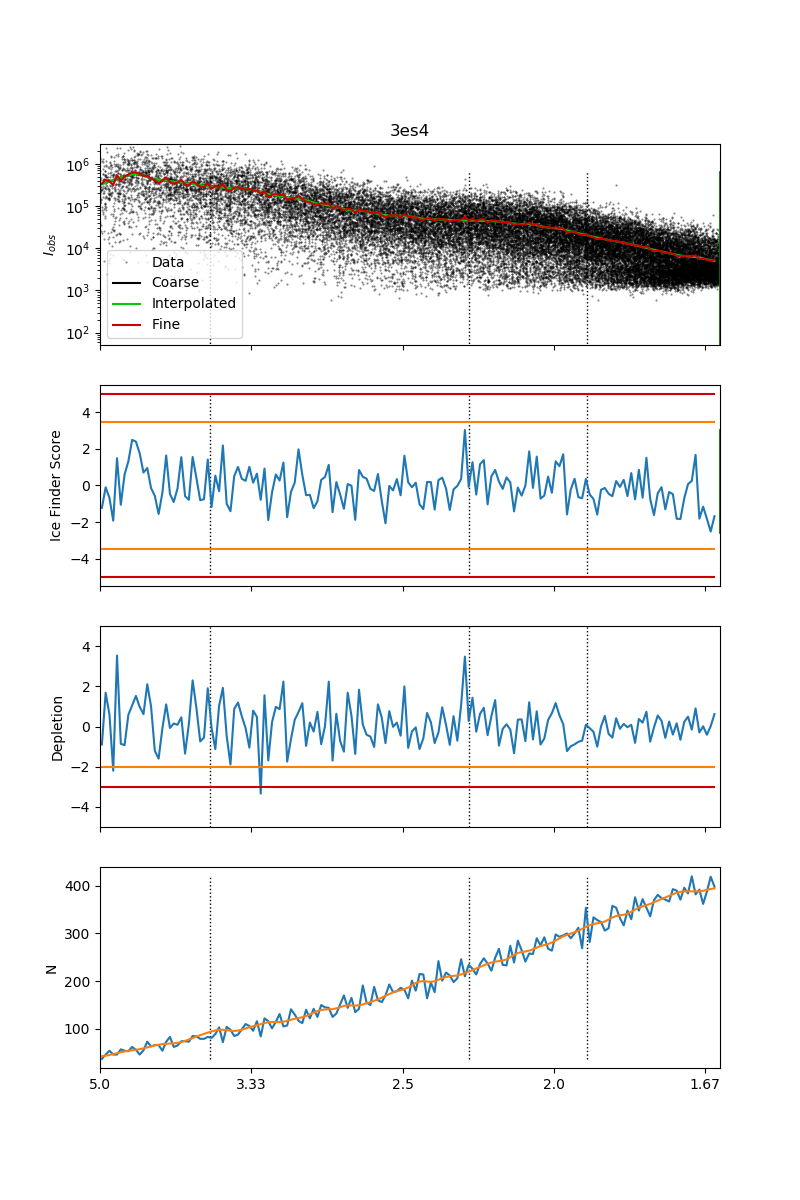

Supplement: Supplementary file 3 [file d-77-00540-sup3.zip › IceBiasingImages/3es4.png]

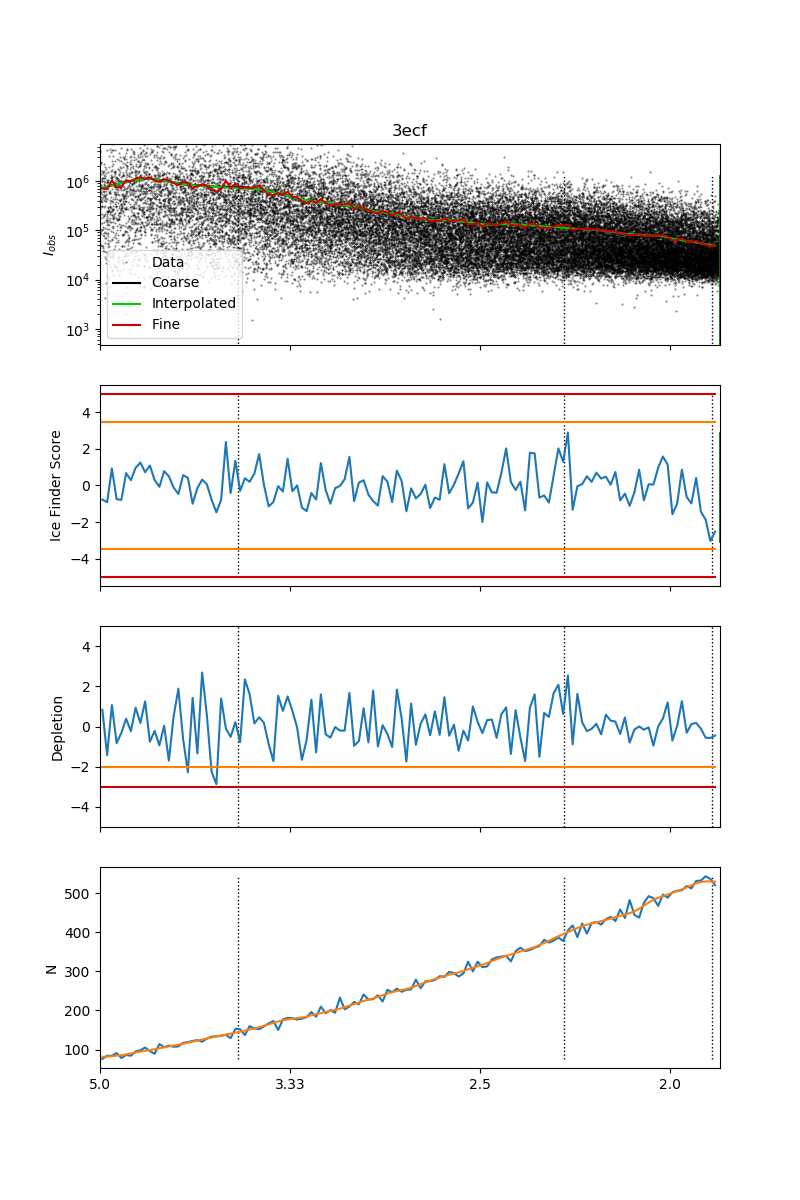

Supplement: Supplementary file 3 [file d-77-00540-sup3.zip › IceBiasingImages/3ecf.png]

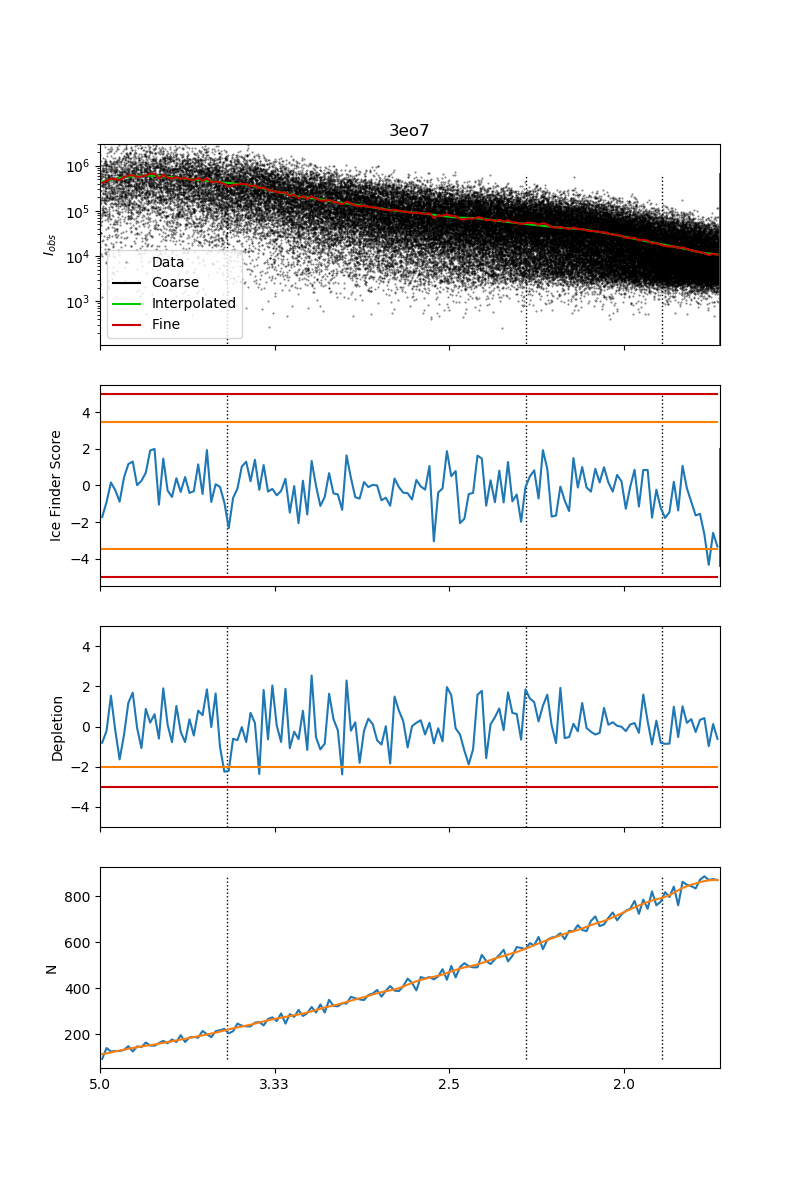

Supplement: Supplementary file 3 [file d-77-00540-sup3.zip › IceBiasingImages/3eo7.png]

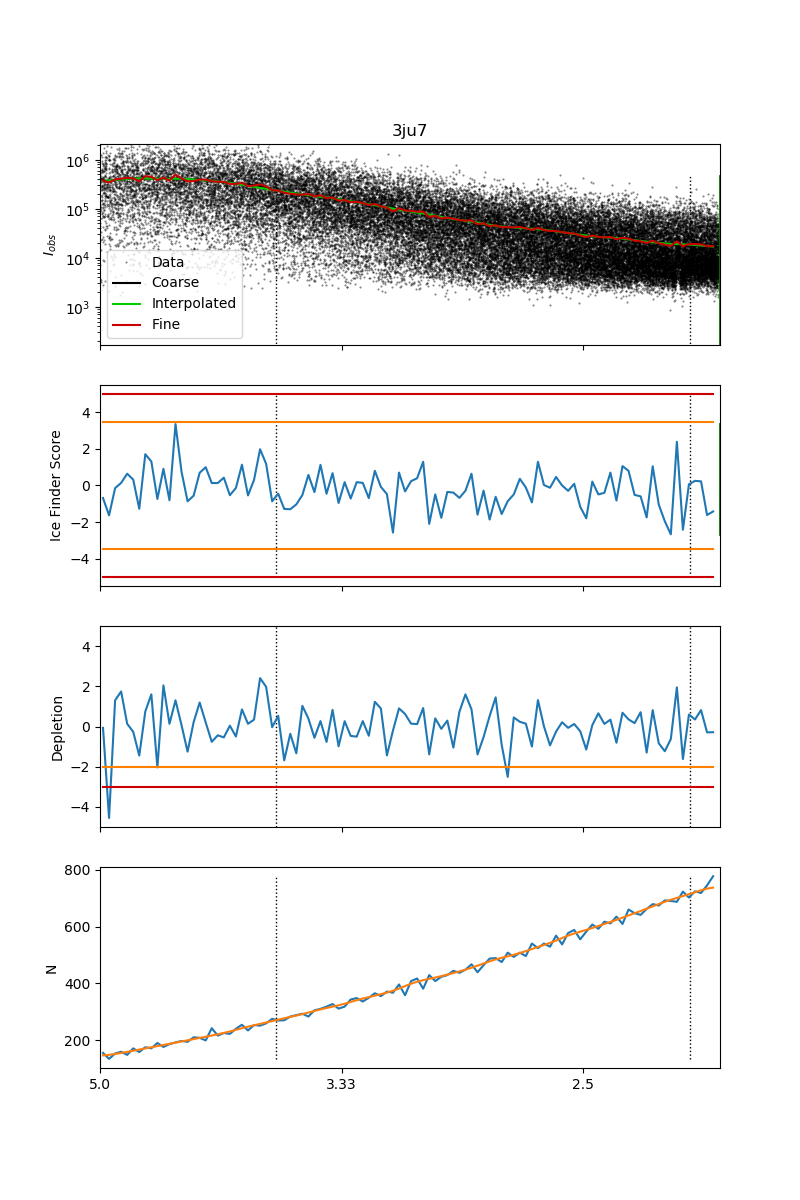

Supplement: Supplementary file 3 [file d-77-00540-sup3.zip › IceBiasingImages/3ju7.png]

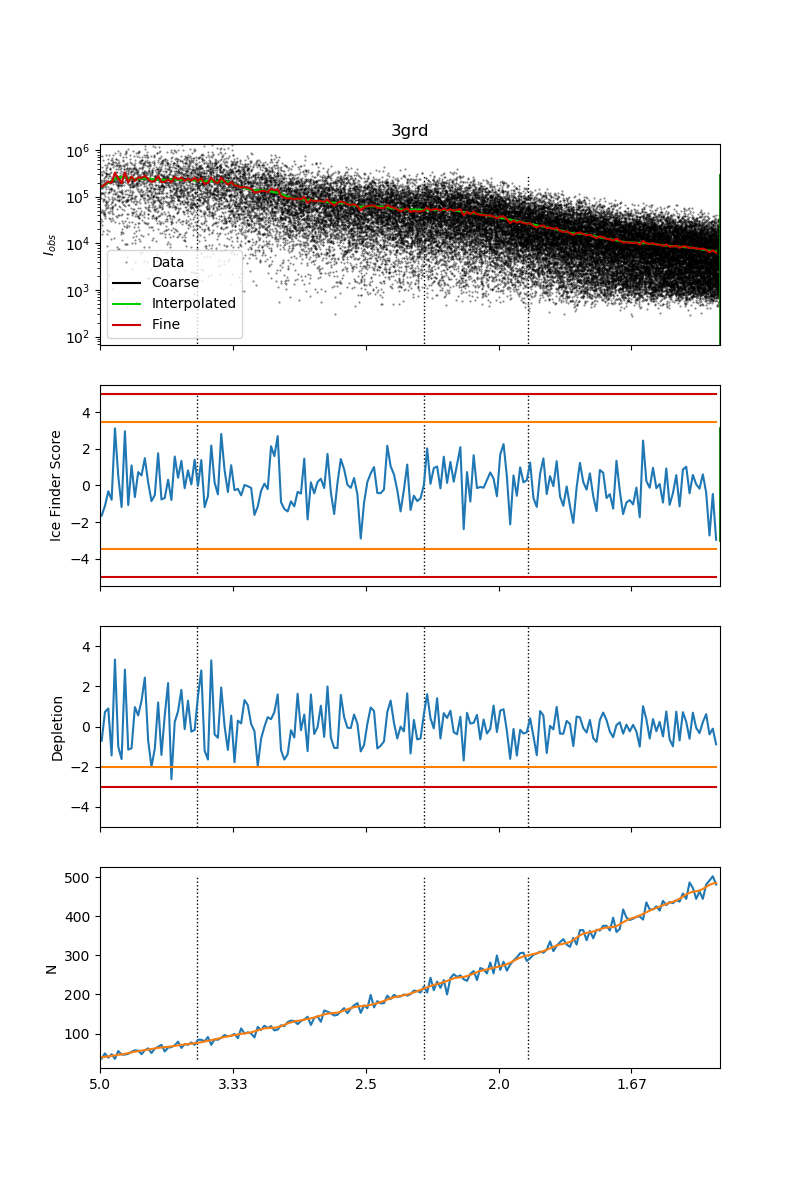

Supplement: Supplementary file 3 [file d-77-00540-sup3.zip › IceBiasingImages/3grd.png]

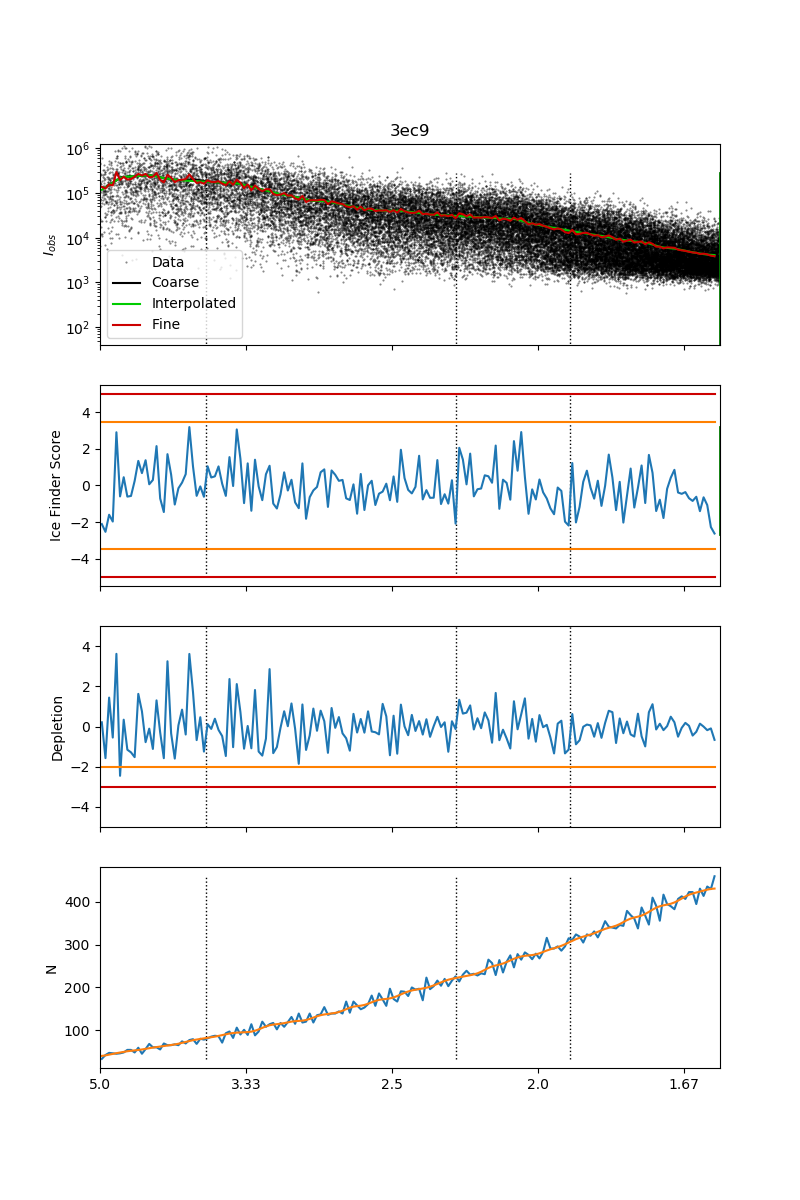

Supplement: Supplementary file 3 [file d-77-00540-sup3.zip › IceBiasingImages/3ec9.png]

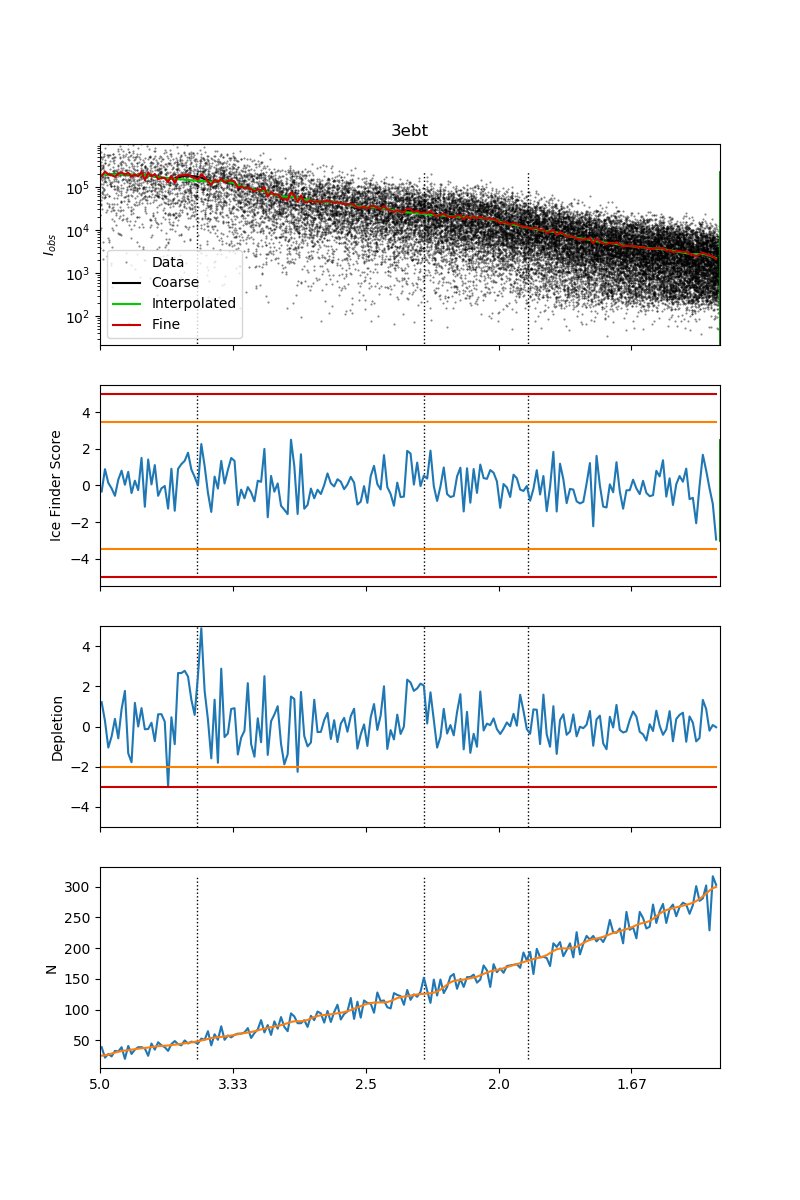

Supplement: Supplementary file 3 [file d-77-00540-sup3.zip › IceBiasingImages/3ebt.png]

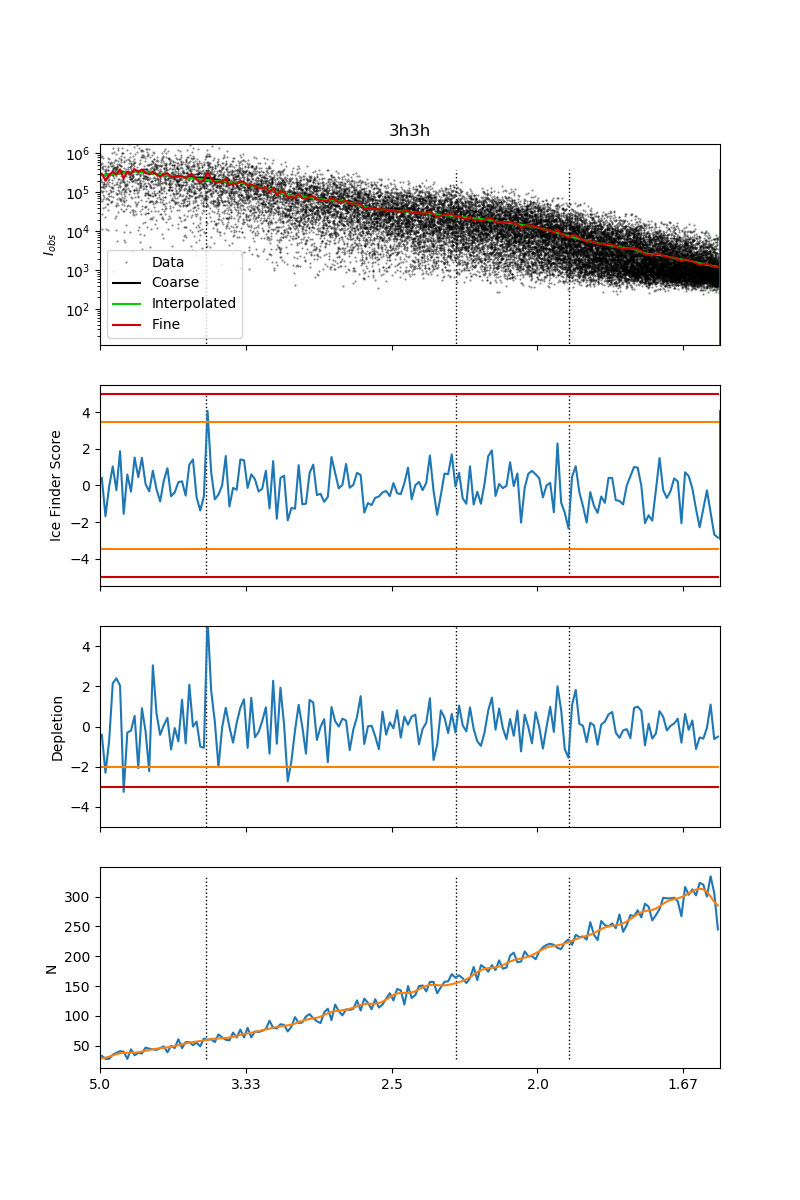

Supplement: Supplementary file 3 [file d-77-00540-sup3.zip › IceBiasingImages/3h3h.png]

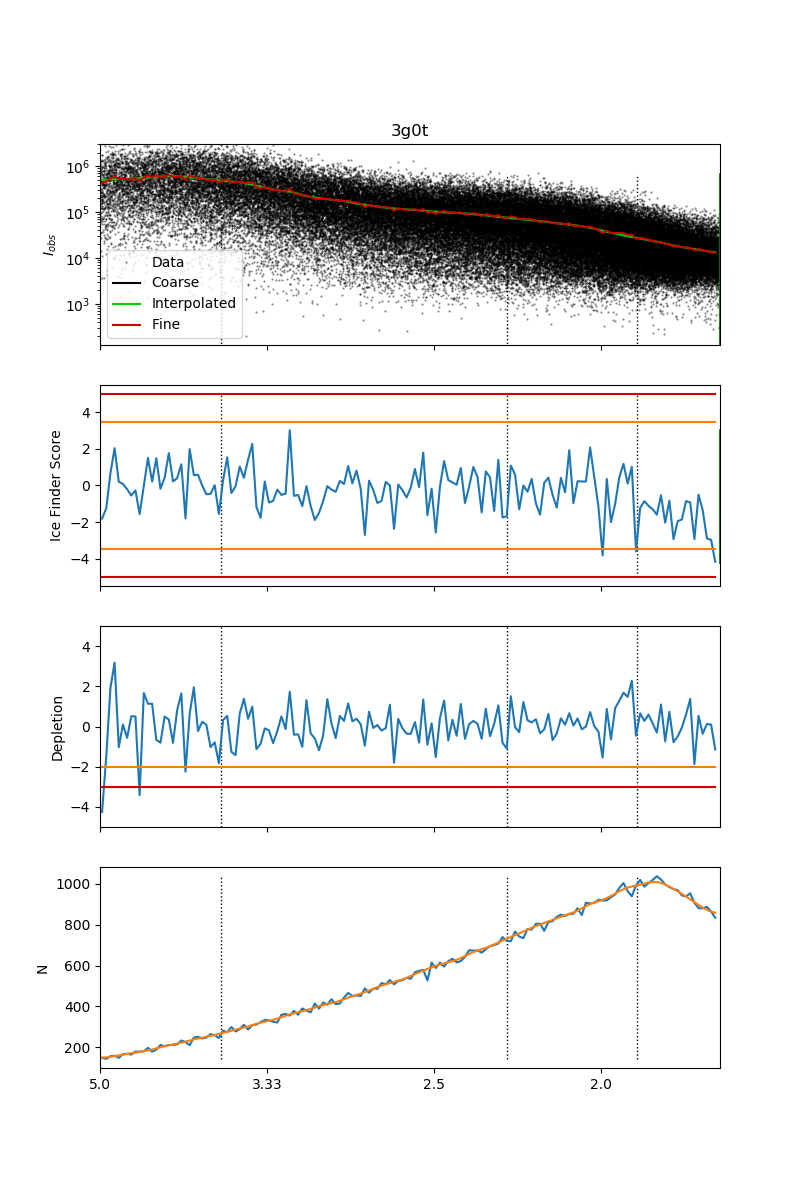

Supplement: Supplementary file 3 [file d-77-00540-sup3.zip › IceBiasingImages/3g0t.png]

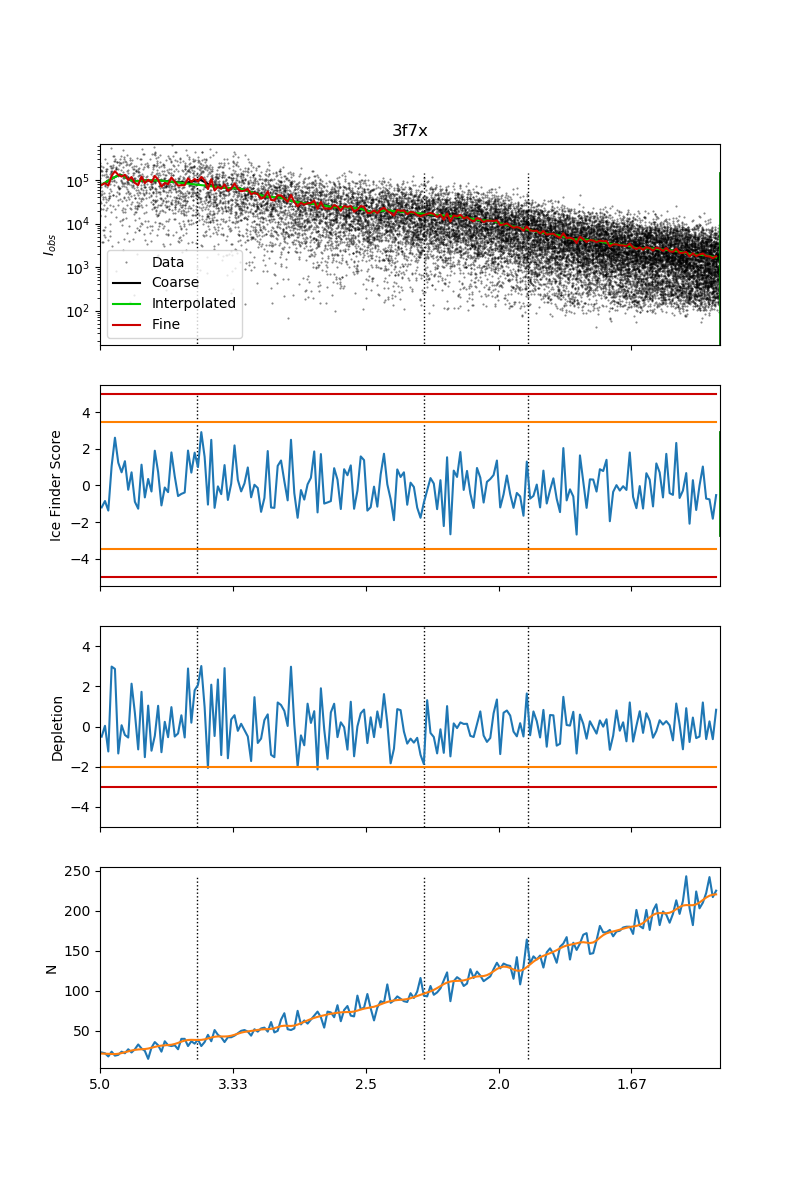

Supplement: Supplementary file 3 [file d-77-00540-sup3.zip › IceBiasingImages/3f7x.png]

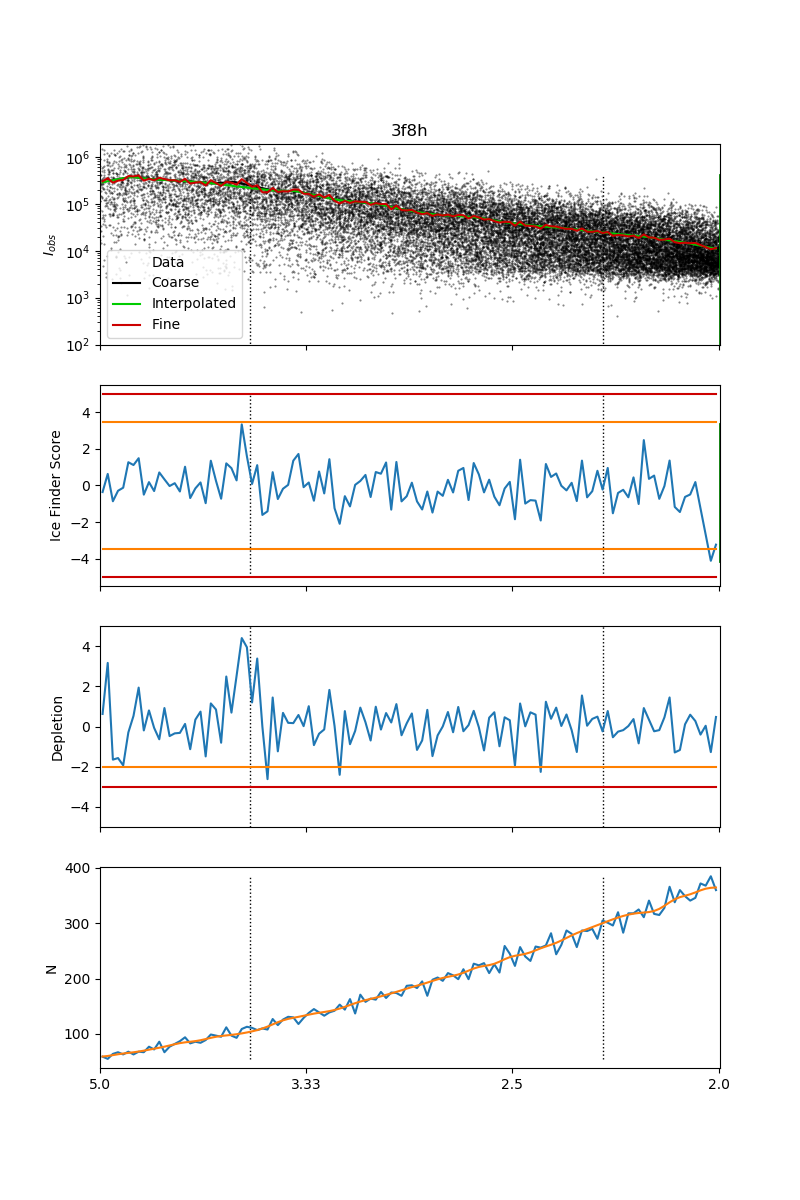

Supplement: Supplementary file 3 [file d-77-00540-sup3.zip › IceBiasingImages/3f8h.png]

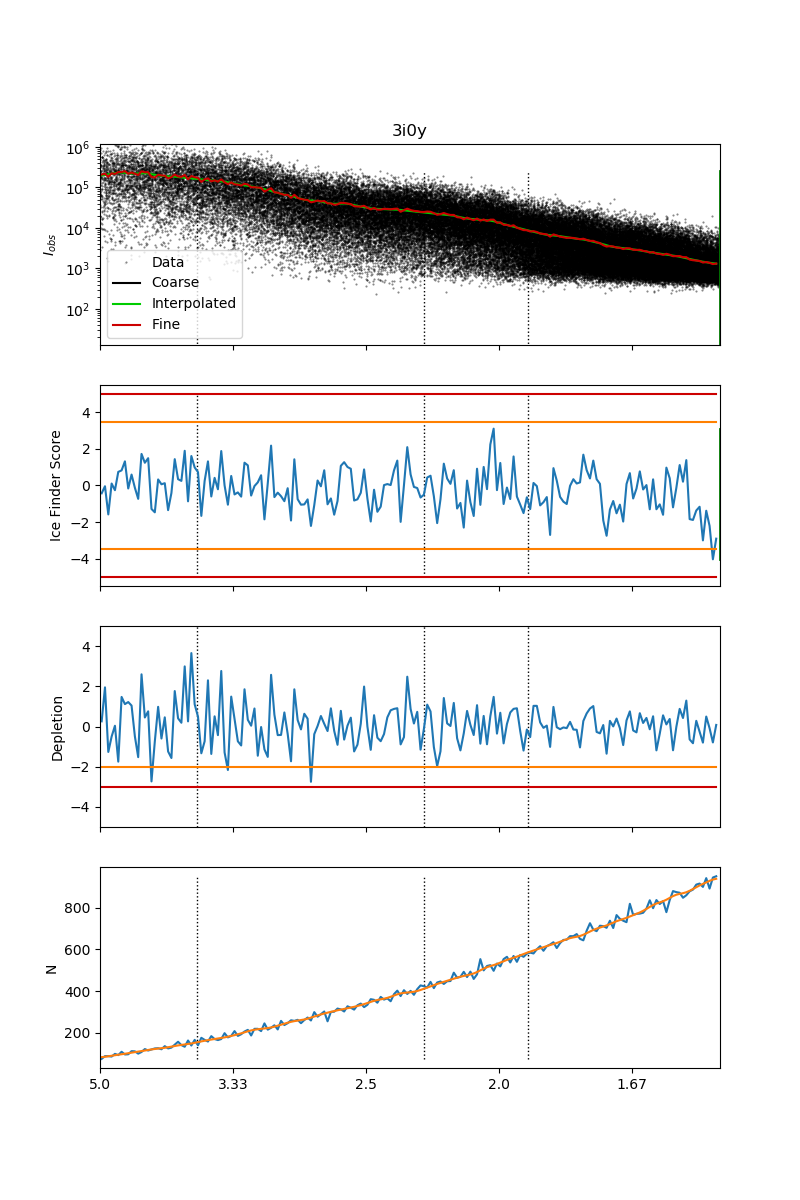

Supplement: Supplementary file 3 [file d-77-00540-sup3.zip › IceBiasingImages/3i0y.png]

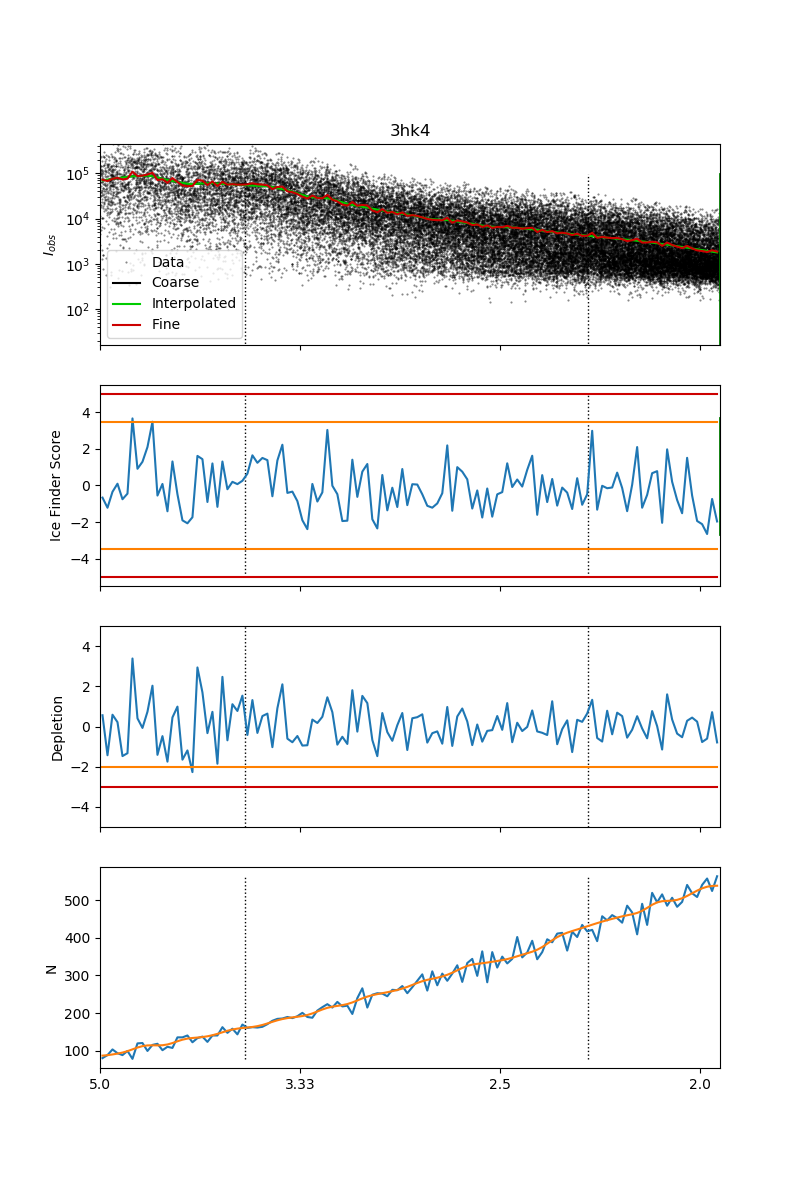

Supplement: Supplementary file 3 [file d-77-00540-sup3.zip › IceBiasingImages/3hk4.png]

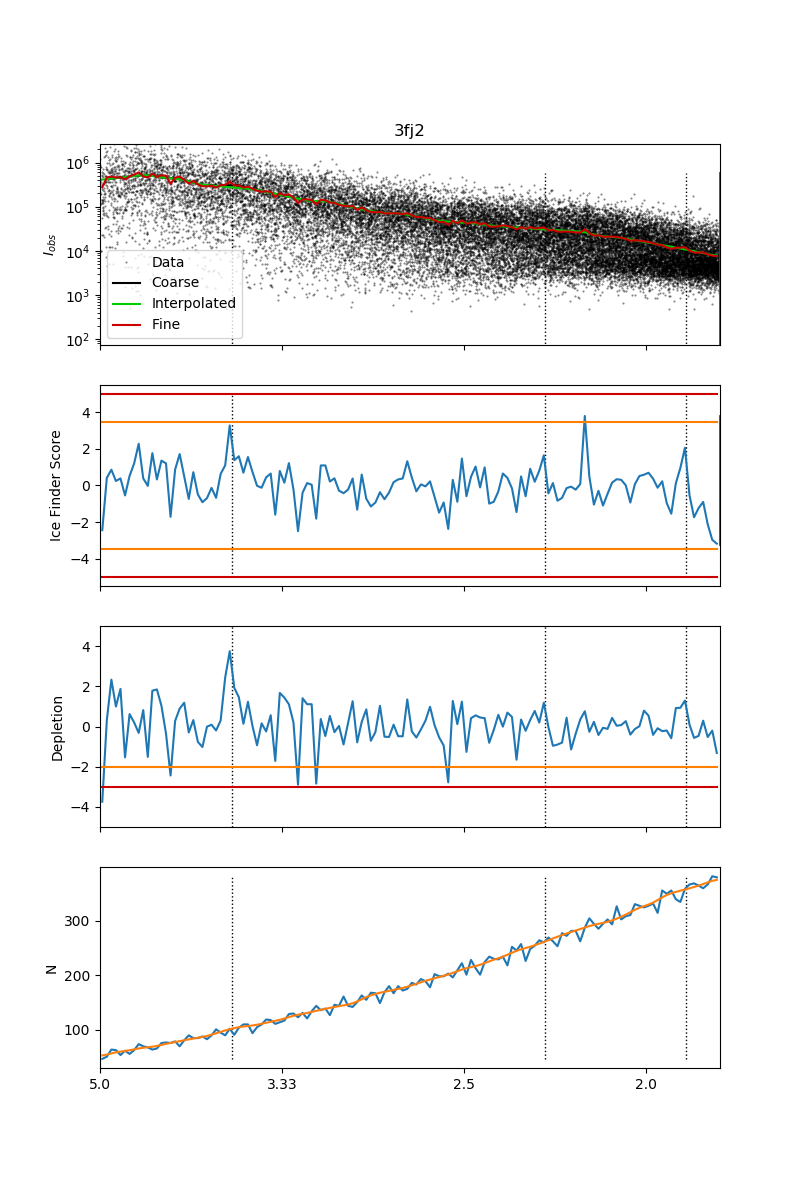

Supplement: Supplementary file 3 [file d-77-00540-sup3.zip › IceBiasingImages/3fj2.png]

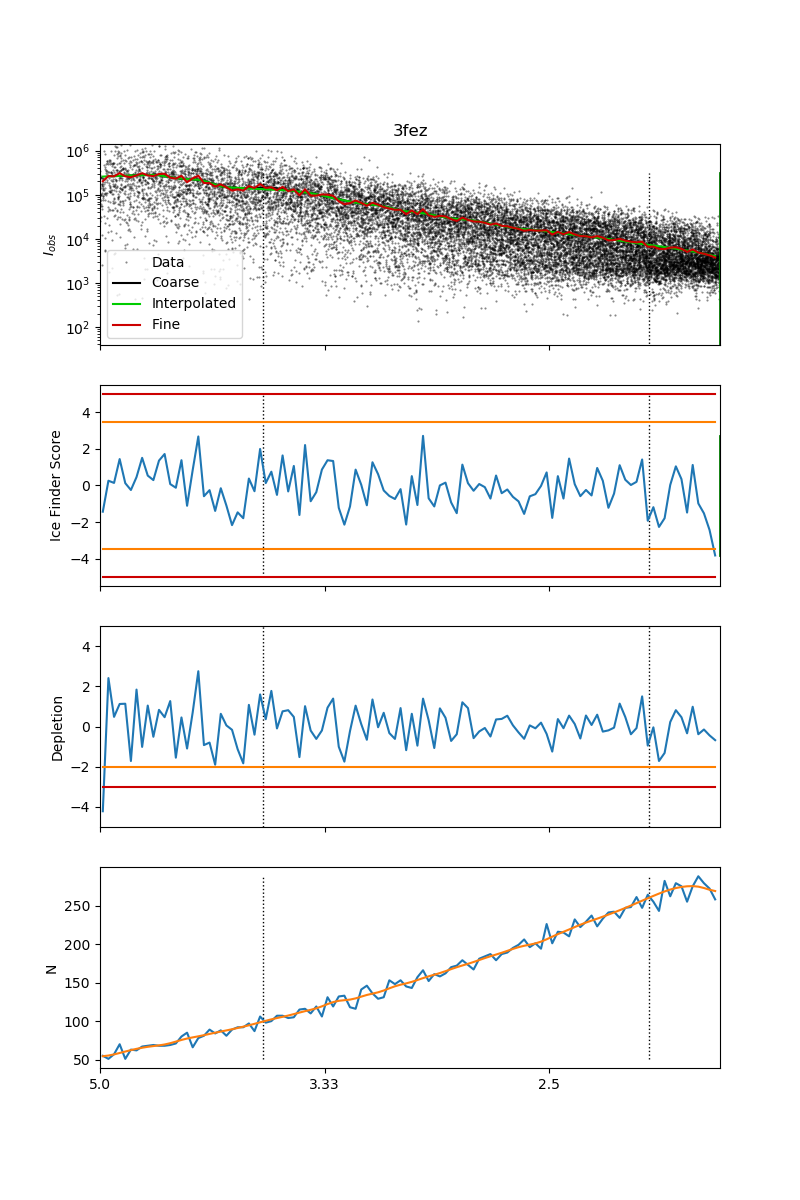

Supplement: Supplementary file 3 [file d-77-00540-sup3.zip › IceBiasingImages/3fez.png]

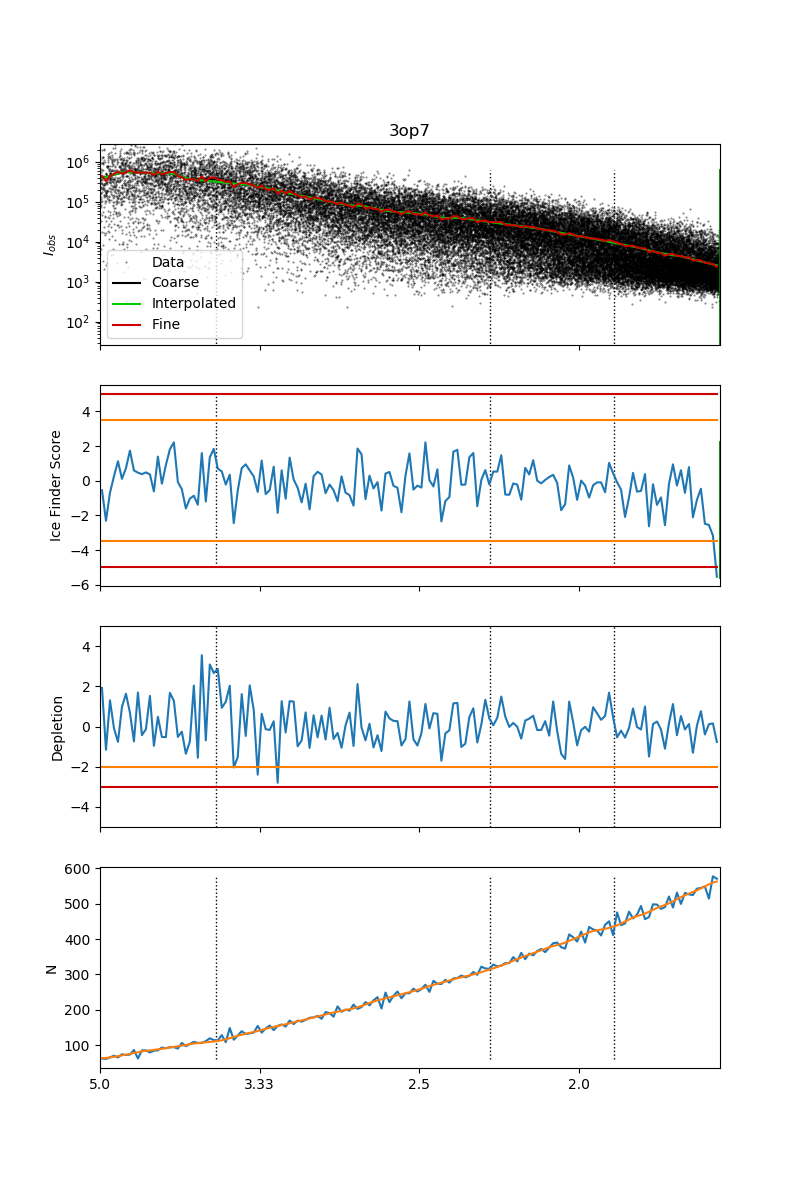

Supplement: Supplementary file 3 [file d-77-00540-sup3.zip › IceBiasingImages/3op7.png]

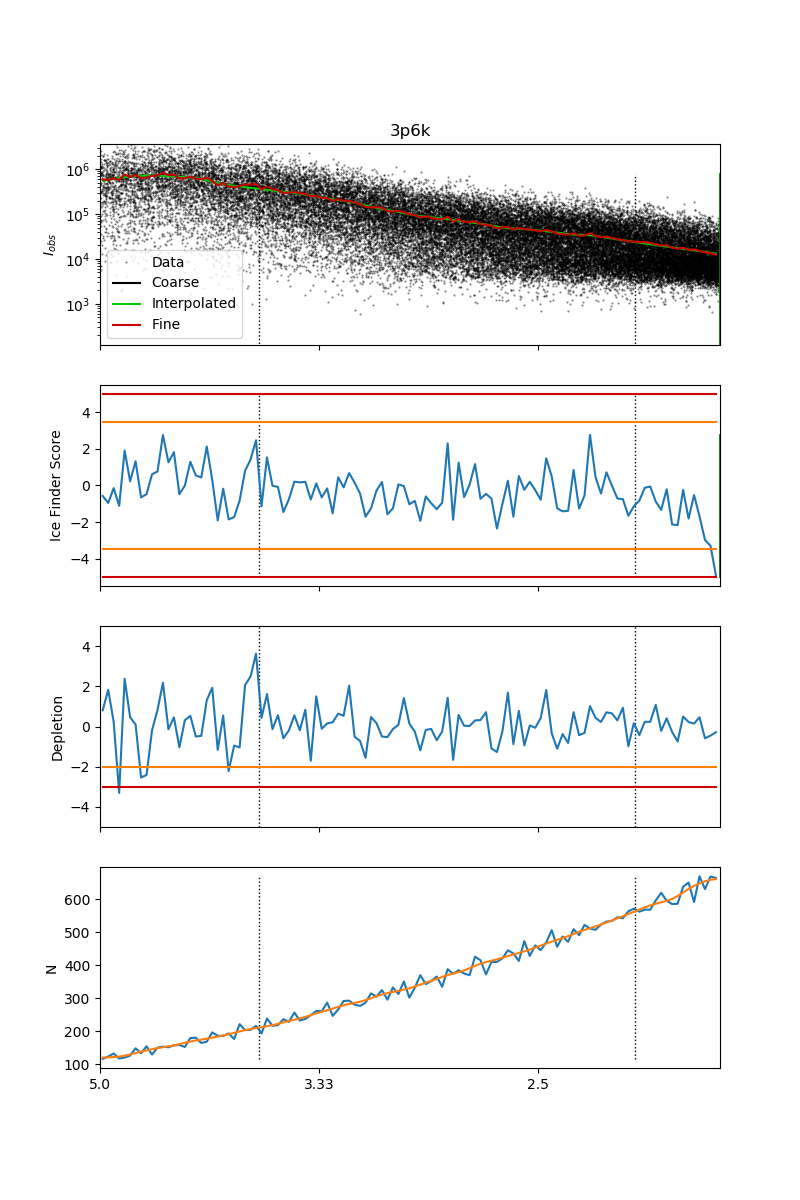

Supplement: Supplementary file 3 [file d-77-00540-sup3.zip › IceBiasingImages/3p6k.png]

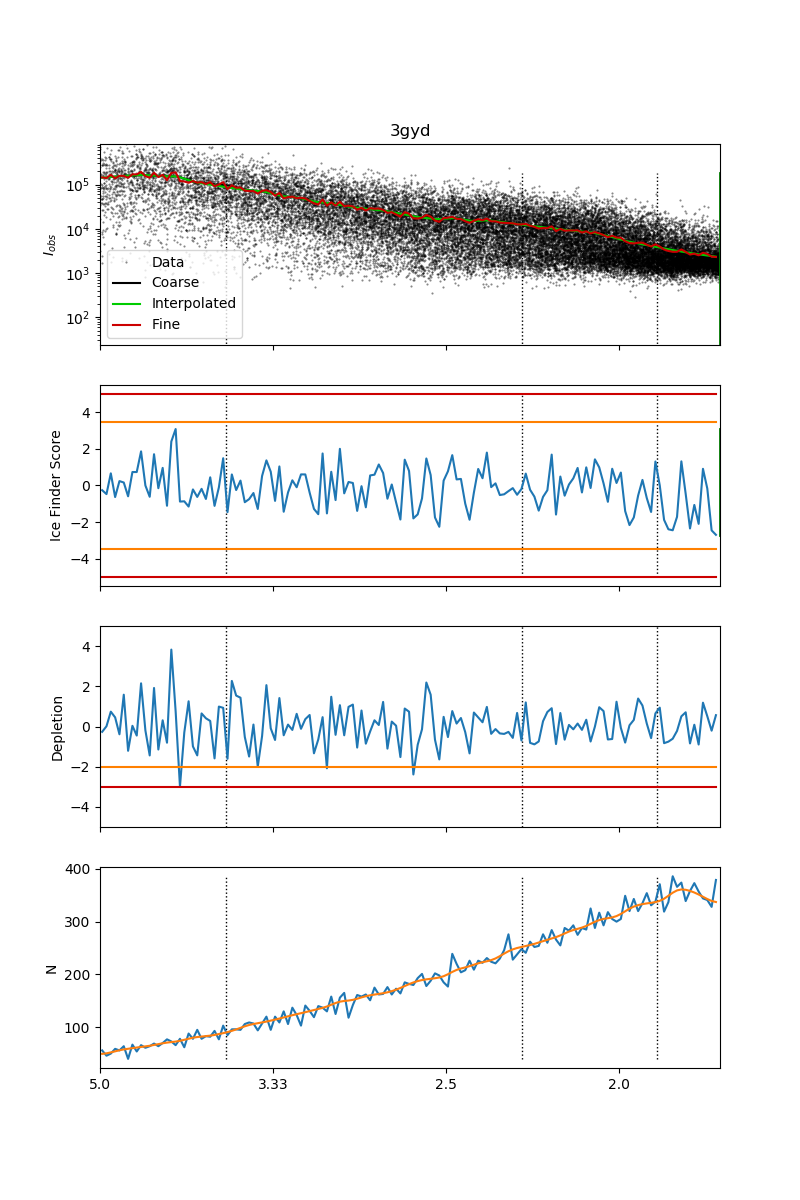

Supplement: Supplementary file 3 [file d-77-00540-sup3.zip › IceBiasingImages/3gyd.png]

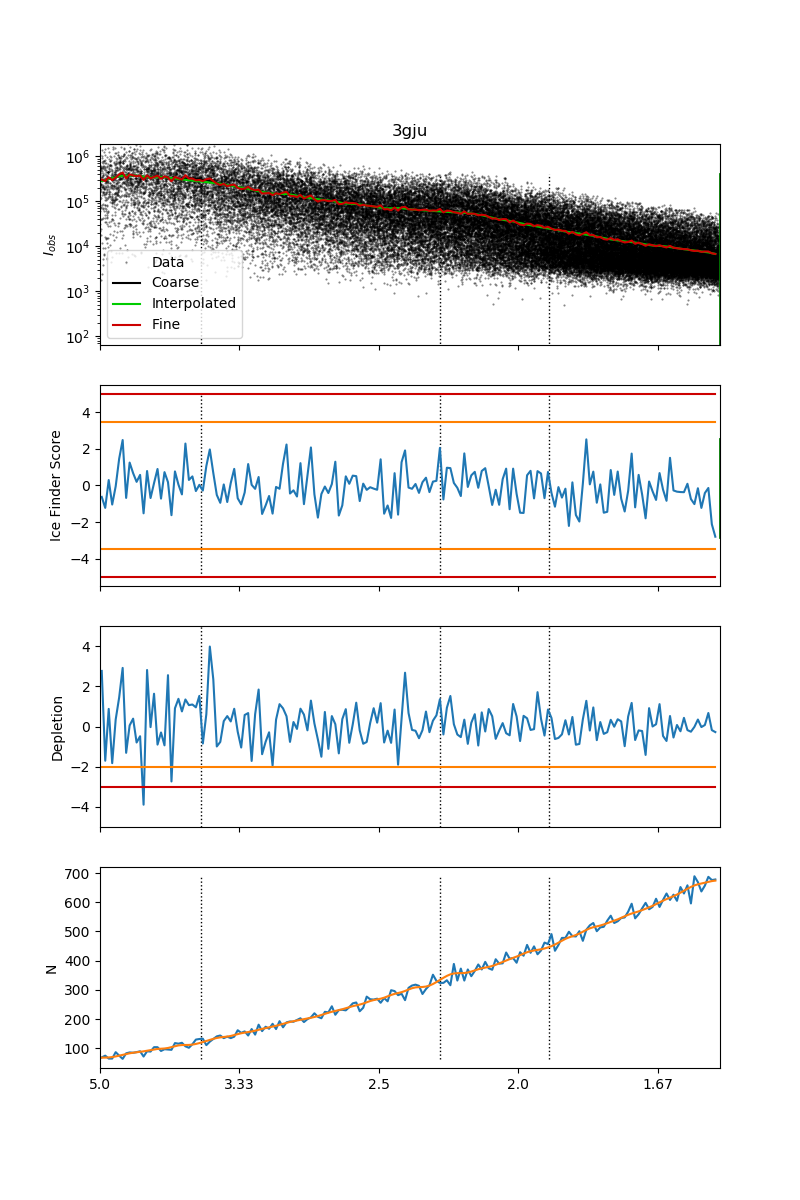

Supplement: Supplementary file 3 [file d-77-00540-sup3.zip › IceBiasingImages/3gju.png]

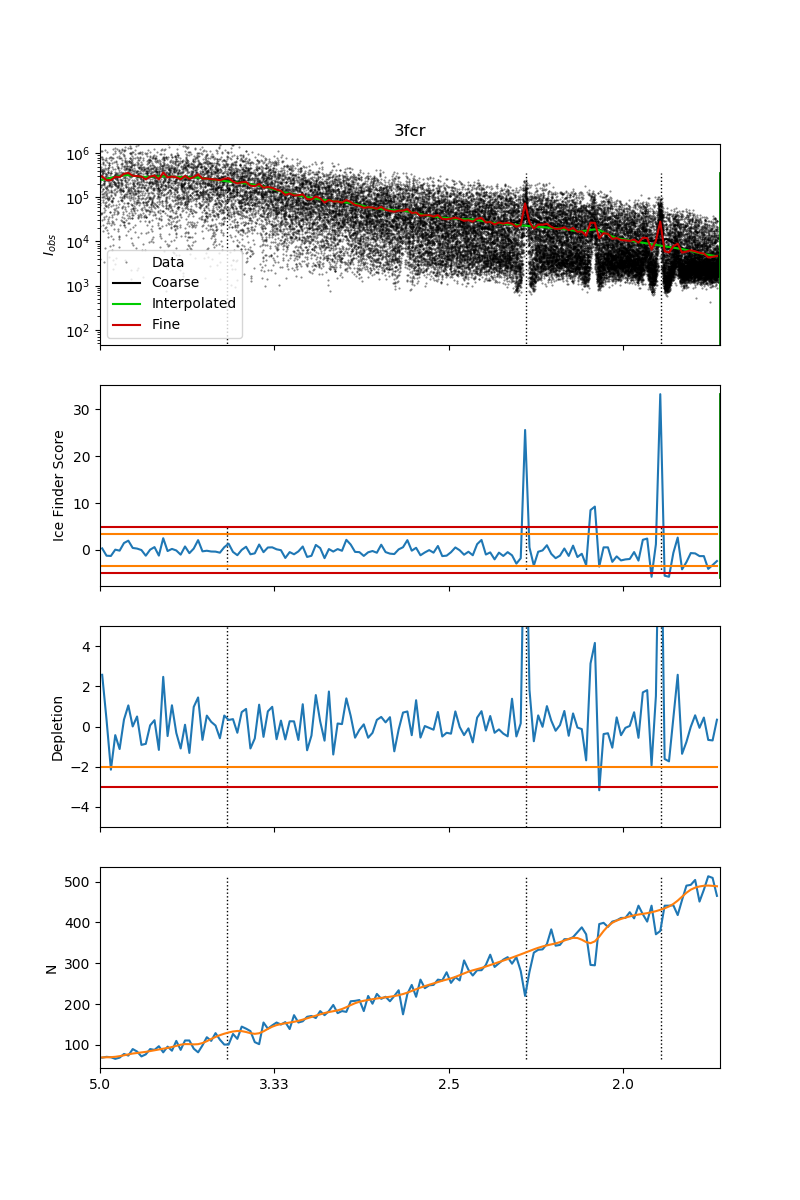

Supplement: Supplementary file 3 [file d-77-00540-sup3.zip › IceBiasingImages/3fcr.png]

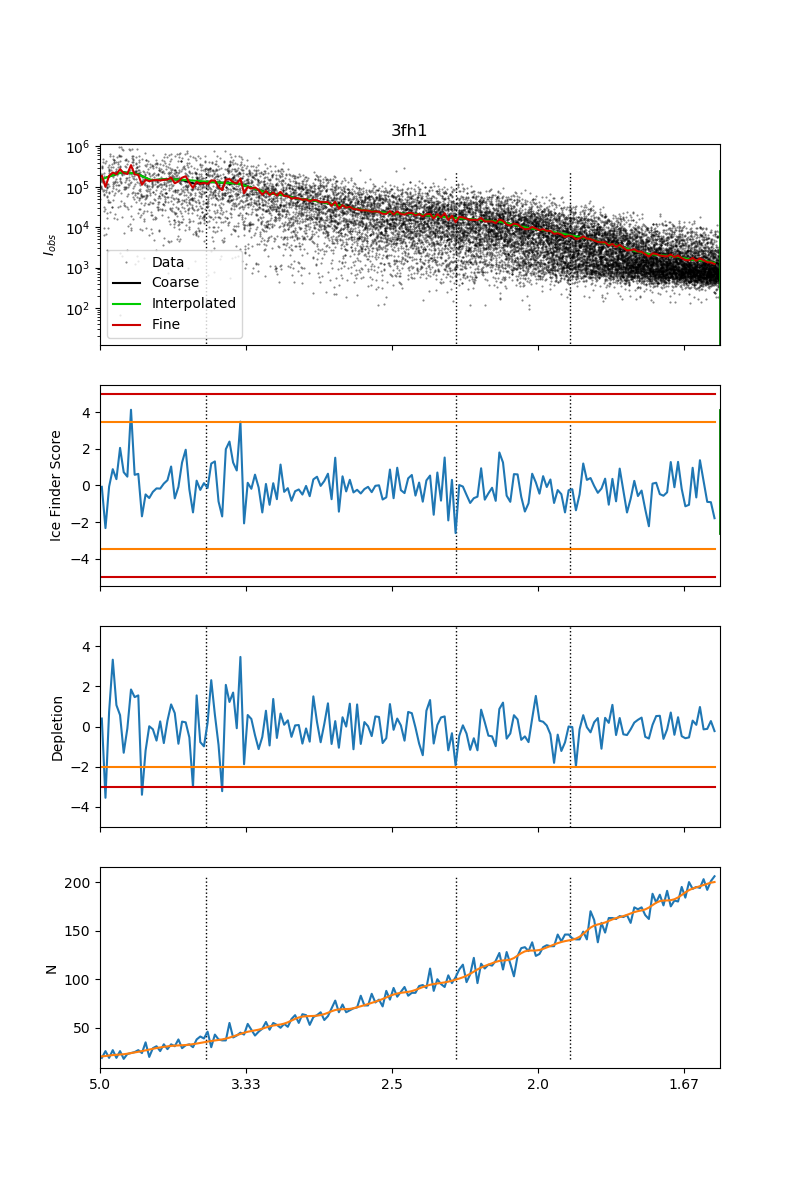

Supplement: Supplementary file 3 [file d-77-00540-sup3.zip › IceBiasingImages/3fh1.png]

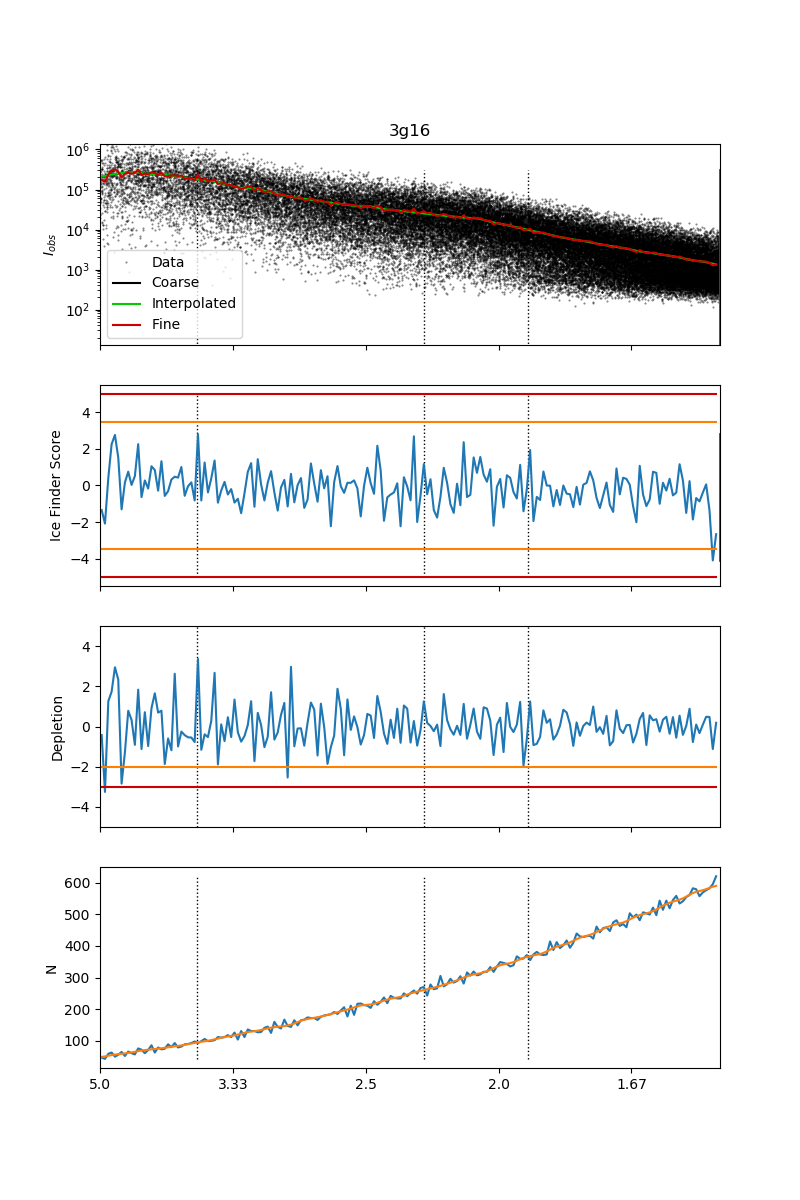

Supplement: Supplementary file 3 [file d-77-00540-sup3.zip › IceBiasingImages/3g16.png]

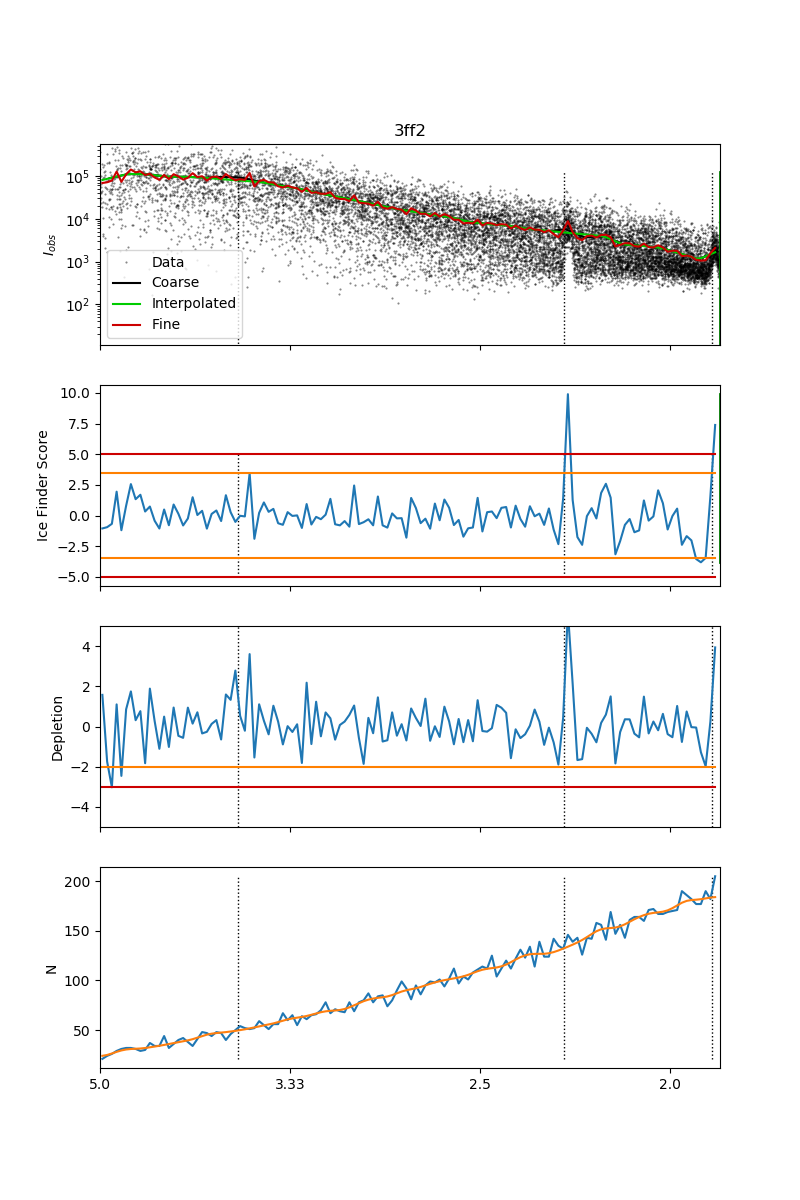

Supplement: Supplementary file 3 [file d-77-00540-sup3.zip › IceBiasingImages/3ff2.png]

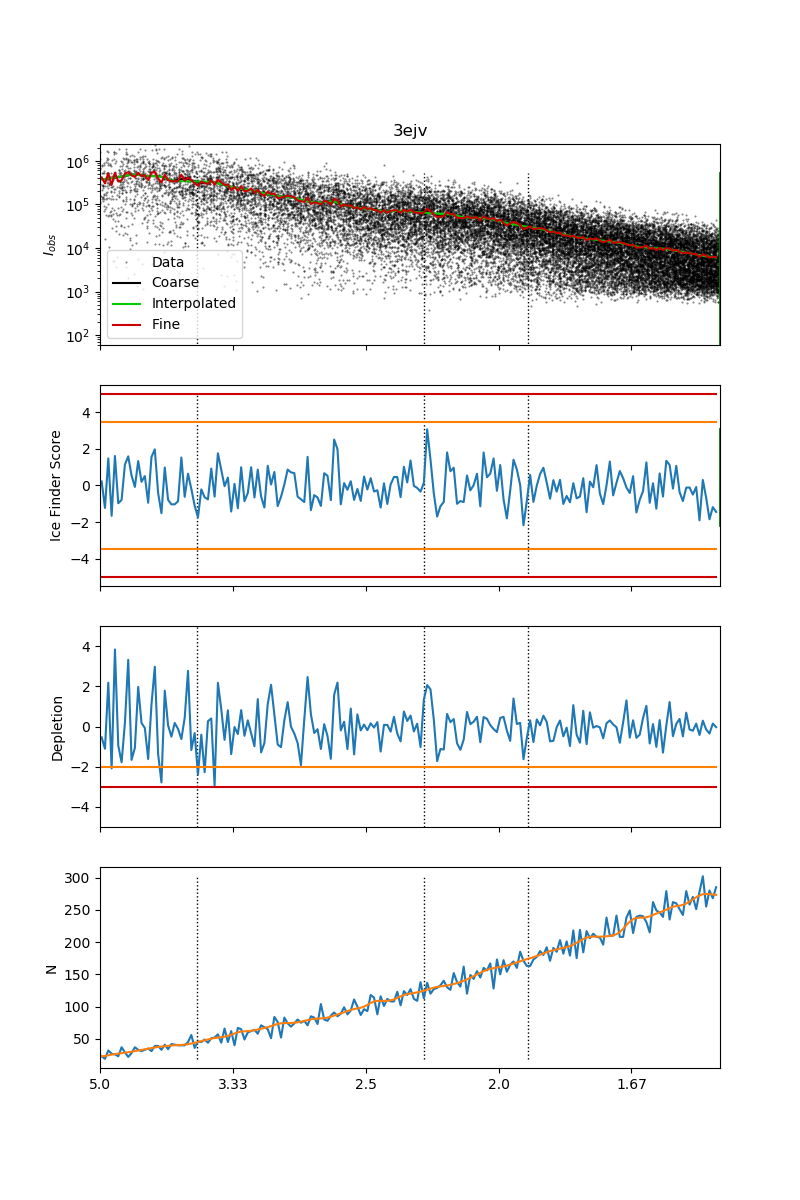

Supplement: Supplementary file 3 [file d-77-00540-sup3.zip › IceBiasingImages/3ejv.png]

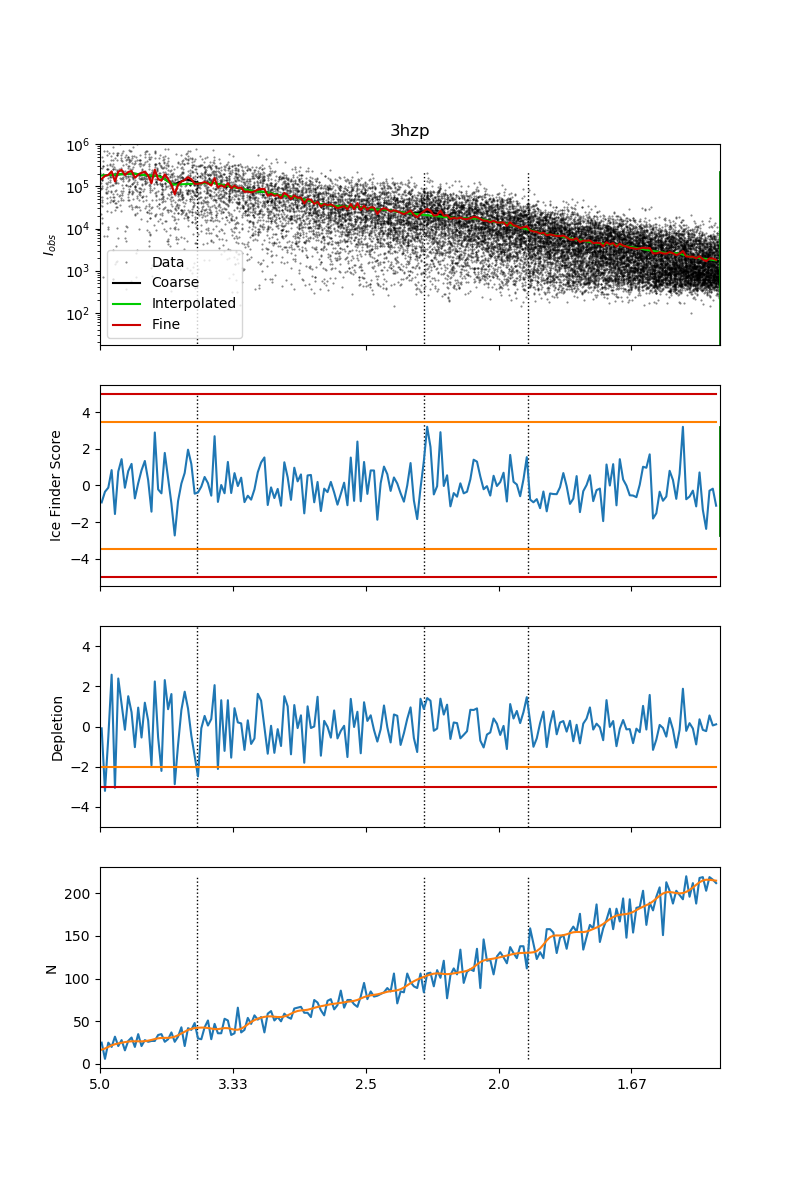

Supplement: Supplementary file 3 [file d-77-00540-sup3.zip › IceBiasingImages/3hzp.png]

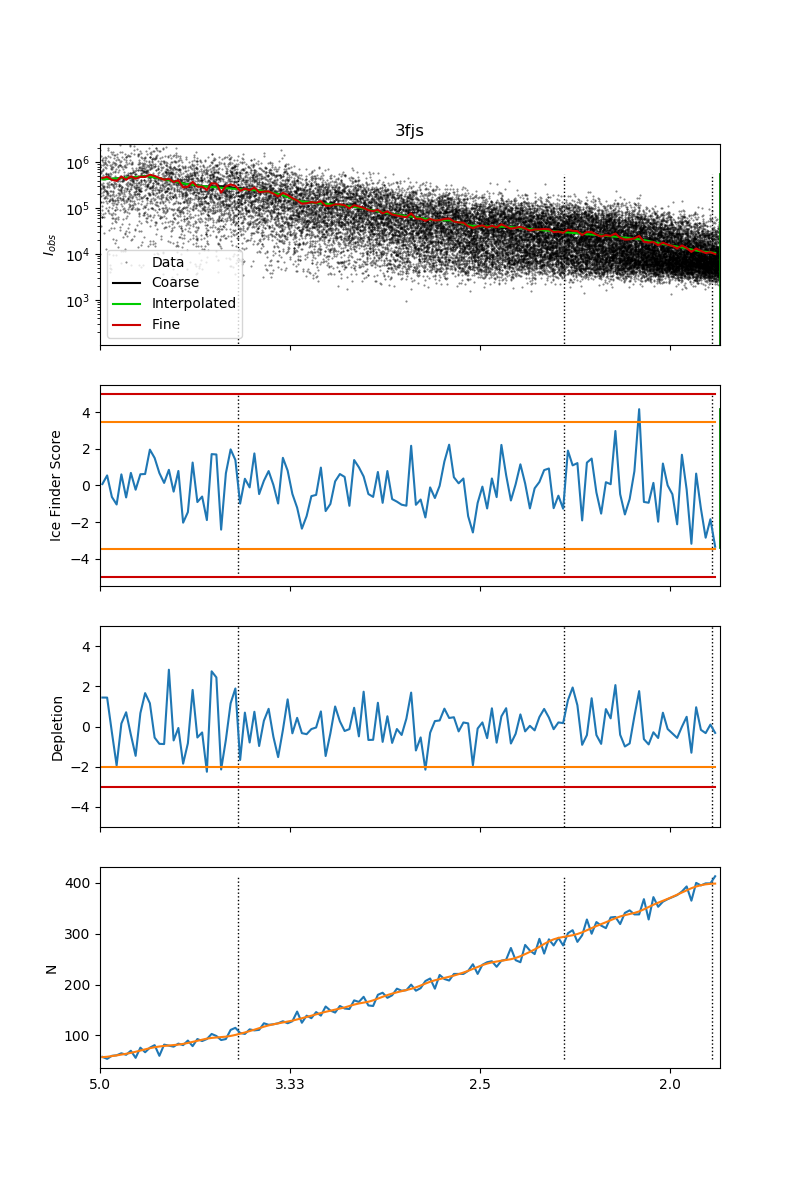

Supplement: Supplementary file 3 [file d-77-00540-sup3.zip › IceBiasingImages/3fjs.png]

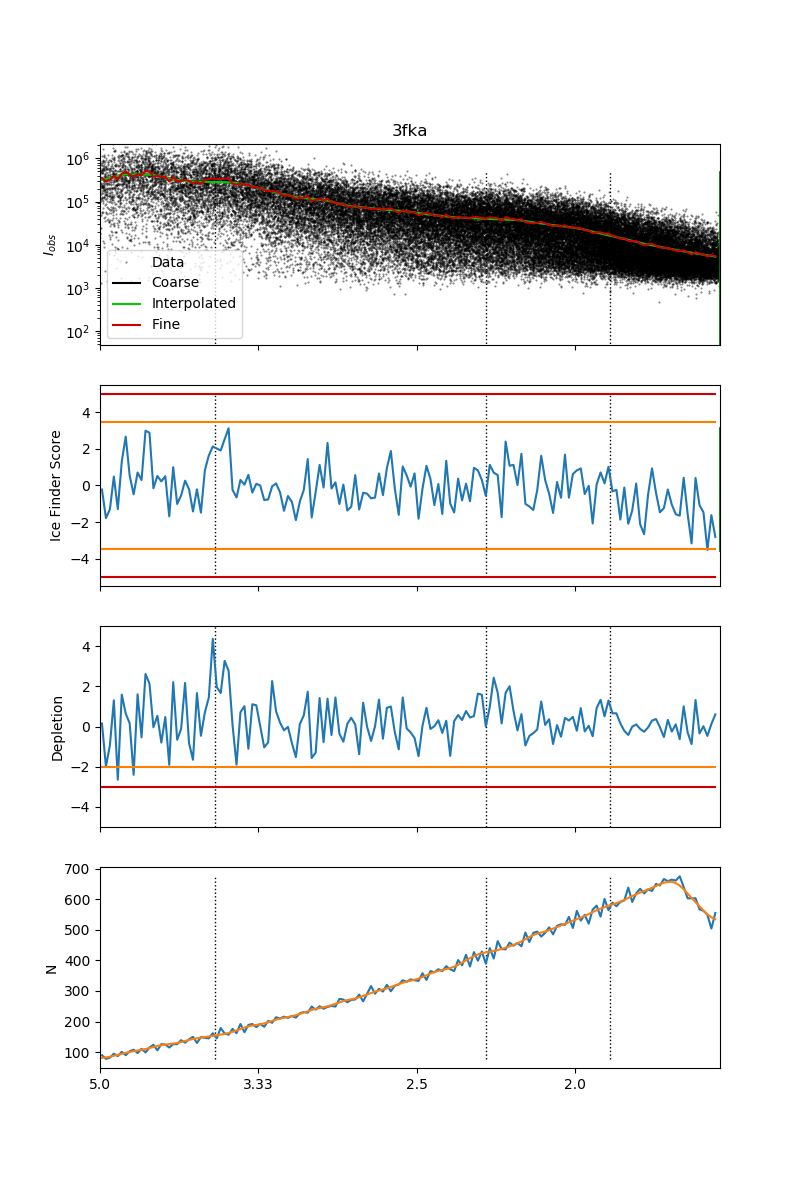

Supplement: Supplementary file 3 [file d-77-00540-sup3.zip › IceBiasingImages/3fka.png]

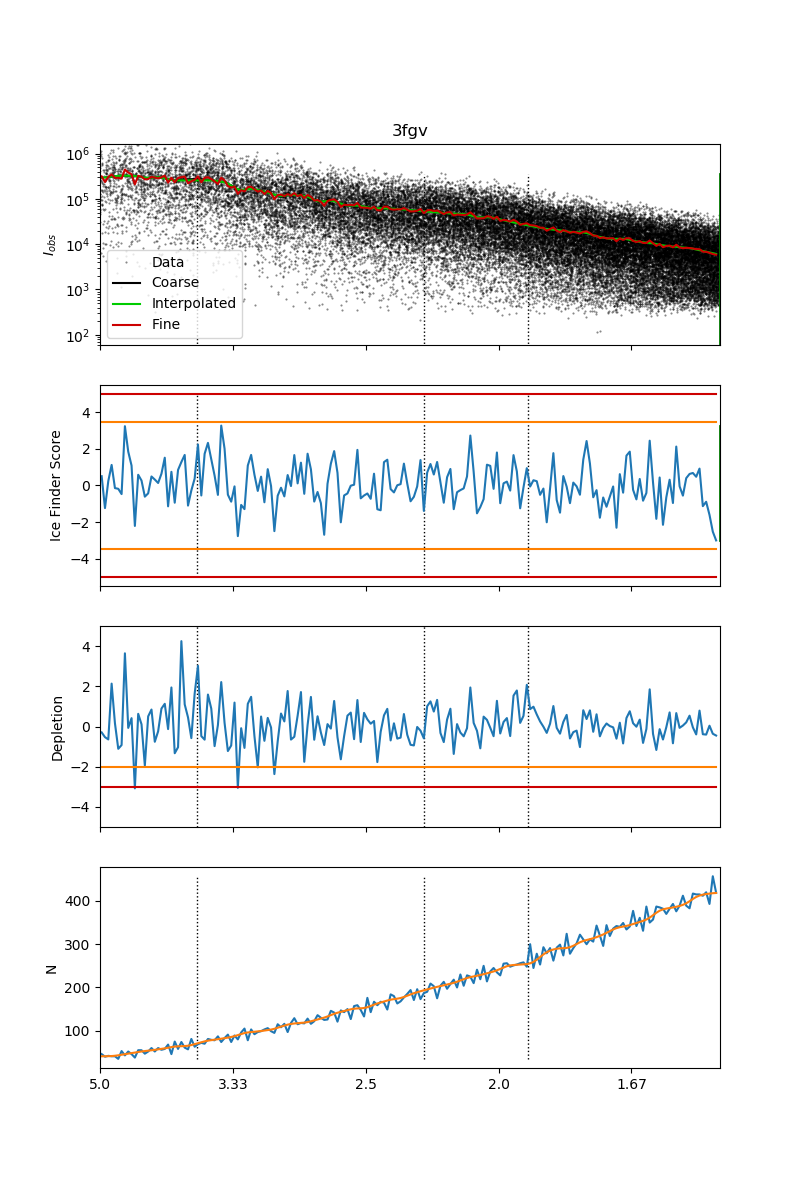

Supplement: Supplementary file 3 [file d-77-00540-sup3.zip › IceBiasingImages/3fgv.png]

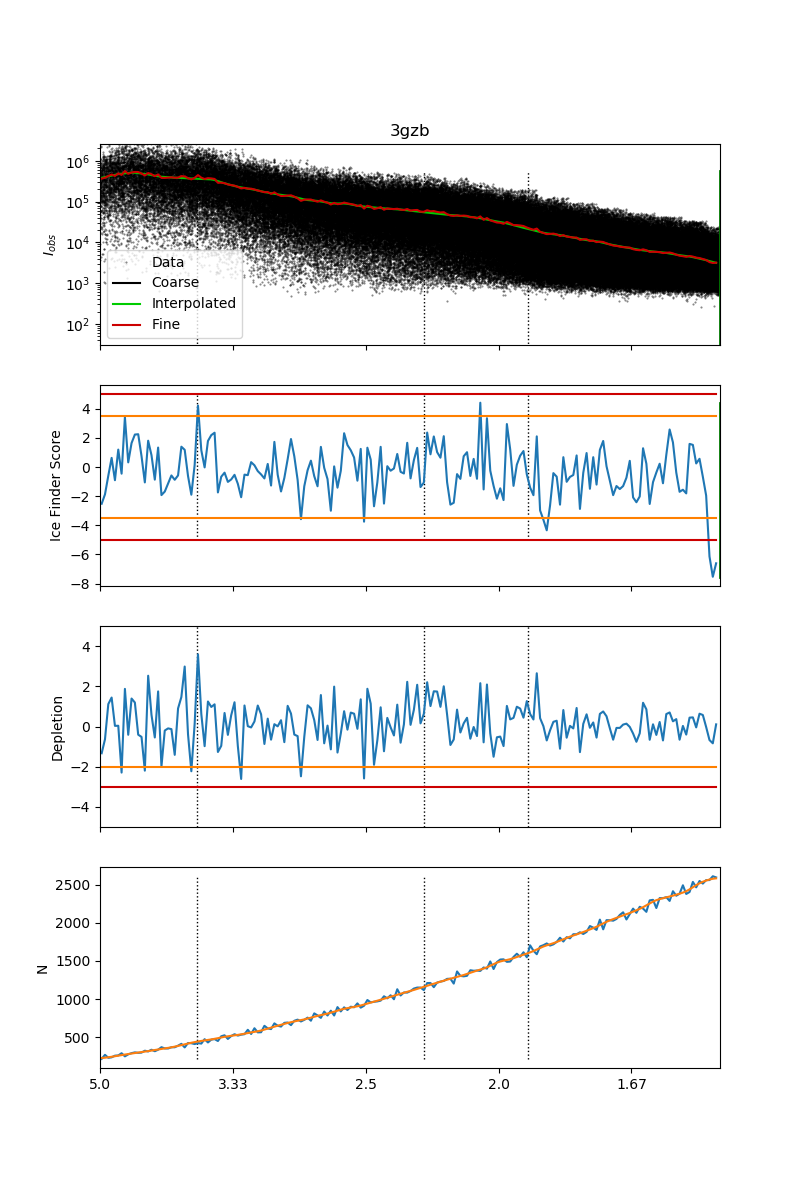

Supplement: Supplementary file 3 [file d-77-00540-sup3.zip › IceBiasingImages/3gzb.png]

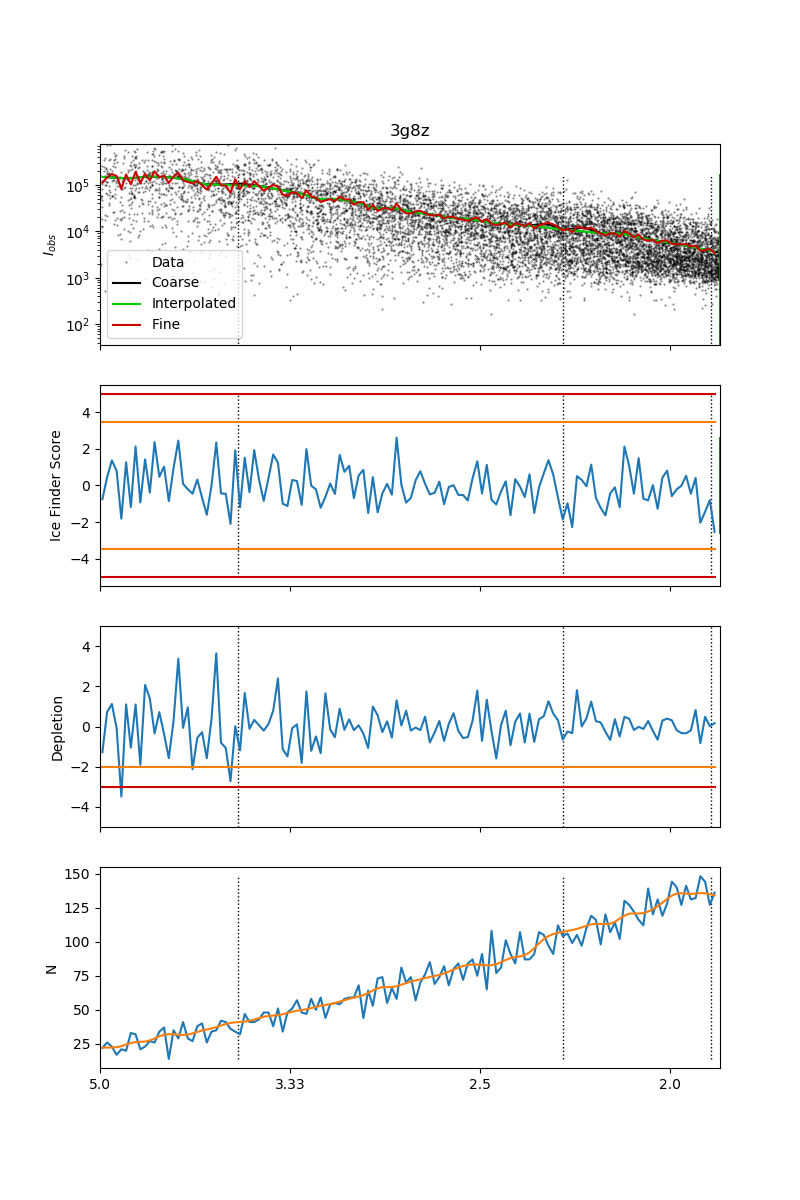

Supplement: Supplementary file 3 [file d-77-00540-sup3.zip › IceBiasingImages/3g8z.png]

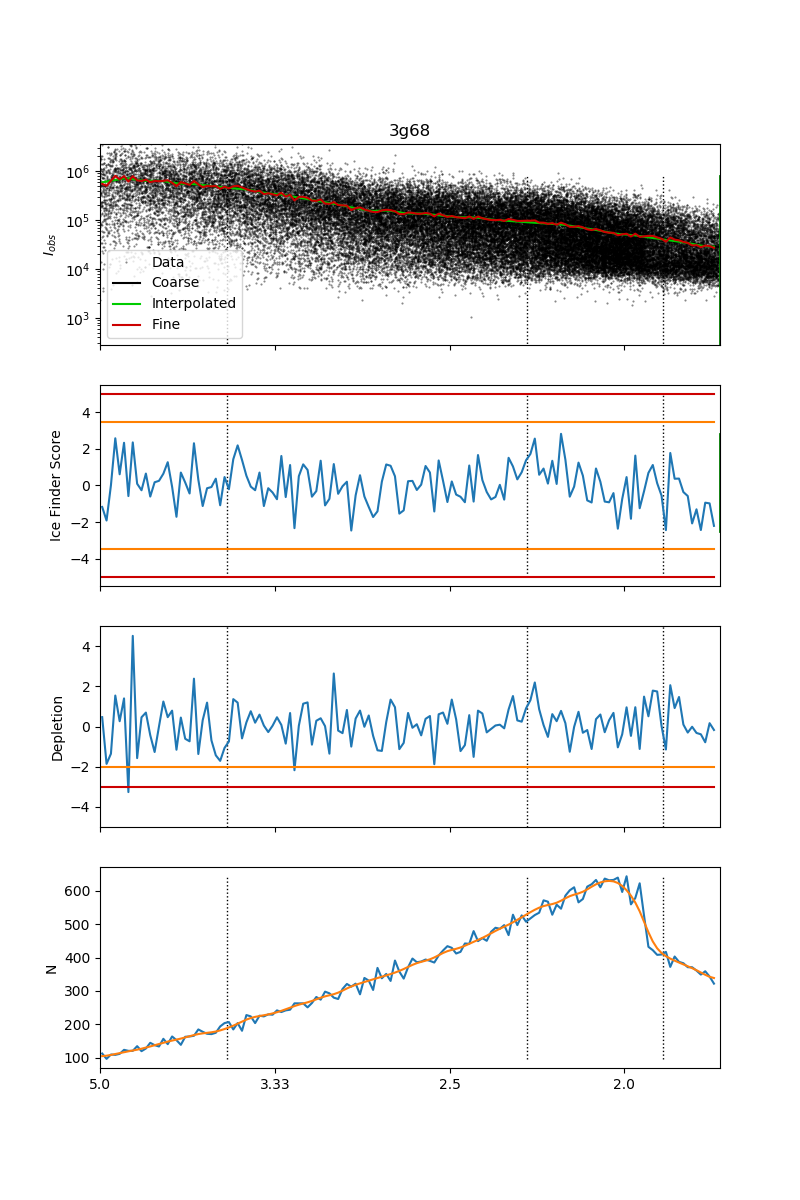

Supplement: Supplementary file 3 [file d-77-00540-sup3.zip › IceBiasingImages/3g68.png]

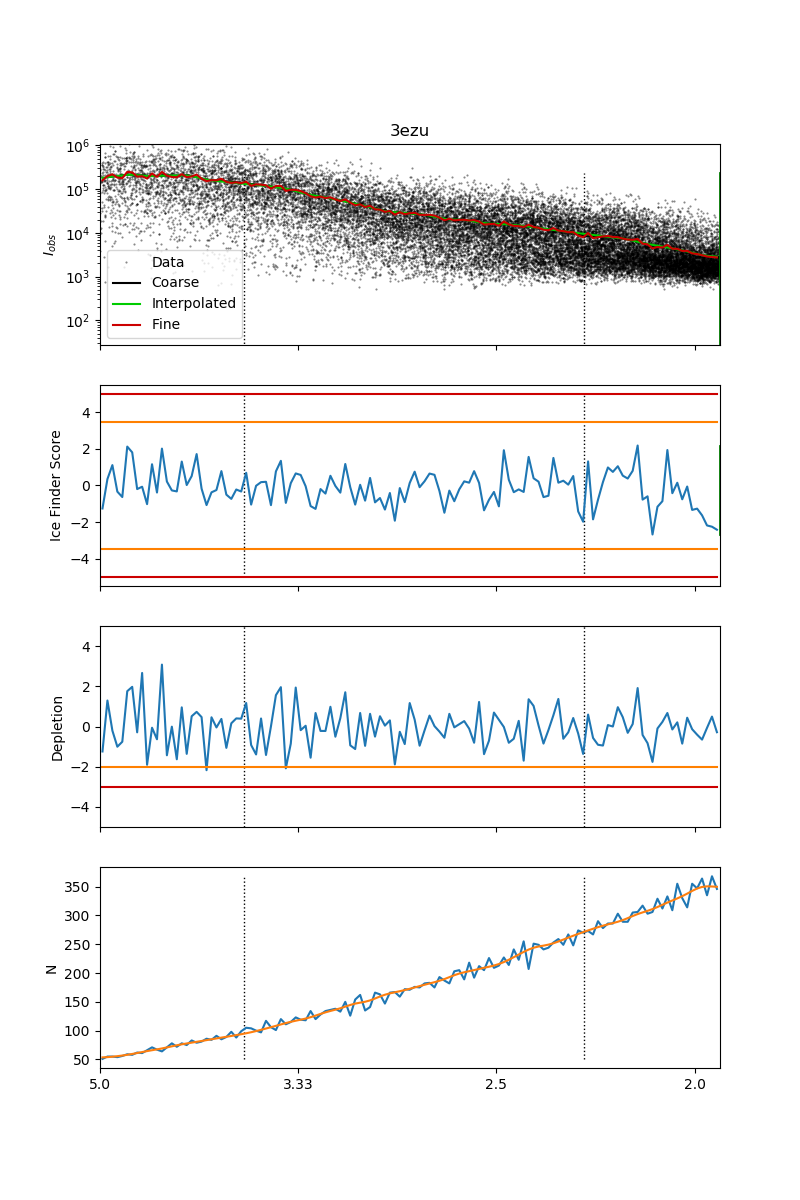

Supplement: Supplementary file 3 [file d-77-00540-sup3.zip › IceBiasingImages/3ezu.png]
